# Self‐Regeneration and Self‐Healing in DNA Origami Nanostructures

**DOI:** 10.1002/anie.202012986

**Published:** 2021-01-28

**Authors:** Michael Scheckenbach, Tom Schubert, Carsten Forthmann, Viktorija Glembockyte, Philip Tinnefeld

**Affiliations:** ^1^ Department of Chemistry and Center for NanoScience Ludwig-Maximilians-Universität München Butenandtstr. 5–13 81377 München Germany; ^2^ Department of Chemistry and Center for NanoScience Ludwig-Maximilians-Universität München Butenandtstr. 5–13 81377 München Germany

**Keywords:** DNA, molecular devices, nanotechnology, self-assembly, self-healing

## Abstract

DNA nanotechnology and advances in the DNA origami technique have enabled facile design and synthesis of complex and functional nanostructures. Molecular devices are, however, prone to rapid functional and structural degradation due to the high proportion of surface atoms at the nanoscale and due to complex working environments. Besides stabilizing mechanisms, approaches for the self‐repair of functional molecular devices are desirable. Here we exploit the self‐assembly and reconfigurability of DNA origami nanostructures to induce the self‐repair of defects of photoinduced and enzymatic damage. We provide examples of repair in DNA nanostructures showing the difference between unspecific self‐regeneration and damage specific self‐healing mechanisms. Using DNA origami nanorulers studied by atomic force and superresolution DNA PAINT microscopy, quantitative preservation of fluorescence properties is demonstrated with direct potential for improving nanoscale calibration samples.

## Graphical abstract

Self‐assembly and reconfigurability of DNA origami is exploited to realize self‐repairing functional nanodevices. A self‐regenerating brightness label is realized by constant exchange of labeling strands from solution. Structural self‐healing of a DNA origami nanoruler is achieved by excess of staple strands that exchange with enzymatically or photo‐induced damaged strands.

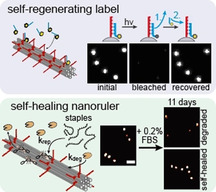

## Introduction

A molecular machine after Stoddart is defined as the assembly of a controlled number of molecular building units, that is designed to perform controlled motions as the output for an external stimulation (input).^[1]^ In the last two decades, a variety of nanoscale devices acting as molecular motors, switches, pumps or ratchets has been established.[^1^, ^2^, ^3^] Such nanodevices exhibit high functionality and increasing complexity driven by progress in different fields.[^4^, ^5^, ^6^] Especially, DNA nanotechnology and progress in DNA origami assemblies have enabled easy design and synthesis of unprecedented complex nanostructures with high yields.[^7^, ^8^, ^9^] With DNA nanotechnology, the integration and the exact arrangement of a manifold of new functionalities are creating emerging potentials for drug delivery,[^10^, ^11^] nanophotonics^[12]^ and biosensing.^[13]^ These developments are reviving the dreams of early molecular nanotechnology including medical nanorobots that autonomously swarm through our bodies to detect and eliminate disease factors and sources of pain. One aspect that has yet caught little attention but will become increasingly important is the maintenance of autonomously working self‐assembled nanomachines and devices. Can we develop strategies to maintain the activity and functionality under conditions of wear, for example, in complex chemical environments, in the presence of degrading enzymes or under the influence of photodamage in light‐driven devices?

Fundamentally, molecular devices are prone to rapid degradation and loss of functionality due to the high proportion of surface atoms and molecules.^[14]^ The importance of self‐repair is underscored by the sophistication and complexity of nature's molecular machineries and their accompanying self‐healing abilities and self‐repairing systems. Almost every atom in our body is frequently replaced and the biomolecules in our cells undergo constant self‐regeneration. On the molecular level, chemical stress and unintended side reactions need to be contained and repaired. DNA repair systems, for example, constantly deal with the repair of thousands of lesions, abasic sites and oxidized guanosines.[^15^, ^16^] During photosynthesis, Photosystem II calls for immediate response to oxidative side reactions requiring recognition of damaged D1 subunits and their replacement.[^17^, ^18^] These and many other examples from nature teach us, that in our strive for artificial molecular machines with sustainability and similar functionalities as their natural counterparts we should also consider dynamic strategies of how to compensate for loss of functionality.

For applications of functional DNA nanotechnology, research has focused mainly on the improvement of stabilization of DNA nanostructures in complex environments, for example, by coating or encapsulation of the structure or strengthening the backbone by covalent cross‐linking.[^19^, ^20^, ^21^, ^22^] The demand of self‐repairing functional nanostructures is just emerging. Recently, the stabilization of artificial DNA nanotubes in degrading conditions could be shown by incubation with intact DNA tiles forming the nanotubes.^[23]^ Self‐assembling nanostructures could simply be stabilized by the excess of intact building units. Another recent example is the design of a stable fluorescence single‐particle tracking label by exchanging transient labels in the form of short fluorescently labeled oligonucleotides.^[24]^

**Scheme 1:**
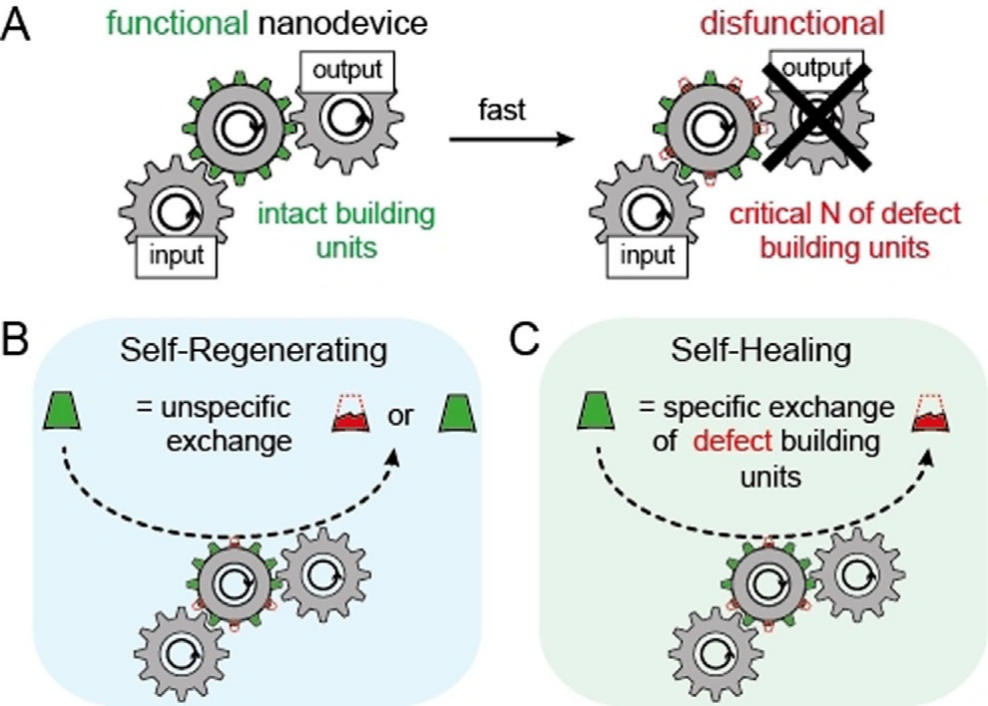
A) Schematic representation of a self‐assembling molecular nanodevice as cogwheel with molecular building units represented by cogs. Intact building units are highlighted in green, defective building units in red. Principle of self‐regenerating (B) and self‐healing (C) nanodevices. Steady‐state exchange of random building units (intact and defective) with intact building units is defined as “self‐regenerating”, while specific exchange of defective building units is defined as “self‐healing”.

Here, we propose to exploit the self‐assembly and reconfiguration abilities of DNA origami technique to introduce general mechanisms for self‐repair within defective or externally damaged nanostructures.[^9^, ^25^, ^26^, ^27^] We classify self‐repair mechanisms in two categories, that is, self‐regenerating and self‐healing systems as explained in the following. Scheme 1 shows a functional molecular nanodevice represented by a force transmission system using cogwheels. Intact molecular building units are illustrated as green cogs while defective building units are shown as broken red cogs. Damage under wear conditions leads to loss of intact building units until a critical number of defective building units is reached so that the functionality of the nanodevice breaks down (Scheme 1 A). We imagine two possibilities to maintain the functional force transmission system. First, the building blocks that are outwearing are constantly exchanged by new building blocks (referred to as self‐regenerating, Scheme 1 B), or alternatively, only the defective pieces are exchanged specifically (referred to as self‐healing, Scheme 1 C). Following this classification, we present self‐repair of DNA origami devices and demonstrate them on selected examples showing how emulated as well as random and unknown enzymatic and light‐induced damages can be reversed. The different self‐repair systems are demonstrated using atomic force microscopy (AFM) and single‐device fluorescence experiments. Among others, we show the ability to recover DNA origami nanorulers used in superresolution microscopy and DNA origamis with defined brightness which can become nanoscale calibration references. This work represents a starting point for developing more comprehensive and sustainable approaches towards functional, self‐repairing molecular devices.

## Results and Discussion

**Figure 1:**
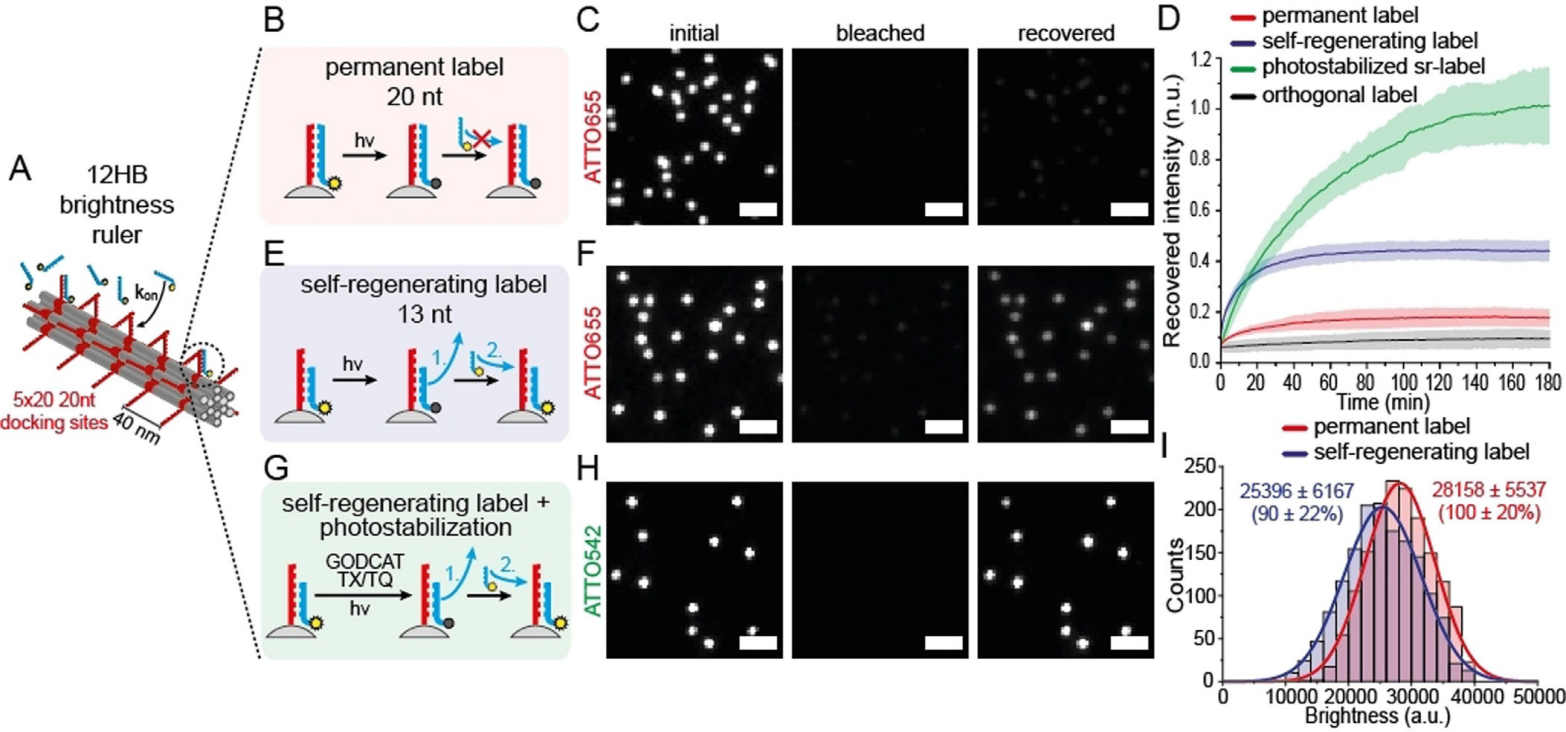
A) Scheme of a 12HB brightness ruler with 5×20 docking sites for external labeling. B) Scheme of conventional permanent external brightness labeling with 20 nt imager strands. C) Exemplary TIRF images of initial, bleached and recovered (180 min) immobilized 12HB brightness rulers with permanent label (20 nt). D) Extracted averaged and normalized DNA origami intensity transients after photobleaching (75 W cm^−2^) for different imager strands. The permanent 20 nt label (ATTO655) is highlighted in red, an orthogonal imager strand reference (ATTO655, 20 nt) is highlighted in grey, the self‐regenerating label (ATTO655, 13 nt) is highlighted in blue, respectively. The photostabilized self‐regenerating label (ATTO542, 13 nt) is highlighted in green (higher bleaching power of 0.5 kW cm^−2^). Data represent average of three experiments, highlighted areas represent the standard deviation. E) Scheme of dynamic and regenerating external labeling with 13 nt imager strands. F) Exemplary TIRF images of initial, bleached and recovered (180 min) immobilized 12HB brightness rulers with self‐regenerating label (13 nt). G) Scheme of dynamic and regenerating external labeling with 13 nt imager strands and photostabilization. H) Exemplary TIRF images of initial, bleached and recovered (180 min) immobilized 12HB brightness rulers with photostabilized self‐regenerating label (13 nt, ATTO542). I) Exemplary brightness histograms for immobilized 12HB brightness rulers with permanent label (red) and self‐regenerating label (blue). Scale bars are 2 μm.

To establish exemplary self‐repair mechanisms within functional nanodevices, we focused on DNA origami nanostructures with a controlled number and position of fluorescent labels acting as nanorulers. Such nanorulers can serve as distance reference structures for emerging super‐resolution microscopy applications as well as brightness reference standards to determine, for example, the sensitivity of a smartphone microscope.[^28^, ^29^, ^30^, ^31^, ^32^] While bright point light sources are highly desired for calibration purposes,^[33]^ nanoscale brightness references suffer from molecular device degradation under wear conditions, for example, by photobleaching of the labels and photoinduced damage to the nanostructure during the measurement so that a brightness reference is only providing reliable data for a short period of time. First, we studied the possibility to maintain the DNA device by regenerating the brightness functionality by refreshing with non‐bleached units. The 12‐helix bundle (12HB) DNA origami shown in Figure 1 A was labeled with fluorescent dyes by hybridizing protruding single‐stranded DNA extensions (called docking strands) with an excess of complementary dye‐labeled strands (called imager strands) in solution (experimental procedures, methods and materials are provided in Supporting Information).^[29]^ For the 12HB shown in Figure 1 A, labeling of 100 docking sites was ensured by saturating the docking sites with a 5 nM solution of 20 nucleotide (nt) long complementary fluorescent imager strands (Figure 1 B). DNA origamis were immobilized via incorporated biotin modified DNA strands on neutravidin‐biotin‐BSA passivated coverslips and imaged via total internal reflection fluorescence (TIRF) microscopy so that only fluorescent dyes at the surface were excited and affected by photobleaching (see image in Figure 1 C). Upon continuous illumination (3 min with 75 W cm^−2^), the DNA origamis photobleach (Figure 1 C, middle). Thus, each area can only be imaged once and pre‐illuminated areas will not contain DNA origamis with the expected brightness. We used time‐lapse imaging avoiding photobleaching (640 nm with 75 W cm^−2^, 100 ms every 60 s) of the same imaging area to see whether the brightness rulers recover in the presence of the 5 nM solution of imager strands but only observed a small recovery of the fluorescence (Figure 1 C, right image). For quantification, we identified the locations of the brightness nanorulers and plotted their average brightness against recovery time (Figure 1 D, red graph). The recovery of up to 15 % is ascribed to post‐labeling of previously inaccessible docking strands^[34]^ as it was shown that not all docking strands of DNA origami are always accessible (see Figure S2 and discussion). An orthogonal imager strand with non‐complementary sequence was used as a control and did not yield any fluorescence recovery (Figure 1 D, gray). To induce self‐regeneration, we rationalized that the binding interaction between the docking and imager strands has to be weakened to allow for strand exchange exploiting the ambient thermal energy. Using 13 nt long imager strands, the labeling is transient with binding times on the order of minutes (Figure 1 E) while the brightness nanorulers are also efficiently labeled (Figure 1 F). Photobleaching still yielded dark areas (Figure 1 F, middle) which recovered over the course of three hours (Figure 1 F right). The intensity of the spots, however, did not recover completely but saturated at 20–60 % of the initial fluorescence intensity (see Figure 1 D, blue). Besides the fluorophore photobleaching, generation of reactive oxygen species (ROS) under constant illumination conditions can also lead to photodamage of the DNA scaffold and the staple strands.^[35]^ Hence, full recovery cannot be reached in line to what has been observed for binding site bleaching in DNA PAINT experiments.[^36^, ^37^] To suppress the damage to the docking sites by ROS and to photostabilize the fluorescent labels, we removed oxygen enzymatically and quenched reactive triplet and radical states by a reducing and oxidizing system (ROXS) (Figure 1 G).[^38^, ^39^] The dye was changed to ATTO542 as ATTO655 shows pronounced blinking when using ROXS.^[40]^ Interestingly, under these conditions, the self‐regenerating label recovered completely to 100 % of its initial brightness (Figure 1 H,and D green) although higher bleaching powers (0.5 kW cm^−2^ at 532 nm) had to be applied to achieve the complete bleaching of the self‐regenerating labels (Figure 1 H and Figure S3). We also investigated the ability to recover the self‐regenerating label over multiple bleaching events with and without photostabilization (Figure S4). While the self‐regenerating label without photostabilization revealed decreasing recovery over every bleaching cycle, the photostabilized self‐regenerating label showed a stable recovery of over 60 % of initial brightness even after 5 bleaching cycles.

The successful self‐regeneration of the brightness rulers with higher bleaching powers indicates that the nucleic acid structure is protected by the photoprotection buffer even more efficiently than the fluorescent dyes. The self‐regeneration of brightness nanorulers might be of immediate importance for the development of nanoscale reference structures. Importantly, self‐regeneration could be achieved without significantly compromising the brightness of the structures (Figure 1 I) as the binding equilibrium is on the side of bound imager strands and the binding/unbinding kinetics might be further optimized by adapting concentrations and the length of imager strands.

In the self‐repair by self‐assembly mechanisms shown here for DNA brightness standards, thermal energy is exploited to drive the dynamic equilibrium reaction. As the labeling units are constantly exchanged, independent of whether they are photobleached or not, we refer to this self‐repair mechanism as self‐regeneration (as defined in Scheme 1 B).

**Figure 2:**
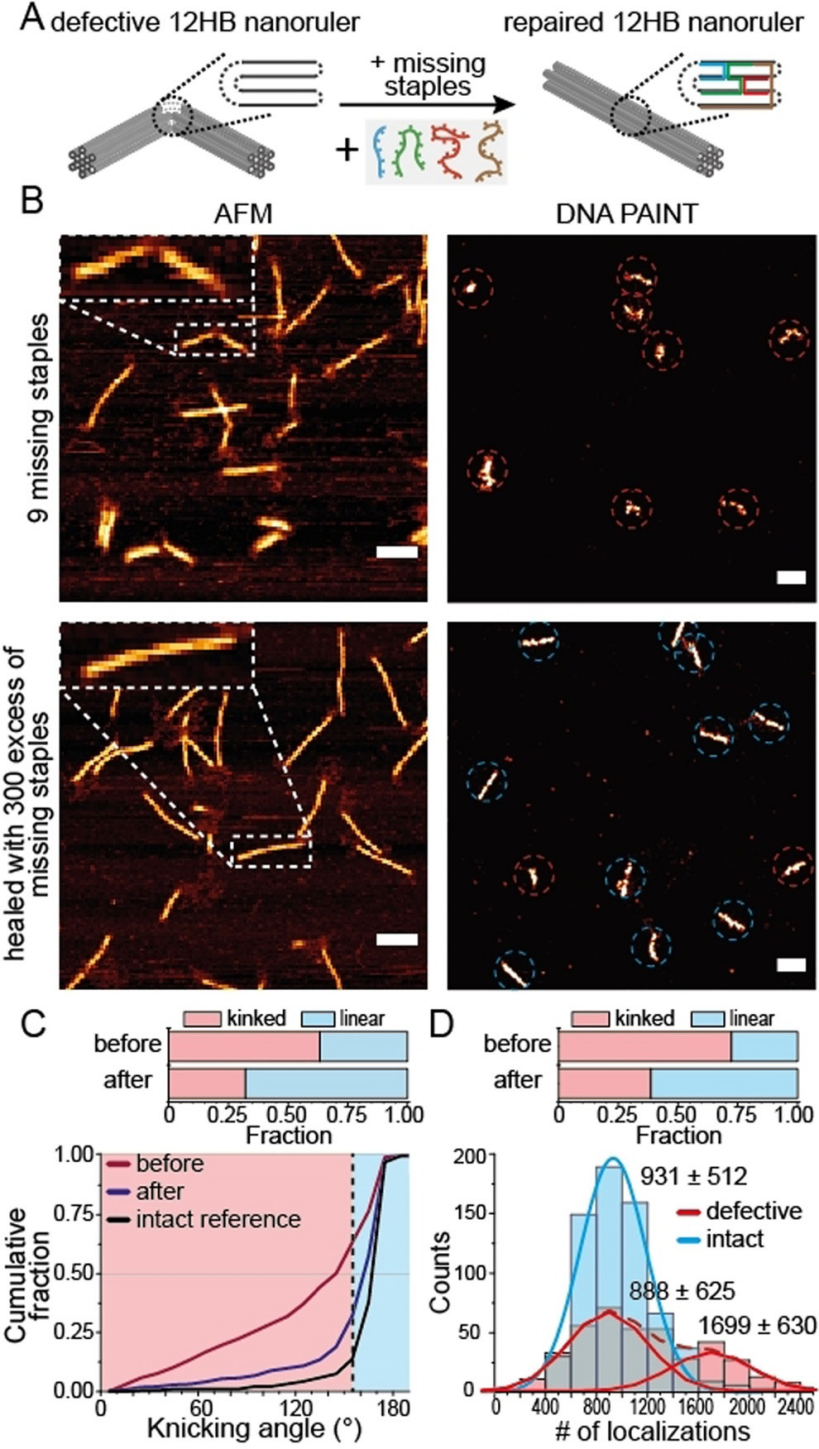
A) Scheme of a defective and kinked 12HB nanoruler missing 9 staple strands in the central region. Incorporation of the missing staples recovers the designed linear structure. B) Exemplary AFM (left) and DNA PAINT (right) images of defective 12HB sample missing 9 staples (top) and repaired 12HB sample after incubation with the set of missing staple strands (bottom). AFM scale bars 100 nm. DNA PAINT scale bars 200 nm. C) Cumulative angular distributions extracted from AFM images for defective (red), repaired (dark blue) and intact reference (black) 12HB nanorulers. Fraction of defective/kinked nanorulers (angle below 160°) decreased from 63 % to 32 % during repair. D) Number of localizations per nanoruler extracted from DNA PAINT images for repaired 12HB sample. Defective fraction (red) were identified as monomer and dimer populations and decreased from 72 % to 38 % during repair.

Next, we studied self‐healing of a structural damage within a DNA origami nanostructure via AFM and DNA PAINT imaging. In our definition of self‐repair processes, self‐healing implies that the repairing reaction only occurs in the presence of a damage (see Scheme 1 C). We synthesized a DNA origami 12‐helix bundle (12HB) and emulated a structural damage by leaving out 9 staple strands in the center of the structure (Figure 2 A). AFM images on mica showed that a large fraction of damaged DNA origamis contained kinks and lower heights in the region of missing staple strands (Figure 2 B). DNA PAINT imaging on BSA‐coated coverslips revealed a large fraction of defective, collapsed nanorulers (Figure 2 B). In order to test whether the missing staple strands can be incorporated into the already existing DNA origami and whether the linear conformation can be restored, we incubated the solution of damaged 12HB DNA origami structures with a 300× excess of the missing staple strands starting at 50 °C (i.e. below the denaturation temperature of the 12HB) and slowly cooling to room temperature (see Table S3). Imaging of immobilized 12HB origami nanostructures by AFM and DNA PAINT illustrates the successful repair of a significant fraction (Figure 2 B). The majority of repaired 12HB exhibited a stretched linear structure and constant height along the whole 200 nm axis of the nanostructure. DNA PAINT imaging confirmed the stretched contour of intact 12HB for the majority of the structures. AFM image quantification (Figure 2 C and Figure S5) showed that the resulting angle distribution of the repaired 12HB nanorulers is similar to the distribution of intact reference 12HB structures, while the damaged sample showed a broad distribution ranging between 0° to 180°. Assigning 12HB nanostructures with an angle over 160° as linear and intact resulted in a decrease of the defective fraction from initially 63 % to only 32 % after incorporation of the missing staple strands. DNA PAINT image quantification (Figure 2 D and Figure S6) by picking defective, collapsed vs. intact, linear nanorulers with the Picasso software^[41]^ exhibited a similar decrease of the defective fraction from initially 72 % to 38 %. To further validate the repair of defective 12HB nanorulers, we extracted the number of DNA PAINT localizations per picked nanoruler for intact and defective fractions within the repaired 12HB sample. The histograms in Figure 2 D reveal similar numbers of localizations for defective (888) and intact (931) nanoruler monomers but also nanoruler dimers (1699) within the set of defective nanorulers. Dimer formation is ascribed to sticking of two defective 12HB nanorulers in the single‐stranded region of the damage. The comparable number of localizations for defective and intact monomers indicates that the repair recovered structural features without influencing the designed docking sites for DNA PAINT. Successful incorporation of at least a sub‐set of the 9 missing staple strands was additionally proven by co‐localized widefield‐DNA PAINT imaging by incorporation of Cy5 labeled staple strands (Figure S7). While previous studies showed the removal of incorporated staple strands from DNA origami nanostructures using staple strand toeholds and complementary external catching strands,[^26^, ^42^] the repair of the kinked 12HB exhibits that staple strands can also be incorporated into existing DNA origami nanostructures and that the structural integrity can be restored. Nevertheless, these experiments do not finally prove a self‐healing mechanism as it is conceivable that also intact staple strands within the DNA origami could be constantly exchanged by staple strands in solution.

To assess whether a damage is required for the exchange between DNA nanostructure and free staples in solution, we designed a rectangular DNA origami (new rectangular origami, NRO) containing two spots with three docking strands per spot for DNA PAINT imaging experiments (Figure S8). We then added staple strands with DNA PAINT docking strand extensions that would form a third spot on the DNA origami for DNA PAINT binding studies when incorporated. Efficient incorporation of the added staples could only be observed when the NRO nanorulers were previously assembled with shorter staple strands so that a toehold of 4 or 8 nucleotides was formed within the scaffold strand (Figure S8 and S9) confirming the notion that a toehold is required for efficient strand displacement reactions also within an intact DNA origami.^[43]^ This observation suggests, that the exchange of a defective staple strand within a DNA origami is kinetically and thermodynamically feasible due to the incomplete hybridization to the scaffold, while intact staple strands are not replaced. Hence we concluded, that a self‐healing mechanism can stabilize DNA origami structures when a toehold is formed as part of the damage. We aimed to find out if this self‐healing strategy could increase the stability of DNA origami nanorulers when the damage is random and unknown. In the experiments with the kinked 12HB and the reconfigurable NRO nanostructures, the damage was artificially inserted. In a more realistic setting, DNA origamis have to function in complex environment with various factors, including degrading enzymes, posing a risk to their stability. To study this, we assessed the stability of DNA nanostructures in a complex medium such as fetal bovine serum (FBS) containing typically a set of various endo‐ and exonucleases.

**Figure 3:**
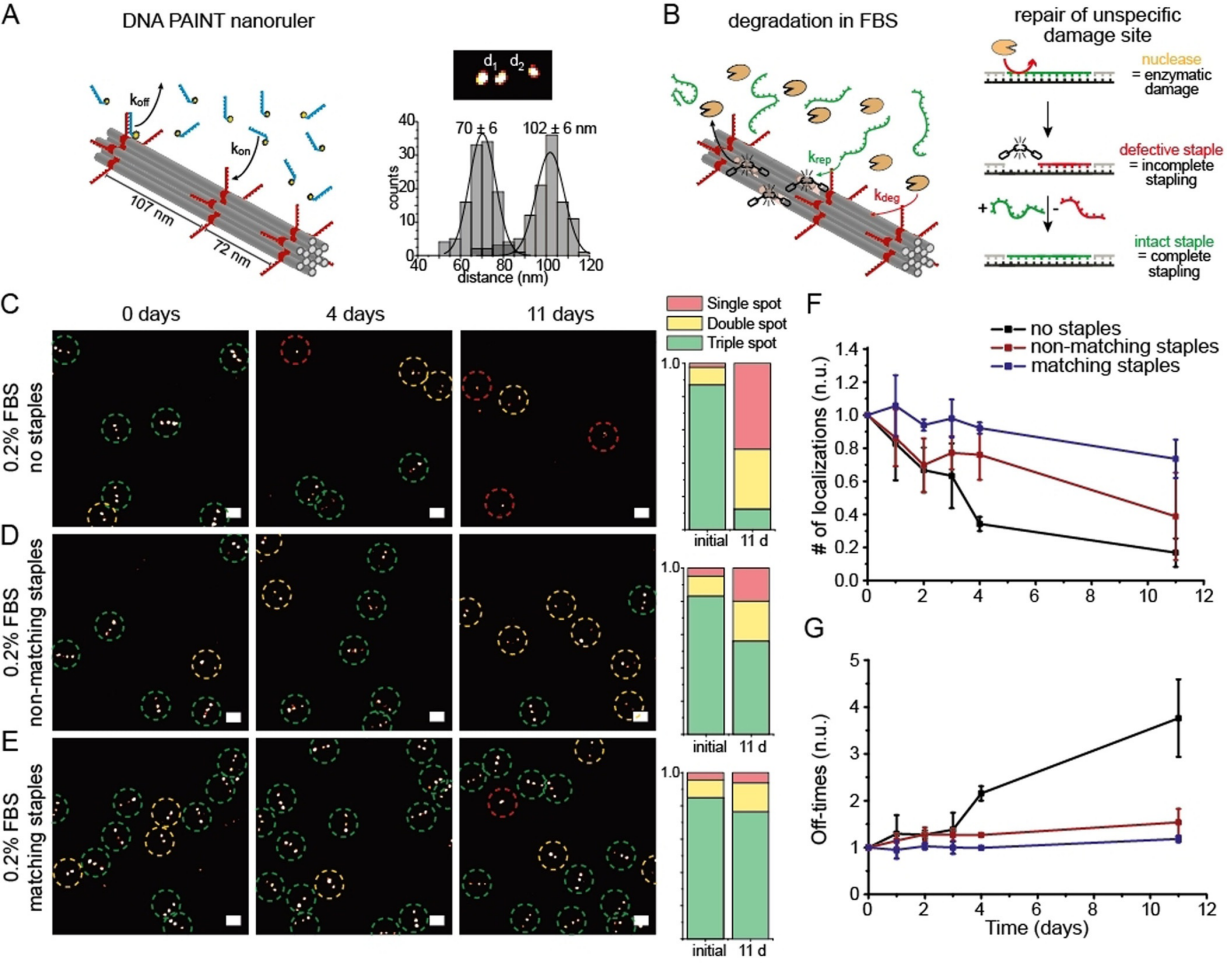
A) Left: Scheme of the 12HB nanoruler containing three DNA PAINT binding spots. Right: Exemplary DNA PAINT image of triple‐spot nanoruler with exemplary distance histogram. B) Scheme of 12HB nanoruler degradation incubated in FBS solution. Damaged staple strands are repaired by intact staple strands in solution via self‐healing. C–E) Exemplary DNA PAINT images of 12HB triple spot nanorulers in 0.2 % FBS solution, with a mix of non‐matching DNA strands and with a mix of matching staple strands over 11 days, respectively. Triple‐spot nanorulers are highlighted by green, double‐spot nanorulers by yellow and single‐spot nanorulers by red circles. Bar plots (right) summarize the extracted fractions of triple, double and single spot nanorulers after immobilization and after 11 days of incubation. Scale bars represent 200 nm. F) Corresponding extracted, averaged and normalized number of localizations per nanoruler. G) Corresponding extracted, averaged and normalized off‐times per nanoruler. Each line represents the average of three different measured samples, error bars represent standard deviation.

Previous work showed the rapid degradation of unmodified DNA nanostructures in 10 % FBS solution within 24 h.[^44^, ^45^, ^46^] Therefore, to monitor the structural stability of DNA nanostructure over time, we designed a 12HB nanoruler equipped with three marks each containing ten docking sites for DNA PAINT (see Figure 3 A for Scheme and superresolution DNA PAINT image with inter‐mark distances of 70 nm and 102 nm). We incubated immobilized 12HB nanorulers with FBS (diluted to 0.2 %) and checked the integrity of the structures over several days. We reasoned that damage to the staple strands yields toeholds in the DNA origami scaffold that could be repaired by intact staple strands in solution via strand displacement reactions (Figure 3 B, right Scheme). We carried out three parallel experiments. First, 12HB nanorulers were incubated with degrading FBS solution only. In the second experiment, we also added a full set of matching staple strands of the 12HB DNA origami at an overall staple concentration of 5 μM (i.e. 22.5 nM per individual staple strand). In a third experiment, we added the same concentration of non‐matching DNA staples, that is, a set of oligonucleotides showing no relevant overlap with the scaffold. In FBS, the 12HB nanorulers were strongly degraded after 11 days and the number of DNA origami structures with three marks in DNA PAINT decreased from 87 % to 12 % of all structures (Figure 3 C, more exemplary data in Figure S10). We observed that the degradation of 12HBs was retarded in the presence of non‐matching DNA strands and that the number of structures still exhibiting 3 marks in the DNA PAINT image of Figure 3 D decreased from 83 % to 56 %. We ascribed this stabilizing effect by the non‐matching DNA to the sacrificial degradation of the added DNA strands slowing down the degradation rate of the immobilized nanorulers. Interestingly, the 12HBs were even further stabilized and protected in the presence of the specific staple strands and 76 % (starting at 85 %) of nanorulers still exhibited 3 fluorescent marks after 11 days of incubation in FBS (Figure 3 E). Besides manual counting of the fluorescent spots in picked nanorulers, the degradation was also visualized by the decreasing number of localizations (Figure 3 F) and increasing off‐time (time between two binding events, Figure 3 G) per nanoruler in the DNA PAINT experiments. Quantitative analysis of the number of localizations and off‐times supported the results from manual counting: the 12HB origami in FBS solution revealed a strong decrease of localizations per nanoruler after 11 days to under 20 %, while the mean off‐time increased almost 4‐fold. The sample incubated in non‐matching DNA strands revealed a medium decrease for the number of localizations and a small increase of the mean off‐time, while the 12HB incubated with the set of specific staples showed stable localization counts and off‐times almost over the whole 11‐day period. We interpret the stabilization of the 12HB nanorulers by the set of matching staple strands as autonomous self‐healing as the displacement of staple strands within the structure is only kinetically and thermodynamically favored for the sites containing a toehold as a result of a previous damage (see discussion in SI and Figure S8 and S9). We also studied the stability of immobilized 12HB DNA PAINT nanorulers in 10 % FBS solution (Figure S11 and S12). With the higher concentrations of nucleases present in the 10 % FBS solution fast structural degradation of the 12 HB was observed. The addition of the DNA staples in solution allowed to preserve the stability of 12 HB nanostructures even over 2 h in 10 % FBS. Here we found a comparable stabilization of nanorulers by the matching and non‐matching DNA staples, suggesting that fast degradation under these conditions cannot be compensated by self‐healing as shown for 0.2 % FBS incubation over days. Self‐healing of DNA origami is thus limited to lower damage rates, while the sacrificial degradation of added DNA can stabilize the nanorulers even at high FBS concentration.

**Figure 4:**
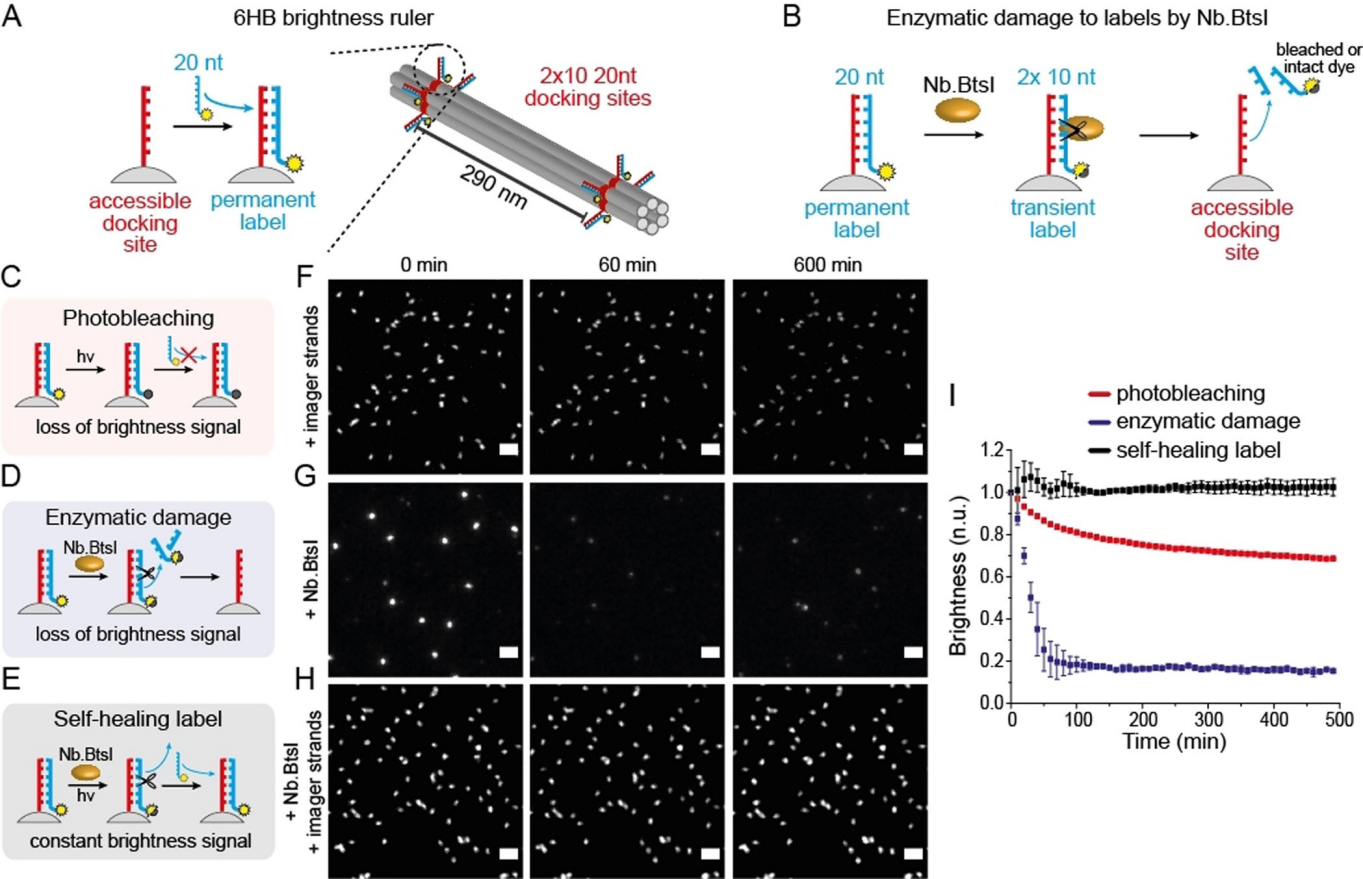
A) Scheme of 6HB brightness ruler with 2×20 nt docking sites for permanent labeling. B) Scheme of label cleavage by restriction enzyme *Nb.BtsI* cutting the double‐stranded 20 nt imager strands into two 10 nt fragments. C) Scheme of conventional permanent external brightness label and slow loss of signal due to photobleaching. D) Scheme of enzymatic damage to brightness label by *Nb.BtsI* and fast loss of brightness signal. E) Scheme of self‐healing label by simultaneous addition of imager strands and *Nb.BtsI*. F–H) Exemplary time‐lapse TIRF images (75 W cm^−2^ for 100 ms every 10 min). Scale bars are 2 μm. F) Photobleaching of permanent label in 5 nM imager solution leads to loss of brightness signal. G) Enzymatic damage induced by *Nb.BtsI* leads to rapid loss of brightness signal. H) Simultaneous addition of *Nb.BtsI* and 5 nM imager strand solution establishes self‐healing label and leads to stable brightness signal over time. I) Extracted normalized and averaged single nanoruler intensity transients for the three labeling conditions. The graphs represent the averages of three different experiments. Error bars represent the standard deviation.

Finally, to show that both self‐healing and self‐regenerations mechanisms can be combined within one DNA nanostructure, we designed robust brightness labels consisting of a 6HB DNA origami with two spots each containing ten binding sites at a contour length distance of 290 nm (Figure 4 A). The binding sites were labeled with a 20 nt long imager strand carrying the fluorescent dye ATTO655. Our analysis of fluorescence intensity of the immobilized nanorulers showed that external labeling occurred with 60 % labeling efficiency (Figure S13A). We then added the nicking enzyme *Nb.BtsI* to the labeled nanorulers that specifically hydrolyses the imager strand exactly in the middle when it is hybridized to the docking site so that single‐stranded imager strands in solution stay intact. The resulting two 10 nt fragments are not stably bound to the DNA origami and dissociate rapidly leaving a brightness ruler with strongly reduced fluorescence signal (Figure 4 B). After washing the nicking enzyme away and adding intact imager strands, the labels recovered back to almost 100 % of the initial labeling brightness (Figure S13B and C). Next, we compared three different labeling conditions to visualize the concepts of a self‐healing and self‐regenerating label within one system (see Figure 4 C–E). Figure 4 shows exemplary time‐lapse TIRF images of immobilized 6HB brightness rulers incubated with a 5 nM solution of imager strands (F), with a solution of *Nb.BtsI* (G) and with a solution of 5 nM imager strands and *Nb.BtsI* (H) (additional TIRF images given in Figure S14). Extracted averaged intensity transients over hundreds of nanorulers under time‐lapse imaging (640 nm at 75 W cm^−2^, 100 ms every 10 minutes) are given in Figure 4 I. Photobleaching led to slow degradation to 70 % after 8 h (Figure 4 F and Figure 4 I, red graph). Addition of *Nb.BtsI* to externally labeled 6HB brightness ruler led to accelerated loss of brightness due to enzymatic cleavage. After about 2 h a plateau below 20 % of the initial fluorescence intensity was reached. When the *Nb.Btsl* and a 5 nM imager strand solution were added simultaneously, no degradation was visible over 1200 min of time‐lapse imaging showing that self‐repair mechanisms can quantitatively compensate mechanisms of wear out (Figure 4 and Figure S14). Here, the repair of brightness function of the DNA origami nanorulers can be considered as self‐healing with respect to the enzymatic damage to the attached imager strands, as only those strands exchange that were cleaved by the enzyme. With respect to the fluorescent dye, the photobleaching damage is repaired in a self‐regenerating mechanism as dyes are exchanged independent of whether they are photodamaged or not. Hence, the self‐repair in this example shows that self‐regeneration and self‐healing can occur simultaneously within one system when different sources of damage are present.

Differently from enzymatic repair approaches where the prior knowledge of the damage site is required to evoke the repair (e.g. DNA ligases require sequence specificity while DNA polymerase also require specific primers), active self‐healing and self‐regeneration mechanisms outlined here provide general strategies to address random and unknown damage. This is best illustrated by drastic improvement in the stability of DNA nanostructures in a complex and chemically demanding FBS environment shown in Figure 3. On the other hand, it is important to mention that the approach of exchanging the damaged building blocks with intact ones in solution also has its limitations. With respect to DNA origami nanostructures, only staple strands can be repaired via this approach, while damages to the long DNA scaffold strand cannot be addressed—under high stress conditions cumulative and prolonged damages to the scaffold strand may indeed provide the breakdown of the function of the DNA nanostructure. Furthermore, elevated temperatures used to restore the structural stability of kinked 12 HB DNA nanostructure, as shown in Figure 2, might also not be suitable for all applications. Nevertheless, we think that the self‐repair strategies introduced in this work provide a complementary tool to the existing enzymatic[^47^, ^48^, ^49^] and chemical approaches[^19^, ^20^, ^21^, ^22^] to stabilize and manipulate DNA nanostructures and can be combined together to obtained even more robust and “smart” designs on the nanoscale.

## Conclusion

In the development of materials and molecular machines with increasing complexity, their robustness and their resistance against wear as well as their ability to prevail in complex environments call for new approaches of protection. In this context, self‐repair mechanisms that are common in nature also become more important to be implemented in artificial systems. As DNA nanotechnology enables self‐assembling nanostructures and molecular functional devices of highest complexity, we exploit self‐assembling and reconfiguration properties to implement self‐repair mechanisms. These include self‐regeneration by a pool of intact building blocks and exchange under conditions of thermal equilibration as well as more specific self‐healing that only allows exchange of building blocks upon occurrence of a damage. We showed that such mechanisms can already be implemented in existing applications of DNA origami nanorulers and brightness references. Self‐repair strategies might become a crucial area of research when pursuing our visions of sustainable, long‐lasting molecular nanorobots.

## Conflict of interest

The authors declare no conflict of interest.

## Supporting information

As a service to our authors and readers, this journal provides supporting information supplied by the authors. Such materials are peer reviewed and may be re‐organized for online delivery, but are not copy‐edited or typeset. Technical support issues arising from supporting information (other than missing files) should be addressed to the authors.

Supplementary
