# Supplementary material for: Self‐Regeneration and Self‐Healing in DNA Origami Nanostructures
Source: Angew Chem Int Ed Engl. 2021 Jan 28;60(9):4931–8. doi: 10.1002/anie.202012986 (PMC7986372; doi:10.1002/anie.202012986)
Supplement: Supplementary file 1 — Supplementary [file ANIE-60-4931-s001.pdf]

Angewandte Chemie
Eine Zeitschrift der Gesellschaft Deutscher Chemiker

Supporting Information

# Self-Regeneration and Self-Healing in DNA Origami Nanostructures

*Michael Scheckenbach, Tom Schubert, Carsten Forthmann, Viktorija Glembockyte,* and Philip Tinnefeld**

anie_202012986_sm_miscellaneous_information.pdf

## Table of Contents

Table of Contents ..... 2
1. Experimental Procedures ..... 3
1.1. General materials ..... 3
1.2. DNA Origami folding ..... 3
1.3. Purification of DNA origami nanostructures ..... 4
1.4. Surface-Immobilization of DNA origami nanorulers ..... 4
1.5. Photostabilization of fluorescent labels ..... 5
1.6. Fluorescence imaging of DNA origami nanorulers used as brightness standards ..... 5
1.7. DNA PAINT imaging ..... 5
1.8. Analysis of DNA PAINT data ..... 6
1.9. AFM imaging with JPK Nanowizard ..... 6
1.10. Self-Regenerating brightness label on 12HB brightness ruler ..... 6
1.11. Repair of kinked 12HB nanoruler ..... 9
1.12. Staple strand exchange from dual spot to triple spot in NRO nanoruler ..... 11
1.13. Self-Healing of 12HB nanoruler in degrading conditions ..... 12
1.14. Self-Regeneration and Self-Healing of an enzymatically cleavable brightness label on 6HB brightness nanoruler ..... 13
2. Results and Discussion ..... 15
2.1. Self-Regenerating brightness label on 12HB brightness nanoruler ..... 15
2.2. Repair of kinked 12HB nanoruler ..... 18
2.3. Staple strand exchange from dual spot to triple spot in NRO nanoruler ..... 21
2.4. Self-Healing of 12HB nanorulers in degrading conditions ..... 23
2.5. Self-regeneration and relf-healing of an enzymatically cleavable label on 6HB brightness ruler ..... 26
3. Appendix ..... 28
References ..... 41
Author Contributions ..... 41

# 1. Experimental Procedures

## 1.1. General materials

For folding, purification and storage of DNA origami nanostructures, a 1x TAE buffer consisting of 20 mM acetic acid, 1 mM EDTA, and 50 mM Tris was used. The 1x PBS buffer used for fluorescence imaging consisted of 2.7 mM KCl, 137 mM NaCl, 1.8 mM $KH_2PO_4$, and 10 mM $Na_2HPO_4$. Individual concentrations of $Mg^{2+}$ used for every DNA origami nanostructure are given in Table S1.
The scaffold strands were extracted from M13mp18 bacteriophages. Unmodified staple strands were purchased from Eurofins Genomics GmbH and Integrated Device Technology Inc. Dye labeled oligonucleotides for DNA PAINT imaging or brightness labeling were purchased from Eurofins Genomics GmbH (Germany).

The restriction enzyme *Nb.BtsI* together with the 1X CutSmart® buffer (20 mM Tris-acetate, 10 mM magnesium acetate, 50 mM potassium acetate, 100 µg/ml BSA) were purchased from New England Biolabs (NEB, USA).

Specific materials used for individual experiments are described in the sections below.

## 1.2. DNA Origami folding

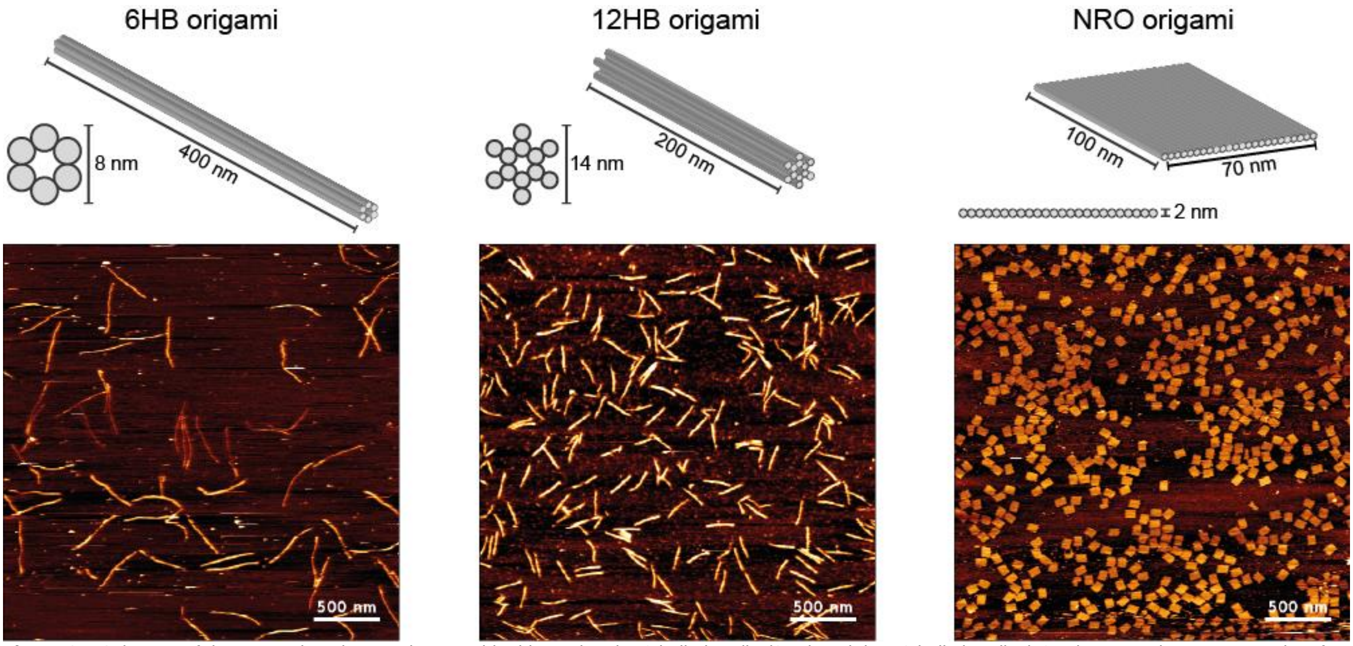


**Figure S1.** Schemes of the DNA origami nanorulers used in this study. The 6 helix bundle (6HB) and the 12 helix bundle (12HB) are used as 1D nanorulers for modern fluorescence microscopy. The new rectangular origami (NRO) can be used as a 2D breadboard for the positioning of various modifications. Corresponding exemplary AFM images obtained after folding and purification shown in the lower panel illustrate the successful self-assembly of predicted structures.

All investigated DNA origami nanostructures (depicted in Figure S1) were synthesized using the corresponding scaffold strands and temperature programs given in Table S1. Modifications of the nanorulers were realized using caDNAno (version 2.2.0). A full list of the unmodified staple strands and sequences of the 12HB DNA origami[1] is given in Table S15. A full list of the unmodified staple strands and sequences of the NRO DNA origami[2] is given in Table S16. A full list of the unmodified staple strands and sequences of the 6HB DNA origami[3] is given in Table S17.

**Table S1** Corresponding scaffold strands, folding programs, folding buffers and magnesium chloride concentrations used for the folding of the DNA origami nanorulers 12HB, 6HB, and NRO shown in Figure S1.

| DNA origami nanoruler | Scaffold | Folding program | Folding buffer | $MgCl_2$ concentration |
|---|---|---|---|---|
| 12HB | P8064 | 1 | 1xTAE | 16 mM |
| 6HB | P7560 | 1 | 1xTE | 14 mM |
| NRO | P7249 | 2 | 1xTE | 12.5 mM |

For the folding of the DNA origami nanostructures, the scaffold strand and the staple strands were mixed as given in Table S2 in the corresponding 1x folding buffer containing $MgCl_2$ concentration as listed in Table S1. Unmodified core staple strands, which are completely incorporated in the origami structure, were used in 10-fold excess with respect to the scaffold strand. Staple strands with protruding 5'-ends, which act as docking sites for DNA PAINT or labeling experiments, were used in 30-fold excess with respect to the scaffold strand. Biotinylated staple strands, which were incorporated for enabling surface immobilization of the DNA origami structures, were used in a 30-fold excess with respect to the scaffold strand.

**Table S2** Final concentrations and relative equivalents of scaffold strand, unmodified staple strands (core staple strands) and modified staple strands (e.g. biotinylated staple strands for immobilization and DNA PAINT docking site staple strands for superresolution imaging) used within this study.

| Reagent | Final concentration [nM] | Equivalents |
|---|---|---|
| Scaffold strand | 13 | 1 |
| Core staple strands | 130 | 10 |
| PAINT docking strands | 390 | 30 |
| Biotinylated staple strands | 390 | 30 |

Folding of the 12HB and 6HB origami was realized with a non-linear thermal annealing ramp over 16 hours (Table S3)[4], while the NRO was folded during a linear annealing ramp over 75 min (Table S4).

**Table S3** Folding Program 1 used for the folding of the1D nanorulers 12HB and 6HB.

| Temperature (°C) | Time per °C (min) | Temperature (°C) | Time per °C (min) |
|---|---|---|---|
| 65 | 2 | 44 | 75 |
| 64 – 61 | 3 | 43 | 60 |
| 60 – 59 | 15 | 42 | 45 |
| 58 | 30 | 41-39 | 30 |
| 57 | 45 | 38-37 | 15 |
| 56 | 60 | 36-30 | 8 |
| 55 | 75 | 29-25 | 2 |
| 54-45 | 90 | 4 | storage |

Folding mixes had a total volume of 100 µl with final concentrations of scaffold strand, core staple strands and modified staple strands (biotinylated, DNA PAINT docking sites) as given in Table S4.

**Table S4** Folding Program 2 used for the folding of the 2D NRO nanorulers.

| Temperature (°C) | Time per °C (min) |
|---|---|
| 90 | 15 |
| 80 – 20 | 1 |
| 4 | storage |

### 1.3. Purification of DNA origami nanostructures

Purification of folded DNA origami nanostructures was realized either by gel electrophoresis or by filter purification.

For gel electrophoresis, a 1 w% aqueous solution of agarose in a 1x TAE buffer with 12 mM $MgCl_2$ was homogenized in a microwave. The solution was cooled down to ca. 50°C and stained with peqGreen (VWR International GmbH, Germany) and solidified by creating the wells for sample loading. The solidified gel was placed within a gel electrophoresis chamber filled with 1x TAE buffer containing 12 mM $MgCl_2$. The whole chamber was placed in an ice bed to prevent melting of the gel. Five parts of sample solution were mixed with one part of 6x BlueJuice loading dye (Thermo Fisher Scientific, USA) and loaded in the wells. The gel was run at 60 V for *ca.* 2 h. Bands of interest were identified using blue light and cut out with a scalpel. Purified DNA solution was extracted by squeezing the cut gel bands. Alternatively, sample purification was realized by filtration using Amicon Ultra filters (100 K, Merck, Germany). The filter was first centrifuged with folding buffer for 7 minutes at 6000 g. The sample solution was then loaded into the filter and centrifuged for 15 minutes at 6000 g. 500 µL of folding buffer was loaded into the filter and centrifuged for 15 minutes at 6000 g, which was repeated. After three washing steps, the filter was inverted and placed into a new collection tube. The purified sample could then be collected by centrifugation for 2 minutes at 1000 g.

Concentrations of purified sample solution were measured via UV/vis spectroscopy (NanoDrop, Fischer Scientific, USA).

### 1.4. Surface-Immobilization of DNA origami nanorulers

For optical microscopy experiments, the DNA origami sample was immobilized on Nunc™ LabTek™ II chambers (Thermo Fisher, USA). The chambers were first cleaned with 400 µL of 1 M KOH solution and washed three times with 1x PBS buffer. Then the surfaces were passivated with 100 µL BSA-biotin (0.5 mg/mL in PBS, Sigma Aldrich, USA) for 30 minutes and washed three times with 1x PBS buffer. The passivated surfaces were incubated with 100 µL neutravidin (0.25 mg mL-1 in PBS, Sigma Aldrich, USA) for 15 minutes and washed three times with 1x PBS buffer. The sample solution with DNA origami featuring several staple strands with biotin modifications on the base was diluted to approximately 50 pM in 1x PBS buffer containing 12.5 mM $MgCl_2$ and incubated in the chambers for 5 to 15 minutes. Sufficient surface density was probed with a TIRF microscope.

### 1.5. Photostabilization of fluorescent labels

Optical measurements with ATTO542 or Cy5 as imager fluorophores were carried out under photostabilizing conditions.[5] A 2.5x TAE buffer with 1 % (wt/v) D-(+)-glucose (Sigma Aldrich, USA), 165 units/mL glucose oxidase (G2133, Sigma Aldrich, USA), 2170 units/mL catalase (C3155, Sigma Aldrich, USA), 1 mM Trolox and 2 M NaCl was used.[6] The sample chamber with surface immobilized origami sample was completely filled with imaging buffer and sealed to prevent oxygen solvation. The first measurements were carried out at least 20 minutes after introducing the oxygen removal system to allow the equilibration of the oxygen concentration in the sample solution.

### 1.6. Fluorescence imaging of DNA origami nanorulers used as brightness standards

Automated long-term experiments with brightness nanorulers (12HB with self-regenerating label and 6HB with *Nb.BtsI* cleaved labels) were carried out on a commercial Nanoimager S (ONI Ltd., UK). Red excitation at 638 nm was realized with a 1100 mW laser, green excitation at 532 nm with a 1000 mW laser, respectively. The microscope was set to TIRF illumination. In order to not corrupt the first frames of the acquired intensity transients by the photobleaching of single nanorulers, the objective was first focused into the sample plane on a random section of the glass surface and the auto focus was activated. Subsequently the imaging lasers were shut off. Before starting time lapse measurements, the sample slide was moved to a new region of interest while still being kept in focus by the auto focus. The data acquisition was initialized by activating the lasers and taking frames of 100 ms over a user defined acquisition protocol (*e.g.* a frame of 100 ms taken every 10 min).

Throughout this study, fluorescence brightness imaging was realized with different imager strands but same imager concentration of 5 nM in 1x PBS buffer containing 12.5 mM $MgCl_2$. Brightness data processing including background subtraction and data analysis were performed with ImageJ 1.52n (version 1.8.0_172). For drift correction the linear stack alignment with SIFT plugin in ImageJ was used. Spot detection was realized using a custom written algorithm in ImageJ.

### 1.7. DNA PAINT imaging

Super-resolution measurements using the DNA PAINT technique were carried out on a custom-built total internal reflection fluorescence (TIRF) microscope, based on an inverted microscope (IX71, Olympus). Red excitation at 644 nm was realized with a 150 mW laser (iBeam smart, Toptica Photonics) spectrally filtered with a clean-up filter (Brightline HC 650/13, Semrock). For yellow excitation, an additional 560 nm/1 W fiber laser (MPB Communications) also filtered with a clean-up filter (Brightline HC 561/4, Semrock) was used. The red and the yellow beams are combined with a dichroic mirror (T612lpxr, Chroma). To expand the beam profile, the laser passed through lenses (Bi-convex f50, Thorlabs; AC f120, Linos). The laser beam was coupled into the microscope with a triple-color beam splitter (Chroma z476-488/568/647, AHF Analysentechnik) and focused on the backfocal plane of an oil-immersion objective (100 ×, NA = 1.4, UPlanSApo, Olympus) aligned for TIRF illumination. To avoid drift the objective was mounted on a nosepiece (IX-2NPS, Olympus). The fluorescence light is guided through an additional 1.6× optical magnification lens, an emission filter (ET 700/75, Chroma for red excitation or ET 605/70m, Chroma for yellow excitation) and finally focused on an scientific Complementary metal-oxide-semiconductor (sCMOS) camera (pco.panda 4.2, 2048x2048 px, PCO AG) for detection. The calibrated pixel size was 42 nm/pixel. For data acquisition, a pixel binning of 2 was used resulting in an acquisition pixel size of 84 nm. Data acquisition was controlled with the software Micro-Manager 1.4.[7-8]

The two used DNA PAINT imager strands (8 nt in length) with their corresponding sequences and fluorescent labels on the 3´end are given in Table S5. In general, DNA PAINT imaging was realized with the ATTO655 imager strand and imager concentrations of 1 to 5 nM in 1x PBS buffer containing 12.5 mM $MgCl_2$. Typically, a frame time of 50 ms over an experiment time of 20 min was used with 640 nm laser excitation at 30 mW. For two color incorporation studies on the repair of kinked 12 HB nanorulers (Figure S7), DNA PAINT images were obtained with Cy3B imager strands and excitation with the 560 nm laser.

Stabilization studies of 12HB DNA PAINT nanorulers after 2 hours incubation in 10% fetal bovine serum (FBS) solution were carried out on a commercial Nanoimager S (ONI Ltd., UK) using a 10 nM solution of the 6 nt ATTO655 imager strand (Table S5) in an 1x PBS buffer containing 12.5 mM $MgCl_2$. A frame time of 25 ms over an experiment time of 10 min was used with excitation at 638 nm set to 160 mW output power.

**Table S5** DNA PAINT imager strands and fluorescent labels used for DNA PAINT measurements. DNA PAINT imaging in red channel was realized with ATTO655 labelled imager strands, while imaging in green channel with Cy3B labelled imager strands, respectively.

| Sequence (5` to 3`) | Fluorophore label on 3´ |
|---|---|
| CGGGCATT | ATTO655 |
| CGGGCATT | Cy3B |
| CGGGCA | ATTO655 |

### 1.8. Analysis of DNA PAINT data

Acquired DNA PAINT raw data were analyzed using the Picasso software package.[9]

The obtained tiff-movies were first analyzed with the “localize” software from Picasso. Centroid position information of single imager strand binding events was localized with a minimal net gradient of 10000 and a box size of 9 for data acquired with the custom-built TIRF setup and with a minimal net gradient of 2500 and a box size of 5 for data obtained on the Nanoimager S, respectively. The fitted localizations were further analyzed with the “render” software from Picasso. X-y-drift correction of the localizations was corrected with the RCC drift correction. DNA origami nanorulers were picked with the Render software and corresponding mean off-times and number of localizations per picked nanorulers were extracted for further analysis.

For quantitative distance analysis, the localization events of the picked nanorulers were exported from Render as csv. files for further examination with the software GATTAnalysis from GATTAquant GmbH, Germany.

### 1.9. AFM imaging with JPK Nanowizard

For probing correct folding of the origami structures and observing structural properties, AFM images were taken. AFM scans in aqueous solution (AFM buffer = 40 mM Tris, 2 mM EDTA, 12.5 mM $Mg(OAc)_2 \cdot 4\ H_2O$) were realized on a NanoWizard® 3 ultra AFM (JPK Instruments AG). For sample immobilization, a freshly cleaved mica surface (Quality V1, Plano GmbH) was incubated with 10 mM solution of $NiCl_2$ for 3 minutes or alternatively with 0.01 % (wt/v) Poly-L-ornithine solution. The mica was washed three times with ultra-pure water to get rid of unbound $Ni^{2+}$ ions or Poly-L-ornithine and blow-dried with air. The dried mica surface was incubated with 1 nM sample solution for 3 minutes and washed with AFM buffer three times. Measurements were performed in AC mode on a scan area of 3 x 3 µm with a micro cantilever ($v_{res}$ = 110 kHZ, $k_{spring}$ = 9 N/m, Olympus Corp.).

### 1.10. Self-Regenerating brightness label on 12HB brightness ruler

For establishing a self-regenerating brightness label on a 12HB nanoruler, we designed 5x20 docking sites for external labeling into the 12HB structure (Figure 1 main text). The five labeling spots were equally distributed along the 200 nm axis of the 12HB with 40 nm inter-spot distances. Therefore, we exchanged 5x20 unmodified core staple strands of the 12HB in caDNAno with the docking site staple strands given in Table S6. The docking site staple strands exhibit an over 20 nt long overhang at the 3‘-ends. The used imager strands, which are complementary to the sequence of the docking sites, are listed in Table S7. The hybridization of 20 nt imager strands creates at room temperature thermodynamically stable and permanent labels, which are prone to photobleaching. Using shorter imager strands of only 13 nt length leads to a transient dynamic label, which can recover after photobleaching events. All used imager strands were labelled on the 3’-end, including the oxazine dye ATTO655 and the rhodamine dye ATTO542.

Self-regenerating of brightness labels on the 12HB brightness ruler (Figure 1 main text) was investigated with respect to the ability to recover brightness labels after photodamage, i.e. photobleaching. After initial brightness measurements (100 ms with 3 mW excitation at 532 or 640 nm), the immobilized nanorulers were photobleached (3 min, 3 mW without photostabilization, 20 mW with photostabilization) to a complete breakdown of the brightness function. The brightness recovery of the bleached nanorulers was measured over time via time-lapse imaging (100 ms every min with 3 mW at 532 or 640 nm).

**Table S6** Modified staple strands of the 12HB brightness ruler. Sequences are denoted from 5’- to 3’-end. The docking site staple strands exhibit an over 20nt long docking site, marked in red, on the 3’-end. For immobilization, the biotinylated staple strands are modified with biotin on the 3’-end. The numbers for the 5’-end 3’-end of the staples represent the helix number in the corresponding caDNAno file. Number in brackets represent the starting and ending position of the staple in the corresponding helix.

| Docking site staple strands | 5'-end | 3'-end |
|---|---|---|
| CGAGTAACAACCGTTTACCAGTCATATTTCCTCTACCACCTACATCAC | 0[118] | 0[96] |
| GCCTTACGCTGCGCGTAAAATTATTTTTTGACGCTCAATCATATTTCCTCTACCACCTACATCAC | 0[179] | 11[188] |
| CGAGCACAGACTTCAAATACCTCAAAAGCTGCAATATATTTCCTCTACCACCTACATCAC | 0[221] | 11[230] |
| AAAAATCTACGTGCGTTTTAATTATATATTTCCTCTACCACCTACATCAC | 0[244] | 0[222] |

| | | |
|---|---|---|
| AAAACGAAAGAGGCTCATTATACATATATTTCCTCTACCACCTACATCAC | 0[286] | 0[264] |
| AGCGTATCATTCCACAGACCCGCCACAGTTGCAGCAAGCGATATTTCCTCTACCACCTACATCAC | 0[347] | 11[363] |
| GCGTCATACATGCCCTCATAGTTATATTTCCTCTACCACCTACATCAC | 0[370] | 0[348] |
| TCACCGTCACCGGCGCAGTCTCTATATTTCCTCTACCACCTACATCAC | 0[412] | 0[390] |
| AAAAAAGGCAGCCTTTACAATCTTACCAGTTTGATATTTCCTCTACCACCTACATCAC | 0[473] | 11[482] |
| TCCCATCCTAATGAGAATAACATATATTTCCTCTACCACCTACATCAC | 0[496] | 0[474] |
| CTGAAAACCTGTTTATCAAACATGTAACGTCAAATATTTCCTCTACCACCTACATCAC | 0[515] | 11[524] |
| CGGTAGTACTCAATCCGCTGCTGGTCATGGTCATATATTTCCTCTACCACCTACATCAC | 0[53] | 11[62] |
| TTAGGTTGGGTTATAGATAAGTCATATTTCCTCTACCACCTACATCAC | 0[538] | 0[516] |
| TTCTGGAATAATCCTGATTTTGCCCGGCCGTAAATATTTCCTCTACCACCTACATCAC | 0[599] | 11[608] |
| AAGATAAAACAGTTGGATTATACATATTTCCTCTACCACCTACATCAC | 0[622] | 0[600] |
| CCGAACCCCCTAAAACATCGACCAGTTTAGAGCATATTTCCTCTACCACCTACATCAC | 0[641] | 11[650] |
| GATTTTAGACAGGCATTAAAAATAATATTTCCTCTACCACCTACATCAC | 0[664] | 0[642] |
| CCGGAAGACGTACAGCGCCGCGATTACAATTCCATATTTCCTCTACCACCTACATCAC | 0[95] | 11[104] |
| AGCAGTCGGGAAACCTGTCATATATTTCCTCTACCACCTACATCAC | 10[205] | 11[218] |
| AATAACGCGCGGGGAGAGGATATATTTCCTCTACCACCTACATCAC | 10[247] | 11[260] |
| TCGTTCACCGCCTGGCCCTATATTTCCTCTACCACCTACATCAC | 10[331] | 11[344] |
| TACCTGGTTTGCCCCAGCAATATTTCCTCTACCACCTACATCAC | 10[373] | 11[386] |
| AAAAGATAGGGTTGAGTGTATATTTCCTCTACCACCTACATCAC | 10[457] | 11[470] |
| CTATATTAAAGAACGTGGAATATTTCCTCTACCACCTACATCAC | 10[499] | 11[512] |
| TGGCAAGTTTTTTGGGGTCATATTTCCTCTACCACCTACATCAC | 10[583] | 11[596] |
| AACACCCTAAAGGGAGCCCATATTTCCTCTACCACCTACATCAC | 10[625] | 11[638] |
| GTATGTGAAATTGTTATCCATATATTTCCTCTACCACCTACATCAC | 10[79] | 11[92] |
| TAAAGGATTGTATAAGCGCACAAACGACATTAAATGTGAGATATTTCCTCTACCACCTACATCAC | 11[135] | 0[119] |
| GTGCCTGCTTTAAACAGGGAGAGAGTTTCAAAGCGAACCAATATATTTCCTCTACCACCTACATCAC | 11[219] | 0[203] |
| CGGTTAACAAAGCTGCTGTAACAACAAGGACGTTGGGAAGATATATTTCCTCTACCACCTACATCAC | 11[261] | 0[245] |
| GGCGACACCACCCTCAGGTTGTACTGTACCGTTCCAGTAAATATTTCCTCTACCACCTACATCAC | 11[387] | 0[371] |
| CTCCAATTTAGGCAGAGACAATCAATCAAGAAAAATAATAATATTTCCTCTACCACCTACATCAC | 11[513] | 0[497] |
| CCGATAATAAAAGGGACTTAACACCGCGAACCACCAGCAGATATTTCCTCTACCACCTACATCAC | 11[639] | 0[623] |
| GCTCAAGTTGGGTAACGGGCGGAAAAATTTGTGAGAGATAATATATTTCCTCTACCACCTACATCAC | 11[93] | 0[77] |
| CCGAACTTTAATAAAAGCAAAGCGGATTATATATTTCCTCTACCACCTACATCAC | 2[223] | 3[223] |
| TGTAGGGGATTTAGTAACACTGAGTTTCATATTTCCTCTACCACCTACATCAC | 2[349] | 3[349] |
| ATTAAAATAAGTGCGACGATTGGCCTTGATATTTCCTCTACCACCTACATCAC | 2[391] | 3[391] |
| AAATAGGTAATTTACAAATAAGAAACGAATATTTCCTCTACCACCTACATCAC | 2[475] | 3[475] |
| ACGCGTCGGCTGTAAGACGACGACAATAATATTTCCTCTACCACCTACATCAC | 2[517] | 3[517] |
| TCAATAATAAAGTGTATCATCATATTCCATATTTCCTCTACCACCTACATCAC | 2[601] | 3[601] |
| GATAGTGCAACATGATATTTTTGAATGGATATTTCCTCTACCACCTACATCAC | 2[643] | 3[643] |
| GCCTTATACCCTGTAATACCAATTCTTGCGCTCATATATTTCCTCTACCACCTACATCAC | 2[97] | 3[97] |
| GCTAAATCGGTTTGACTATTATAATATATTTCCTCTACCACCTACATCAC | 3[182] | 3[204] |
| GCATCAAAAAGAAGTAAATTGGGATATATTTCCTCTACCACCTACATCAC | 3[224] | 3[246] |
| CTAAAGACTTTTAGGAACCCATGATATTTCCTCTACCACCTACATCAC | 3[308] | 3[330] |
| GTCACCAGTACAAGGTTGAGGCAATATTTCCTCTACCACCTACATCAC | 3[350] | 3[372] |
| TTTTTTGTTTAATAAAGTAATTCATATTTCCTCTACCACCTACATCAC | 3[476] | 3[498] |
| ATCAGCGGGGTCAGCTTTCAGAGATATATTTCCTCTACCACCTACATCAC | 3[56] | 3[78] |
| TGATTATCAGATATACGTGGCACATATTTCCTCTACCACCTACATCAC | 3[602] | 3[624] |
| GGCGCCCCGCCGAATCCTGAGAAGTGAGGCCGATTAAAGGATATATTTCCTCTACCACCTACATCAC | 3[667] | 0[665] |
| GGATAACCTCACAATTTTTGTTAATATTTCCTCTACCACCTACATCAC | 3[98] | 3[120] |
| CCAGCCAAACTTCTGATTGCCGTTTTGGGTAAAGTTAAACATATATTTCCTCTACCACCTACATCAC | 4[102] | 7[104] |
| GTTTGAGGGGACCTCATTTGCCGATATTTCCTCTACCACCTACATCAC | 4[125] | 4[103] |
| GAGCTTAAGAGGTCCCAATTCTGCAATTCCATATAACAGTATATATTTCCTCTACCACCTACATCAC | 4[228] | 7[230] |

| | | |
|---|---|---|
| GAAAGTTCAACAATCAGCTTGCTTAGCTTTAATTGTATCGATATTTCCTCTACCACCTACATCAC | 4[354] | 7[356] |
| CTATTTCGGAACGAGTGAGAATAATATATTTCCTCTACCACCTACATCAC | 4[377] | 4[355] |
| GCAGCACCGTAAGTGCCCGTATAATATATTTCCTCTACCACCTACATCAC | 4[419] | 4[397] |
| TAAGCCAGAGAGCCAGAAGGAAACTCGATAGCCGAACAAAATATTTCCTCTACCACCTACATCAC | 4[480] | 7[482] |
| AGCAAGCCGTTTAAGAATTGAGTATATTTCCTCTACCACCTACATCAC | 4[503] | 4[481] |
| ACCGCATTCCAACGGTATTCTAAGCGAGATATAGAAGGCTATATTTCCTCTACCACCTACATCAC | 4[522] | 7[524] |
| TGACCTAAATTTTTAAACCAAGTATATTTCCTCTACCACCTACATCAC | 4[545] | 4[523] |
| TCAGAGGTGTGTCGGCCAGAATGAGTGCACTCTGTGGTATATATTTCCTCTACCACCTACATCAC | 4[60] | 7[62] |
| ACAGTTTTTCAGATTTCAATTACCGTCGCAGAGGCGAATTATATATTTCCTCTACCACCTACATCAC | 4[606] | 7[608] |
| CAAATATCAAACCAGATGAATATATATTTCCTCTACCACCTACATCAC | 4[629] | 4[607] |
| GCATCGAGCCAGATATCTTTAGGACCTGAGGAAGGTTATCATATTTCCTCTACCACCTACATCAC | 4[648] | 7[650] |
| TACTTCTTTGATAAAAATCTAAAATATTTCCTCTACCACCTACATCAC | 4[671] | 4[649] |
| CATAATATTCCGTAATGGGATCCGTGCATCTGCCAATATTTCCTCTACCACCTACATCAC | 5[119] | 4[126] |
| TGTAAATCATGCTCCTTTTGATAATTGCTGAATATATATATTTCCTCTACCACCTACATCAC | 5[203] | 4[210] |
| CGCCTGACGGTAGAAAGATTCTAATGCAGATACATATATATTTCCTCTACCACCTACATCAC | 5[245] | 4[252] |
| GCGAAAGACGCAAAGCCGCCACGGGAACATATTTCCTCTACCACCTACATCAC | 5[287] | 4[294] |
| TTCATTTTCTGCTAAACAACTGAACAACTAAAGGAATATTTCCTCTACCACCTACATCAC | 5[329] | 4[336] |
| ATCAGAGCCTTTAACGGGGTCTTAATGCCCCCTGCATATATTTCCTCTACCACCTACATCAC | 5[371] | 4[378] |
| TTGAGAATATCTTTCCTTATCACTCATCGAGAACAATATTTCCTCTACCACCTACATCAC | 5[497] | 4[504] |
| ATACCCTTCGTGCCACGCTGAACCTTGCTGAACCTATATTTCCTCTACCACCTACATCAC | 5[623] | 4[630] |
| AACGTTGTAGAAACAGCGGATAGTTGGGCGGTTGTATATATTTCCTCTACCACCTACATCAC | 5[77] | 4[84] |
| GAAAGATCGCACTCCAGCCAGCTATATTTCCTCTACCACCTACATCAC | 7[105] | 7[127] |
| TTCCGAATTGTAAACGTGTCGCCAGCATCGGTGCGGGCCTATATTTCCTCTACCACCTACATCAC | 7[128] | 8[112] |
| GGCTAAAGTACGGTGTCTGGAAGATATATTTCCTCTACCACCTACATCAC | 7[189] | 7[211] |
| TTTCACGAGAATGACCATTTTCATTTGGTCAATAACCTGTATATATTTCCTCTACCACCTACATCAC | 7[212] | 8[196] |
| TGCAACACTATCATAACCCTCGTATATATTTCCTCTACCACCTACATCAC | 7[231] | 7[253] |
| TTACCAATAAGGCTTGCAGTGCGGAAGTTTAGACTGGATAATATATTTCCTCTACCACCTACATCAC | 7[254] | 8[238] |
| AATCCAAAAAAAAGGCTCCAAAAATATATTTCCTCTACCACCTACATCAC | 7[315] | 7[337] |
| GTGTATTAAGAGGCTGAGACTCCATATATTTCCTCTACCACCTACATCAC | 7[357] | 7[379] |
| TCAAGCAGAACCACCACTCACTCAGGTAGCCCGGAATAGGATATTTCCTCTACCACCTACATCAC | 7[380] | 8[364] |
| TTGAAGCCCTTTTTAAGAAAAGTATATTTCCTCTACCACCTACATCAC | 7[441] | 7[463] |
| GTTTACCGCGCCCAATAGCAAGCATATTTCCTCTACCACCTACATCAC | 7[483] | 7[505] |
| AAATCAGCCAGTAATAACACTATTTTTGAAGCCTTAAATCATATTTCCTCTACCACCTACATCAC | 7[506] | 8[490] |
| ATTTGGCAAATCAACAGTTGAAAATATTTCCTCTACCACCTACATCAC | 7[609] | 7[631] |
| GGAATAACAGAGATAGACATACAAACTTGAGGATTTAGAAATATTTCCTCTACCACCTACATCAC | 7[632] | 8[616] |
| GCCCGCACAGGCGGCCTTTAGTGATATATTTCCTCTACCACCTACATCAC | 7[674] | 8[658] |
| ATGAATCCCAGTCACGATCGAACGTGCCGGCCAGAGCACAATATATTTCCTCTACCACCTACATCAC | 7[86] | 8[70] |
| CTTTTTTTCGTCTCGTCGCTGGCATATATTTCCTCTACCACCTACATCAC | 8[111] | 8[89] |
| TCAGGCTGCGCAACTGTTGGGAAATATTTCCTCTACCACCTACATCAC | 8[153] | 8[131] |
| GCTTGACCATTAGATACATTTCGATATATTTCCTCTACCACCTACATCAC | 8[237] | 8[215] |
| ATTTTGCCAGAGGGGGTAATAGTATATATTTCCTCTACCACCTACATCAC | 8[279] | 8[257] |
| TGAACAGCTTGATACCGATAGTTATATATTTCCTCTACCACCTACATCAC | 8[363] | 8[341] |
| GGTGCCGTCGAGAGGGTTGATATATATTTCCTCTACCACCTACATCAC | 8[405] | 8[383] |
| AAAACGGAATACCCAAAAGAACTATATTTCCTCTACCACCTACATCAC | 8[489] | 8[467] |
| AACGAACCTCCCGACTTGCGGGAATATTTCCTCTACCACCTACATCAC | 8[531] | 8[509] |
| GTATTAGAGCCGTCAATAGATAAATATTTCCTCTACCACCTACATCAC | 8[657] | 8[635] |
| **Biotinylated staple strands** | **5'-end** | **3'-end** |
| AACGCCAAAAGGCGGATGGCTTA | 4[251] | 4[229] |
| AAGAAACAATGACCGGAAACGTC | 4[461] | 4[439] |
| GTACATCGACATCGTTAACGGCA | 4[83] | 4[61] |

| | | |
|---|---|---|
| ATACCACCATCAGTGAGGCCAAACCGTTGTAGCAA | 5[665] | 4[672] |

**Table S7** Imager strands used for external labelling of the 12HB brightness nanoruler. All imager strands are modified on the 3'-end with different fluorophores. Docking sequences of the imager strands are marked in red.

| Sequence (5\` to 3\`) | Fluorophore label on 3´ | Docking length [nt] |
|---|---|---|
| GGTGGTAGAGGAATT | ATTO655 | 13 |
| GGTGGTAGAGGAATT | ATTO542 | 13 |
| GTGATGTAGGTGGTAGAGGAA | ATTO655 | 20 |

### 1.11. Repair of kinked 12HB nanoruler

To emulate a structural damage in a 12HB nanoruler, we folded the DNA origami while leaving out 9 staple strands (Table S9) in the central region of the linear nanostructure resulting in a single stranded scaffold strand across all 12 helices (Figure 2 main text). To investigate the resulting 12HB nanoruler via DNA PAINT, we exchanged 60 staple strands with DNA PAINT staple strands given in Table S8. The DNA PAINT staple strands that were used exhibit the docking site sequence complementary to the 8 nt DNA PAINT imagers in Table S5. The docking sites were equally distributed on two sides along the 200 nm axis of the 12HB in ca. 7 nm distances to visualize the overall contour shape of the 12HB nanorulers.

In a first folding process using the folding Program 1 in Table S3, the 12HB was folded while leaving out the nine staple strands given in Table S9. After purification, one part of the sample solution was examined with AFM and DNA PAINT. The other part of the sample solution was folded in a second step with a mix of the 9 missing staple strands in 300x excess using the folding program 1, but starting from T=50°C to accelerate the incorporation of the missing staples but also to not degrade the already folded 12HB nanorulers. After purification the repaired sample solution could be analysed using AFM and DNA PAINT. Additionally, incorporation studies were performed. Therefore, three of the nine later added staple strands were labelled with Cy5. Via widefield imaging of the same field of view in red channel (incorporated Cy5 staple strands) and subsequent DNA PAINT imaging in yellow channel (Cy3B imager strand) quantitative incorporation of the missing staple strands could be probed. For DNA PAINT experiments, 1 nM solution of the 8 nt ATTO655 or Cy3B imager strands was used.

**Table S8** DNA PAINT staples for the visualization of the contour of the kinked 12HB nanoruler with 9 missing staple strands. All DNA PAINT staples exhibit at their 3'-ends the 8 nt docking site sequence for the 8 nt imager strands in Table S5. The numbers for the 5'- end 3'-end of the staples represent the helix number in the corresponding caDNAno file. Number in brackets represents the starting and ending position of the staple in the corresponding helix. For immobilization, the 3'-biotinylated staple strands are used.

| DNA PAINT staple strands | 5'-end | 3'-end |
|---|---|---|
| ATCAGCGGGGTCAGCTTTCAGAGTTAAATGCCCG | 3[56] | 3[78] |
| TTTTTTGTTTAATAAAGTAATTCTTAAATGCCCG | 3[476] | 3[498] |
| GTCACCAGTACAAGGTTGAGGCATTAAATGCCCG | 3[350] | 3[372] |
| AACTTTAATCATGGGTAGCAACGTTAAATGCCCG | 3[266] | 3[288] |
| TTCCATTGACCCAAAGAGGCTTTGAGGATTAAATGCCCG | 2[307] | 3[307] |
| ACGCGTCGGCTGTAAGACGACGACAATATTAAATGCCCG | 2[517] | 3[517] |
| TTCGCCATAAACTCTGGAGGTGTCCAGCTTAAATGCCCG | 2[55] | 3[55] |
| ATTAAAATAAGTGCGACGATTGGCCTTGTTAAATGCCCG | 2[391] | 3[391] |
| GCTAAATCGGTTTGACTATTATATTAAATGCCCG | 3[182] | 3[204] |
| TATGCATTACAGAGGATGGTTTAATTTCTTAAATGCCCG | 2[265] | 3[265] |
| AAATAGGTAATTTACAAATAAGAAACGATTAAATGCCCG | 2[475] | 3[475] |
| CCGAACTTTAATAAAAGCAAAGCGGATTTTAAATGCCCG | 2[223] | 3[223] |
| AGGGACAAAATCTTCCAGCGCCAAAGACTTAAATGCCCG | 2[433] | 3[433] |
| CTAAAGACTTTTAGGAACCCATGTTAAATGCCCG | 3[308] | 3[330] |
| AAGACGCTGAGACCAGAAGGAGCTTAAATGCCCG | 3[560] | 3[582] |
| AACAACATGTTCATCCTTGAAAATTAAATGCCCG | 3[518] | 3[540] |
| AAAAGGGCGACAATTATTTATCCTTAAATGCCCG | 3[434] | 3[456] |
| TGTAGGGGATTTAGTAACACTGAGTTTCTTAAATGCCCG | 2[349] | 3[349] |
| ATATTCACAAACAAATTCATATGTTAAATGCCCG | 3[392] | 3[414] |
| GAATTATCCAATAACGATAGCTTAGATTTTAAATGCCCG | 2[559] | 3[559] |
| TTATGGCCTGAGCACCTCAGAGCATAAATTAAATGCCCG | 2[181] | 3[181] |

| | | |
|---|---|---|
| GCATCAAAAAGAAGTAAATTGGGTTAAATGCCCG | 3[224] | 3[246] |
| TTCGCGGATTGATTGCTCATTTTTTAACTTAAATGCCCG | 2[139] | 3[139] |
| GATAGTGCAACATGATATTTTTGAATGGTTAAATGCCCG | 2[643] | 3[643] |
| GGATAACCTCACAATTTTTGTTATTAAATGCCCG | 3[98] | 3[120] |
| TCAATAATAAAGTGTATCATCATATTCCTTAAATGCCCG | 2[601] | 3[601] |
| CAATAGGAACGCAAATTAAGCAATTAAATGCCCG | 3[140] | 3[162] |
| GCGAAAGACGCAAAGCCGCCACGGGAACTTAAATGCCCG | 2[97] | 3[97] |
| TGATTATCAGATATACGTGGCACTTAAATGCCCG | 3[602] | 3[624] |
| CTATTAGTCTTTCGCCGCTACAGTTAAATGCCCG | 3[644] | 3[666] |
| AAGCACAGAGCCTAATTATTGTTAGCGATTAAGACTCCTTTTAAATGCCCG | 7[464] | 8[448] |
| TGAACAGCTTGATACCGATAGTTTTAAATGCCCG | 8[363] | 8[341] |
| AAATCAGCCAGTAATAACACTATTTTTGAAGCCTTAAATCTTAAATGCCCG | 7[506] | 8[490] |
| GGTGCCGTCGAGAGGGTTGATATTTAAATGCCCG | 8[405] | 8[383] |
| AACGAACCTCCCGACTTGCGGGATTAAATGCCCG | 8[531] | 8[509] |
| CCGAACGGTGTACAGACCAGGCGTTAAATGCCCG | 8[321] | 8[299] |
| GGAGCAGCCACCACCCTTCGCATAACGACAATGACAACAATTAAATGCCCG | 7[338] | 8[322] |
| TTTCACGAGAATGACCATTTTCATTTGGTCAATAACCTGTTTAAATGCCCG | 7[212] | 8[196] |
| TCGGTCATACCGGGGGTTTCTGCTTAAATGCCCG | 8[69] | 8[47] |
| TTACCAATAAGGCTTGCAGTGCGGAAGTTTAGACTGGATATTAAATGCCCG | 7[254] | 8[238] |
| AAAACGGAATACCCAAAAGAACTTTAAATGCCCG | 8[489] | 8[467] |
| AGACGTCGTCACCCTCAGATCTTGACGCTGGCTGACCTTCTTAAATGCCCG | 7[296] | 8[280] |
| AAATGCGTTATACAAATTCTTACTTAAATGCCCG | 8[573] | 8[551] |
| TAAGATCTGTAAATCGTTGTTAATTGTAAAGCCAACGCTCTTAAATGCCCG | 7[548] | 8[532] |
| ATGAATCCCAGTCACGATCGAACGTGCCGGCCAGAGCACATTAAATGCCCG | 7[86] | 8[70] |
| ATTCTTTTCATAATCAAAATCACTTAAATGCCCG | 8[447] | 8[425] |
| AATCGTTGAGTAACATTGGAATTACCTAATTACATTTAACTTAAATGCCCG | 7[590] | 8[574] |
| ATTTTGCCAGAGGGGGTAATAGTTTAAATGCCCG | 8[279] | 8[257] |
| AGCGCCACCACGGAATACGCCTCAGACCAGAGCCACCACCTTAAATGCCCG | 7[422] | 8[406] |
| TCAAGCAGAACCACCACTCACTCAGGTAGCCCGGAATAGGTTAAATGCCCG | 7[380] | 8[364] |
| CTTTTTTTCGTCTCGTCGCTGGCTTAAATGCCCG | 8[111] | 8[89] |
| GTTGAAACAAACATCAAGAAAACTTAAATGCCCG | 8[615] | 8[593] |
| GCTTGACCATTAGATACATTTCGTTAAATGCCCG | 8[237] | 8[215] |
| TTAACAAGAGAATCGATGAACGGTTAAATGCCCG | 8[195] | 8[173] |
| GTATTAGAGCCGTCAATAGATAATTAAATGCCCG | 8[657] | 8[635] |
| TCAGGCTGCGCAACTGTTGGGAATTAAATGCCCG | 8[153] | 8[131] |
| TTTTTATCCAATAAATCTCTACCCCGGTAAAACTAGCATGTTAAATGCCCG | 7[170] | 8[154] |
| GGAATAACAGAGATAGACATACAAACTTGAGGATTTAGAATTAAATGCCCG | 7[632] | 8[616] |
| TTCCGAATTGTAAACGTGTCGCCAGCATCGGTGCGGGCCTTTAAATGCCCG | 7[128] | 8[112] |
| GCCTTACGCTGCGCGTAAAATTATTTTTTGACGCTCAATCTTAAATGCCCG | 7[674] | 8[658] |
| **Biotinylated staple strands** | **5'-end** | **3'-end** |
| AACGCCAAAAGGCGGATGGCTTA | 4[251] | 4[229] |
| AAGAAACAATGACCGGAAACGTC | 4[461] | 4[439] |
| GTACATCGACATCGTTAACGGCA | 4[83] | 4[61] |
| ATACCACCATCAGTGAGGCCAAACCGTTGTAGCAA | 5[665] | 4[672] |

**Table S9** Left out staples to emulate a structural damage in a 12HB nanoruler. For incorporation studies, three staples were labelled with Cy5 on their 5'-end (marked in red). The numbers for the 5'- end 3'-end of the staples represent the helix number in the corresponding caDNAno file, while the numbers in brackets represent the starting and ending position of the staple in the corresponding helix.

| Missing staple strands | 5'-end | 3'-end |
|---|---|---|

| | | |
|---|---|---|
| (Cy5)-GGTGCCGTCGAGAGGGTTGATAT | 8[405] | 8[383] |
| TACCTGGTTTGCCCCAGCA | 10[373] | 11[386] |
| AAGTAAGAGCCGCCAGTACCAGGCGG | 8[382] | 9[398] |
| (Cy5)-ATTAAAATAAGTGCGACGATTGGCCTTG | 2[391] | 3[391] |
| ATCAGAGCCTTTAACGGGGTCTTAATGCCCCCTGC | 5[371] | 4[378] |
| GGCGACACCACCCTCAGGTTGTACTGTACCGTTCCAGTAA | 11[387] | 0[371] |
| TCAAGCAGAACCACCACTCACTCAGGTAGCCCGGAATAGG | 7[380] | 8[364] |
| AACAGAGTGCCTGGGGTTTTGCTCACAGAAGGATTAGGAT | 4[396] | 7[398] |
| (Cy5)-GAATTGTAGCCAGAATGGATCAGAGCAAATCCT | 0[389] | 11[398] |

### 1.12. Staple strand exchange from dual spot to triple spot in NRO nanoruler

To probe potential exchange of staple strands within a DNA origami with staple strands from solution, we designed an NRO DNA PAINT nanoruler with initially two DNA PAINT labelling spots, each consisting of 3 docking sites, with a distance of 40 nm. Therefore, we exchanged the 6 unmodified staple strands with the DNA PAINT staple strands given in Table S10. After folding and purification, the dual spot NRO could be imaged with the DNA PAINT imagers from Table S5.
For probing the exchange of staple strands within the DNA origami with staples from solution, we incubated the dual spot NRO with a mix of three DNA PAINT staple strands, which are selected to form a third label spot for DNA PAINT in 40 and 70 nm distance to the initial two spots, resulting in a triangle-shaped triple spot. To accelerate the incorporation, we used an 300x excess of the invasive DNA PAINT staple strands with respect to the dual spot NRO in the corresponding folding buffer (Table S1) and put the sample solution into the NRO folding program (Table S4) starting from just T= 50°C to prevent melting of the already folded dual spot NRO. In order to also emulate partially damaged staple strands, we folded the initial dual spot NRO with shorter unmodified staple strands, which are to be displaced by the invasive DNA PAINT staple strands. We used 3 initial staple strands, which are in one case 4 nt too short, in a second case 8 nt too short, resulting in a 4 nt and 8 nt toehold on the scaffold strand. For all three cases of initial staple strands (0, 4, 8 nt scaffold toeholds), the initial dual spot NROs were incubated with the invasive DNA PAINT staple strands as mentioned above.

To examine the invasion of the DNA PAINT staple strands, DNA PAINT experiments with 5 nM solution of the 8 nt ATTO655 imager strands were conducted.

**Table S10** DNA PAINT staples for a double spot NRO nanoruler (2x3 docking sites) and invasive staple strands forming a third labelling spot. The three initial staple strands designed to be displaced by invasive third label spot staple strands exhibited 0 to 8 nt shortened sequences to establish toeholds in the scaffold strand (left out sequences highlighted in green and blue). All DNA PAINT staples exhibit at their 3'-ends the 8 nt docking site sequence for the 8 nt imager strands in Table S5 (highlighted in red). The numbers for the 5'- end 3'-end of the staples represent the helix number in the corresponding caDNAno file. Number in brackets represents the starting and ending position of the staple in the corresponding helix. For immobilization, the 3'-biotinylated staple strands are used.

| Dual spot DNA PAINT staple strands | 5'-end | 3'-end |
|---|---|---|
| TCAATATCGAACCTCAAATATCAATTCCGAAATTAAATGCCCG | 21[160] | 22[144] |
| TCGGCAAATCCTGTTTGATGGTGGACCCTCAATTAAATGCCCG | 22[143] | 21[159] |
| AACGTGGCGAGAAAGGAAGGGAAACCAGTAATTAAATGCCCG | 23[128] | 23[159] |
| GTTTATTTTGTCACAATCTTACCGAAGCCCTTTAATATCATTAAATGCCCG | 7[248] | 9[255] |
| GAGAGATAGAGCGTCTTTCCAGAGGTTTTGAATTAAATGCCCG | 9[256] | 11[255] |
| GCCTTAAACCAATCAATAATCGGCACGCGCCTTTAAATGCCCG | 11[256] | 13[255] |
| **Triple spot staple strands** | **5'-end** | **3'-end** |
| ACAAACGGAAAAGCCCCAAAAACACTGGAGCA-TTAAATGCCCG | 16[47] | 14[48] |
| AACAAGAGGGATAAAAATTTTTAGCATAAAGC-TTAAATGCCCG | 14[47] | 12[48] |
| TAAATCGGGATTCCCAATTCTGCGATATAATG-TTAAATGCCCG | 12[47] | 10[48] |
| **Biotinylated staple strands** | **5'-end** | **3'-end** |
| CGGATTCTGACGACAGTATCGGCCGCAAGGCGATTAAGTT | 16[63] | 18[56] |
| ATAAGGGAACCGGATATTCATTACGTCAGGACGTTGGGAA | 4[63] | 6[56] |
| GAAACGATAGAAGGCTTATCCGGTCTCATCGAGAACAAGC | 10[191] | 12[184] |
| TAGAGAGTTATTTTCATTTGGGGATAGTAGTAGCATTA | 10[127] | 12[120] |
| AGCCACCACTGTAGCGCGTTTTCAAGGGAGGGAAGGTAAA | 4[255] | 6[248] |
| GAGAAGAGATAACCTTGCTTCTGTTCGGGAGAAACAATAA | 16[255] | 18[248] |

### 1.13. Self-Healing of 12HB nanoruler in degrading conditions

For probing self-healing processes of a DNA origami nanoruler in degrading conditions, we designed a triple-spot 12HB nanoruler suitable for DNA PAINT imaging using the 6 or 8 nt imager strands shown in Table S5. We designed three labeling spots on the 12HB origami with 107 and 70 nm interspot distances, by exchanging 3x10 staple strands by the corresponding DNA PAINT staple strands in Table S11. The DNA PAINT staple strands exhibit a 10 nt long docking site for DNA PAINT experiments, which show complementary sequence to the used imager strands.

Self-Healing studies in degrading conditions were performed by incubation of the immobilized DNA PAINT nanorulers in the folding buffer of 12HB (Table S1) containing additionally 0.2% (vol) or 10% (vol) fetal bovine serum (FBS) from ThermoFisher Scientific. For testing the stabilizing effect of random additional oligonucleotide sequences, we added a mix of unmodified staples strands from the 6HB origami (Table S17) which do not show any significant overlap to the scaffold strand of the 12HB origami. Therefore, we added the mix of 146 different oligonucleotides to the incubation buffer in final overall staple concentration of 5 µM, 500 nM or 50 nM (i.e. 34.2 nM, 3.2 nM or 0.3 nM per strand). To test the self-healing effect provided by staple strands, we added in another incubation experiment a mix of the 222 unmodified 12HB staple strands to the FBS solution. The unmodified 12HB staple strands (Table S15) were added at 5 µM, 500 nM or 50 nM overall staple concentration (i.e. 22.5 nM, 2.2 nM or 0.2 nM per strand). While incubating with the FBS buffer containing no added DNA, orthogonal staples and the 12HB staples, the samples were stored at room temperature. Before every DNA PAINT imaging experiment, the incubation solution was taken off the sample and the surface was washed three times with plane imaging buffer (1x PBS, 12.5 mM $MgCl_2$). After DNA PAINT imaging, the imaging buffer was washed away three times and new incubation solution was applied. For experiments with 0.2% FBS, a 2 nM solution of the 8 nt ATTO655 imaging strand was used in DNA PAINT experiments. For studies at higher damage rates in 10% FBS, a 10 nM solution of the 6 nt ATTO655 imager strand was used in DNA PAINT experiments.

**Table S11** Sequences of staple strands used to produce triple-spots in 12HB nanoruler. All DNA PAINT staples exhibit at their 3'-ends the 8 nt docking site sequence for the 8 nt imager strands in Table S5 (highlighted in red). The numbers for the 5'- end 3'-end of the staples represent the helix number in the corresponding caDNAno file. Numbers in brackets represent the starting and ending position of the staple in the corresponding helix. For immobilization, the 3' biotinylated staple strands are used.

| Triple spot DNAPAINT staple strands | 5'-end | 3'-end |
|---|---|---|
| GTATGTGAAATTGTTATCCTTAAATGCCCG | 10[79] | 11[92] |
| CTTTTTTTCGTCTCGTCGCTGGCTTAAATGCCCG | 8[111] | 8[89] |
| CCAGCCAAACTTCTGATTGCCGTTTTGGGTAAAGTTAAACTTAAATGCCCG | 4[102] | 7[104] |
| GCCCGCACAGGCGGCCTTTAGTGTTAAATGCCCG | 7[63] | 7[85] |
| CCGGAAGACGTACAGCGCCGCGATTACAATTCCTTAAATGCCCG | 0[95] | 11[104] |
| TAAAGGATTGTATAAGCGCACAAACGACATTAAATGTGAGTTAAATGCCCG | 11[135] | 0[119] |
| GGATAACCTCACAATTTTTGTTATTAAATGCCCG | 3[98] | 3[120] |
| GCGAAAGACGCAAAGCCGCCACGGGAACTTAAATGCCCG | 2[97] | 3[97] |
| TTCCGAATTGTAAACGTGTCGCCAGCATCGGTGCGGGCCTTTAAATGCCCG | 7[128] | 8[112] |
| CGAGTAACAACCGTTTACCAGTCTTAAATGCCCG | 0[118] | 0[96] |
| GATGTTTTTCTTTTCACCATTAAATGCCCG | 10[289] | 11[302] |
| CCGAACGGTGTACAGACCAGGCGTTAAATGCCCG | 8[321] | 8[299] |
| GGAGCAGCCACCACCCTTCGCATAACGACAATGACAACAATTAAATGCCCG | 7[338] | 8[322] |
| TTCCATTGACCCAAAGAGGCTTTGAGGATTAAATGCCCG | 2[307] | 3[307] |
| CTAAAGACTTTTAGGAACCCATGTTAAATGCCCG | 3[308] | 3[330] |
| ACTACCTTTAAACGGGTAACAGGGAGACGGGCATTAAATGCCCG | 0[305] | 11[314] |
| GAGAGCCTCAGAACCGCATTTTCTGTAACGATCTAAAGTTTTAAATGCCCG | 11[345] | 0[329] |
| ATTTGCCAAGCGGAACTGACCAACGAGTCAATCATAAGGGTTAAATGCCCG | 4[312] | 7[314] |
| TTGTCGTCTTTCTACGTAATGCCTTAAATGCCCG | 0[328] | 0[306] |
| ACTACTTAGCCGGAACGAGGCGCTTAAATGCCCG | 7[273] | 7[295] |
| ACAGTTTTTCAGATTTCAATTACCGTCGCAGAGGCGAATTTTAAATGCCCG | 4[606] | 7[608] |
| ATTTGGCAAATCAACAGTTGAAATTAAATGCCCG | 7[609] | 7[631] |
| TTCTGGAATAATCCTGATTTTGCCCGGCCGTAATTAAATGCCCG | 0[599] | 11[608] |
| GTATTAGAGCCGTCAATAGATAATTAAATGCCCG | 8[657] | 8[635] |
| CCGATAATAAAAGGGACTTAACACCGCGAACCACCAGCAGTTAAATGCCCG | 11[639] | 0[623] |
| GGAATAACAGAGATAGACATACAAACTTGAGGATTTAGAATTAAATGCCCG | 7[632] | 8[616] |
| GATAGTGCAACATGATATTTTTGAATGGTTAAATGCCCG | 2[643] | 3[643] |

| | | |
|---|---|---|
| AACACCCTAAAGGGAGCCCTTAAATGCCCG | 10[625] | 11[638] |
| GATTTTAGACAGGCATTAAAAATATTAAATGCCCG | 0[664] | 0[642] |
| TGATTATCAGATATACGTGGCACTTAAATGCCCG | 3[602] | 3[624] |
| **Biotinylated staple strands** | **5'-end** | **3'-end** |
| AACGCCAAAAGGCGGATGGCTTA | 4[251] | 4[229] |
| AAGAAACAATGACCGGAAACGTC | 4[461] | 4[439] |
| GTACATCGACATCGTTAACGGCA | 4[83] | 4[61] |
| ATACCACCATCAGTGAGGCCAAACCGTTGTAGCAA | 5[665] | 4[672] |

### 1.14. Self-Regeneration and Self-Healing of an enzymatically cleavable brightness label on 6HB brightness nanoruler

For establishing a self-regenerating brightness label on a 6HB nanoruler by enzymatic cleavage, we designed 2x10 docking sites for the external labeling of the 6HB structure. The two labeling spots were distributed along the 400 nm axis of the 16HB with a 290 nm inter-spot distance. Therefore, we exchanged the 2x10 unmodified core staple strands of the 6HB in caDNAno with the docking site staple strands given in Table S12. The 20 nt ATTO655 imager strand sequence (Table S14) was designed to exhibit the specific *Nb.BtsI* binding sequence *CACTGC*, so that the enzyme could bind to a labeled imager strand and cut it into two 10 nt fragments.

6HB brightness rulers were immobilized and externally labeled in 1x PBS with 12.5 mM $MgCl_2$ and 5 nM ATTO655 imager strand (Table S14) over 1 hour. Excessive imager strands were washed away. Enzymatic cleavage of imager strands bound to the docking sites on the 6HB was realized in 1x CutSmart® buffer with 12.5 mM $MgCl_2$ and 100 units/ml *Nb.BtsI*. For probing the activity of the restriction enzyme, internally labeled 6HB brightness rulers were immobilized, externally labeled and imaged. Then the enzyme was added and the sample was imaged after one night of incubation. After washing the enzyme away, the immobilized brightness rulers were again externally labeled and imaged. Brightness values were extracted for individual DNA origami, averaged and normalized to initial brightness.
For a self-healing label by enzymatic cleavage, immobilized 6HB brightness rulers were incubated with 1x CutSmart® buffer with 12.5 mM $MgCl_2$,100 units/ml *Nb.BtsI* and 5 nM of imager strands simultaneously. After waiting for steady-state conditions for 30 minutes, the self-healing label could be imaged.

**Table S12** The sequences of staple strands used to realize a 6HB brightness ruler for widefield imaging. Dual spot staple strands exhibit a 20 nt docking site sequence with binding site for *Nb.BtsI*. The numbers for the 5'- end 3'-end of the staples represent the helix number in the corresponding caDNAno file. Numbers in brackets represent the starting and ending position of the staple in the corresponding helix. For immobilization, the 3' biotinylated staple strands are used.

| Dual spot staple strands | 5'-end | 3'-end |
|---|---|---|
| CAGATTCAGTACCGCATTCCAAGAACGGTTGTAGAAACCAATTTTTTACTAGCAGTGATCTTAGCAT | 5[1134] | 2[1134] |
| CAATAATCGGCTAGAATATCCCATCCTAGTCCTGAACATTGGTTTTTACTAGCAGTGATCTTAGCAT | 2[1133] | 5[1133] |
| ATAGCAAGCAAAATGAATCATTACCGCGATTTTATTTTCATCTTTTTACTAGCAGTGATCTTAGCAT | 3[1106] | 1[1119] |
| ACGTAATTTAGGCAAAAGTACCGACAAAAAAAGGGTAAAGCCTTTTTACTAGCAGTGATCTTAGCAT | 1[1162] | 4[1162] |
| AACGCGCCTGTTTAACACGACCAGTAATAGGTAAAGTAATATTTTTTACTAGCAGTGATCTTAGCAT | 4[1161] | 0[1148] |
| GCTAATGCAGAACGCAATAAACAACATGGTTCTGTCCAGACGTTTTTACTAGCAGTGATCTTAGCAT | 3[1148] | 1[1161] |
| ATGTATCATATGCGGAACCCTTCTGACCATAAGGCGTTAAAGTTTTTACTAGCAGTGATCTTAGCAT | 4[1203] | 0[1190] |
| GTGTCTTTCCTTATCACTCATCGAGAACATTATTTACAAGAATTTTTACTAGCAGTGATCTTAGCAT | 1[1120] | 4[1120] |
| GGCCAACAGAATATGAGGCATTTTCGAGAGCGCCATATTTAATTTTTACTAGCAGTGATCTTAGCAT | 5[1176] | 2[1176] |
| CAACGCCAACATGACTCAACAGTAGGGCACCAGTAACATTCTTTTTTACTAGCAGTGATCTTAGCAT | 2[1175] | 5[1175] |
| ATCTCTGACCTCCTAGTCGGGAAACCTGGCACGAATATAGTTTTTTTACTAGCAGTGATCTTAGCAT | 4[195] | 0[182] |
| GGTTAAGTTGGGTAAACGACGGCCAGTGGGCGGTTATCGGTGTTTTTACTAGCAGTGATCTTAGCAT | 1[238] | 4[238] |
| ACTGCCCGAAATTGTCATGGTCATAGCTAAACGGAGGATCCCTTTTTACTAGCAGTGATCTTAGCAT | 5[168] | 2[168] |
| CGCGCACGACTTAAAACGCGCGGGGAGACCAAGCTTTCTCCCTTTTTACTAGCAGTGATCTTAGCAT | 4[237] | 0[224] |
| CATTTCTCCGAAGAGACGCATTTCACATGTGGGCCTTGAATCTTTTTACTAGCAGTGATCTTAGCAT | 3[182] | 1[195] |
| TTAATGACTGTAAGGATACCGACAGTGCTGTCTAATCTATTTTTTTTACTAGCAGTGATCTTAGCAT | 5[210] | 2[210] |
| GGGAGTGACTCTATCAACTCGTCGGTGGTCGTGCCCAACCTTTTTTTACTAGCAGTGATCTTAGCAT | 1[196] | 4[196] |
| ACGCTCGCCCTGCTCAATGTCCCGCCAAGAATTGTAGCTGCATTTTTACTAGCAGTGATCTTAGCAT | 2[209] | 5[209] |
| GCTGCAAGGCGATGCCTCTTCGCTATTAAAGGGCGTGCGTATTTTTTACTAGCAGTGATCTTAGCAT | 2[251] | 5[251] |

| | | |
|---|---|---|
| TGTGAATTCATGGGGATGTTCTTCTAAGGGAGGAGAAGCCAGTTTTTACTAGCAGTGATCTTAGCAT | 3[224] | 1[237] |
| **Biotinylated staple strands** | **5'-end** | **3'-end** |
| AGATTTAAGTCCACTTAATTGCTGAATAAACTAAAGTACGGG | 0[475] | 3[475] |
| ATTTTTTCAGGGCGTCTTTCCAGACGTTAACAACTTTCAATA | 0[769] | 3[769] |
| TCAGAGGCATTGCAAACGATTTTTTGTTGAGAGAATAACATA | 0[1063] | 3[1063] |
| CCTGTGTGCTTTCCGGTTGGTGTAATGAACCTCGATAAAGAT | 0[181] | 3[181] |
| ATCGCGCACCACCAAATTGCGTAGATTTAGTACCTTTTACAT | 0[1315] | 3[1315] |

**Table S13** Internally labelled staple strands for colocalization experiments probing enzymatic activity of *Nb.BtsI*. The numbers for the 5'- end 3'-end of the staples represent the helix number in the corresponding caDNAno file. Numbers in brackets represent the starting and ending position of the staple in the corresponding helix. For immobilization, the 3' biotinylated staple strands are used.

| Internally labeled staple strands (ATTO532) | 5'-end | 3'-end |
|---|---|---|
| ATTO532-GGAATTATCATCACTTATCATTTTGCGGTTAAAAGAGGCGGT | 2[1343] | 5[1343] |
| ATTO532-ACGCTCGCCCTGCTCAATGTCCCGCCAAGAATTGTAGCTGCA | 2[209] | 5[209] |

**Table S14** Brightness imager strand for 6HB brightness ruler and cleavage site for *Nb.BtsI*. Docking sequence is highlighted in red, *Nb.BtsI* binding sequence in blue, position of cleavage by *Nb.BtsI* is indicated by a slash, respectively.

| Sequence (5` to 3`) | Fluorophore label on 3´ | Docking length (nt) |
|---|---|---|
| ATGCTAAGAT/CACTGCTAGTTT | ATTO655 | 20 |

## 2. Results and Discussion

### 2.1. Self-Regenerating brightness label on 12HB brightness nanoruler

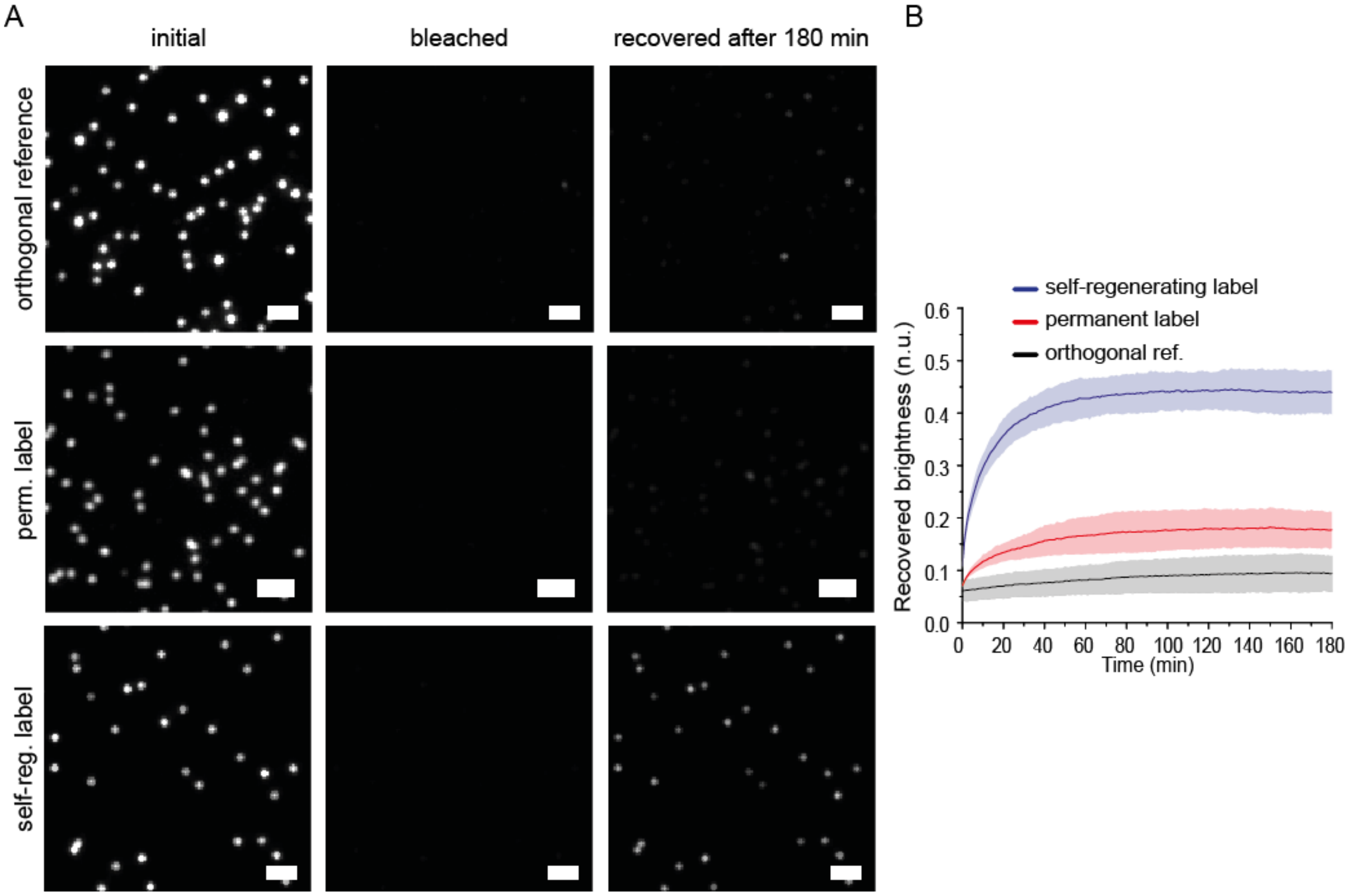


**Figure S2.** (A) Exemplary TIRF images of gradual bleaching and recovery (initial, bleached, recovered after 180 min) of orthogonal permanent imager strand, permanent label and self-regenerating label with ATTO655 (without photostabilization). Samples were bleached over 3 min with 75 W/cm$^2$ excitation at 640 nm. Scale bars represent 2 µm. (B) Corresponding extracted averaged and normalized single DNA origami intensity transients after photobleaching. Self-regenerating labels (blue) show a recovery of around 40%. The permanent label exhibits a small recovery due to post labelling (15%), while an orthogonal imager strand reference shows no significant recovery of brightness. Data represent average of three experiments, highlighted areas represent the standard deviation.

Exemplary TIRF images with initial, bleached and recovered brightness after 180 min for permanent and dynamic brightness labels (ATTO655) are given in Figure S2A. To probe, if a potential recovery is due to unspecific binding of imager strands to the immobilized DNA origami, we also measured the recovery of an orthogonal imager strand, i.e. an oligonucleotide labeled with ATTO655 but with a 20 nt sequence, which is not complementary to the used docking sites. Corresponding extracted and averaged recovery intensity transients per single nanoruler are shown in in Figure S2B. While the orthogonal imager strand exhibited only a very slow, insignificant recovery due to unspecific binding of imager strands to the nanorulers, the permanent label revealed a slightly higher recovery of around 15%. This low recovery could be explained by post-labeling of initially inaccessible docking sites. Accessibility studies of externally labeled DNA origami reveal usually accessibilities in the range of 60 to 90%.[10] After bleaching of permanent labels and subsequent ROS induced damage to the docking sites, initially inaccessible docking sites might become more accessible for intact permanent imager strands from solution. The self-regenerating label though exhibited a significantly improved recovery of around 40% under identical conditions.

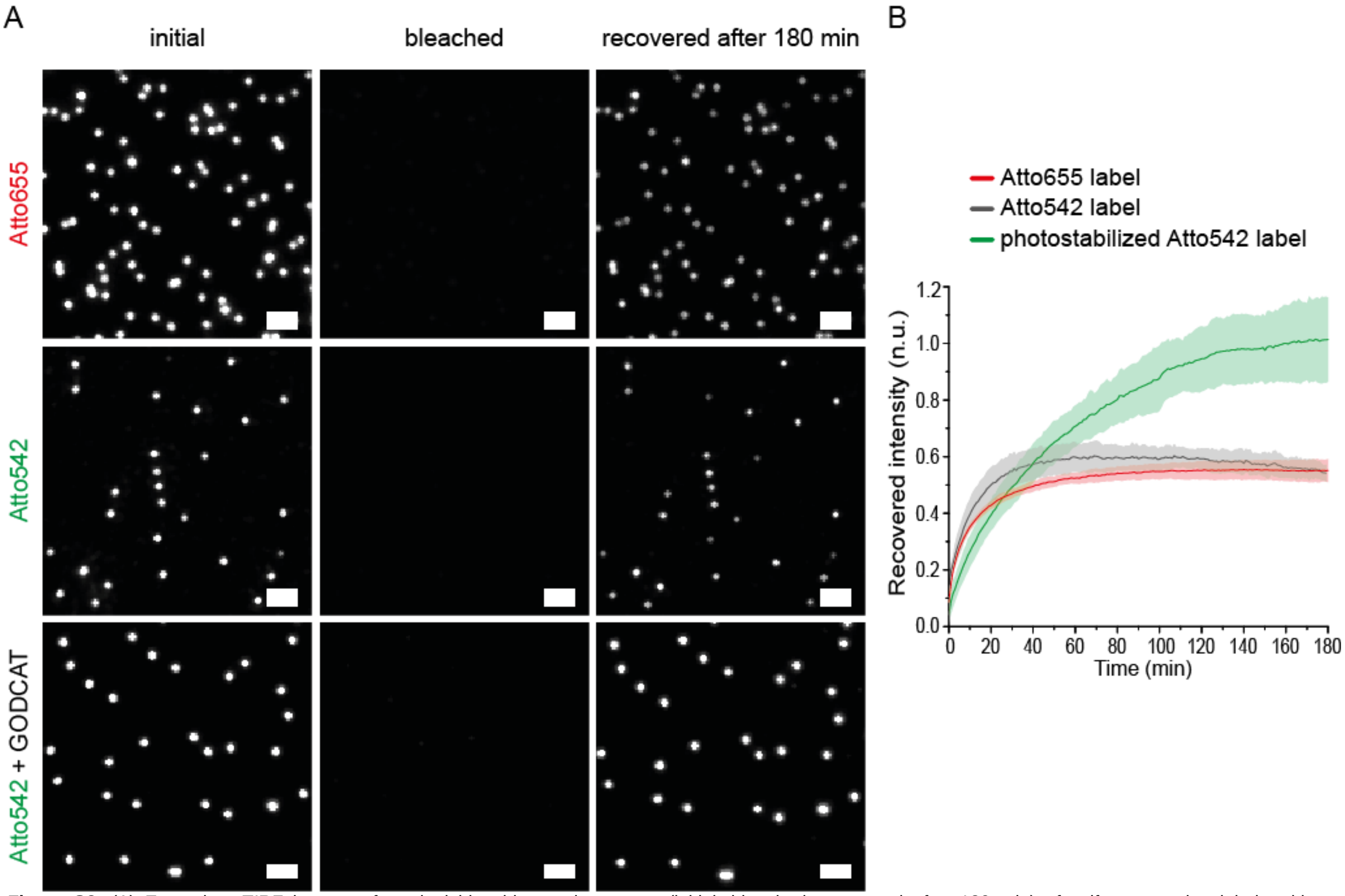


**Figure S3**. (A) Exemplary TIRF images of gradual bleaching and recovery (initial, bleached, recovered after 180 min) of self-regenerating labels without photostabilization (ATTO655 and ATTO542) and self-regenerating label with photostabilization (ATTO542). Samples were bleached over 3 min with 0.5 kW/cm$^2$ excitation power. Scale bars represent 2 µm. (B) Corresponding extracted averaged and normalized single DNA origami intensity transients after photobleaching. Self-regenerating labels without photostabilization (red and grey) show a limited recovery of around 50 to 60%. The photostabilized (GODCAT, Trolox) ATTO542 label exhibits complete recovery of up to 100% of its initial brightness. Data represent average of three experiments, highlighted areas represent the standard deviation.

To overcome limitations by photoinduced damage, we used an imager strand modified with the rhodamine dye ATTO542, which can be photostabilized by an enzymatic oxygen scavenging system and ROXS[5, 11]. For oxygen removal, a 2.5x TAE buffer with glucose, glucose oxidase and catalase was used. To deplete triplet states of the ATTO542 dyes Trolox/Trolox quinone mixture was used as reducing and oxidizing system (ROXS). Gradual bleaching and recovery of the brightness of self-regenerating labels with and without photostabilization is given in Figure S3. In order to bleach the photostabilized labels completely, higher bleaching laser powers at 0.5 kW/cm$^2$ over 3 min were applied. While the self-regenerating ATTO655 and ATTO542 labels without photostabilization showed again limited recovery of only up to 60 %, the photostabilized ATTO542 label revealed a complete recovery of up to 100% of initial brightness. Under the used time lapse imaging conditions and the applied photostabilization, complete repair of the photoinduced damage to the brightness functionality could be realized by self-regenerating labels.

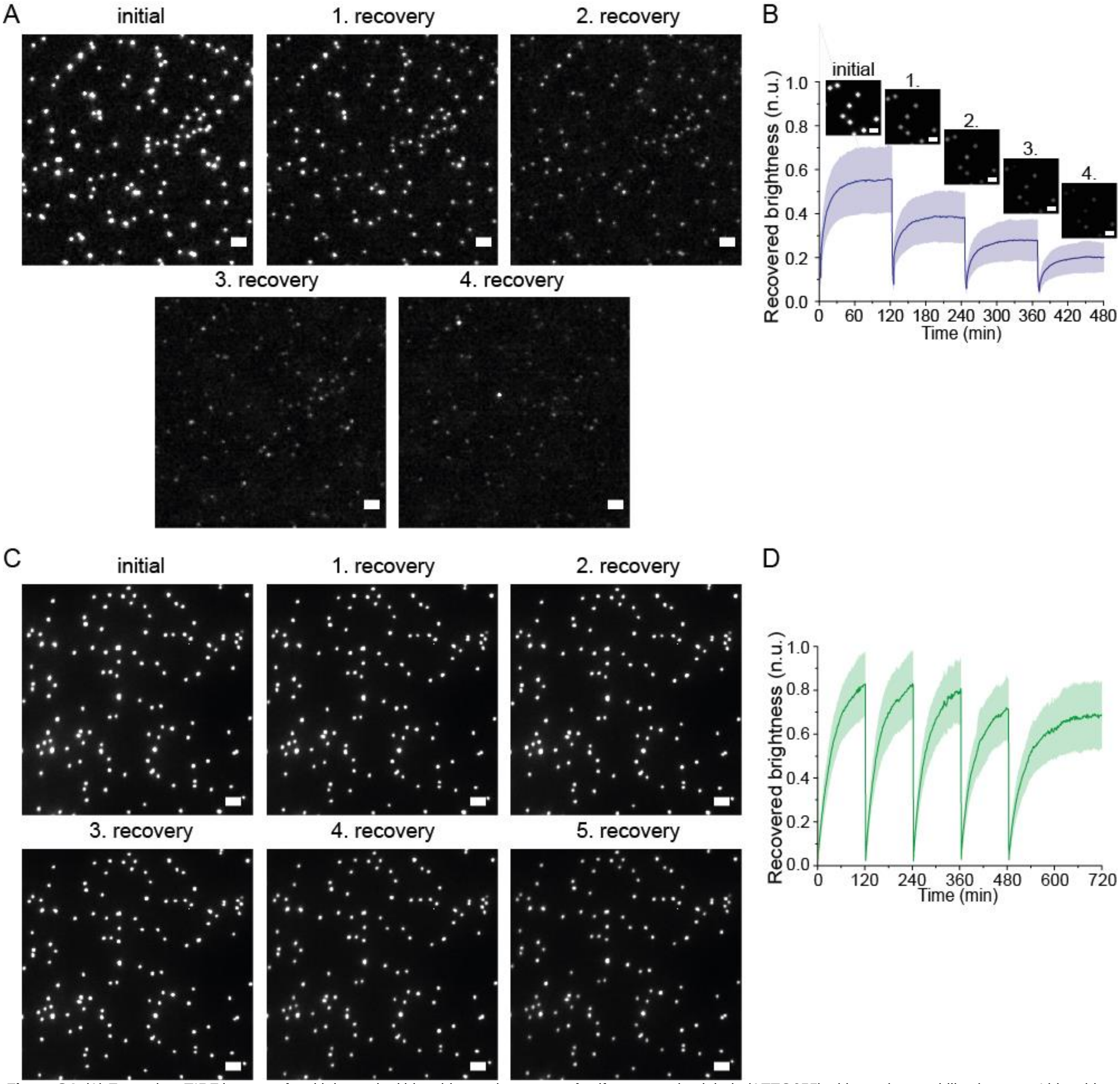


**Figure S4.** (A) Exemplary TIRF images of multiple gradual bleaching and recovery of self-regenerating labels (ATTO655) without photostabilization over 4 bleaching events (3 min bleaching with 75 W/cm$^2$ at 640 nm). Scale bars represent 1 µm. (B) Corresponding extracted averaged and normalized single DNA origami intensity transients over 4 bleaching and recovery cycles. (C) Exemplary TIRF images of multiple gradual bleaching and recovery of self-regenerating label (ATTO542) with photostabilization over 5 bleaching events (3 min bleaching with 0.5 kW/cm$^2$ at 532 nm). Scale bars represent 1 µm. (D) Corresponding extracted averaged and normalized single DNA origami intensity transients over 5 bleaching and recovery cycles. Data represent average of one experiment, highlighted areas represent the standard deviation.

To probe self-regeneration of the labels over multiple damaging events, we bleached the same field of view multiple times and measured the time lapse recovery of the brightness after every bleaching event. Exemplary TIRF images after every bleaching and recovery cycle of the self-regenerating label without photostabilization (Figure S4A) and corresponding extracted average single nanoruler intensity transients in (Figure S4B) revealed that even after 4 bleaching events the self-regenerating labels were able to recover back to over 20% of initial brightness. The photoinduced damage to docking sites by ROS is still clearly visible, since the recovery decreases over every bleaching event from initial ca. 60% to ca. 20%. Analogous multiple bleaching and recovery of the ATTO542 self-regenerating brightness label with photostabilization (Figure S4C) revealed a strongly increased recovery over multiple bleaching events. Under the used imaging parameters (120 min for recovery), the photostabilized self-regenerating label recovered to over 80% for the first three recovery cycles. Only after the fourth bleaching, a decreased recovery of around 70% and 60% after fifth bleaching and hence an increasing damage to the docking sites was visible. Results from Figure S3 indicate a full recovery of the photostabilized self-regenerating label after 180 min. To minimize the chance of defocusing or too much sample drift during data acquisition, the recovery for multiple bleaching events was investigated over 120 min (i.e. before full exchanged had occurred) until next bleaching cycle was initialized.

### 2.2. Repair of kinked 12HB nanoruler

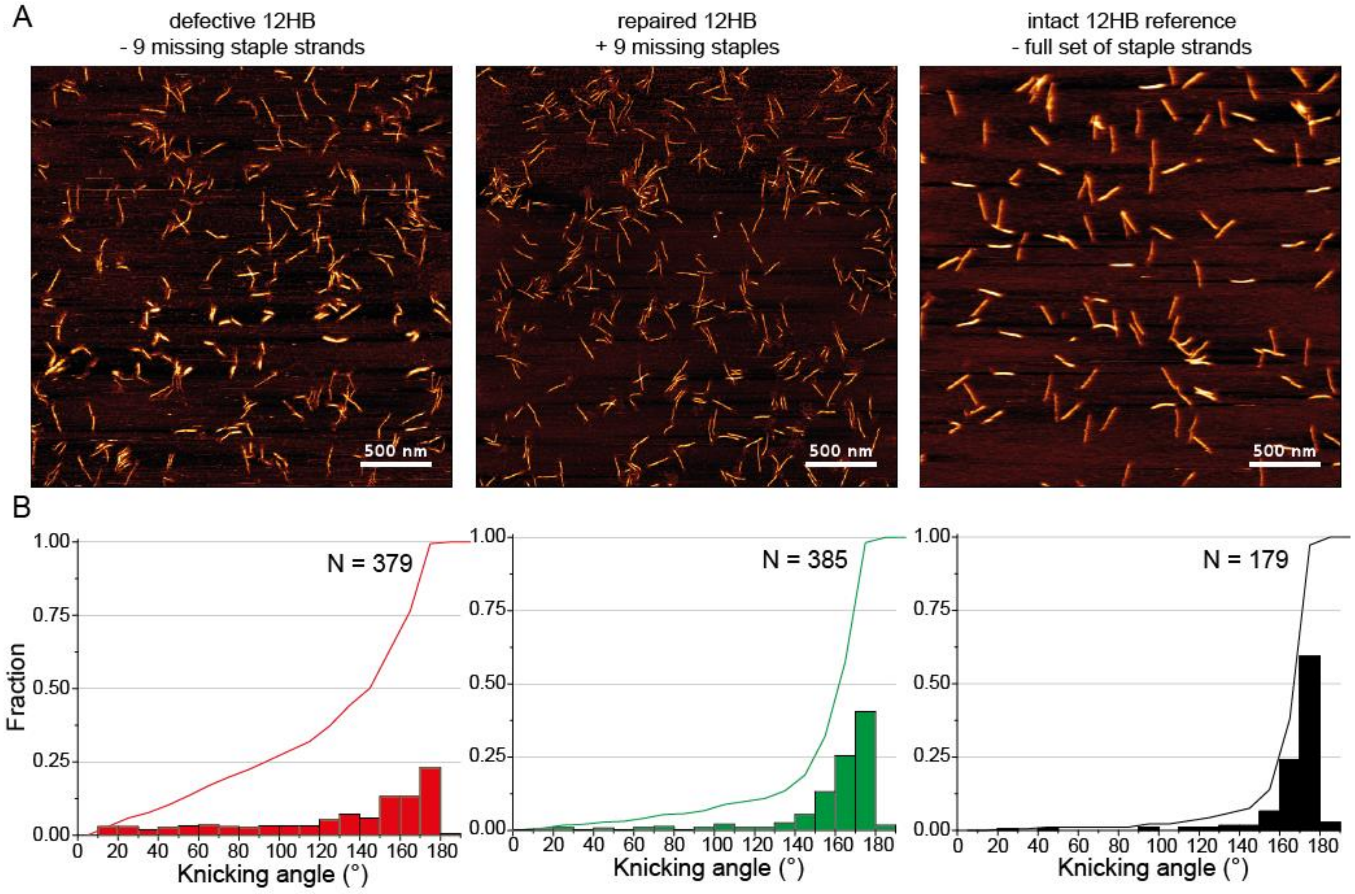


**Figure S5.** A) Exemplary AFM images of 12HB DNA origami folded leaving out 9 staple strands in the central region using folding program 1, repaired 12HB DNA origami with incorporated missing staples after a second folding with folding program 1 for T≤50°C and an intact reference 12HB folded with complete set of staple strands, respectively. Scale bars represent 500 nm. B) Corresponding angular distribution histograms obtained by manual angle measurement of AFM images over N picked molecules. Lines indicate cumulative distributions.

Exemplary AFM images in Figure S5A revealed a large population of defective 12HB DNA origami when leaving out 9 staple strands in the central region of the nanoruler during the first DNA origami folding. The defective structures were mostly kinked and showed a decreased height in the region of single stranded scaffold. To accelerate incorporation of the 9 missing staples into the already folded defective 12HB nanorulers, we used a temperature ramp according to folding Program 1 in Table S3, but with T starting from 50°C, and a 300x excess of the 9 added staples with respect to the purified 12HB. The lower starting T was chosen to prevent melting of the already folded DNA origami. In a similar approach a scaffold strand was folded with a low number of staple strands in a first folding step with high starting temperature. In a second folding with lower starting temperatures, the set of missing staples could successfully fold the prescribed scaffold strand into the desired shape.[12] After addition of the missing staple strands, the population of defective 12HB was significantly decreased. Quantitative analysis was carried out by manual angular measurements between the two halves of picked nanorulers using ImageJ. To investigate nanorulers, whose structures were only influenced by the incomplete stapling of the scaffold strand, only those were analyzed which were immobilized as isolated monomers, while aggregates were dismissed. The obtained angular distributions in Figure S5B show a broad distribution from 0 to 180° for the defective structures. The 12HB sample after repair with the 9 missing staples exhibited an improved and narrowed angular distribution, which was shifted close to the angular distribution of an intact reference 12HB sample, which was folded with the complete set of staple strands. Defining all nanorulers with an angle under 160° as defective, resulted in a defective population of 63% after first folding with 9 staple strands left out and of only 32% after second folding with addition of the missing staples. The angular distributions indicate that the 9 missing staple strands were successfully incorporated into the defective 12HB origami and the emulated structural damage was partially repaired resulting in improved structural integrity of the nanorulers.

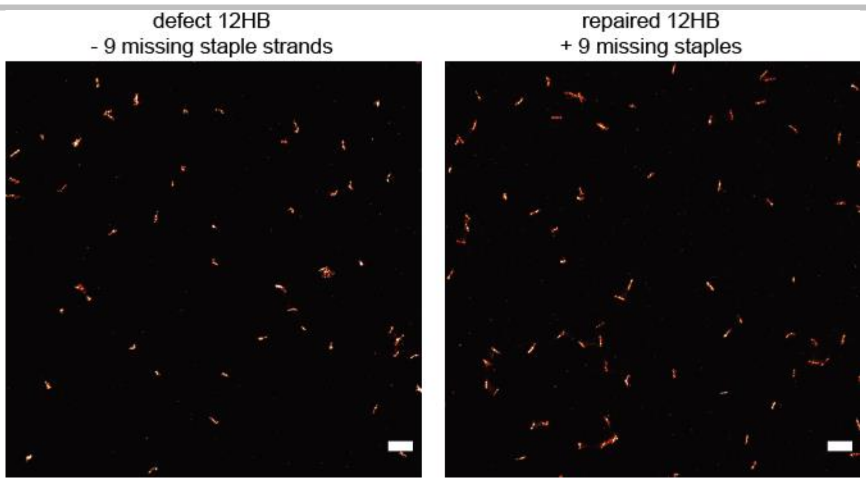


**Figure S6.** Exemplary contour DNA PAINT images. Left: defective 12HB DNA origami with 9 missing staples. Right: repaired 12HB DNA origami. Scale bars represent 500 nm.

Additionally, we investigated the defective and repaired 12HB nanorulers using DNA PAINT imaging and a dense docking site labeling along the whole length of the 12HB. By this labeling strategy, we were able to image and visualize the contour of the nanorulers with super resolution similar to AFM imaging (Figure S6). DNA PAINT images of the defective 12HB nanorulers showed a large population (72%) of collapsed or kinked 12HB and a small population of linear nanorulers. The repaired 12HB nanorulers revealed a significantly increased population of linear, intact nanorulers, while the population of visibly defective nanorulers was decreased to 38%.

It stands out, that the visibly defective 12HB nanorulers in the DNA PAINT images showed more collapsed structures than in corresponding AFM images. We ascribe this difference to the different immobilization strategies (Poly-L-Ornithine on mica in AFM, Biotin-NeutrAvidin immobilization on BSA passivated glass surface in DNA PAINT). In AFM imaging, the nanorulers were immobilized via ionic interactions with the positively charged surface and thus over the whole length of the 12HB. In DNA PAINT, the 12HB were immobilized via only 4 biotinylated staple strands, two on each site of the emulated damage, and should thus have a higher flexibility. The collapsed nanorulers could represent defective, flexible structures, which where immobilized via only one site of the emulated damage.

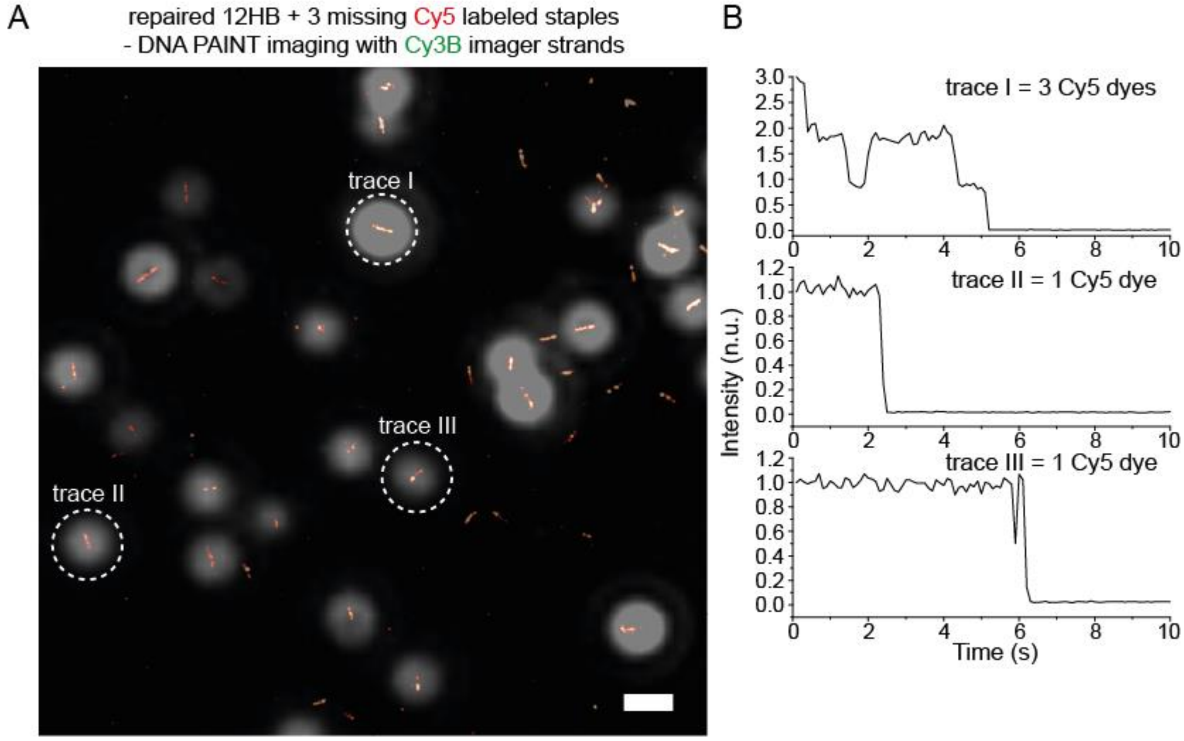


**Figure S7.** (A) Co-localized widefield DNA PAINT image of repaired 12HB, that was incubated with six unmodified missing staples and three Cy5 labelled missing staples in second folding. Contour DNA PAINT imaging was carried out with an 8 nt Cy3B imager in green. Widefield signals from Cy5 labelled staple strands (grey PSF) show successful incorporation for a high fraction of 12HB DNA origami. (B) Exemplary extracted single-nanoruler transients reveal bleaching steps for 1 to 3 incorporated Cy5 dyes per repaired DNA nanoruler. Scale bars represent 500 nm.

Besides the conducted AFM and DNA PAINT characterization of the defective and repaired 12HB nanorulers, we carried out a colocalized widefield DNA PAINT experiment to prove incorporation of the 9 missing staple strands (Figure S7A). Therefore, we exchanged 3 of the 9 missing staple strands with Cy5 labeled staple strands. After repair with the partially Cy5 labeled set of staple strands, we acquired diffraction limited Cy5 signals in red (640 nm) and corresponding DNA PAINT images of same regions with a

Cy3B labeled 8 nt DNA PAINT imager strand. The colocalized image of diffraction limited Cy5 signals and DNA PAINT information revealed a successful incorporation of Cy5 labeled staple strands into most of the 12HB nanorulers measured with DNA PAINT, considering that only one third of the nine missing staples were labeled with Cy5. While the DNA PAINT images revealed the structure of the nanorulers, the diffraction limited single spots could be further investigated by extracting their corresponding time transients until bleaching. While most of the transients exhibited one incorporated Cy5 labeled staple, a minority also exhibited two or even three incorporated Cy5 labeled staple strand (see exemplary transients in Figure S7B). The colocalized images indicate significant incorporation of at least a subset of the nine missing staples, so that the above-mentioned changes in AFM and DNA PAINT experiments during repair indeed can be assigned to the incorporation of missing staple strands.

The results from AFM imaging and DNA PAINT experiments showed consistent results. The incomplete set of staple strands in the first folding resulted in a defective DNA origami population which could partially be repaired and improved in its structural integrity. Comparison with an intact reference structure showed that the repair could not remove the emulated damage in the whole nanoruler population but lead to a significant improvement of the structural distribution.

### 2.3. Staple strand exchange from dual spot to triple spot in NRO nanoruler

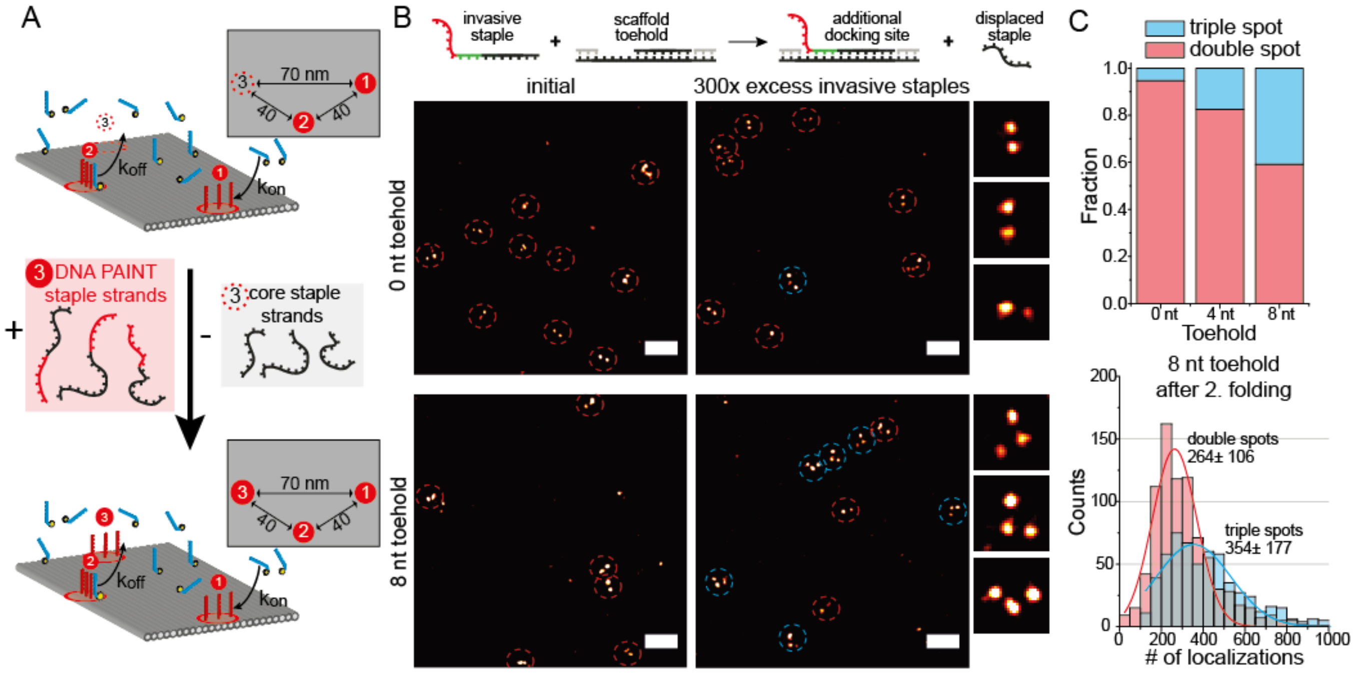


**Figure S8.** (A) Scheme of the reconfigurable NRO nanoruler. By addition of DNA PAINT staple strands in a second folding, a double spot nanoruler (40 nm) can be transformed into a triple spot nanoruler. (B) Scheme of accelerated exchange of staple strands by toehold on the scaffold strand. Exemplary DNA PAINT images of 0 nt and 8 nt toehold nanorulers before and after addition of triple spot staple strands in a second folding reveal double spots (red circles) before and partially triple spots (blue circles) after incubation. Exemplary magnifications show double spot nanorulers (0 nt toehold) and triple spot nanorulers (8 nt toehold) after second folding. Scale bars represent 200 nm (C) Relative fractions of double and triple spot nanorulers for 0, 4 and 8 nt toehold samples after second folding revealing increasing triple spot population with increasing toehold length. Exemplary extracted localizations from DNA PAINT experiments for the 8 nt toehold sample after second folding indicate a 34% increase of docking sites by incorporation of invasive staple strands.

The previous experiment with the kinked 12HB nanoruler shows that DNA strands can be incorporated into existing DNA origami nanostructures but it does not prove a self-healing mechanism as it is conceivable that staple strands would also constantly exchange in intact DNA origami structures. To this end, we designed a rectangular DNA origami with two spots (40 nm distance) consisting of docking strands for DNA PAINT measurements (Figure S8). We then added staple strands with DNA PAINT docking strands extensions that would form a third spot on the DNA origami when incorporated. To increase the exchange kinetics, we incubated the double spot NROs with a 300x excess of the invasive docking strands in solution using the temperature range of the NRO folding program but starting at T=50°C, *i.e.* below the denaturing temperature (Table S4). Interestingly, only a vanishingly small number of triple-spot DNA origamis was observed indicating that staple exchange was kinetically blocked. If, however, the DNA origami was previously synthesized with shorter staple strands in the region of the third mark so that a toehold of 4 or 8 nucleotides was formed in the scaffold, the extended staple strands could invade and replace the existing staple strands more efficiently (see scheme and images in Figure S8). After incubation with the extended staple strands, between 20 and 40% of DNA origamis exhibited the triple mark pattern as displayed in Figure S8B and Figure S8C confirming the notion that a toehold is required for efficient strand displacement reactions also within an intact DNA origami.[12-14] Successful incorporation of the staple strands forming the third labeling spot could also be probed by looking at the number of localizations per DNA origami nanostructure. The number of docking sites is increased during incorporation of the third labeling spot, which should also lead to an increase of localizations per DNA PAINT experiment. For the 8 nt toehold sample, the picked triple-spot nanorulers revealed an average number of localizations of around 354, compared to only 264 localizations for the picked double spots nanorulers within the same sample. For quantitative exchange and incorporation 150% of the localizations of the double spot nanorulers are expected for the triple spot population (since 6 docking sites are increased to 9 docking sites theoretically). The observed increase of localizations to 134% indicates, that on average around two out of the three docking sites are efficiently incorporated under the used conditions.

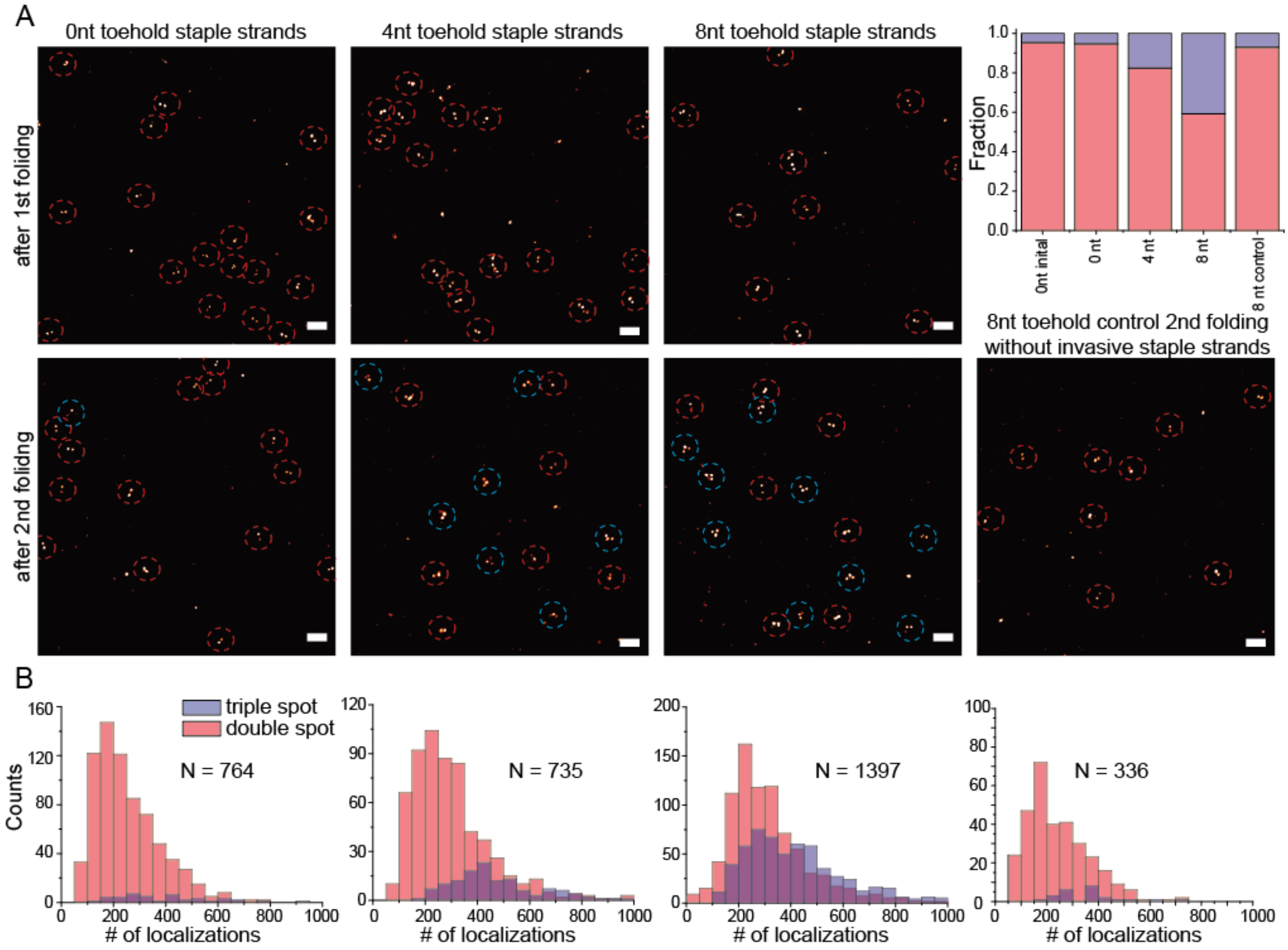


**Figure S9**. (A) Exemplary DNA PAINT images of 0 nt, 4 nt and 8 nt toehold nanorulers before and after addition of triple-spot staple strands in a second folding reveal double-spots (red circles) before and partially triple-spots after (blue circles). Exemplary DNA PAINT image of 8 nt toehold sample after second folding program without addition of triple spot staple strands reveal only double-spot nanorulers. Scale bars represent 200 nm. Relative fractions of double and triple-spot nanorulers for 0 to 8 nt toehold samples and reference after second folding reveal increasing triple-spot populations with increasing toehold length but no significant triple-spot population without addition of invasive staple strands (B). Extracted localizations from DNA PAINT experiments for the 0 to 8 nt toehold samples and 8 nt toehold reference after second folding indicate increase numbers of docking sites by incorporation of invasive staple strands.

A further comparison of exemplary DNA PAINT images of 0 to 8 nt toehold samples and corresponding extracted pick numbers and number of localizations are given in Figure S9. During manual picking of dual and triple-spot nanorulers in the obtained DNA PAINT images, we observed a small fraction of pseudo triple-spots in the samples even before adding the third labeling spot staple strands. Picking of the 0 nt toehold sample before addition of the invasive staple strands revealed e.g. a small fraction of around 5% of such pseudo triple-spots. We ascribe this population to NRO dimers, which accidently form triple spots by superposition of two individual double spot nanorulers. While the addition of the invasive staple strands led to no significant increase of the triple spot fraction for the 0 nt toehold sample, a significant higher fraction of around 20% could be found for 4 nt toehold and around 40% for the 8 nt toehold sample. According to toehold mediated strand displacement kinetics, the exchange is accelerated for increasing toehold lengths. To probe, if the increasing number of triple spots NROs was primarily due to the formation of more pseudo triple spots by unspecific dimerization of the DNA origami during the second folding, we made a reference sample for the second folding. An 8 nt toehold double spot NRO sample was treated with the same buffer and temperature ramp from NRO folding (starting at T= 50°C) but without addition of the invasive staple strands forming the third labelling spot. DNA PAINT images revealed no significant increase of the pseudo triple-spot population (Figure S9), indicating that the picked triple-spot populations after addition of the invasive staple strands can be attributed to successful incorporation into the existing DNA origami nanostructures.

### 2.4. Self-Healing of 12HB nanorulers in degrading conditions

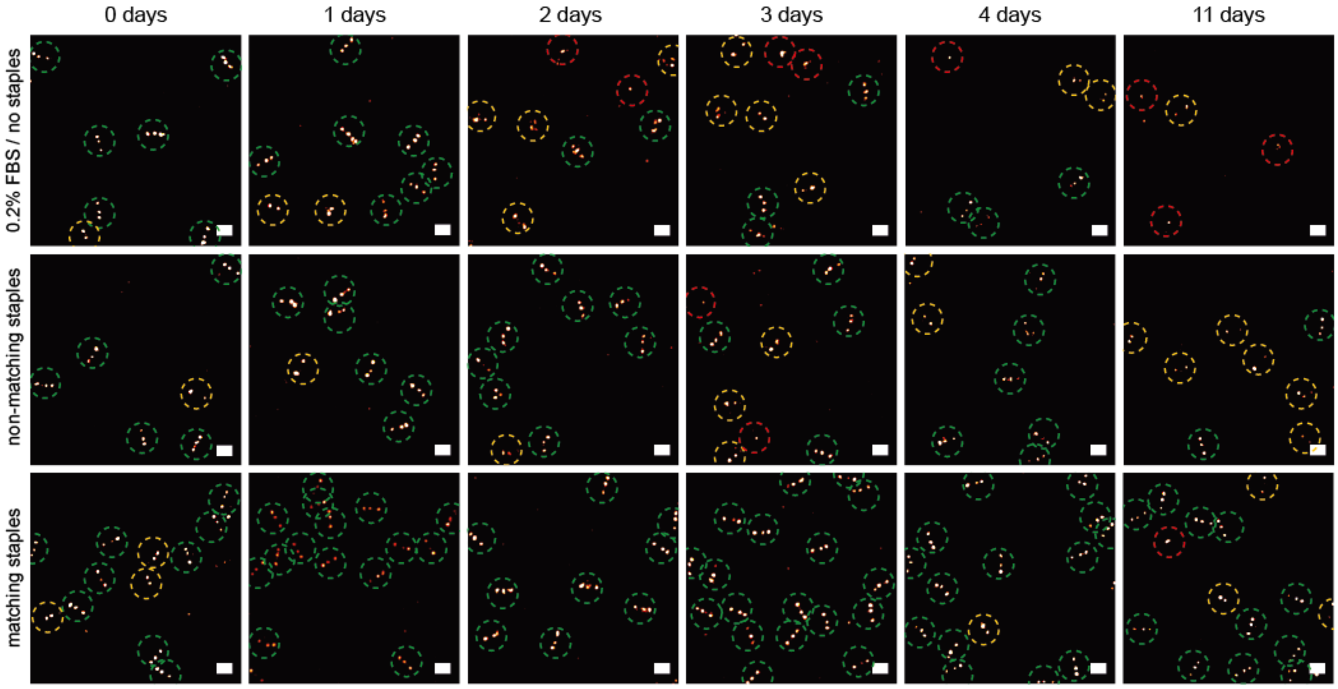


**Figure S10**. Exemplary DNA PAINT images of 12HB triple-spot nanorulers in 0.2% FBS solution (top), with added non-matching DNA strands (middle) and matching staple strands of the nanoruler (bottom) after immobilization, and 1 to 11 days of incubation, respectively. Triple-spot nanorulers are highlighted by green, double-spot nanorulers by yellow and single-spot nanorulers by red circles. Scale bars represent 200 nm.

Exemplary DNA PAINT images of immobilized triple-spot nanorulers (107 and 70 nm inter-mark distance) in the three different incubation conditions are given in Figure S10A. While the sample incubated in 0.2% FBS solution showed rapid degradation, *i.e.* loss of labeling spots and decreasing surface density, the addition of non-matching oligonucleotides led to a visible stabilization over the investigated time of 11 days. The addition of a set of matching unmodified staple strands stabilized the nanorulers significantly in the degrading environment so that even after 11 days, a majority of the nanorulers still exhibited a double or triple spot. For a more quantitative analysis of the induced structural damage, we extracted the number of localizations and off-times per picked nanoruler. For a degradation of the DNA PAINT nanorulers, a decrease of docking sites over time is expected. Lowered numbers of docking sites lead to lower numbers of binding events and thus also of localization events of bound imager dyes within a given time. Simultaneously, a decreasing number of docking sites increases the time between to binding event, i.e. the off-time. To decrease the influence of systematic fluctuations of the used widefield setup on quantitative analysis of the occurring damage of the DNA PAINT nanorulers, we conducted the degradations study for each incubation condition three times. Averaging over all three sets of experiments resulted in the curves given in Figure 3F-G in the main text. The extracted localizations and off-times correspond to the qualitative results from DNA PAINT images.

Combining the results from the previous NRO studies that only damaged staples with incomplete stapling of the scaffold strand are exchanged effectively, with the shown stabilization of 12HB nanorulers by presence of intact staple strands, we conclude that the given example fulfills our definition of self-healing. The applied self/repairing system improved the structural integrity of the nanorulers under wear significantly and could be applied to realize long lasting super resolution nanorulers.

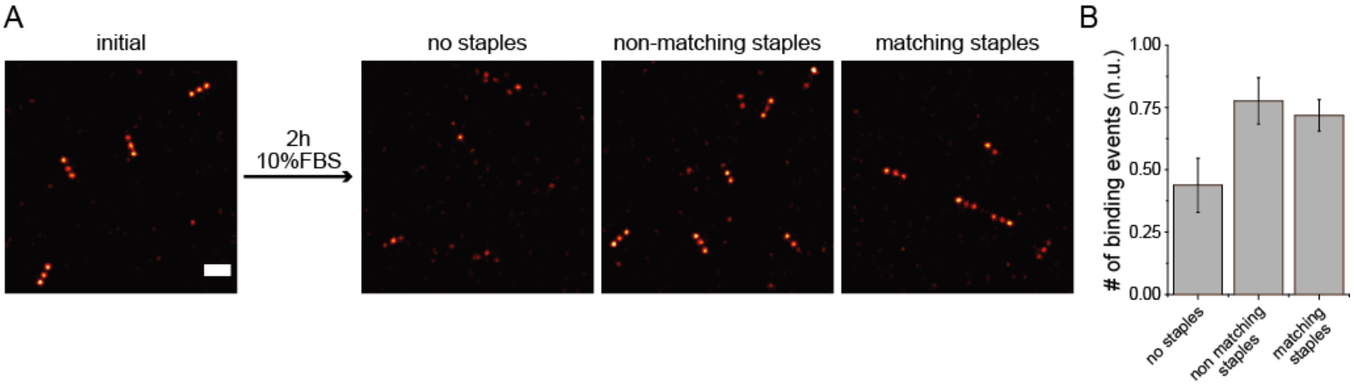


**Figure S11.** (A) Exemplary DNA PAINT images of 12HB triple-spot nanorulers after 2 h incubation in 10% FBS solution without any added DNA strands (left), with added non-matching DNA strands (middle), and with added specific DNA staple strands (right), respectively. Scale bars represent 200 nm. (B) Corresponding extracted, averaged and normalized number of DNA PAINT binding events per nanoruler after 2h incubation in 10% FBS solution with and without added DNA staple strands. Number of binding events were normalized to an intact reference sample. Each column represents the average of three different measured samples, error bars represent standard deviation.

To investigate potential self-healing at higher damage rates, we carried out DNA PAINT studies of DNA PAINT nanorulers incubated in 10% FBS solution for 2 hours. To achieve DNA PAINT images faster, we used a 10 nM solution of the 6 nt ATTO655 imager strand in Table S5 in an 1x PBS buffer containing 12.5 mM $MgCl_2$. With these parameters and a frame time of 25 ms, we were able to achieve super-resolution images of immobilized 12HB nanorulers within 10 minutes. Exemplary DNA PAINT images of an intact reference and after 2 h incubation in 10% FBS solution are given in Figure S11A. Incubation with 10% FBS led to fast degradation of immobilized 12HB nanorulers, while addition of intact non-matching or matching DNA staple strands led to significant stabilization so that a majority of the nanorulers still contained three spot pattern characteristic to an intact structure. To have a more quantitative comparison, we extracted the number of binding events per DNA origami nanoruler after incubation, averaged and normalized to the number of binding events of an intact reference structure (Figure S11B). The commercial setup used for these experiments (ONI nanoimager S) is a closed system. During measurements with continuous excitation such as DNA PAINT imaging, the heat input by the laser illumination leads to an uncontrolled heating of the whole microscope body. On the other hand, the number of localisations during a DNA PAINT experiment is the product of binding times (in units of single frame time) and binding events. Since the binding times are highly dependent on the temperature thus are the number of localisations. The binding events, on the contrary, are more stable for small temperature variations, since they depend mostly on the concentration of the imager strand in solution. To have a temperature-independent comparison, we extracted the number of binding events per picked nanorulers and compared the different incubation conditions (Figure S11B). Two hours incubation with 10% FBS led to a decrease of binding events per nanoruler to under 50%. The addition of a set of non-matching staple strands and the set of matching staple strands at a total concentration of 5 µM lead to a significant stabilization resulting in a number of binding events of around 75%. The comparable results for non-matching and matching DNA staples indicated that the sacrificial degradation of the added DNA is the effective stabilization mechanism at 10% FBS. However, no self-healing effect could be observed. The high concentration of nucleases in the 10% FBS solution induce fast degradation of the DNA origami nanostructures, which cannot be compensated by self-healing as shown for 0.2% FBS incubation over days. Self-healing of DNA origami is thus limited to lower damaging rates, while the sacrificial degradation of added DNA can stabilize the nanorulers effectively even at fast degradation rates.

To further examine the stabilization of the DNA origami nanoruler in 10% FBS by sacrificial degradation of added DNA staples, we added different concentrations of DNA staples (total concentration of 5 µM, 500 nM and 50 nM) to the 10% FBS incubation solution. Exemplary distributions of number of binding events extracted from picked DNA PAINT nanorulers after two hours of incubation represented as box plots are given in Figure S12. For any of the added concentrations of DNA staples, the non-matching and matching staples resulted in comparable binding events, showing that no self-healing was stabilizing the nanorulers. Addition of DNA staple strands with a total concentration of 50 nM led to comparable damage than no addition of DNA, while the addition of staples with 500 nM led to a significant stabilization but lower binding event per nanoruler than addition of 5 µM of DNA staples. The results indicate that even with 500 nM solution of added DNA, DNA origami structures could be stabilized significantly in highly degrading conditions such as in 10% FBS via sacrificial degradation.

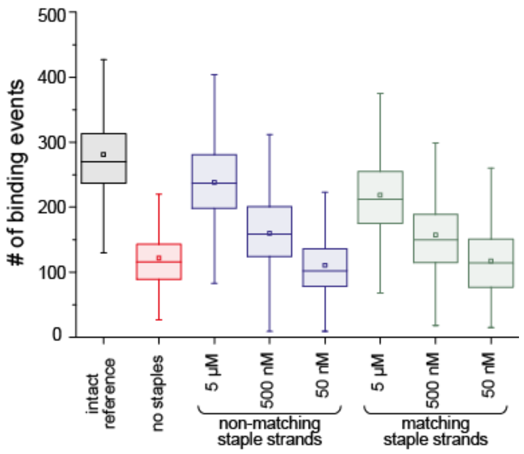


**Figure S12.** Exemplary box plots of number of binding events per single nanoruler. Intact reference sample highlighted in black, sample incubated in 10% FBS in red, samples incubated with 10% FBS and non-matching DNA staple strands in blue and samples incubated with 10% FBS and matching staple strands in green, respectively. Squares indicate the average, central lines the median, box lines the 25% and 75% quantiles and whiskers the 1.5 times interquartile range, respectively.

### 2.5. Self-regeneration and self-healing of an enzymatically cleavable label on 6HB brightness ruler

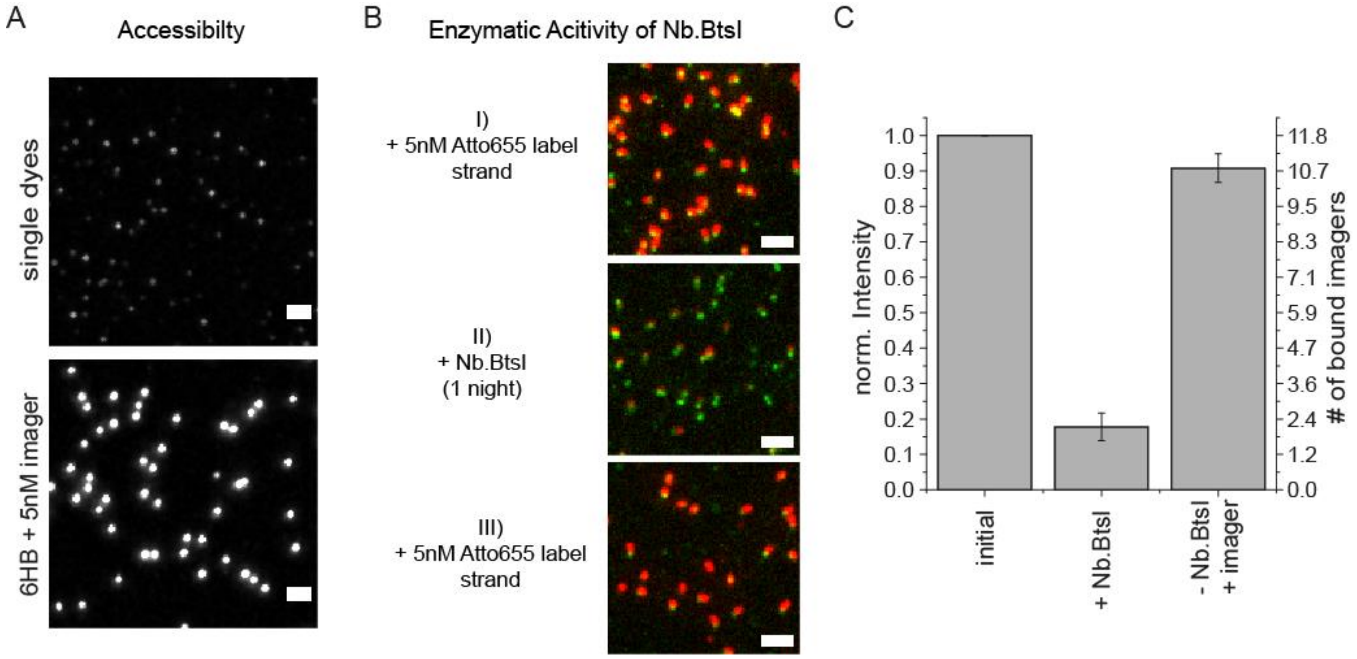


**Figure S13**. (A) Exemplary TIRF images of immobilized single ATTO655 labeled oligonucleotides (top) and 6HB brightness ruler labeled with 20 nt ATTO655 label (bottom) for accessibility studies. Scale bars represent 2 µm. (B) Two-color colocalized TIRF images of internally labeled (ATTO532) 6HB brightness ruler externally labeled with ATTO655 labels, after incubation with *Nb.BtsI* and after relabeling with intact ATTO655 labels. Scale bars represent 2 µm. (C) Extracted brightness values initially, after addition of *Nb.BtsI* and after relabeling with intact imager strands. Number of bound imager strands could be estimated from intensity comparison of single dyes and 6HB brightness rulers in (A). Data represent averaged normalized brightness values of three different experiments, errors represent standard deviation.

To estimate the external labeling efficiency of the brightness ruler, we compared the intensity of single immobilized ATTO655 labeled oligonucleotides and the 6HB brightness ruler (Figure S13A). The designed brightness ruler exhibited a labeling number of 11.8 (59%). Next we probed the enzymatic activity of the used *Nb.BtsI* by comparing the brightness values before and after the incubation with the restriction enzyme. Therefore, we internally labeled the 6HB brightness rulers with ATTO532 (labeled staple strands in Table S13) to localize brightness rulers with complete label cleavage (B). Cleavage by *Nb.BtsI* overnight led to average brightness loss to below 20%. After washing and addition of imager strands brightness recovered back to over 90% of initial brightness, indicating that the majority of the docking sites were not affected by the enzyme (Figure S13C).

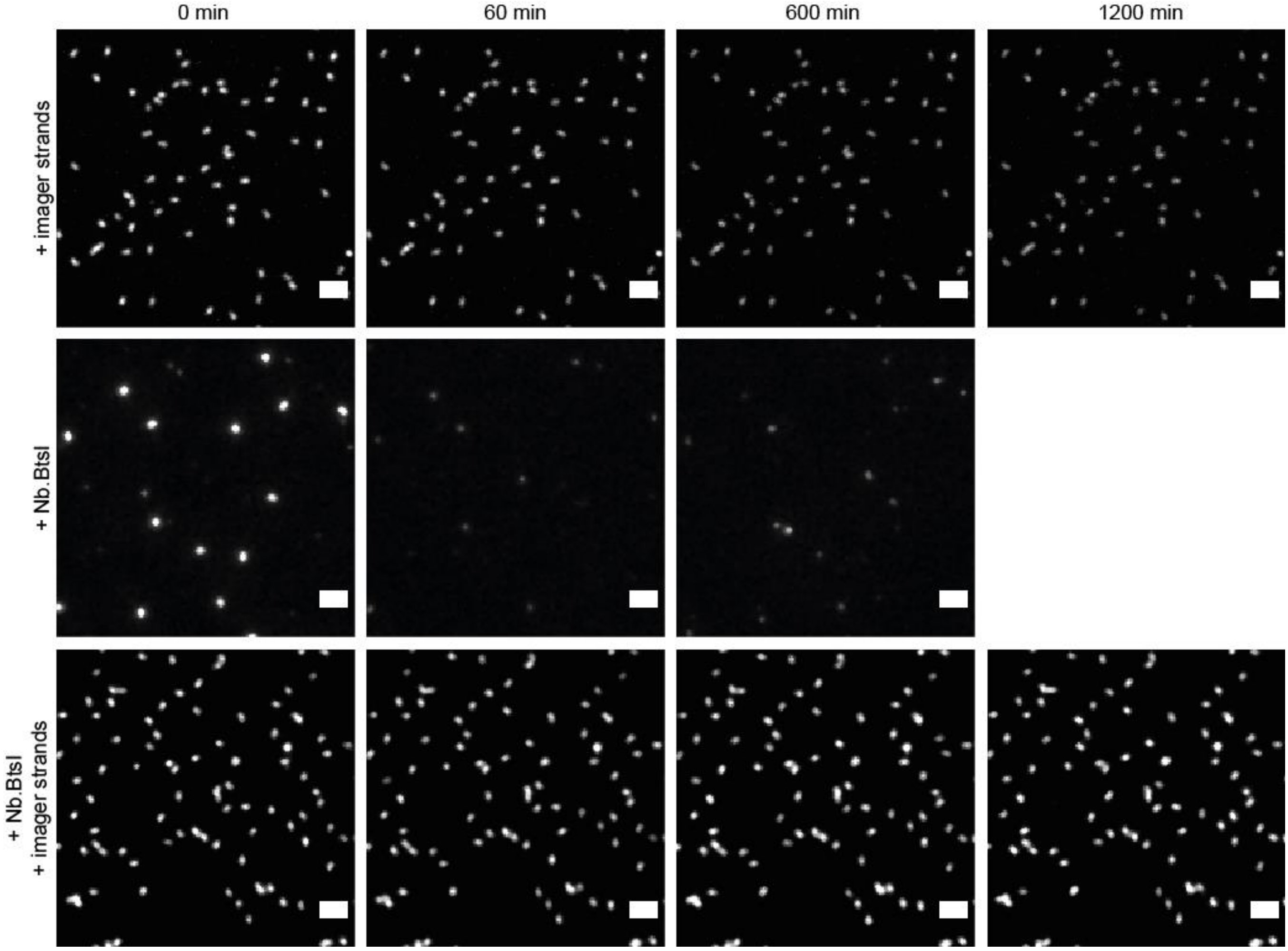


**Figure S14.** Exemplary TIRF images over time for three different labeling conditions under time lapse imaging (75 W/cm$^2$ 100 ms every 10 min). Top: Photobleaching of permanent label in 5nM imager solution over time. Middle: Enzymatic damage induced by *Nb.BtsI* leads to rapid loss brightness signal. Bottom: Simultaneous addition of *Nb.BtsI* and 5 nM imager strand solution leads to stable brightness signal over time. Transient binding is achieved by a steady-state between enzymatic cleavage, fast dissociation and association. Scale bars represent 2 µm.

To investigate and emphasize the concepts of self-regeneration and self-healing, we compared three different incubation conditions via time lapse TIRF imaging (3 mW at 640 nm every 10 min). Figure S14 shows exemplary TIRF images of immobilized 6HB brightness rulers over time incubated with a 5 nM solution of imager strands (top), with a solution of *Nb.BtsI* (middle) and a solution containing 5 nM imager strands and *Nb.BtsI* (bottom). While the addition of the restriction enzyme alone cleaved the labels and led to a rapid loss of the brightness signal after 1 hour, the incubation in a 5 nM imager strand solution could not recover the slow photobleaching during time-lapse imaging. Simultaneous addition of the restriction enzyme *Nb.BtsI* and of imager strands led to a dynamic stable brightness label showing no photobleaching effects under the used time-lapse imaging conditions even after 20 h.

In summary, the applied system recovers the building units of the brightness function, *i.e.* the imager strands, unspecifically with respect to photobleaching, as bleached and photoactive dyes are exchanged. With respect to the enzymatic damage by the restriction enzyme, only damaged units dissociate fast enough as two 10 nt fragments which can be replaced by an intact imager strand from solution. The self-repair in this example shows that self-regeneration and self-healing can occur simultaneously within one system when different sources of damage are present.

## 3. Appendix

**Table S15**. Unmodified staple strands of 12HB DNA origami. Sequences are denoted from 5'- to 3'-end. The numbers for the 5'- end 3'-end of the staples represent the helix number in the corresponding caDNAno file. Number in brackets represent the starting and ending position of the staple in the corresponding helix.

| Unmodified staple strands | 5'-end | 3'-end |
|---|---|---|
| AAAGGGCGCTGGCAAGTATTGGC | 11[681] | 10[668] |
| GCGCCTGAATGCCAACGGCCCAGCCTCCCGCGTGCCTGTTCTTCTTTTT | 7[42] | 8[25] |
| TTGACGGGGAAAGCTTCACCAGAAATGGCATCACT | 11[651] | 6[658] |
| CATTCAACCCAAAATGTAGAACCCTCATGAATTAGTACAACC | 9[147] | 5[160] |
| TCAGAGGTGTGTCGGCCAGAATGAGTGCACTCTGTGGT | 4[60] | 7[62] |
| GGCATAAGCGTCTTCGAGGAAACGCA | 8[466] | 9[482] |
| TACATAAATTCTGGGCACTAACAACT | 8[634] | 9[650] |
| CAATCCAAAATACTGAACAGTAG | 3[457] | 10[458] |
| CATAGTTAATTTGTAAATGTCGC | 3[541] | 10[542] |
| GAACAAGAGTCCACCAATTTTTTAGTTGTCGTAGG | 11[483] | 6[490] |
| TTGAAGCCCTTTTTAAGAAAAGT | 7[441] | 7[463] |
| AAGCACAGAGCCTAATTATTGTTAGCGATTAAGACTCCTT | 7[464] | 8[448] |
| GATGTTTTTCTTTTCACCA | 10[289] | 11[302] |
| GGTCACGCCAGCACAGGAGTTAG | 3[373] | 10[374] |
| TGAACAGCTTGATACCGATAGTT | 8[363] | 8[341] |
| AAAATTCCATTCAGGCTTTTGCAAAA | 8[256] | 9[272] |
| TCCCATCCTAATGAGAATAACAT | 0[496] | 0[474] |
| ATCAGCGGGGTCAGCTTTCAGAG | 3[56] | 3[78] |
| TTCGCTATTCGCAAGACAAAGTTAATTTCATCTTC | 5[539] | 4[546] |
| TTGAGAATATCTTTCCTTATCACTCATCGAGAACA | 5[497] | 4[504] |
| GGGCGTGAAATATTAGCGCCATTCGC | 8[130] | 9[146] |
| GGCGCCCCGCCGAATCCTGAGAAGTGAGGCCGATTAAAGG | 3[667] | 0[665] |
| TTTTTTGTTTAATAAAGTAATTC | 3[476] | 3[498] |
| AAATCAGCCAGTAATAACACTATTTTTGAAGCCTTAAATC | 7[506] | 8[490] |
| AGCACTAAATCGGATCGTATTTAGACTTATATCTG | 11[609] | 6[616] |
| GGTGCCGTCGAGAGGGTTGATAT | 8[405] | 8[383] |
| GTCAGAATCAGGCAGGATTCGCG | 3[205] | 10[206] |
| TTTTTTATAACGTGCTTTCCTCTTTATAACAGTACTAT | 2[698] | 3[678] |
| AGACGGGAGAATTGACGGAAATT | 0[454] | 0[432] |
| TAAGCCAGAGAGCCAGAAGGAAACTCGATAGCCGAACAAA | 4[480] | 7[482] |
| CGCCTGACGGTAGAAAGATTCTAATGCAGATACAT | 5[245] | 4[252] |
| CAGTCTTGATTTTAAGAACTCAACGTTGCGTAT | 0[263] | 11[272] |
| CATAGAATTTGCGGTTTGAAAGAGGA | 8[298] | 9[314] |
| GCGCAGCGACCAGCGATTATATATCATCGCCTGAT | 5[287] | 4[294] |
| TTTTTAAAAACGCTCATGGAAATA | 8[698] | 8[679] |
| AATCAGTTAAAACGTGGGAGAAA | 3[121] | 10[122] |
| AGACAACCTGAACAGTATTCGAC | 3[625] | 10[626] |
| TTTGCAACCAGCTTACGGCGGTGGTGAGGTTTCAGTTGAGGATCCTTTTT | 3[25] | 10[29] |
| TGCAACACTATCATAACCCTCGT | 7[231] | 7[253] |
| AACGAACCTCCCGACTTGCGGGA | 8[531] | 8[509] |
| CCGAACGGTGTACAGACCAGGCG | 8[321] | 8[299] |
| ATTCAAGGGGAAGGTAAATGTGGCAAATAAATC | 0[431] | 11[440] |
| GTCACCAGTACAAGGTTGAGGCA | 3[350] | 3[372] |

| | | |
|---|---|---|
| TAAATCGGTTGGTGCACATCAAAAATAA | 6[153] | 2[140] |
| AGACGGCGAACGTGGCGAG | 10[667] | 11[680] |
| CCCTTCATATAAAAGAACGTAGAGCCTTAAAGGTGAATTA | 11[429] | 0[413] |
| AACTTTAATCATGGGTAGCAACG | 3[266] | 3[288] |
| ACCATCACCCAAATAAACAGTTCATTTGATTCGCC | 11[567] | 6[574] |
| TGCCTAATGAGTGAGAAAAGCTCATATGTAGCTGA | 11[147] | 6[154] |
| TTTTTTGGTAATGGGTAACCATCCCACTTTTT | 1[21] | 2[25] |
| GGAGCAGCCACCACCCTTCGCATAACGACAATGACAACAA | 7[338] | 8[322] |
| AAAAGTGTCAGCAACAATTGCAGGCGCT | 6[69] | 2[56] |
| GGTTTGCGCATTTTAACGCGAGGCGT | 8[508] | 9[524] |
| AAAAGAATAGCCCGATACATACGCAGTAAGCTATC | 11[441] | 6[448] |
| TTTCACGAGAATGACCATTTTCATTTGGTCAATAACCTGT | 7[212] | 8[196] |
| TCGGTCATACCGGGGGTTTCTGC | 8[69] | 8[47] |
| CCTCCGAAATCGGCAAAAT | 10[415] | 11[428] |
| TTCCATTGACCCAAAGAGGCTTTGAGGA | 2[307] | 3[307] |
| ACGCGTCGGCTGTAAGACGACGACAATA | 2[517] | 3[517] |
| GTCCGTCCTGCAAGATCGTCGGATTCTCTTCGCATTGGACGA | 9[105] | 5[118] |
| GTCAGTCGTTTAACGAGATGGCAATTCA | 6[615] | 2[602] |
| GAGCTTAAGAGGTCCCAATTCTGCAATTCCATATAACAGT | 4[228] | 7[230] |
| GCAGCACTTTGCTCTGAGCCGGGTCACTGTTGCCCTGCGGCTTTTT | 10[48] | 0[21] |
| TACCTGGTTTGCCCCAGCA | 10[373] | 11[386] |
| AATGCTGTAGCTGAGAAAGGCCG | 4[209] | 4[187] |
| CTATATTAAAGAACGTGGA | 10[499] | 11[512] |
| CGGTAGTACTCAATCCGCTGCTGGTCATGGTC | 0[53] | 11[62] |
| CTTGAAAACACCCTAACGGCATA | 3[247] | 10[248] |
| AAGTAAGAGCCGCCAGTACCAGGCGG | 8[382] | 9[398] |
| AAAAGATAGGGTTGAGTGT | 10[457] | 11[470] |
| TTCGCCATAAACTCTGGAGGTGTCCAGC | 2[55] | 3[55] |
| AGGGCGAAAAACCGATTTAACGTAGGGCAAATACC | 11[525] | 6[532] |
| CCCACATGTGAGTGAATAACTGATGCTTTTAACCTCCGGC | 11[555] | 0[539] |
| TTTTTAGGAGCGGGCGCTAGGAAGGGAAGAAAGCGAATTTTT | 10[702] | 11[702] |
| TGCCATACATAAAGATTAACTGAACACCAACAGCCGGAATAG | 9[441] | 5[454] |
| TTTTTCCGGTGCAGCACCGATCCCTTACACTTGCC | 5[29] | 4[52] |
| ACAGCTGATTGCCCGTCGCTGCGCCCACACGTTGA | 11[315] | 6[322] |
| ATTAAAATAAGTGCGACGATTGGCCTTG | 2[391] | 3[391] |
| AAAACGAAAGAGGCTCATTATAC | 0[286] | 0[264] |
| TGTCCAAGTACCAGAAACCCCAG | 3[499] | 10[500] |
| TTACCAATAAGGCTTGCAGTGCGGAAGTTTAGACTGGATA | 7[254] | 8[238] |
| TTAGTGTGAATCCCTCTAATAAAACGAAAGAACGATGAATTA | 9[231] | 5[244] |
| ATCAGAGCCTTTAACGGGGTCTTAATGCCCCCTGC | 5[371] | 4[378] |
| TTACCTCTTAGCAAATTTCAACCGATTG | 6[447] | 2[434] |
| AAAACGGAATACCCAAAAGAACT | 8[489] | 8[467] |
| GTCCACGCGCCACCTCACCGTTGAAACA | 11[364] | 6[364] |
| TTTTTATCCAGCGCAGTGTCACTGC | 7[21] | 7[41] |
| GATGAATAAATCCTGTAGGTGAGGCGGTAGCGTAAGTCCTCA | 9[609] | 5[622] |
| GCTAAATCGGTTTGACTATTATA | 3[182] | 3[204] |
| CAGCTTTGAATACCAAGTTACAA | 7[567] | 7[589] |
| GGTTGCTTTGACGAGCACGTTTTT | 3[679] | 3[698] |
| CATGCCAGTGAGCGCTAATATCCAATAATAAGAGC | 5[455] | 4[462] |

| | | |
|---|---|---|
| TATGCATTACAGAGGATGGTTTAATTTC | 2[265] | 3[265] |
| ACTGCCCGCTTTCCTGAAAAGCTATATTTTAAATA | 11[189] | 6[196] |
| TGATTTAGAAAACTCAAGAGTCAATAGT | 6[573] | 2[560] |
| TGGGCGCCAGGGTGATTCATTAGAGTAACCTGCTC | 11[273] | 6[280] |
| TGCAACTCAAAAGGCCGTACCAAAAACA | 6[195] | 2[182] |
| AAATAGGTAATTTACAAATAAGAAACGA | 2[475] | 3[475] |
| TGTTCCAACGCTAACGAACAAGTCAGCAGGGAAGCGCATT | 11[471] | 0[455] |
| GTGCCTGCTTTAAACAGGGAGAGAGTTTCAAAGCGAACCA | 11[219] | 0[203] |
| GTTTGATGGTGGTTCAGAACCCCGCCTCACAGAAT | 11[399] | 6[406] |
| TCACCGTCACCGGCGCAGTCTCT | 0[412] | 0[390] |
| AGACGTCGTCACCCTCAGATCTTGACGCTGGCTGACCTTC | 7[296] | 8[280] |
| TTTAGCAAACGCCACAATATAACTATATTCCCTTATAAATGG | 9[525] | 5[538] |
| AGCGTATCATTCCACAGACCCGCCACAGTTGCAGCAAGCG | 0[347] | 11[363] |
| GTATGTGAAATTGTTATCC | 10[79] | 11[92] |
| CCGAACTTTAATAAAAGCAAAGCGGATT | 2[223] | 3[223] |
| GTGAGTTAAAGGCCGCTGACACTCATGAAGGCACCAACCT | 11[303] | 0[287] |
| GCGCCCGCACCCTCTCGAGGTGAATT | 8[340] | 9[356] |
| ACAGTTTTTCAGATTTCAATTACCGTCGCAGAGGCGAATT | 4[606] | 7[608] |
| TTTAGAACGCGAATTACTAGAAAACTATAAACACCGGAAT | 4[564] | 7[566] |
| TGACCTAAATTTTTAAACCAAGT | 4[545] | 4[523] |
| TAAAGAGGCAAAATATTTTATAA | 3[163] | 10[164] |
| GTTTACCGCGCCCAATAGCAAGC | 7[483] | 7[505] |
| TACCGGGATAGCAATGAATATAT | 3[331] | 10[332] |
| AAATTGTGTCGAGAATACCACAT | 4[293] | 4[271] |
| AAATGCGTTATACAAATTCTTAC | 8[573] | 8[551] |
| CAGATATAGGCTTGAACAGACGTTAGTAAAGCCCAAAAATTT | 9[315] | 5[328] |
| TAAGATCTGTAAATCGTTGTTAATTGTAAAGCCAACGCTC | 7[548] | 8[532] |
| CATTCTATCAGGGCGATGG | 10[541] | 11[554] |
| CTCCAATTTAGGCAGAGACAATCAATCAAGAAAAATAATA | 11[513] | 0[497] |
| GAGACAAAGATTATCAGGTCATTGACGAGAGATCTACAAA | 4[186] | 7[188] |
| AGGGACAAAATCTTCCAGCGCCAAAGAC | 2[433] | 3[433] |
| AAAATTTTTTAAAATGAGCAAAAGAA | 8[592] | 9[608] |
| CATCGGGAGAAATTCAAATATAT | 4[587] | 4[565] |
| ATCATTTACATAAAAGTATCAAAATTATAAGAAACTTCAATA | 9[567] | 5[580] |
| GCTACGACAGCAACTAAAAACCG | 3[289] | 10[290] |
| TTAGGTTGGGTTATAGATAAGTC | 0[538] | 0[516] |
| TATTGCCTTTAGCGTCAGACTGT | 7[399] | 7[421] |
| TTTTTCCGGGTACCGAGCTCGAATTCGTAATCTGGTCA | 11[29] | 10[49] |
| CTAAAGACTTTTAGGAACCCATG | 3[308] | 3[330] |
| GTGGAACGACGGGCTCTCAACTT | 3[79] | 10[80] |
| TCAGGTGAAATTTCTACGGAAACAATCG | 6[111] | 2[98] |
| AAGACGCTGAGACCAGAAGGAGC | 3[560] | 3[582] |
| AGCAGTCGGGAAACCTGTC | 10[205] | 11[218] |
| AACAACATGTTCATCCTTGAAAA | 3[518] | 3[540] |
| ATAATGAATCCTGAGATTACGAGCATGTGACAAAAACTTATT | 9[483] | 5[496] |
| GAGGTAACGTTATTAATTTTAAAACAAATAATGGAAGGGT | 11[597] | 0[581] |
| ACCGCATTCCAACGGTATTCTAAGCGAGATATAGAAGGCT | 4[522] | 7[524] |
| CAGCATCAACCGCACGGCGGGCCGTT | 8[46] | 9[62] |
| GCTCAAGTTGGGTAACGGGCGGAAAAATTTGTGAGAGATA | 11[93] | 0[77] |

| | | |
|---|---|---|
| GGAATCGGAACATTGCACGTTAA | 3[583] | 10[584] |
| ATAAGAAGCCACCCAAACTTGAGCCATTATCAATACATCAGT | 9[399] | 5[412] |
| GGCGACACCACCCTCAGGTTGTACTGTACCGTTCCAGTAA | 11[387] | 0[371] |
| CATGTCAGAGATTTGATGTGAATTACCT | 6[279] | 2[266] |
| AATAGCTGTCACACGCAACGGTACGCCAGCGCTTAATGTAGTA | 9[651] | 5[664] |
| GCAGCACCGTAAGTGCCCGTATA | 4[419] | 4[397] |
| ATGAATCCCAGTCACGATCGAACGTGCCGGCCAGAGCACA | 7[86] | 8[70] |
| TATGTGATAAATAAGGCGTTAAA | 7[525] | 7[547] |
| TTAATGAATCGGCCATTCATTCCAATACGCATAGT | 11[231] | 6[238] |
| ATTCTTTTCATAATCAAAATCAC | 8[447] | 8[425] |
| AATCGTTGAGTAACATTGGAATTACCTAATTACATTTAAC | 7[590] | 8[574] |
| ATTTTGCCAGAGGGGGTAATAGT | 8[279] | 8[257] |
| AGCGCCACCACGGAATACGCCTCAGACCAGAGCCACCACC | 7[422] | 8[406] |
| AAAAAAGGCAGCCTTTACAATCTTACCAGTTTG | 0[473] | 11[482] |
| TAATCGTAGCATTACCTGAGAGTCTG | 8[172] | 9[188] |
| CAAGTGCTGAGTAAGAAAATAAATCCTC | 6[405] | 2[392] |
| GGCTAAAGTACGGTGTCTGGAAG | 7[189] | 7[211] |
| CCTACATACGTAGCGGCCAGCCATTGCAACAGGTTTTT | 8[678] | 9[698] |
| CTATTTCGGAACGAGTGAGAATA | 4[377] | 4[355] |
| TCAACATCAGTTAAATAGCGAGAGTGAGACGACGATAAAA | 4[270] | 7[272] |
| AATAACGCGCGGGGAGAGG | 10[247] | 11[260] |
| AAGAGATTCATTTTGTTTAAGAGGAAGC | 6[237] | 2[224] |
| CAAATGGTTCAGAAGAACGAGTAGAT | 8[214] | 9[230] |
| AAAAGGGCGACAATTATTTATCC | 3[434] | 3[456] |
| ATAGCTGTTTCCTGGAACGTCCATAACGCCGTAAA | 11[63] | 6[70] |
| TGTAGGGGATTTAGTAACACTGAGTTTC | 2[349] | 3[349] |
| AAAAATCTACGTGCGTTTTAATT | 0[244] | 0[222] |
| AGAGTTTATACCAGTAGCACCTGAAACCATCGATA | 5[413] | 4[420] |
| GTGTATTAAGAGGCTGAGACTCC | 7[357] | 7[379] |
| GAAGTCAACCCAAATGGCAAAAGAATACTCGGAACAGAATCC | 9[273] | 5[286] |
| CGGTTAACAAAGCTGCTGTAACAACAAGGACGTTGGGAAG | 11[261] | 0[245] |
| ACTACCTTTAAACGGGTAACAGGGAGACGGGCA | 0[305] | 11[314] |
| AATCCAAAAAAAAGGCTCCAAAA | 7[315] | 7[337] |
| GAGAGCCTCAGAACCGCATTTTCTGTAACGATCTAAAGTT | 11[345] | 0[329] |
| AAATCCCCGAAACAATTCATGAGGAAGT | 6[321] | 2[308] |
| TACCTAATATCAAAATCATTCAATATTACGTGA | 0[557] | 11[566] |
| GTATACAGGTAATGTGTAGGTAGTCAAATCACCAT | 5[161] | 4[168] |
| AACGTTGTAGAAACAGCGGATAGTTGGGCGGTTGT | 5[77] | 4[84] |
| GTTTATGTCACATGGGAATCCAC | 3[415] | 10[416] |
| ATATTCACAAACAAATTCATATG | 3[392] | 3[414] |
| GACCGGAAGCAATTGCGGGAGAA | 0[202] | 0[180] |
| TCAAGCAGAACCACCACTCACTCAGGTAGCCCGGAATAGG | 7[380] | 8[364] |
| AGCCTCCCCAGGGTCCGGCAAACGCG | 8[88] | 9[104] |
| TTCATTTTCTGCTAAACAACTGAACAACTAAAGGA | 5[329] | 4[336] |
| TCGTTCACCGCCTGGCCCT | 10[331] | 11[344] |
| CGGAAGCACGCAAACTTATTAGCGTT | 8[424] | 9[440] |
| GAGCAAGGTGGCATTTACTCCAACAGGTTCTTTACGTCAACA | 9[189] | 5[202] |
| ATTGCGAATAATGTACAACGGAG | 4[335] | 4[313] |
| CTTTTTTTCGTCTCGTCGCTGGC | 8[111] | 8[89] |

| | | |
|---|---|---|
| GACCGTCGAACGGGGAAGCTAATGCAGA | 6[531] | 2[518] |
| GCGTCATACATGCCCTCATAGTT | 0[370] | 0[348] |
| GAAAGTTCAACAATCAGCTTGCTTAGCTTTAATTGTATCG | 4[354] | 7[356] |
| TGTAAATCATGCTCCTTTTGATAATTGCTGAATAT | 5[203] | 4[210] |
| TTCACCTAGCGTGGCGGGTGAAGGGATACCAGTGCATAAAAA | 9[63] | 5[76] |
| ATTTGCCAAGCGGAACTGACCAACGAGTCAATCATAAGGG | 4[312] | 7[314] |
| TAGAACCTACCAGTCTGAGAGAC | 0[580] | 0[558] |
| GGGTTACCTGCAGCCAGCGGTGTTTTT | 4[51] | 4[29] |
| GAATTATCCAATAACGATAGCTTAGATT | 2[559] | 3[559] |
| TTGTCGTCTTTCTACGTAATGCC | 0[328] | 0[306] |
| ACTACTTAGCCGGAACGAGGCGC | 7[273] | 7[295] |
| TTTTTGTCCATCACGCAAATTCCGAGTAAAAGAGTCTTTTTT | 4[702] | 5[702] |
| TTTTTCGGGAGCTAAACAGGTTGTTAGAATCAGAGTTTTT | 0[694] | 1[694] |
| AATCATAATAACCCGGCGTCAAAAATGA | 6[489] | 2[476] |
| AGCAAGCCGTTTAAGAATTGAGT | 4[503] | 4[481] |
| AACAGAGTGCCTGGGGTTTTGCTCACAGAAGGATTAGGAT | 4[396] | 7[398] |
| CCAGCCAAACTTCTGATTGCCGTTTTGGGTAAAGTTAAAC | 4[102] | 7[104] |
| TGAAATTGTTTCAGGGAACTACAACGCC | 6[363] | 2[350] |
| GCCCGCACAGGCGGCCTTTAGTG | 7[63] | 7[85] |
| CAGTAAGAACCTTGAGCCTGTTTAGT | 8[550] | 9[566] |
| ACCAAATTACCAGGTCATAGCCCCGAGTTTTCATCGGCAT | 4[438] | 7[440] |
| TCTTATACTCAGAAAGGCTTTTGATGATATTGACACGCTATT | 9[357] | 5[370] |
| GCCTTATACCCTGTAATACCAATTCTTGCGCTC | 0[179] | 11[188] |
| TTTTTGCGTCCGTGCCTGCATCAGACGTTTTT | 9[25] | 6[21] |
| TTATGGCCTGAGCACCTCAGAGCATAAA | 2[181] | 3[181] |
| CGAGCACAGACTTCAAATACCTCAAAAGCTGCA | 0[221] | 11[230] |
| GCATCAAAAAGAAGTAAATTGGG | 3[224] | 3[246] |
| TAAGTAGAAGAACTCAAACTATCG | 7[651] | 7[673] |
| ATTTGGCAAATCAACAGTTGAAA | 7[609] | 7[631] |
| GTTGAAACAAACATCAAGAAAAC | 8[615] | 8[593] |
| GAATTGTAGCCAGAATGGATCAGAGCAAATCCT | 0[389] | 11[398] |
| GCTTGACCATTAGATACATTTCG | 8[237] | 8[215] |
| CTGAAAACCTGTTTATCAAACATGTAACGTCAA | 0[515] | 11[524] |
| GACTTTCTCCGTGGCGCGGTTG | 0[76] | 0[54] |
| ACACAACATACGAGGGATGTGGCTATTAATCGGCC | 11[105] | 6[112] |
| TTTTTAACAATATTACCGTCGCTGGTAATATCCAGTTTTT | 6[694] | 7[694] |
| TGCCTGAACAGCAAATGAATGCGCGAACT | 6[657] | 2[644] |
| CAAATATCAAACCAGATGAATAT | 4[629] | 4[607] |
| CAATATGATATTGATGGGCGCAT | 4[167] | 4[145] |
| TTCTGGAATAATCCTGATTTTGCCCGGCCGTAA | 0[599] | 11[608] |
| TTAACAAGAGAATCGATGAACGG | 8[195] | 8[173] |
| GGGCCGGAAGCATAAAGTG | 10[121] | 11[134] |
| GTTTGAGGGGACCTCATTTGCCG | 4[125] | 4[103] |
| GTATTAGAGCCGTCAATAGATAA | 8[657] | 8[635] |
| GCTAATGCCGGAGAGGGTAGCTA | 7[147] | 7[169] |
| TACTTCTTTGATAAAAATCTAAA | 4[671] | 4[649] |
| GAAAGATCGCACTCCAGCCAGCT | 7[105] | 7[127] |
| TCAGGCTGCGCAACTGTTGGGAA | 8[153] | 8[131] |
| ATACCCTTCGTGCCACGCTGAACCTTGCTGAACCT | 5[623] | 4[630] |

| | | |
|---|---|---|
| CATAATATTCCGTAATGGGATCCGTGCATCTGCCA | 5[119] | 4[126] |
| TTTTTATCCAATAAATCTCTACCCCGGTAAAACTAGCATG | 7[170] | 8[154] |
| CCGATAATAAAAGGGACTTAACACCGCGAACCACCAGCAG | 11[639] | 0[623] |
| CATCAGCGTCTGGCCTTCCACAGGAACCTGGGG | 0[137] | 11[146] |
| GGAATAACAGAGATAGACATACAAACTTGAGGATTTAGAA | 7[632] | 8[616] |
| CCGGAAGACGTACAGCGCCGCGATTACAATTCC | 0[95] | 11[104] |
| TTCGCGGATTGATTGCTCATTTTTTAAC | 2[139] | 3[139] |
| TAAAGGATTGTATAAGCGCACAAACGACATTAAATGTGAG | 11[135] | 0[119] |
| GATAAAAATTTTTAGCCAGCTTT | 0[160] | 0[138] |
| GATAGTGCAACATGATATTTTTGAATGG | 2[643] | 3[643] |
| GGATAACCTCACAATTTTTGTTA | 3[98] | 3[120] |
| TCAATAATAAAGTGTATCATCATATTCC | 2[601] | 3[601] |
| CAATAGGAACGCAAATTAAGCAA | 3[140] | 3[162] |
| GCGAAAGACGCAAAGCCGCCACGGGAAC | 2[97] | 3[97] |
| TTCCGAATTGTAAACGTGTCGCCAGCATCGGTGCGGGCCT | 7[128] | 8[112] |
| ACATCATTTAAATTGCGTAGAAACAGTACCTTTTA | 5[581] | 4[588] |
| AAGATAAAACAGTTGGATTATAC | 0[622] | 0[600] |
| AACACCCTAAAGGGAGCCC | 10[625] | 11[638] |
| GCATCGAGCCAGATATCTTTAGGACCTGAGGAAGGTTATC | 4[648] | 7[650] |
| CGTAAAGGTCACGAAACCAGGCAATAGCACCGCTTCTGGT | 4[144] | 7[146] |
| CGAGTAACAACCGTTTACCAGTC | 0[118] | 0[96] |
| GCCTTACGCTGCGCGTAAAATTATTTTTTGACGCTCAATC | 7[674] | 8[658] |
| CCGAACCCCCTAAAACATCGACCAGTTTAGAGC | 0[641] | 11[650] |
| TGCGTACTAATAGTAGTTGAAATGCATATTTCAACGCAAG | 11[177] | 0[161] |
| GATTTTAGACAGGCATTAAAAATA | 0[664] | 0[642] |
| TGATTATCAGATATACGTGGCAC | 3[602] | 3[624] |
| TGGCAAGTTTTTTGGGGTC | 10[583] | 11[596] |
| TCAGCTAACTCACATTAAT | 10[163] | 11[176] |
| CTATTAGTCTTTCGCCGCTACAG | 3[644] | 3[666] |
| AACGCCAAAAGGCGGATGGCTTA | 4[251] | 4[229] |
| AAGAAACAATGACCGGAAACGTC | 4[461] | 4[439] |
| GTACATCGACATCGTTAACGGCA | 4[83] | 4[61] |
| ATACCACCATCAGTGAGGCCAAACCGTTGTAGCAA | 5[665] | 4[672] |

**Table S16**. Unmodified staple strands of NRO DNA origami. Sequences are denoted from 5'- to 3'-end. The numbers for the 5'- end 3'-end of the staples represent the helix number in the corresponding caDNAno file. Number in brackets represent the starting and ending position of the staple in the corresponding helix.

| Unmodified staple strands | 5'-end | 3'-end |
|---|---|---|
| CATAAATCTTTGAATACCAAGTGTTAGAAC | 17[224] | 19[223] |
| AAGCCTGGTACGAGCCGGAAGCATAGATGATG | 20[143] | 19[159] |
| TCATTCAGATGCGATTTTAAGAACAGGCATAG | 5[96] | 7[95] |
| GCCATCAAGCTCATTTTTTAACCACAAATCCA | 16[143] | 15[159] |
| TATAACTAACAAAGAACGCGAGAACGCCAA | 16[175] | 14[176] |
| TTGCTCCTTTCAAATATCGCGTTTGAGGGGGT | 10[111] | 8[112] |
| GTATAGCAAACAGTTAATGCCCAATCCTCA | 1[224] | 3[223] |
| AAAGTCACAAAATAAACAGCCAGCGTTTTA | 9[224] | 11[223] |
| GGCCTTGAAGAGCCACCACCCTCAGAAACCAT | 3[192] | 5[191] |
| TTAACGTCTAACATAAAAACAGGTAACGGA | 10[175] | 8[176] |
| AGTATAAAGTTCAGCTAATGCAGATGTCTTTC | 14[239] | 12[240] |

| | | |
|---|---|---|
| GATGTGCTTCAGGAAGATCGCACAATGTGA | 18[79] | 16[80] |
| TCAAATATAACCTCCGGCTTAGGTAACAATTT | 15[192] | 17[191] |
| TTTCGGAAGTGCCGTCGAGAGGGTGAGTTTCG | 2[207] | 0[208] |
| GAGGGTAGGATTCAAAAGGGTGAGACATCCAA | 14[111] | 12[112] |
| TATTAAGAAGCGGGGTTTTGCTCGTAGCAT | 2[175] | 0[176] |
| GCCCTTCAGAGTCCACTATTAAAGGGTGCCGT | 21[64] | 23[63] |
| ATGCAGATACATAACGGGAATCGTCATAAATAAAGCAAAG | 7[56] | 9[63] |
| AGCCAGCAATTGAGGAAGGTTATCATCATTTT | 22[207] | 20[208] |
| TAAATGAATTTTCTGTATGGGATTAATTTCTT | 0[111] | 1[95] |
| AAACAGCTTTTTGCGGGATCGTCAACACTAAA | 1[96] | 3[95] |
| CGGATTCTGACGACAGTATCGGCCGCAAGGCGATTAAGTT | 16[63] | 18[56] |
| GCAATTCACATATTCCTGATTATCAAAGTGTA | 19[160] | 20[144] |
| GCGCAGACAAGAGGCAAAAGAATCCCTCAG | 4[79] | 2[80] |
| AGAGAGAAAAAAATGAAAATAGCAAGCAAACT | 9[160] | 10[144] |
| GACAAAAGGTAAAGTAATCGCCATATTTAACAAAACTTTT | 13[184] | 15[191] |
| ACACTCATCCATGTTACTTAGCCGAAAGCTGC | 3[96] | 5[95] |
| CTACCATAGTTTGAGTAACATTTAAAATAT | 19[224] | 21[223] |
| TATATTTTGTCATTGCCTGAGAGTGGAAGATTGTATAAGC | 13[64] | 15[71] |
| CGGATTGCAGAGCTTAATTGCTGAAACGAGTA | 9[64] | 11[63] |
| TAAATCATATAACCTGTTTAGCTAACCTTTAA | 12[111] | 10[112] |
| GTACCGCAATTCTAAGAACGCGAGTATTATTT | 12[207] | 10[208] |
| TCTTCGCTGCACCGCTTCTGGTGCGGCCTTCC | 18[111] | 16[112] |
| GATTTAGTCAATAAAGCCTCAGAGAACCCTCA | 11[64] | 13[63] |
| GCAAGGCCTCACCAGTAGCACCATGGGCTTGA | 5[160] | 6[144] |
| ATTACCTTTGAATAAGGCTTGCCCAAATCCGC | 6[111] | 4[112] |
| CTTATCATTCCCGACTTGCGGGAGCCTAATTT | 12[239] | 10[240] |
| TTATACCACCAAATCAACGTAACGAACGAG | 6[79] | 4[80] |
| GTAATAAGTTAGGCAGAGGCATTTATGATATT | 13[160] | 14[144] |
| CAACCGTTTCAAATCACCATCAATTCGAGCCA | 14[143] | 13[159] |
| GATGGTTTGAACGAGTAGTAAATTTACCATTA | 6[143] | 5[159] |
| GCACAGACAATATTTTTGAATGGGGTCAGTA | 23[224] | 22[240] |
| AGCAAGCGTAGGGTTGAGTGTTGTAGGGAGCC | 21[96] | 23[95] |
| TCCACAGACAGCCCTCATAGTTAGCGTAACGA | 0[175] | 0[144] |
| TCACCAGTACAAACTACAACGCCTAGTACCAG | 0[207] | 1[191] |
| ATTATACTAAGAAACCACCAGAAGTCAACAGT | 19[192] | 21[191] |
| TAAGAGCAAATGTTTAGACTGGATAGGAAGCC | 7[96] | 9[95] |
| ATACATACCGAGGAAACGCAATAAGAAGCGCATTAGACGG | 7[192] | 9[199] |
| CAACTGTTGCGCCATTCGCCATTCAAACATCA | 18[143] | 17[159] |
| GATGGCTTATCAAAAAGATTAAGAGCGTCC | 10[79] | 8[80] |
| TAGGTAAACTATTTTTGAGAGATCAAACGTTA | 13[96] | 15[95] |
| AGGCAAAGGGAAGGGCGATCGGCAATTCCA | 17[128] | 19[127] |
| ATTATCATTCAATATAATCCTGACAATTAC | 20[175] | 18[176] |
| GAAATTATTGCCTTTAGCGTCAGACCGGAACC | 6[239] | 4[240] |
| AATGGTCAACAGGCAAGGCAAAGAGTAATGTG | 11[96] | 13[95] |
| CCAATAGCTCATCGTAGGAATCATGGCATCAA | 11[160] | 12[144] |
| ATACCCAACAGTATGTTAGCAAATTAGAGC | 8[175] | 6[176] |
| ATAAGGGAACCGGATATTCATTACGTCAGGACGTTGGGAA | 4[63] | 6[56] |
| CACCAGAAAGGTTGAGGCAGGTCATGAAAG | 4[175] | 2[176] |
| ATCCCAATGAGAATTAACTGAACAGTTACCAG | 10[207] | 8[208] |

| | | |
|---|---|---|
| CATGTAATAGAATATAAAGTACCAAGCCGT | 14[175] | 12[176] |
| CCAACAGGAGCGAACCAGACCGGAGCCTTTAC | 10[143] | 9[159] |
| GCTATCAGAAATGCAATGCCTGAATTAGCA | 14[79] | 12[80] |
| GACCTGCTCTTTGACCCCCAGCGAGGGAGTTA | 4[111] | 2[112] |
| AGGAACCCATGTACCGTAACACTTGATATAA | 0[239] | 1[223] |
| CAGCGAAACTTGCTTTCGAGGTGTTGCTAA | 2[79] | 0[80] |
| GCTTTCCGATTACGCCAGCTGGCGGCTGTTTC | 17[96] | 19[95] |
| ACAACTTTCAACAGTTTCAGCGGATGTATCGG | 0[79] | 1[63] |
| CAGCAAAAGGAAACGTCACCAATGAGCCGC | 6[175] | 4[176] |
| ACCTTTTTATTTTAGTTAATTTCATAGGGCTT | 16[207] | 14[208] |
| CGATAGCATTGAGCCATTTGGGAACGTAGAAA | 5[192] | 7[191] |
| GCCCGAGAGTCCACGCTGGTTTGCAGCTAACT | 22[111] | 20[112] |
| ATTTTAAAATCAAAATTATTTGCACGGATTCG | 20[239] | 18[240] |
| ACCTTGCTTGGTCAGTTGGCAAAGAGCGGA | 22[175] | 20[176] |
| CTGAGCAAAAATTAATTACATTTTGGGTTA | 18[175] | 16[176] |
| CCTGATTGCAATATATGTGAGTGATCAATAGT | 18[239] | 16[240] |
| TCAATATCGAACCTCAAATATCAATTCCGAAA | 21[160] | 22[144] |
| AAAGGCCGGAGACAGCTAGCTGATAAATTAATTTTTGT | 13[120] | 15[127] |
| CTTTAGGGCCTGCAACAGTGCCAATACGTG | 21[224] | 23[223] |
| AATAGTAAACACTATCATAACCCTCATTGTGA | 8[111] | 6[112] |
| TCACCGACGCACCGTAATCAGTAGCAGAACCG | 6[207] | 4[208] |
| GCCCGTATCCGGAATAGGTGTATCAGCCCAAT | 2[239] | 0[240] |
| TGTAGCCATTAAAATTCGCATTAAATGCCGGA | 16[111] | 14[112] |
| TCGGCAAATCCTGTTTGATGGTGGACCCTCAA | 22[143] | 21[159] |
| TGACAACTCGCTGAGGCTTGCATTATACCA | 1[128] | 3[127] |
| CCACCCTCTATTCACAAACAAATACCTGCCTA | 4[207] | 2[208] |
| CCCGATTTAGAGCTTGACGGGGAAAAAGAATA | 23[96] | 22[112] |
| AAGTAAGCAGACACCACGGAATAATATTGACG | 8[239] | 6[240] |
| AAATTAAGTTGACCATTAGATACTTTTGCG | 12[79] | 10[80] |
| CACATTAAAATTGTTATCCGCTCATGCGGGCC | 20[111] | 18[112] |
| TTAAAGCCAGAGCCGCCACCCTCGACAGAA | 3[224] | 5[223] |
| ATATTCGGAACCATCGCCCACGCAGAGAAGGA | 2[143] | 1[159] |
| TTCTACTACGCGAGCTGAAAAGGTTACCGCGC | 12[143] | 11[159] |
| AACGTGGCGAGAAAGGAAGGGAAACCAGTAA | 23[128] | 23[159] |
| GAATTTATTTAATGGTTTGAAATATTCTTACC | 16[239] | 14[240] |
| AGCGCGATGATAAATTGTGTCGTGACGAGA | 3[128] | 5[127] |
| AACGCAAAGATAGCCGAACAAACCCTGAAC | 7[224] | 9[223] |
| AGAAAACAAAGAAGATGATGAAACAGGCTGCG | 17[160] | 18[144] |
| CGCGCAGATTACCTTTTTTAATGGGAGAGACT | 18[207] | 16[208] |
| CACAACAGGTGCCTAATGAGTGCCCAGCAG | 19[128] | 21[127] |
| GCGGAACATCTGAATAATGGAAGGTACAAAAT | 20[207] | 18[208] |
| TAAAAGGGACATTCTGGCCAACAAAGCATC | 23[160] | 22[176] |
| AATTGAGAATTCTGTCCAGACGACTAAACCAA | 14[207] | 12[208] |
| GCGAAAAATCCCTTATAAATCAAGCCGGCG | 21[128] | 23[127] |
| AACACCAAATTTCAACTTTAATCGTTTACC | 5[128] | 7[127] |
| TAAATCAAAATAATTCGCGTCTCGGAAACC | 15[128] | 17[127] |
| GAAACGATAGAAGGCTTATCCGGTCTCATCGAGAACAAGC | 10[191] | 12[184] |
| GCCTCCCTCAGAATGGAAAGCGCAGTAACAGT | 4[239] | 2[240] |
| GCGAACCTCCAAGAACGGGTATGACAATAA | 11[224] | 13[223] |

| | | |
|---|---|---|
| TTAGGATTGGCTGAGACTCCTCAATAACCGAT | 1[160] | 2[144] |
| ATCGCAAGTATGTAAATGCTGATGATAGGAAC | 15[160] | 16[144] |
| GCGGATAACCTATTATTCTGAAACAGACGATT | 1[192] | 3[191] |
| AAGGAAACATAAAGGTGGCAACATTATCACCG | 8[207] | 6[208] |
| ACCCTTCTGACCTGAAAGCGTAAGACGCTGAG | 23[192] | 22[208] |
| ATATTTTGGCTTTCATCAACATTATCCAGCCA | 15[96] | 17[95] |
| TCAAGTTTCATTAAAGGTGAATATAAAAGA | 5[224] | 7[223] |
| TCTAAAGTTTTGTCGTCTTTCCAGCCGACAA | 0[143] | 1[127] |
| TTCCAGTCGTAATCATGGTCATAAAAGGGG | 20[79] | 18[80] |
| AAAGCACTAAATCGGAACCCTAATCCAGTT | 23[64] | 22[80] |
| AATACTGCCCAAAAGGAATTACGTGGCTCA | 8[79] | 6[80] |
| TTTATCAGGACAGCATCGGAACGACACCAACCTAAAACGA | 1[64] | 3[71] |
| TTGACAGGCCACCACCAGAGCCGCGATTTGTA | 3[160] | 4[144] |
| CTGTGTGATTGCGTTGCGCTCACTAGAGTTGC | 19[96] | 21[95] |
| GCGAGTAAAAATATTTAAATTGTTACAAAG | 16[79] | 14[80] |
| TAGAGAGTTATTTTCATTTGGGGATAGTAGTAGCATTA | 10[127] | 12[120] |
| CGAAAGACTTTGATAAGAGGTCATATTTCGCA | 9[96] | 11[95] |
| TCATCGCCAACAAAGTACAACGGACGCCAGCA | 4[143] | 3[159] |
| TTAACACCAGCACTAACAACTAATCGTTATTA | 22[239] | 20[240] |
| TTATTACGAAGAACTGGCATGATTGCGAGAGG | 7[160] | 8[144] |
| GCCAGTTAGAGGGTAATTGAGCGCTTTAAGAA | 10[239] | 8[240] |
| ACAACATGCCAACGCTCAACAGTCTTCTGA | 13[224] | 15[223] |
| CATTTGAAGGCGAATTATTCATTTTTGTTTGG | 17[192] | 19[191] |
| TGAAAGGAGCAAATGAAAAATCTAGAGATAGA | 21[192] | 23[191] |
| TGGAACAACCGCCTGGCCCTGAGGCCCGCT | 22[79] | 20[80] |
| TACCGAGCTCGAATTCGGGAAACCTGTCGTGCAGCTGATT | 19[56] | 21[63] |
| GTTTATTTTGTCACAATCTTACCGAAGCCCTTTAATATCA | 7[248] | 9[255] |
| ACAAACGGAAAAGCCCCAAAAACACTGGAGCA | 16[47] | 14[48] |
| GTTTATCAATATGCGTTATACAAACCGACCGTGTGATAAA | 13[256] | 15[263] |
| ACGGCTACAAAAGGAGCCTTTAATGTGAGAAT | 2[47] | 0[48] |
| GACCAACTAATGCCACTACGAAGGGGGTAGCA | 4[47] | 2[48] |
| AAGGCCGCTGATACCGATAGTTGCGACGTTAG | 2[111] | 0[112] |
| CTCCAACGCAGTGAGACGGGCAACCAGCTGCA | 22[47] | 20[48] |
| ACCGATTGTCGGCATTTTCGGTCATAATCA | 6[271] | 4[272] |
| CAGAAGATTAGATAATACATTTGTCGACAA | 22[271] | 20[272] |
| TGCATCTTTCCCAGTCACGACGGCCTGCAG | 17[32] | 19[31] |
| TTAGTATCACAATAGATAAGTCCACGAGCA | 14[271] | 12[272] |
| GTTTTAACTTAGTACCGCCACCCAGAGCCA | 2[271] | 0[272] |
| TTAATGAACTAGAGGATCCCCGGGGGGTAACG | 20[47] | 18[48] |
| CTTTTACAAAATCGTCGCTATTAGCGATAG | 18[271] | 16[272] |
| ATCCCCCTATACCACATTCAACTAGAAAAATC | 8[47] | 6[48] |
| AGAAAGGAACAACTAAAGGAATTCAAAAAAA | 0[47] | 1[31] |
| TTTTATTTAAGCAAATCAGATATTTTTTGT | 12[175] | 10[176] |
| AGCCACCACTGTAGCGCGTTTTCAAGGGAGGGAAGGTAAA | 4[255] | 6[248] |
| AACAAGAGGGATAAAAATTTTTAGCATAAAGC | 14[47] | 12[48] |
| GCCGTCAAAAAACAGAGGTGAGGCCTATTAGT | 21[256] | 23[255] |
| TGTAGAAATCAAGATTAGTTGCTCTTACCA | 12[271] | 10[272] |
| GAGAGATAGAGCGTCTTTCCAGAGGTTTTGAA | 9[256] | 11[255] |
| CCACCCTCATTTTCAGGGATAGCAACCGTACT | 0[271] | 1[255] |

| | | |
|---|---|---|
| CTTTAATGCGCGAACTGATAGCCCCACCAG | 23[256] | 22[272] |
| CCAGGGTTGCCAGTTTGAGGGGACCCGTGGGA | 18[47] | 16[48] |
| CAAATCAAGTTTTTTGGGGTCGAAACGTGGA | 23[32] | 22[48] |
| ACGCTAACACCCACAAGAATTGAAAATAGC | 10[271] | 8[272] |
| CTTTTGCAGATAAAAACCAAAATAAAGACTCC | 8[143] | 7[159] |
| TACGTTAAAGTAATCTTGACAAGAACCGAACT | 6[47] | 4[48] |
| TAATCAGCGGATTGACCGTAATCGTAACCG | 15[32] | 17[31] |
| TTTTCACTCAAAGGGCGAAAAACCATCACC | 21[32] | 23[31] |
| GCCTTAAACCAATCAATAATCGGCACGCGCCT | 11[256] | 13[255] |
| AATAGCTATCAATAGAAAATTCAACATTCA | 8[271] | 6[272] |
| CATCAAGTAAAACGAACTAACGAGTTGAGA | 5[32] | 7[31] |
| CAGGAGGTGGGGTCAGTGCCTTGAGTCTCTGAATTTACCG | 1[256] | 3[263] |
| AAATCACCTTCCAGTAAGCGTCAGTAATAA | 4[271] | 2[272] |
| CTCGTATTAGAAATTGCGTAGATACAGTAC | 20[271] | 18[272] |
| TTTACCCCAACATGTTTTAAATTTCCATAT | 9[32] | 11[31] |
| CCTAAATCAAAATCATAGGTCTAAACAGTA | 15[224] | 17[223] |
| GTCGACTTCGGCCAACGCGCGGGGTTTTTC | 19[32] | 21[31] |
| CGTAAAACAGAAATAAAAATCCTTTGCCCGAAAGATTAGA | 19[248] | 21[255] |
| AGGCTCCAGAGGCTTTGAGGACACGGGTAA | 1[32] | 3[31] |
| GAGAAGAGATAACCTTGCTTCTGTTCGGGAGAAACAATAA | 16[255] | 18[248] |
| TTTAGGACAAATGCTTTAAACAATCAGGTC | 7[32] | 9[31] |
| AATACGTTTGAAAGAGGACAGACTGACCTT | 3[32] | 5[31] |
| CTTAGATTTAAGGCGTTAAATAAAGCCTGT | 16[271] | 14[272] |
| TAAATCGGGATTCCCAATTCTGCGATATAATG | 12[47] | 10[48] |
| AACAGTTTTGTACCAAAAACATTTTATTTC | 11[32] | 13[31] |
| CTGTAGCTTGACTATTATAGTCAGTTCATTGA | 10[47] | 8[48] |
| AGACGACAAAGAAGTTTTGCCATAATTCGAGCTTCAA | 7[128] | 9[135] |
| AACGCAAAATCGATGAACGGTACCGGTTGA | 13[32] | 15[31] |

**Table S17**. Unmodified staple strands of 6HB DNA origami. Sequences are denoted from 5'- to 3'-end. The numbers for the 5'- end 3'-end of the staples represent the helix number in the corresponding caDNAno file. Number in brackets represent the starting and ending position of the staple in the corresponding helix.

| Unmodified staple strands | 5'-end | 3'-end |
|---|---|---|
| TACAACGAAAGGAGGTAAAATACGTAATAGGCAAAAGAATTT | 0[685] | 3[685] |
| ATCTCTGACCTCCTAGTCGGGAAACCTGGCACGAATATAGTT | 4[195] | 0[182] |
| ACCTTTTTAACCCATCATAGGTCTGAGATTAGTGAATTTATC | 3[1232] | 1[1245] |
| ACCATCAGCGTCCAAATAGTAAAATGTTTAAGAGGCTTTTGC | 5[546] | 2[546] |
| ATCAATATCTGGTCAGTTGGCAAATC | 3[1372] | 3[1397] |
| GTGTCTTTCCTTATCACTCATCGAGAACATTATTTACAAGAA | 1[1120] | 4[1120] |
| TCAGAGGCATTGCAAACGATTTTTTGTTGAGAGAATAACATA | 0[1063] | 3[1063] |
| AACAGTTGAAAGGAATTGAGGAAGAG | 2[1397] | 2[1372] |
| CTTTTCTTAAACAGTAACCACCACACCCAAAGGCTCCAAACA | 4[741] | 0[728] |
| AACCGTTCTAGCAAAGGCCGGAGACAGTGATTCAAAAATCCT | 2[377] | 5[377] |
| TATCCTGACGCTCAGGCTTATCCGGTATCGACTTGCGGGACA | 0[1105] | 3[1105] |
| GCTGCAAGGCGATGCCTCTTCGCTATTAAAGGGCGTGCGTAT | 2[251] | 5[251] |
| AGATTTAAGTCCACTTAATTGCTGAATAAACTAAAGTACGGG | 0[475] | 3[475] |
| GGCCAACAGAATATGAGGCATTTTCGAGAGCGCCATATTTAA | 5[1176] | 2[1176] |
| GCGCGTTTTCATCGAGCGACAGAATCAAAGCAGCACCGAGTA | 2[923] | 5[923] |
| ATAACGTACACTGAGCCCAATAGGAACCATCCTCAGAGCCAC | 5[798] | 2[798] |

| | | |
|---|---|---|
| GGAGGCCAGGATTATAAGAGGCTGAGACTGTATTTCGGAACC | 5[840] | 2[840] |
| TGCCCAGCGATTATGTATCATCGCCTGAAGAAAGGCGAAGGC | 1[658] | 4[658] |
| ATGCGCGATAGCTTCCTTAGAATCCTTGACCTTGCTTCTGTA | 5[1260] | 2[1260] |
| TACGAGCCGGAAGCTCGAATTCGTAA | 1[142] | 1[167] |
| AAAGAGTCATCTTTTCATAGCCCCCTTAACCGTCAGACTGTA | 5[924] | 2[924] |
| AATAATTAATTTTCAGATTAAGACGCTGGCTATTAGAAACAG | 1[1246] | 4[1246] |
| TATCCGGCTTAGGTAATATTTTTGAATGAGAAGAGTCAATTA | 4[1245] | 0[1232] |
| CAATAATCGGCTAGAATATCCCATCCTAGTCCTGAACATTGG | 2[1133] | 5[1133] |
| CAACTCGTATTACAACTTTACAAACAATATGATTTAGAAGTA | 3[1358] | 1[1371] |
| AGAATAAGTTTATTATAACATCACTTGCCGTAGAAAATACAG | 4[993] | 0[980] |
| TAATAAGAGAGATATTATACAAATTCTTTTAATTGAGAATAA | 0[1189] | 3[1189] |
| ACGCTGAGAGCCAGCAGCAAATGAAAAATCTAA | 5[1372] | 5[1404] |
| CAACGCCAACATGACTCAACAGTAGGGCACCAGTAACATTCT | 2[1175] | 5[1175] |
| AAAGCGCCATTCCGTGGTGCCGGAAACCCCTTTCCGGCACCG | 3[266] | 1[279] |
| TATTATTCTGAACGCGTATAAACAGTTAGCCTTGACTAAACA | 2[839] | 5[839] |
| TAAACCAACCAGTCTCAACAATAGATAAATTTACGAGCATCA | 0[1147] | 3[1147] |
| ATCGCGCACCACCAAATTGCGTAGATTTAGTACCTTTTACAT | 0[1315] | 3[1315] |
| TACCGTAGCTTTCCCCGTACTCAGGAGGCAGAACCGCCACAA | 0[811] | 3[811] |
| TTGTTATCTAAAATATCTTTAGGAGCACTA | 1[1372] | 1[1401] |
| ATTATGATAGCCCGTACTAATAGTAGTACAAAGAATTAGCGC | 0[433] | 3[433] |
| ACCGGCATTTTCGGTCATAATCAAAATCGAGGCCACCGTAAT | 1[910] | 4[910] |
| AATGGCATCAATTCAGATAGGGTTGAGTCGCAAATGGTCAAC | 4[447] | 0[434] |
| GTAAACAGGGCTTAAGGAGCTAA | 4[167] | 4[145] |
| GTAAATTGGGCTGAGAAACACCAGAACGGAAGGCTTGCCCTG | 3[602] | 1[615] |
| TAAAGCCTCAGAAATCATACAGGCAAGGGCATTAAATAAATC | 2[419] | 5[419] |
| AAAGCCTGTTTAGCGAATCATAATTACTCCATAAGAATAAAC | 3[1190] | 1[1203] |
| ATGGGCGCATCGTCGCGGATTGACCGTATCTCCGTACGGGCA | 2[293] | 5[293] |
| GAGGCTTGCAGGTTCAACCATCGCCCACTTGCGCCGTGTAGC | 2[713] | 5[713] |
| AATCAGATATAGAAATCGTCTGAAATGGAAGCAAGCCGTTTT | 4[1119] | 0[1106] |
| ATCAATAAACACCGGAACGTTATTAATTAACAAAGAAACCGA | 0[1357] | 3[1357] |
| AACATTAAATCGGAATTTCAACTTTAATTGGCTCATTATATA | 0[601] | 3[601] |
| GCGAACCAAAACCGAAATCAGGTCTTTATGCATCAAAAAGAG | 0[517] | 3[517] |
| TTGACGGATCAACGCAAGAACCGGATATCGCATAGGCTGGCT | 5[630] | 2[630] |
| CACCACCCTCAGAGTTTTTATAATCAGTACCGGAACCAGACT | 4[909] | 0[896] |
| ACAGGAGTGTACCCTACATGGCTTTTGATCGTTCCAGTAAGC | 3[854] | 1[867] |
| CCTGTGAGCGAGTATTCACCGCCTGGCCCGCCATCAAAAACG | 4[321] | 0[308] |
| CACCCTCATTTTACAGAACCGCCACCCTTTTAGTAAGCACGT | 2[797] | 5[797] |
| CCCTCAGCTGCGCGCTTGATACCGATAGGCATAACCGATAGC | 0[727] | 3[727] |
| AATAACGGATTCAAGAATATACAGTAACTCAGGTTAAAATAC | 2[1301] | 5[1301] |
| TAGCATAAAGCTAAATACTTTTGCGGGAATCCCTTCATCCAA | 1[406] | 4[406] |
| TCGCCAGAGGGGGTATACTGCGGAATCGGGCCCACCCTCGTT | 1[532] | 4[532] |
| GAGCTGAAAAGGCATATTTTCATTTGGGAAATAACCTGTTTA | 3[434] | 1[447] |
| GAAAGCGGAGATTTACCAAGCGCGAAACTTACACTAAAACAC | 5[672] | 2[672] |
| GGTCACGCAGCGAAGCTTTTGCGGGATCTTTATTCGGTCGCT | 5[714] | 2[714] |
| GGTGCCTAATGAGTCTACGTGGTGCTTGTT | 3[138] | 3[167] |
| TTTCGAGGTGAAGTATCGGTTTATCAGCGTAGGAGCCTTTAA | 3[728] | 1[741] |
| CTTAACCGTGCATCCTCAGGAAGATCGCCAGTGAGGGGAACA | 1[280] | 4[280] |
| TCAAACTATTACGCAGAACTGGCATGATCAAAACCGAGGAAA | 5[1008] | 2[1008] |
| CCCGAAAGACTTTTGAAGCAAAGCGGATCCCTGACACGTCAA | 2[503] | 5[503] |

| | | |
|---|---|---|
| ATGTAAATATTGACTCACCGACTTGAGCCGTTGTATCATATG | 1[952] | 4[952] |
| TCACGACCAGGGTGGCGCAACTGTTGGGCGCCAGCTGGCGGC | 0[265] | 3[265] |
| ATAGCAAGCAAAATGAATCATTACCGCGATTTTATTTTCATC | 3[1106] | 1[1119] |
| GCTAATGCAGAACGCAATAAACAACATGGTTCTGTCCAGACG | 3[1148] | 1[1161] |
| CATGAGGAAGTTAAGAGGACTAAAGACTAACGGCTACAGAGG | 3[686] | 1[699] |
| ACTCCTTATCGGCCCTTACCGAAGCCCTGTTACCAGAAGGAG | 0[1021] | 3[1021] |
| ATCAACATTAAACCTTCCTGTAGCCAGCGATAATTCGCGTCT | 3[308] | 1[321] |
| CCAAAGTTTTGTCGCGTACTATGGTTGCACAAACTACAACTA | 4[783] | 0[770] |
| TTGGGAAGAAAAAAGCGATTTTAAGAACCATTGTGGCCGTAA | 2[587] | 5[587] |
| AGTGAGATGGTTTAACCCTAAAGGGAGCATTCAGTGAATAAC | 4[615] | 0[602] |
| GCACATGAAAGTATGGATTAGCGGGGTTGCGGGAGGTAACAG | 1[826] | 4[826] |
| AAAGCAATAGCTATTTGCTGGTAATATCAGAGATAACCCAAG | 4[1035] | 0[1022] |
| TTGCACGTAAAACACTACCATATCAAAACAATGGAAGGGTTA | 3[1316] | 1[1329] |
| TTCTTTGATCACCGGGAAATTATTCATTCGATTCAACCGATT | 5[966] | 2[966] |
| GTATGGCTTAGAGCTATTAAAGAACGTGAGGTCAGGATTAGT | 4[489] | 0[476] |
| AGCATCACCTTGCTGAACCTCAAATATCAAACC | 4[1404] | 4[1372] |
| CAGATTCAGTACCGCATTCCAAGAACGGTTGTAGAAACCAAT | 5[1134] | 2[1134] |
| AGGCATGTCAATCACTGGTTTGCCCCAGTCATTGCCTGAGTA | 4[363] | 0[350] |
| TCAAATATATTTCGAAATCCAATCGCAAATATGTAGTAAGAA | 2[1217] | 5[1217] |
| TGGGCGCGTTGTAAACGCCAGGGTTTTCAGAAAGGGGGATGT | 5[252] | 2[252] |
| ACCGGTCAATCATACGGCGAACGTGGCGTAAATTGTGTCGAT | 4[657] | 0[644] |
| GCATCCAAATAAGAACAGGAAAAACGCTCGTCTTTCCAGAAG | 4[1077] | 0[1064] |
| CTCACATTAATTGCGTTGCGCTC | 5[145] | 5[167] |
| ACGCTCGCCCTGCTCAATGTCCCGCCAAGAATTGTAGCTGCA | 2[209] | 5[209] |
| CGGGTACCGAGCATAAAGTGTAAAGCCTGG | 2[167] | 2[138] |
| GGGAGTGACTCTATCAACTCGTCGGTGGTCGTGCCCAACCTT | 1[196] | 4[196] |
| TGTGAATTCATGGGGATGTTCTTCTAAGGGAGGAGAAGCCAG | 3[224] | 1[237] |
| TAGGGAGAATTAACAGCGCTAATATCAGCAGAACAAAAAATG | 1[1036] | 4[1036] |
| GCAACAGTTGATTCCATTAGATACATTTGTTGTTCGTAGCTC | 1[448] | 4[448] |
| ATCGTAAAACTAAAGAGAATCGATGAACGTAGTCTGGAGCAA | 3[350] | 1[363] |
| CATTAAAAGAAGTGCCACCACCCTCAGACAGCATTGACAGCA | 0[895] | 3[895] |
| GTGAAACGTCACCATCACGCAAATTAACCATTTGGGAATTAG | 4[951] | 0[938] |
| GTTTGATAGATCTAGCCGGAGAGGGTAGTCAATATGATATTC | 5[378] | 2[378] |
| AGTGAGAATAGAGTATGGGATTTTGCTAAGTAAATTTAATGC | 2[755] | 5[755] |
| CTGAGTTAAAGGCCAGACAGCATCGGAACTGGCAAGACAATG | 1[700] | 4[700] |
| AAGACACCACGGCAACATATAAAAGAAAAAATACATAAAGGT | 3[980] | 1[993] |
| ACGACAGATTGCCCACAACCCGTCGGATATGGGATAGGTCTC | 0[307] | 3[307] |
| GCCAAAAGGAATATCTAATGCAGATACATAGGAATACCACAT | 3[560] | 1[573] |
| CGTTTGCCTGTCCAATGAAACCATCGATGTTTGCCTTTAGCA | 0[937] | 3[937] |
| TTCATTCCATATTATGTTTTAAATATGCTAATGCTCAGTTTG | 2[461] | 5[461] |
| AATCCTGGCCAGAACACAAACAAATAAAGCGAGGTTGAGGCA | 5[882] | 2[882] |
| GAGGGAGGGAAGCAACCAGCGCCAAAGAAGAAAATGCAATAC | 2[965] | 5[965] |
| GCCGCTACACGTTGAAGGAATTGCGAATGTCAGTTTCAGCGG | 5[756] | 2[756] |
| TAACCATAAATCAATCTATCAGGGCGATTCATAAATATTCAA | 4[531] | 0[518] |
| CCCTCAGAACCGGTCCTCCCTCAGAGCCTCGCCACCACCGGA | 3[896] | 1[909] |
| CTGCCATATCGGCCGTGTCCTTAGTGCTAATAACCCCGCTGT | 0[223] | 3[223] |
| AATTAATTACATATCAAACATCAAGAAAAAAAAGAAGATGAT | 3[1274] | 1[1287] |
| TACGTGGGAAATACTCTTCTGACCTAAAGAGAGAAAACTTTT | 5[1218] | 2[1218] |
| CATTTTGAATCTTATGCACCCAGCTACACCGGTTTTGAAGCC | 5[1092] | 2[1092] |

| | | |
|---|---|---|
| AACAAATATCGCGTAAGCAAACTCCAACGACTCCATATTATA | 1[490] | 4[490] |
| TGATAGGTGTATCATCGTTAGAATCAGATTGCTCAGTACCTG | 4[825] | 0[812] |
| GAATATTCCTGATTGATTGTTTGGATTAAGAGGTGTTTGAGT | 1[1330] | 4[1330] |
| ACGAGTAATCTTGATAACAAAGCTGCTCCCCCGATTTGAAAG | 1[616] | 4[616] |
| CGCAATAATAACCAGATAGCCGAACAAATTTTAAGGAAGAAC | 2[1007] | 5[1007] |
| AACAGAAATAAAGAGCAGAAGATAAAACTACTTCTGAATAAA | 4[1329] | 0[1316] |
| ACAGCTGTATCGGCTGCCAGTTTGAGGGTTACGTTGGTGTAG | 5[294] | 2[294] |
| CGCGCACGACTTAAAACGCGCGGGGAGACCAAGCTTTCTCCC | 4[237] | 0[224] |
| AGTAGTTGCTATTTCCAACGCTAACGAGCATGGAAACGCGAG | 1[1078] | 4[1078] |
| TTAAGGAACAACTAAAAATCTCCAAAAAGCCGCGCGAATTTT | 1[742] | 4[742] |
| GCAAGCGTCATTTTCGCATTAAATTTTTGGTATTTAAATTGT | 5[336] | 2[336] |
| AAACAATGAAATAACCCAATAATAAGAGTACAAGAATTGAGT | 3[1022] | 1[1035] |
| TACAATGCCTGAGTCCGAAATCGGCAAAGAAGCCTTTATTAT | 4[405] | 0[392] |
| ATGGTTTCACAGACTGGGTTATATAACTGACAAAGAACGCCT | 0[1231] | 3[1231] |
| ATTTTTTCAGGGCGTCTTTCCAGACGTTAACAACTTTCAATA | 0[769] | 3[769] |
| AGTTAACAATTTCAAGCCCTAAAACATCATTACCTGAGCAAA | 4[1287] | 0[1274] |
| TTAATGACTGTAAGGATACCGACAGTGCTGTCTAATCTATTT | 5[210] | 2[210] |
| AAAAGAACCCTGTAATCGGTTGTACCAAGCAAAATTAAGCAA | 5[420] | 2[420] |
| ACTCCATTAAACGGCGGGCGCTAGGGCGCGAGGGTAGCAAAG | 4[699] | 0[686] |
| CATTTCTCCGAAGAGACGCATTTCACATGTGGGCCTTGAATC | 3[182] | 1[195] |
| TTTACGAGGCATAGCAAGTTTTTTGGGGAGTTGAGATTTAGA | 4[573] | 0[560] |
| TTTTGAGGGTGGTTAATGTGTAGGTAAACAAATCACCATCAT | 0[391] | 3[391] |
| ACTGATAAATTAATCAAAGGCTATCAGGCAGGCGAAAGGGTG | 1[364] | 4[364] |
| ACGTAATTTAGGCAAAAGTACCGACAAAAAAAGGGTAAAGCC | 1[1162] | 4[1162] |
| TACCCAAGGAAAGCAGGGAACCGAACTGACAGACCAGGCGGA | 0[643] | 3[643] |
| CATGGTAATAAGTTGGGATTTTAGACAGTCTGAATTTACCCT | 4[867] | 0[854] |
| AGCACTATTACAGGAAAACGAACTAACGAGCCAGTCAGGACG | 5[588] | 2[588] |
| GTATAGCCCGGACCTCGAGAGGGTTGATCAAGGCGGATAAGT | 3[812] | 1[825] |
| TCATCTACGTTAATTAGAAAGATTCATCTCGAGGTAATTACC | 1[574] | 4[574] |
| AATCAGCGTCCACGTATGTACCCCGGTTTGTATAAGCAAATA | 0[349] | 3[349] |
| GGAATTATCATCACTTATCATTTTGCGGTTAAAAGAGGCGGT | 2[1343] | 5[1343] |
| AACGCGCCTGTTTAACACGACCAGTAATAGGTAAAGTAATAT | 4[1161] | 0[1148] |
| AAGCCATTCAGGCTGTTTTTCTTTTCACACTCCAGCCAGCAG | 4[279] | 0[266] |
| TTAGCAAGGCCGTTCCAGTAGCACCATTTTAGAGCCAGCAAA | 3[938] | 1[951] |
| ACGAGGCGCAGACACCATGTTACTTAGCTCAAATCCGCGACC | 3[644] | 1[657] |
| CGCCAGCGTAATTGTGAACACCCTGAACCATAAAAACAGGGA | 5[1050] | 2[1050] |
| TCATTTTTGCGGCAGCTCCTTTTGATAAGAGAGAGTACCTTT | 3[476] | 1[489] |
| ACTGCCCGAAATTGTCATGGTCATAGCTAAACGGAGGATCCC | 5[168] | 2[168] |
| TCATCTTTGACCCTACCTAAAACGAAAGGCCACTAAAGGGAA | 2[671] | 5[671] |
| GAACAAGGTTTGACCCAATTCTGCGAACGAGTGTCTGGAAGT | 5[462] | 2[462] |
| CCTGTGTGCTTTCCGGTTGGTGTAATGAACCTCGATAAAGAT | 0[181] | 3[181] |
| CTGGATACCCAAATTAAGAGCAACACTAACCAAAATAGCGAC | 0[559] | 3[559] |
| AAACGTTAATATCCAAAAACAGGAAGATGATAATCAGTTGCA | 2[335] | 5[335] |
| AAAAGAAGTTTTAAAGACGACGATAAAATCATAACTACGTGA | 2[545] | 5[545] |
| ATGTATCATATGCGGAACCCTTCTGACCATAAGGCGTTAAAG | 4[1203] | 0[1190] |
| GGTTAAGTTGGGTAAACGACGGCCAGTGGGCGGTTATCGGTG | 1[238] | 4[238] |
| CGAACGAAGAGGCGTGAATACCAAGTTATTATCGGGAGAAAC | 5[1302] | 2[1302] |
| GGTTTGTTAAAATTTTAACCAATAGGAACTGAGAGAGAAAAG | 1[322] | 4[322] |
| AATCGTCGCTATAATAAATCAATATATGTTTAATGGTCTTTA | 2[1259] | 5[1259] |

| | | |
|---|---|---|
| GTGAATTATTAGTATTGTCACAATCAATCAAAAGGGCGACCA | 0[979] | 3[979] |
| CATAGCGAACTGATTTTGAATTACCTTTTGAGTGAATAACAA | 0[1273] | 3[1273] |
| GAGCCTGATTGCTTAATTATTCATTTCAGCCATTATAACGTC | 1[1288] | 4[1288] |
| AAAACGAGAATGCCATGCTTTAAACAGTCAATTGAATCCCCC | 3[518] | 1[531] |
| GGGGAATACCCAAAAGTATGTTAGCAAACTGAGTAAAAAGTA | 1[994] | 4[994] |
| ACTAGTTAATTTCACGACCGTGTGATAATGAAAGCAATGCTG | 1[1204] | 4[1204] |
| CTAATCCTTTGCCCCCTGCAACAGTGCCAATACATTTGAGTC | 4[1371] | 0[1358] |
| CAGTATTTAATCCTATCAGATGATGGCATCACCAGAAGGAGC | 5[1344] | 2[1344] |
| GGTCAGACGATTCAACCAGAGCCGCCGCGCCGCCAACGCCAG | 2[881] | 5[881] |
| ACAACTAATAGATTAGAGCCGTCAATAGAT | 0[1401] | 0[1372] |
| CCCAGGGATAGCAAGTTTCGTCACCAGTTTTGACGCCGCCAC | 1[784] | 4[784] |
| GTGGCCTTGATATTTGGAAAGCGCAGTCGAACGGTCCAGAAC | 1[868] | 4[868] |
| TTATTTATCCCAGTACAAAATAAACAGCAAGCCTAATTTGCC | 3[1064] | 1[1077] |
| TTATCCGCTCACAATTCCACACAACA | 0[167] | 0[142] |
| GCGTAACGATCTTCAGACAGCCCTCATAAAGCCTGTAGCATT | 3[770] | 1[783] |
| GACCTTCATCAAGACAGATGAACGGTGTACCAACTTTAGAGC | 2[629] | 5[629] |
| CAAGAGAGATTAAATTAACGGGGTCAGTATGCCCCCTGCCAT | 0[853] | 3[853] |
| ATATTTTAAATGAAAATTTTTAGAACCCCTTCAACGCAAGGA | 3[392] | 1[405] |
| TTAAATCAAGATTTTTTAGCGAACCTCCTCTAAGAATACCTA | 2[1091] | 5[1091] |
| AGGGCGAAGACCGGTTTAATTCGAGCTTTCATTAAGAGGAAG | 5[504] | 2[504] |
| AGCGCATTAGACAGTAGCAGCCTTTACATAACGTCATATTAC | 2[1049] | 5[1049] |

## References

[
[1] N. D. Derr, B. S. Goodman, R. Jungmann, A. E. Leschziner, W. M. Shih, S. L. Reck-Peterson, *Science* **2012**, *338*, 662-665.
[2] S. Woo, P. W. Rothemund, *Nat Chem* **2011**, *3*, 620-627.
[3] R. Schreiber, S. Kempter, S. Holler, V. Schüller, D. Schiffels, S. S. Simmel, P. C. Nickels, T. Liedl, *Small* **2011**, *7*, 1795-1799.
[4] P. C. Nickels, B. Wünsch, P. Holzmeister, W. Bae, L. M. Kneer, D. Grohmann, P. Tinnefeld, T. Liedl, *Science* **2016**, *354*, 305-307.
[5] T. Cordes, J. Vogelsang, P. Tinnefeld, *J Am Chem Soc* **2009**, *131*, 5018-5019.
[6] R. Roy, S. Hohng, T. Ha, *Nat Methods* **2008**, *5*, 507-516.
[7] A. D. Edelstein, M. A. Tsuchida, N. Amodaj, H. Pinkard, R. D. Vale, N. Stuurman, *2014* **2014**.
[8] A. Edelstein, N. Amodaj, K. Hoover, R. Vale, N. Stuurman, *Current Protocols in Molecular Biology* **2010**, *92*, 14.20.11-14.20.17.
[9] J. Schnitzbauer, M. T. Strauss, T. Schlichthaerle, F. Schueder, R. Jungmann, *Nature Protocols* **2017**, *12*, 1198-1228.
[10] M. T. Strauss, F. Schueder, D. Haas, P. C. Nickels, R. Jungmann, *Nature Communications* **2018**, *9*, 1600.
[11] J. Vogelsang, R. Kasper, C. Steinhauer, B. Person, M. Heilemann, M. Sauer, P. Tinnefeld, *Angewandte Chemie International Edition* **2008**, *47*, 5465-5469.
[12] Y. Zhang, Q. Li, X. Liu, C. Fan, H. Liu, L. Wang, *Small* **2020**, *16*, 2000793-2000793.
[13] F. C. Simmel, B. Yurke, H. R. Singh, *Chemical Reviews* **2019**, *119*, 6326-6369.
[14] D. Y. Zhang, E. Winfree, *Journal of the American Chemical Society* **2009**, *131*, 17303-17314.

## Author Contributions

C. Forthmann, V. Glembockyte, M. Scheckenbach and P. Tinnefeld designed experiments. M. Scheckenbach performed experiments and data analysis. M. Scheckenbach and T. Schubert performed DNA PAINT experiments and analysis of immobilized nanorulers in degrading conditions (FBS solution). C. Forthmann, V. Glembockyte and P. Tinnefeld supervised the study. V. Glembockyte, M. Scheckenbach and P. Tinnefeld wrote the manuscript. All authors reviewed and approved the manuscript.

## Inhaltsverzeichnis

Inhaltsverzeichnis ..... 2
1. Materialien und Methoden ..... 3
1.1. Allgemeine Materialien ..... 3
1.2. DNA Origami Faltung ..... 3
1.3. Aufreinigung der DNA-Origami-Nanostrukturen ..... 4
1.4. Oberflächenimmobilisierung von DNA-Origami-Nanolinealen ..... 5
1.5. Photostabilisierung von Fluoreszenzmarkierungen ..... 5
1.6. Fluoreszenz-Aufnahmen von DNA-Origami-Helligkeitsstandards ..... 5
1.7. DNA PAINT-Aufnahmen ..... 5
1.8. Auswertung der DNA PAINT Daten ..... 6
1.9. AFM-Aufnahmen mit JPK Nanowizard ..... 6
1.10. Selbstregenerierende Helligkeitsmarkierung auf 12HB Helligkeitsstandard ..... 6
1.11. Reparatur der geknickten 12HB Nanolineale ..... 9
1.12. Staple-Strang-Austausch vom Doppel-Punkt zum Triple-Punkt im NRO-Nanolineal ..... 11
1.13. Selbstheilung von 12HB Nanolinealen unter degradierenden Bedingungen ..... 12
1.14. Selbstregeneration und Selbstheilung einer enzymatisch spaltbaren Helligkeitsmarkierung ..... 13
2. Ergebnisse und Diskussion ..... 15
2.1. Selbstregenerierende Helligkeitsmarkierung auf 12HB Helligkeitsstandard ..... 15
2.2. Reparatur der geknickten 12HB Nanolineale ..... 19
2.3. Staple-Strang-Austausch vom Doppel-Punkt zum Triple-Punkt im NRO-Nanolineal ..... 22
2.4. Selbstheilung von 12HB Nanolinealen unter degradierenden Bedingungen ..... 24
2.5. Selbstregeneration und Selbstheilung einer enzymatisch spaltbaren Helligkeitsmarkierung ..... 27
3. Anhang ..... 29
Verweise ..... 42
Autorenbeiträge ..... 42

# 1. Materialien und Methoden

## 1.1. Allgemeine Materialien

Zum Falten, Aufreinigen und Lagern von DNA-Origami-Nanostrukturen wurde ein 1x TAE-Puffer mit 20 mM Essigsäure, 1 mM EDTA und 50 mM Tris verwendet. Der 1x PBS Puffer für die Fluoreszenzmikroskopie-Aufnahmen enthielt 2,7 mM KCl, 137 mM NaCl, 1,8 mM $KH_2PO_4$und 10 mM $Na_2HPO_4$. Die für jede DNA-Origami-Nanostruktur individuell verwendeten $Mg^{2+,}$ Konzentrationen sind in Tabelle S1 gegeben.
Die Scaffold-Stränge wurden aus M13mp18 Bakteriophagen extrahiert. Unmodifizierte Staple-Stränge wurden von der Eurofins Genomics GmbH und Integrated Device Technology Inc. bezogen. Mit Farbstoffen modifizierte Oligonukleotide für DNA PAINT-Bildgebung oder Helligkeitsmarkierungen wurden von der Eurofins Genomics GmbH (Deutschland) bezogen.

Das Restriktionsenzym *Nb.BtsI* wurde zusammen mit dem 1X CutSmart® Puffer (20 mM Trisacetat, 10 mM Magnesiumacetat, 50 mM Kaliumacetat, 100 g/ml BSA) von New England Biolabs (NEB, USA) erworben.

Spezifische Materialien, die für die einzelnen Experimente verwendet werden, werden in den folgenden Abschnitten beschrieben.

## 1.2. DNA Origami Faltung

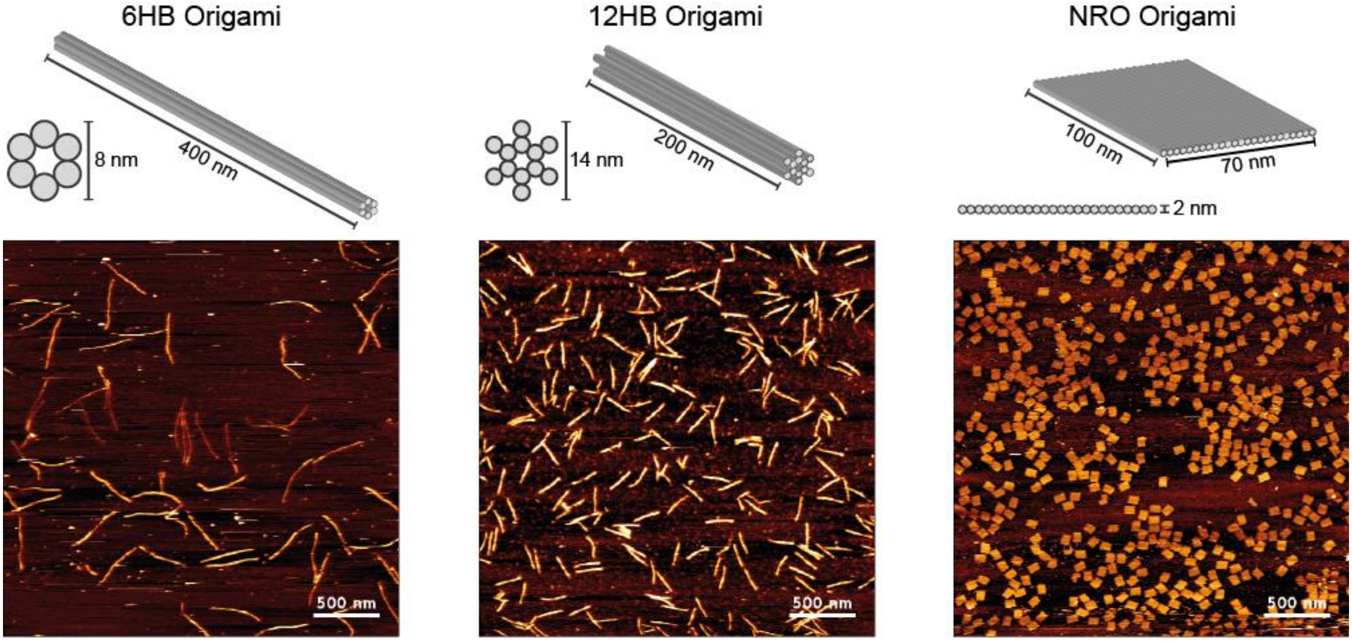


**Abbildung S1.** Schemata der in dieser Studie verwendeten DNA-Origami-Nanostrukturen. Das 6-Helix-Bundle (6HB) und das 12-Helix-Bundle (12HB) werden als 1D-Nanolineale für die moderne Fluoreszenzmikroskopie eingesetzt. Das neue rechteckige Origami (NRO) kann als 2D-Steckbrett für die Positionierung verschiedener Modifikationen verwendet werden. Entsprechende exemplarische AFM-Bilder, die nach dem Falten und Aufreinigen aufgenommen wurden, veranschaulichen die erfolgreiche Selbstassemblierung der designten Nanostrukturen.

Alle untersuchten DNA-Origami-Nanostrukturen (dargestellt in Abbildung S1) wurden mit den entsprechenden Scaffold-Strängen und Temperaturprogrammen in Tabelle S1 synthetisiert. Modifikationen der Nanolineale wurden mit caDNAno (Version 2.2.0) vorgenommen. Eine vollständige Liste der unmodifizierten Staple-Stränge und Sequenzen des 12HB DNA Origami[1] Tabelle S15 gegeben. Eine vollständige Liste der unmodifizierten Staple-Stränge und Sequenzen des NRO DNA Origami[2] ist in Tabelle S16 enthalten. Eine vollständige Liste der unmodifizierten Staple-Stränge und Sequenzen des 6HB DNA Origami[3] ist in Tabelle S17 gegeben.

**Tabelle S1.** Entsprechende Scaffold-Stränge, Faltungsprogramme, Faltungspuffer und $MgCl_2$-Konzentrationen, die zum Falten der in Abbildung S1 dargestellten DNA-Origami-Nanostrukturen 12HB, 6HB und NRO verwendet werden.

| DNA-Origami-Nanostrukture | Scaffold | Faltungsprogramm | Faltungspuffer | $MgCl_2$ Konzentration |
|---|---|---|---|---|
| 12HB | P8064 | 1 | 1xTAE | 16 mM |
| 6HB | P7560 | 1 | 1xTE | 14 mM |
| NRO | P7249 | 2 | 1xTE | 12,5 mM |

Zum Falten der DNA-Origami-Nanostrukturen wurden der Gerüst-Strang und die Staple-Stränge gemäß Tabelle S2 in dem entsprechenden 1x Faltungspuffer mit der entsprechenden $MgCl_2$-Konzentration gemäß Tabelle S1 gemischt. Unmodifizierte Staple-Stränge, die vollständig in die Origami-Struktur integriert sind, wurden in 10-fachem Überschuss in Bezug auf den Scaffold-Strang eingesetzt. Staplet-Sränge mit vorstehenden 5'-Enden, die als Anbindestellen für DNA PAINT- oder Markierungs-Experimente fungieren, wurden in 30-fachem Überschuss in Bezug auf den Scaffold-Strang verwendet. Biotin modifizierte Staple-Stränge, die zur Oberflächenimmobilisierung der DNA-Origami-Strukturen eingebaut wurden, wurden in einem 30-fachen Überschuss in Bezug auf den Scaffold-Strang verwendet.

**Tabelle S2** Eingesetzte Konzentrationen und relative Äquivalente des Scaffold-Strangs, der unmodifizierten Staple-Stränge und der modifizierten Staple-Stränge (z. B. Biotin modifizierte Staple-Stränge für Immobilisierung und mit DNA PAINT-Anbindestellen modifizierte Staple-Stränge für Superauflösungsmikroskopie),die in dieser Studie verwendet werden.

| Reagenz | Konzentration [nM] | Äquivalent |
|---|---|---|
| Scaffold-Strang | 13 | 1 |
| Unmod. Staple-Strang | 130 | 10 |
| DNA-PAINT-Staple-Strang | 390 | 30 |
| Biotin mod. Staple-Strang | 390 | 30 |

Das Falten des 12HB und des 6HB Origami wurde mit einer nichtlinearen Temperaturrampe über 16 Stunden realisiert (Tabelle S3)[4], während das NRO mit einer linearen Temperaturrampe über 75 min gefaltet wurde (Tabelle S4).

**Tabelle S3** Faltungsprogramm 1 zum Falten der 1D Nanolineale 12HB und 6HB.

| Temperatur (°C) | Zeit pro °C (min) | Temperatur (°C) | Zeit pro °C (min) |
|---|---|---|---|
| 65 | 2 | 44 | 75 |
| 64 – 61 | 3 | 43 | 60 |
| 60 – 59 | 15 | 42 | 45 |
| 58 | 30 | 41-39 | 30 |
| 57 | 45 | 38-37 | 15 |
| 56 | 60 | 36-30 | 8 |
| 55 | 75 | 29-25 | 2 |
| 54-45 | 90 | 4 | Lagerung |

Die Faltungsansätze hatten ein Gesamtvolumen von 100 µl mit Endkonzentrationen des Scaffold-Strangs, der unmodifizierten Staple-Stränge und der modifizierten Staple-Stränge (Biotin, DNA PAINT Anbindestellen) wie in Tabelle S2 angegeben.

**Tabelle S4** Faltungsprogramm 2 zum Falten der 2D NRO Nanolineale.

| Temperatur (°C) | Zeit pro °C (min) |
|---|---|
| 90 | 15 |
| 80 – 20 | 1 |
| 4 | Speicher |

### 1.3. Aufreinigung der DNA-Origami-Nanostrukturen

Die Aufreinigung gefalteter DNA-Origami-Nanostrukturen wurde entweder durch Gelelektrophorese oder durch Filterreinigung realisiert.

Für die Gelelektrophorese wurde eine 1 w% wässrige Lösung von Agarose in einem 1x TAE Puffer mit 12 mM $MgCl_2$ in einer Mikrowelle homogenisiert. Die Lösung wurde auf ca. 50°C abgekühlt und mit peqGreen (VWR International GmbH, Deutschland) eingefärbt und in einem Rahmen zur Verfestigung abgekühlt. Das erstarrte Gel wurde in eine Gelelektrophoresekammer mit 1x TAE Puffer mit 12 mM $MgCl_2$ überführt. Die gesamte Kammer wurde in ein Eisbett gelegt, um ein Schmelzen des Gels zu verhindern. Fünf Teile der Probenlösung wurden mit einem Teil des 6x BlueJuice-Ladefarbstoffs (ThermoFisher Scientific, USA) vermischt und in die Kammer geladen. Das Gel wurde für *ca.* 2 h unter 60 V gesetzt. Probenbänder wurden mit blauem Licht identifiziert und mit einem Skalpell ausgeschnitten. Die gereinigte DNA-Lösung wurde durch Ausdrücken der ausgeschnittenen Gelbänder extrahiert. Alternativ wurde die Probenreinigung durch Filtration mit Amicon Ultra Filtern (100 K, Merck, Deutschland) realisiert. Der Filter wurde zunächst mit Faltungspuffer für 7 Minuten bei 6000 g zentrifugiert. Die Probenlösung wurde dann in den Filter geladen und 15 Minuten lang bei 6000 g zentrifugiert. 500 µl Faltungspuffer wurden in den Filter geladen und 15 Minuten lang bei 6000 g zentrifugiert, was zweimal wiederholt wurde. Nach drei Waschschritten wurde der Filter umgedreht und in ein neues Auffangröhrchen gelegt. Die gereinigte Probe konnte durch Zentrifugation für 2 Minuten bei 1000 g aufgesammelt werden.

Konzentrationen der aufgereinigten Probenlösung wurden mittels UV/Vis-Spektroskopie gemessen (NanoDrop, Fischer Scientific, USA).

### 1.4. Oberflächenimmobilisierung von DNA-Origami-Nanolinealen

Für optische Mikroskopie Experimente wurde die DNA-Origami-Probe auf Nunc™ LabTek™ II Kammern (Thermo Fisher, USA) immobilisiert. Die Kammern wurden zunächst mit 400 µL einer 1 M KOH Lösung gereinigt und dreimal mit 1x PBS-Puffer gewaschen. Anschließend wurden die Oberflächen 30 Minuten lang mit 100 µl BSA-Biotin (0,5 mg/ml in PBS, Sigma Aldrich, USA) passiviert und dreimal mit 1x PBS-Puffer gewaschen. Die passivierten Oberflächen wurden 15 Minuten lang mit 100 µl Neutravidin (0,25 mg/ml in PBS, Sigma Aldrich, USA) inkubiert und dreimal mit 1x PBS-Puffer gewaschen. Die Probenlösung mit DNA-Origami mit mehreren mit Biotin modifizierten Staple-Strängen wurde in 1x PBS-Puffer mit 12,5 mM $MgCl_2$ auf ca. 50 pM verdünnt und 5 bis 15 Minuten in den Kammern inkubiert. Ausreichende Oberflächendichte wurde mit einem TIRF-Mikroskop kontrolliert.

### 1.5. Photostabilisierung von Fluoreszenzmarkierungen

Optische Messungen mit ATTO542 oder Cy5 als Imager-Fluorophoren wurden unter photostabilisierenden Bedingungen durchgeführt.[5] Ein 2,5-facher TAE-Puffer mit 1 % (wt/v) D-(+)-Glucose (Sigma Aldrich, USA), 165 Einheiten/ml Glucose-Oxidase (G2133, Sigma Aldrich, USA), 2170 Einheiten/ml-Katalase (C3155, Sigma Aldrich, USA), 1 mM Trolox und 2 M NaCl wurde verwendet.[6] Die Probenkammer mit immobilisierter DNA-Origami wurde vollständig mit dem Puffer befüllt und versiegelt, um das Lösen von Sauerstoff zu verhindern. Die ersten Messungen wurden mindestens 20 Minuten nach Einführung des Sauerstoffentfernungssystems durchgeführt, um eine Einstellung der Sauerstoffkonzentration in der Probenlösung abzuwarten.

### 1.6. Fluoreszenz-Aufnahmen von DNA-Origami-Helligkeitsstandards

Automatisierte Langzeitexperimente mit Helligkeitsstandards (12HB mit selbstregenerierender Markierung und 6HB mit durch *Nb.BtsI* abspaltbaren Markierungen) wurden auf einem kommerziellen Nanoimager S (ONI Ltd., UK) durchgeführt. Rote Anregung bei 638 nm wurde mit einem 1100 mW Laser, grüne Anregung bei 532 nm mit einem 1000 mW Laser realisiert. Das Mikroskop wurde auf TIRF-Beleuchtung eingestellt. Um die ersten Bilder der aufgenommenen Intensitätsspuren nicht durch das Photobleichen einzelner Nanolineale zu korrumpieren, wurde das Objektiv zunächst auf einem zufälligen Abschnitt der Glasoberfläche in die Probenebene fokussiert und der Autofokus aktiviert. Anschließend wurden die Anregungslaser abgeschaltet. Vor dem Starten von Zeitrafferaufnahmen wurde die Probe zu einem neuen Bereich verschoben, während der Fokus weiterhin über den Autofokus gehalten wurde. Die Aufnahme wurde gestartet, indem die Laser aktiviert und Bilder von 100 ms über ein benutzerdefiniertes Programm *(*z.B. ein Bild von 100 ms alle 10 min) aufgenommen wurden.

Innerhalb dieser Studie wurden Fluoreszenzhelligkeitsaufnahmen mit verschiedenen Imager-Strängen realisiert, aber mit der gleichen Imager-Strang Konzentration von 5 nM in 1x PBS Puffer mit 12,5 mM $MgCl_2$. Die Verarbeitung der Bildaufnahmen einschließlich Hintergrundsubtraktion und Datenanalyse wurde mit ImageJ 1.52n (Version 1.8.0_172) durchgeführt. Für eine Driftkorrektur wurde die lineare Stack-Ausrichtung mit dem SIFT-Plugin in ImageJ verwendet. Die Spot-Erkennung wurde mit einem benutzerdefinierten Algorithmus in ImageJ realisiert.

### 1.7. DNA PAINT-Aufnahmen

Superauflösungsaufnahmen mit der DNA PAINT-Technik wurden auf einem selbstgebauten Total-Internal Reflection-Fluoreszenzmikroskop (TIRF) auf Basis eines invertierten Mikroskops (IX71, Olymp) durchgeführt. Rote Anregung bei 644 nm wurde mit einem 150 mW Laser (iBeam smart, Toptica Photonics) spektral gefiltert mit einem Reinigungsfilter (Brightline HC 650/13, Semrock) realisiert. Zur gelben Anregung wurde ein zusätzlicher 560 nm/1 W Faserlaser (MPB Communications) ebenfalls mit einem Reingigungsfilter (Brightline HC 561/4, Semrock) spektral aufgereinigt. Der Laserstrahl wurde mit einem Drei-Farben-Strahlteiler (Chroma z476-488/568/647, AHF Analysetechnik) in das Mikroskop eingekoppelt und auf die rückseitige fokale Ebene eines Öl-Immersion-Objektivs (100×, NA= 1.4, UPlanSApo, Olympus) fokusiert, welches für TIRF-Beleuchtung ausgerichtet wurde. Das Fluoreszenzlicht wurde durch eine zusätzliche 1,6× optische Vergrößerungslinse und einen Emissionsfilter (ET 700/75, Chroma für rote Anregung oder ET 605/70m, Chroma für gelbe Anregung) geführt und anschließend auf eine wissenschaftliche Complementary-Symmetry Metal-Oxide-Semiconductor Kamera (sCMOS) zur Detektion fokusiert (pco.panda 4.2, 2048x2048 px, PCO AG). Die kalibrierte Pixelgröße betrug 42 nm/Pixel. Für die Datenerfassung wurde ein Pixelbinning von 2 verwendet, was zu einer effektiven Pixelgröße von 84 nm führte. Die Datenerfassung wurde mit der Software Micro-Manager 1.4 gesteuert. [7-8]

Die beiden verwendeten DNA PAINT-Imager-Stränge (8 nt lang) mit den entsprechenden Sequenzen und fluoreszierenden Markierungen am 3'-Ende sind in Tabelle S5 angegeben. Im Allgemeinen wurden DNA PAINT-Aufnahmen mit dem ATTO655-Imager-Strang und Imager-Konzentrationen von 1 bis 5 nM im 1x PBS-Puffer mit 12,5 mM $MgCl_2$ realisiert. Typischerweise wurde eine Belichtungszeit von 50 ms über eine Versuchszeit von 20 min mit 640 nm Laseranregung bei 30 mW verwendet. Für Zwei-Farben-Inkorporationsstudien zur Reparatur von geknickten 12 HB-Nanolinealen (Abbildung S7) wurden DNA PAINT-Imager-Stränge mit Cy3B-markierungen und Anregung mit dem 560 nm Laser verwendet.

Stabilisierungsstudien mit 12HB DNA PAINT Nanolinealen nach 2 Stunden Inkubation in einer 10% fetalen Rinderserum (FBS) Lösung wurden auf dem kommerziellen Nanoimager S (ONI Ltd., UK) mit einer 10 nM Lösung des 6 nt ATTO655 Imager-Strangs (Tabelle S5) in einem 1x PBS Puffer mit 12,5 mM $MgCl_2$ durchgeführt. Eine Rahmenzeit von 25 ms über eine Versuchszeit von 10 min wurde mit Anregung bei 638 nm auf 160 mW Ausgangsleistung verwendet.

**Tabelle S5** DNA PAINT-Imager-Stränge und fluoreszierende Markierungen, die für DNA-PAINT-Messungen verwendet wurden. Die DNA PAINT-Aufnahmen im roten Kanal wurden mit ATTO655-markierten Imager-Strängen realisiert, während die Bildgebung im grünen Kanal mit Cy3B-markierten Imager-Strängen durchgeführt wurde.

| Sequenz (5' bis 3') | Fluorophor-Markierung auf 3' |
|---|---|
| CGGGCATT | ATTO655 |
| CGGGCATT | Cy3B |
| CGGGCA | ATTO655 |

### 1.8. Auswertung der DNA PAINT Daten

Erhaltene DNA PAINT Rohdaten wurden mit dem Picasso-Softwarepaket ausgewertet.[9]

Die erhaltenen tiff-Filme wurden zunächst mit der "localize" Software von Picasso analysiert. Die zentralen Positionsinformationen der einzelnen Imager-Strang-Bindungsereignisse wurden mit einem minimalen Netzgradienten von 10000 und einer Boxgröße von 9 für Daten lokalisiert, die mit dem selbstgebauten TIRF-Setup aufgenommen wurden, und mit einem minimalen Netzgradienten von 2500 und einer Boxgröße von 5 für auf dem Nanoimager S erworbene Daten. Die gefitteten Lokalisierungen wurden mit der "Render"-Software von Picasso weiter analysiert. Die X-Y-Drift-Korrektur der Lokalisationen wurde mit der RCC-Driftkorrektur korrigiert. DNA-Origami-Nanolineale wurden mit der Render-Software ausgewählt und entsprechende mittlere Off-Times und Anzahl der Lokalisationen pro ausgewähltem Nanolineal zur weiteren Analyse extrahiert.

Für eine quantitative Distanzsanalyse wurden die Lokalisierungsereignisse der ausgewählten Nanolineale aus Render als csv-Datei exportiert zur weiteren Auswertung mit der Software GATTAnalysis der GATTAquant GmbH, Deutschland.

### 1.9. AFM-Aufnahmen mit JPK Nanowizard

Zur Überprüfung des korrekten Faltens der Origamistrukturen und zur Beobachtung struktureller Eigenschaften wurden AFM-Bilder aufgenommen. AFM-Scans in wässriger Lösung (AFM-Puffer = 40 mM Tris, 2 mM EDTA, 12,5 mM $Mg(OAc)_2$ x 4 $H_2O$) wurden auf einem NanoWizard® 3 ultra AFM (JPK Instruments AG) realisiert. Zur Probenimmobilisierung wurde eine frisch vorbereitete Glimmeroberfläche (Quality V1, Plano GmbH) mit einer 10 mM Lösung $NiCl_2$ für 3 Minuten oder alternativ mit 0,01 % (wt/v) Poly-L-Ornithin-Lösung inkubiert. Der Glimmer wurde dreimal mit ultrareinem Wasser gewaschen, um ungebundene $Ni^{2+}$-Ionen oder Poly-L-Ornithin wegzuwaschen, und mit Druckluft trocken geblasen. Die getrocknete Glimmeroberfläche wurde 3 Minuten lang mit 1 nM Probenlösung inkubiert und dreimal mit AFM-Puffer gewaschen. Die Messungen wurden im AC-Modus über eine Scanfläche von 3 x 3 µm mit einem Mikro Cantilever (-res = 110 kHZ, $k_{Feder}$ = 9 N/m, Olympus Corp.) durchgeführt.

### 1.10. Selbstregenerierende Helligkeitsmarkierung auf 12HB Helligkeitsstandard

Zur Erzeugung einer selbstregenerierenden Helligkeitsmarkierung auf einem 12HB-Helligkeitsstandard haben wir 5x20 Anbindestellen für die externe Markierung in die 12HB-Struktur designt (Abbildung 1 Haupttext). Die fünf Markierungspunkte waren gleichmäßig entlang der 200 nm Achse des 12HB mit 40 nm Zwischenabständen verteilt. Hierzu haben wir 5x20 unmodifizierte Staple-Stränge des 12HB in caDNAno mit den in Tabelle S6 angegebenen Anbindestelle-Staple-Strängen ausgetauscht. Die Anbindestelle-Staple-Stränge weisen einen über 20 nt langen Überhang an den 3'-Enden auf. Die verwendeten Imager-Stränge, die komplementär zu den Anbindestellen sind, sind in Tabelle S7 aufgeführt. Die Hybridisierung der 20 nt Imager-Stränge erzeugt bei Raumtemperatur thermodynamisch stabile und permanente Markierungen, die anfällig für Photobleichen sind. Die Verwendung kürzerer Imager-Stränge von nur 13 nt Länge führt zu einer transienten dynamischen Markierung, welche sich nach Photobleichereignissen erholen kann. Alle verwendeten Imager-Stränge wurden am 3'-Ende markiert, einschließlich des Oxazinfarbstoffs ATTO655 und des Rhodamin-Farbstoffs ATTO542.

Die Selbstregenerierung der Helligkeitsmarkierungen auf dem 12HB Helligkeitsstandard (Abbildung 1 Haupttext) wurde im Hinblick auf die Fähigkeit untersucht, die Markierungen nach Photoschäden, d. h. Photobleichen, wiederherzustellen. Nach initialen Helligkeitsmessungen (100 ms bei 3 mW Anregung bei 532 oder 640 nm) wurden die immobilisierten Helligkeitsstandards (3 min, 3 mW ohne Photostabilisierung, 20 mW mit Photostabilisierung) bis zu einem vollständigen Zusammenbruch der Helligkeitsfunktion geblichen. Die Regeneration der geblichenen Heligkeitsstandards wurde über die Zeit mittels Zeitraffer-Aufnahme (100 ms pro min mit 3 mW bei 532 oder 640 nm) gemessen.

**Tabelle S6** Modifizierte Staple-Stränge des 12HB Helligkeitsstandards. Sequenzen sind vom 5'- zum 3'-Ende angegeben. Die Anbindestellen-Staple-Stränge weisen eine über 20nt lange Andockstelle, rot markiert, an dem 3'-Ende auf. Zur Immobilisierung sind Staple-Stränge mit Biotin am 3'-Ende modifiziert. Die Zahlen für das 5'- Ende 3'-Ende der Staple-Stränge stellen die Helixnummer in der entsprechenden caDNAno-Datei dar. Die Zahl in Klammern stellt die Start- und Endposition des Staple-Strangs in der entsprechenden Helix dar.

| Anbindestellen-Staple-Stränge | 5'-Ende | 3'-Ende |
|---|---|---|
| CGAGTAACAACCTTTTACCAGTCATATCTCTCTACCACCTACATCAC | 0[118] | 0[96] |
| GCCTTACGCTGCGCGTAAAATTATTTTTTGACGCTCAATCATATCTCTCTACCACCTACATCAC | 0[179] | 11[188] |
| CGAGCACAGACTTCAAATACCTCAAAAGCTGCAATATATCTCTCTACCTACATCAC | 0[221] | 11[230] |
| AAAAATCTACGTGCGTTTTAATTATATATTTCCTCTACCACCTACATCAC | 0[244] | 0[222] |
| AAAACGAAAGAGGCTCATTATACATATATTTCCTCTACCACCTACATCAC | 0[286] | 0[264] |
| AGCGTATCACACACCACAGACCCGAGTTGCAGCAAGCGATATCTCTCTACCACCTACATCAC | 0[347] | 11[363] |
| GCGTCATACATGCCCTCATAGTTATATCTCTCTACCACCTACATCAC | 0[370] | 0[348] |
| TCACCGTCACCGGCGCACTCTATATTTCCTCTACCACCTACATCAC | 0[412] | 0[390] |
| AAAAAAGGCAGCCTTTACAATCTTACCAGTTTGATATTTCCTCTACCACCTACATCAC | 0[473] | 11[482] |
| TCCCATCCTAATGAGAATAACATATATCTCTCTACCACCTACATCAC | 0[496] | 0[474] |
| CTGAAAACCTGTTTATCAAACATGTAACGTCAAATATTTCCTCTACCACCTACATCAC | 0[515] | 11[524] |
| CGGTAGTACTCAATCCGCTGCTGGTCATGGTCATATATCTCTCTACCTACATCAC | 0[53] | 11[62] |
| TTAGGTTGGGTTATAGATAAGTCATATCTCTCTACCACCTACATCAC | 0[538] | 0[516] |
| TTCTGGAATAATCCTGATTTTGCCCGGCCGTAAATATCTCTCTACCTACTACATCAC | 0[599] | 11[608] |
| AAGATAAAACAGTTGGATTATACATATCTCTCTACCTACTACATCAC | 0[622] | 0[600] |
| CCGAACCCCCTAAAACATACCAGTTTAGAGCATATCTCTACCACCTACATCAC | 0[641] | 11[650] |
| GATTTTAGACAGGCATTAAAAATAATATTTCCTCTACCACCTACATCAC | 0[664] | 0[642] |
| CCGGAAGACGTACAGCGCCGCGATTACAATTCCATATCTCTCTACCTACATCAC | 0[95] | 11[104] |
| AGCAGTCGGGAAAACCTGTCATATATCTCTCTACCACCTACATCAC | 10[205] | 11[218] |
| AATAACGCGCGGGGAGAGGATATATCTCTCTACCTACTACATCAC | 10[247] | 11[260] |
| TCGTTCACCGCCTGGCCCTATCTCTCTACCACCTACATCAC | 10[331] | 11[344] |
| TACCTGGTTTGCCCCAGCAATATCTCTCTACCACCTACATCAC | 10[373] | 11[386] |
| AAAAGATAGGGTTGAGTGTATCTCTCTACCACCTACATCAC | 10[457] | 11[470] |
| CTATATTAAAGAACGTGGAATATCTCTCTACCACCTACATCAC | 10[499] | 11[512] |
| TGGCAAGTTTTTTGGGGTCATATCTCTCTACCACCTACATCAC | 10[583] | 11[596] |
| AACACCCTAAGGGAGCCCATATCTCTCTACCACCTACATCAC | 10[625] | 11[638] |
| GTATGTGAAATTGTTATCCATATATCTCTCTACCACCTACATCAC | 10[79] | 11[92] |
| TAAAGGATTGTATAAGCGCACAAACGACATTAAATGTGAGATATTTCCTCTACCACCTACATCAC | 11[135] | 0[119] |
| GTGCCTGCTATAACAGGGAGAGAGTTTCAAAGCGAACCAATATATTTCCTCTACCACCTACATCAC | 11[219] | 0[203] |
| CGGTTAACAAAGCTGCTGTACAACACaAGGACGTTGGGAAGATATATCTCTCTACCACCTACATCAC | 11[261] | 0[245] |
| GGCGACACACCTCTCTCTCTCTTTGTACTGTACCGTTCCAGTAAATATTTCCTCTACCACCTACATCAC | 11[387] | 0[371] |
| CTCCAATTTAGGCAGAGACAATCAATCAAGAAAAATAATAATAATATTTCCTCTACCACCTACATCAC | 11[513] | 0[497] |
| CCGATAATAAAAGGGACTTAACACGCGAACCAGCAGATATTTCCTCTACCACCTACATCAC | 11[639] | 0[623] |
| GCTCAAGTTGGGTAACGGGGAAAAATTTGTGAGAGATAATATATTTCCTCTACCACCTACATCAC | 11[93] | 0[77] |
| CCGAACTTTAATAAAAGCAAAGCGGATTATATATCTCTACCACCTACATCAC | 2[223] | 3[223] |
| TGTAGGGGATTTAGTAACACTTTTCATATTTCCTCTACCACCTACATCAC | 2[349] | 3[349] |
| ATTAAAAAGTGCGACGATTGGCCTTGATATCTCTACCACCTACATCAC | 2[391] | 3[391] |
| AAATAGGTAATTTACAAATAAGAAAACGAATATCTCTCTACCTACATCAC | 2[475] | 3[475] |
| ACGCGTCGGCTGTAAGACGACGACAATAATATCTCTACCACCTACATCAC | 2[517] | 3[517] |
| TCAATAATAAAGTGTATCATCATATTCCATATCTCTACCACCTACATCAC | 2[601] | 3[601] |
| GATAGTGCAACATGATATTTGAATGGATATTTCCTCTACCACCTACATCAC | 2[643] | 3[643] |
| GCCTTATACCCTGTAATACCAATTCTTGCGCTCATATATCTCTCTACCTACATCAC | 2[97] | 3[97] |
| GCTAAATCGGTTTGACTATTATAATATATTTCCTCTACCACCTACATCAC | 3[182] | 3[204] |
| GCATCAAAAAGAAAATTGGGATATATTTCCTCTACCACCTACATCAC | 3[224] | 3[246] |
| CTAAAGACTTTTAGGAACCCATGATATCTCTCTACCACCTACATCAC | 3[308] | 3[330] |

| | | |
|---|---|---|
| GTCACCAGTACAAGGTTGAGGCAATATTTCCTCTACCACCTACATCAC | 3[350] | 3[372] |
| TTTTTTTTTTAATAAAGTAATTCATATTTCCTCTACCACCTACATCAC | 3[476] | 3[498] |
| ATCAGCGGGGCTCTCTAGAGATATATTTCCTCTACCTACTACATCAC | 3[56] | 3[78] |
| TGATTATCAGATATACGTGGCACATATCTCTCTACCTACTACATCAC | 3[602] | 3[624] |
| GGCGCCCCGCCGAATCCTGAGAAGTGAGGCCGATTAAAGGATATATTTCCTCTACCACCTACATCAC | 3[667] | 0[665] |
| GGATAACCTCACAATTTTTTAATATCTCTCTACCACCTACATCAC | 3[98] | 3[120] |
| CCAGCCAAACTTCTGATTGCCGTTTTGGGTAAAGTTAAAAAAAACATATCTCTCTACCACCTACATCAC | 4[102] | 7[104] |
| GTTTGAGGGGACCTCATTTGCCGATATCTCTCTACCACCTACATCAC | 4[125] | 4[103] |
| GAGCTTAAGAGGTCCCAATTCCAATTCCATATAACAGTATATATTTCCTCTACCACCTACATCAC | 4[228] | 7[230] |
| GAAAGTTCAACAATCAGCTTGCTTAGCTTTAATTGTATCGATATTTCCTCTACCACCTACATCAC | 4[354] | 7[356] |
| CTATTTCGGAACGAGTGAGAATAATATATTTCCTCTACCTACTACATCAC | 4[377] | 4[355] |
| GCAGCACCGTAAGTGCCCGTATAATATATCTCTCTACCACCTACATCAC | 4[419] | 4[397] |
| TAAGCCAGAGAGCGAGAGAAACTCGATAGCCGAACAAAATATTTCCTCTACCACCTACATCAC | 4[480] | 7[482] |
| AGCAAGCCGTTTAAGAATTGAGTATATCTCTCTACCACCTACATCAC | 4[503] | 4[481] |
| ACCGCATTCCAACGGTATTCTAAGCGAGATATAGAAGGCTATATTTCCTCTACCACCTACATCAC | 4[522] | 7[524] |
| TGACCTAAATTTTTAAAAAGTATATTTCCTCTACCACCTAC | 4[545] | 4[523] |
| TCAGAGGTGTGTCGGCGAGAATGAGTGCACTCTGTGGTATATATTTCCTCTACCACCTACATCAC | 4[60] | 7[62] |
| ACAGTTTTTTTCAGATTTCAATTACCGTCGCAGAGGCGAATTATATATTTCCTCTACCACCTACATCAC | 4[606] | 7[608] |
| CAAATATCAAACCAGATGAATATATATCTCTCTACCTACTACATCAC | 4[629] | 4[607] |
| GCATCGAGCCAGATATCTTTAGGACCTGAGGAAGGTTATCATATTTCCTCTACCTACTACATCAC | 4[648] | 7[650] |
| TACTTCTTTGATAAAAACTAAAATATTTCCTCTACCACCTACATCAC | 4[671] | 4[649] |
| CATAATATTCCGTAATGGGATCCGTGCATCTGCCAATATTTCCTCTACCACCTACATCAC | 5[119] | 4[126] |
| TGTAAATCATGCTCCTTGATAATTGCTGAATATATATATCTCTCTACCACCTACATCAC | 5[203] | 4[210] |
| CGCCTGACGGTAGAAAGATTCTAATGCAGATACATATATATCTCTCTACCACCTACATCAC | 5[245] | 4[252] |
| GCGAAAGACGCAAAGCCGCGGGAACATATTTCCTCTACCACCTACATCAC | 5[287] | 4[294] |
| TTCATTTTCTGCTAAAAACTGAACAACTAAAAGGAATATTTCCTCTACCACCTACATCAC | 5[329] | 4[336] |
| ATCAGAGCCTTTAACGGGGTCTTAATGCCCCCTGCATATATCTCTCTACCACCTACATCAC | 5[371] | 4[378] |
| TTGAGAATATCTCTCTCTCTCTCTACTCATCTCGAGAACAATATTTCCTCTACCACCTACATCAC | 5[497] | 4[504] |
| ATACCCTTCGTGCCACGCTGAACCTTGCTGAACCTATCTCTCTACCTACTACATCAC | 5[623] | 4[630] |
| AACGTTGTAGAAAAGCGGATAGTTGGGCGGTTGTATATATTTCCTCTACCACCTACATCAC | 5[77] | 4[84] |
| GAAAGATCGCACTCCAGCCAGCTATATCTCTCTACCACCTACATCAC | 7[105] | 7[127] |
| TTCCGAATTGTAAAAGTGTCGCCAGCATCGGTGCGGGCCTATATTTCCTCTACCACCTACATCAC | 7[128] | 8[112] |
| GGCTAAAGTACGGTCTGGAAGATATATTTCCTCTACCACCTACATCAC | 7[189] | 7[211] |
| TTTCACGAGAATGACCATTTTCATTTGGTCAATAACCTGTATATATTTCCTCTACCACCTACATCAC | 7[212] | 8[196] |
| TGCAACACTATCATAACCCTCGTATATATCTCTCTACCACCTACATCAC | 7[231] | 7[253] |
| TTACCAATAGGCTTGCAGTGCGGAAGTTTAGACTGGATAATATATTTCCTCTACCACCTACATCAC | 7[254] | 8[238] |
| AATCCAAAAAAAGCTCCAAAAATATATCTCTCTACCACCTACATCAC | 7[315] | 7[337] |
| GTGTATTAAGAGGCTGAGACTCCATATATCTCTCTACCACCTACATCAC | 7[357] | 7[379] |
| TCACACAGAACCACCACTACTCAGGTAGCCCGGAATAGGATATTTCCTCTACCACCTACATCAC | 7[380] | 8[364] |
| TTGAAGCCCTTTAAGAAAAGTATATCTCTCTACCACCTACATCAC | 7[441] | 7[463] |
| GTTTACCGCGCCCAATAGCAAGCATATTTCCTCTACCACCTACTACCAC | 7[483] | 7[505] |
| AAATCAGCCAGTAATAACACTATTTTTGAAGCCTTAAATCATATCTCTACCACCTACATCAC | 7[506] | 8[490] |
| ATTTGGCAAATCAACAGTTGAAAATATTTCCTCTACCACCTACATCAC | 7[609] | 7[631] |
| GGAATAACAGAGATAGACATACAAACTTGAGGATTTAGAAATATTTCCTCTACCACCTACATCAC | 7[632] | 8[616] |
| GCCCGCACAGGCGGCCTTTAGTGATATATCTCTCTACCTACTACTAC | 7[674] | 8[658] |
| ATGAATCCCAGTCACGATCGAACGTGCCGGCCAGAGCACAATATATCTCTCTACCACCTACATCAC | 7[86] | 8[70] |
| CTTTTTTTGTCTCTCGTCGCTGGCATATATTTCCTCTACCACCTACATCAC | 8[111] | 8[89] |
| TCAGGCTGCGCAACTGTTGGGAAATATCTCTCTACCACCTACATCAC | 8[153] | 8[131] |
| GCTTGACCATTAGATACATTTCGATATATTTCCTCTACCACCTACTACCAC | 8[237] | 8[215] |
| ATTTTGCCAGAGGGTAATAGTATATATTTCCTCTACCTACTACATCAC | 8[279] | 8[257] |

| | | |
|---|---|---|
| TGAACAGCTTGATACCGATAGTTATATATCTCTCTACCACCTACATCAC | 8[363] | 8[341] |
| GGTGCCGTCGAGAGGGTTGATATATATTTCCTCTACCACCTACATCAC | 8[405] | 8[383] |
| AAAACGGAATACCCAAAAGAACTATATTTCCTCTACCACCTACATCAC | 8[489] | 8[467] |
| AACGAACCTCCCGACTTGCGGGAATATTTCCTCTACCTACTAC | 8[531] | 8[509] |
| GTATTAGAGCCGTCAATAGATAAATATTTCCTCTACCTACTACATCAC | 8[657] | 8[635] |
| **Biotin modifizierte Staple-Stränge** | **5'-Ende** | **3'-Ende** |
| AACGCCAAAAGGCGGATGGCTTA | 4[251] | 4[229] |
| AAGAAACAATGACCGGAAACGTC | 4[461] | 4[439] |
| GTACATCGACATCGTTAACGGCA | 4[83] | 4[61] |
| ATACCACCATCAGTGAGGCCAAACCGTTGTAGCAA | 5[665] | 4[672] |

**Tabelle S7** Imager-Stränge zur externen Markierung des 12HB Helligkeitsstandards. Alle Imager-Stränge sind am 3'-Ende mit verschiedenen Fluorophoren modifiziert. Anbinde-Sequenzen der Imager-Stränge sind rot markiert.

| Sequenz (5' bis 3') | Fluorophor-Markierung auf 3' | Andocklänge [nt] |
|---|---|---|
| GGTGGTAGAGGAATT | ATTO655 | 13 |
| GGTGGTAGAGGAATT | ATTO542 | 13 |
| GTGATGTAGGTGGTAGAGGAA | ATTO655 | 20 |

### 1.11. Reparatur der geknickten 12HB Nanolineale

Um einen strukturellen Schaden in einem 12HB-Nanolineal nachzuahmen, haben wir bei der Faltung der DNA-Origami 9 Staple-Stränge (Tabelle S9) im zentralen Bereich der linearen Nanostruktur ausgelassen, was zu einem einsträngigen Scaffold-Strang über alle 12 Helices führte (Abbildung 2 Haupttext). Um das resultierende 12HB-Nanolineal mittels DNA PAINT zu untersuchen, tauschten wir 60 Staple-Stränge mit DNA PAINT Staple-Strängen aus, die in Tabelle S8 angegeben sind. Die verwendeten DNA PAINT Staple-Stränge weisen eine Andock-Sequenz auf, die zu den 8 nt DNA PAINT-Imager-Strängen in Tabelle S5 komplementär ist. Die Anbindestellen wurden gleichmäßig auf zwei Seiten entlang der 200 nm Achse des 12HB in ca. 7 nm Entfernungen verteilt, um die Gesamtkontur der 12HB Nanolineale zu visualisieren.
In einem ersten Faltungsvorgang mit dem Faltungsprogramm 1 in Tabelle S3 wurde das 12HB gefaltet, wobei die neun InStaple-Stränge in Tabelle S9 ausgelassen wurden. Nach der Aufreinigung wurde ein Teil der Probenlösung mit AFM und DNA PAINT untersucht. Der andere Teil der Probenlösung wurde in einem zweiten Schritt mit einer Mischung der 9 fehlenden Staple-Stränge in 300x Überschuss mit dem Faltungsprogramm 1 gefaltet, mit einer Startemparatur von T=50°C um die Inkoproration der fehlenden Staple-Stränge zu beschleunigen, aber die bereits gefalteten 12HB Nanolineale nicht gleichzeitig zu zerstören. Nach Aufreinigung konnte die reparierte Probenlösung mit AFM und DNA PAINT analysiert werden. Zusätzlich wurden Inkorporationsstudien durchgeführt. Hierzu wurden drei der neun später zugesetzten Staple-Stränge mit Cy5 markiert. Durch Weitfeld-Aufnamen eines Sichtfeldes in rotem Kanal (eingearbeitete Cy5-Staple-Stränge) und anschließender DNA-PAINT-Aufnahme desselben Bereichs im gelben Kanal (Cy3B-Imager-Strang) konnte die quantitative Inkorporation der fehlenden Staple-Stränge untersucht werden. Für DNA PAINT Experimente wurde eine 1 nM Lösung der 8 nt ATTO655 oder Cy3B Imager-Stränge verwendet.

**Tabelle S8** DNA PAINT Staple-Stränge zur Visualisierung der Kontur des geknickten 12HB Nanolineals mit 9 fehlenden Staple-Strängen. Alle DNA PAINT Staple-Stränge weisen an ihren 3'-Enden die 8 nt Andock-Sequenz für die 8 nt Imager-Stränge in Tabelle S5 auf. Zur Immobilisierung sind Staple-Stränge mit Biotin am 3'-Ende modifiziert. Die Zahlen für das 5'- Ende 3'-Ende der Staple-Stränge stellen die Helixnummer in der entsprechenden caDNAno-Datei dar. Die Zahl in Klammern stellt die Start- und Endposition des Staple-Strangs in der entsprechenden Helix dar.

| DNA PAINT Staple-Stränge | 5'-Ende | 3'-Ende |
|---|---|---|
| ATCAGCGGGGCTCTTTCAGAGTTAAATGCCCG | 3[56] | 3[78] |
| TTTTTTTTTTAATAAAGTAATTCTTAAATGCCCG | 3[476] | 3[498] |
| GTCACCAGTACAAGGTTGAGGCATTAAATGCCCG | 3[350] | 3[372] |
| AACTTTAATCATGGGTAGCAACGTTAAATGCCCG | 3[266] | 3[288] |
| TTCCATTGACCCAAAGAGGCTTTGAGGATTAAATGCCCG | 2[307] | 3[307] |
| ACGCGTGGCTGTAAGACGACGACAATATTAAATGCCCG | 2[517] | 3[517] |
| TTCGCCATAAACTCTGGAGGTGTCCAGCTTAAATGCCCG | 2[55] | 3[55] |
| ATTAAAATAAGTGCGACGATTGGCCTTGTTAAATGCCCG | 2[391] | 3[391] |
| GCTAAATCGGTTTGACTATTATATTAAATGCCCG | 3[182] | 3[204] |
| TATGCATTACAGAGGATGGTTTAATTTCTTAAATGCCCG | 2[265] | 3[265] |

| | | |
|---|---|---|
| AAATAGGTAATTTACAAATAAGAAAACGATTAAATGCCCG | 2[475] | 3[475] |
| CCGAACTTTAATAAACAAAGCGGATTTTAAATGCCCG | 2[223] | 3[223] |
| AGGGACAAAATCTTCGCGCCAAAGACTTAAATGCCCG | 2[433] | 3[433] |
| CTAAAGACTTTTAGGAACCCATGTTAAATGCCCG | 3[308] | 3[330] |
| AAGACGCTGAGACCAGAAGGAGCTTAAATGCCCG | 3[560] | 3[582] |
| AACAACATGTTCATCCGAGAAAATTAAATGCCCG | 3[518] | 3[540] |
| AAAAGGGCGACAATTATTTATCCTTAAATGCCCG | 3[434] | 3[456] |
| TGTAGGGGATTTAGTAACGAGTTTCTTAAATGCCCG | 2[349] | 3[349] |
| ATATTCACAAACAAATTCATATGTTAAATGCCCG | 3[392] | 3[414] |
| GAATTATCCAATAACGATAGCTTAGATTTTAAATGCCCG | 2[559] | 3[559] |
| TTATGGCCTGAGCACCTCAGAGCATAAATTAAATGCCCG | 2[181] | 3[181] |
| GCATCAAAAAGAAATTGGGTTAAATGCCCG | 3[224] | 3[246] |
| TTCGCGGATTGATTGCTCATTTTTTAACTTAAATGCCCG | 2[139] | 3[139] |
| GATAGTGCAACATGATATTTGAATGGTTAAATGCCCG | 2[643] | 3[643] |
| GGATAACCTCACAATTTTTTATTAAATGCCCG | 3[98] | 3[120] |
| TCAATAATAAAGTGTCATCATATTCCTTAAATGCCCG | 2[601] | 3[601] |
| CAATAGGAACGCAAATTAAGCAATTAAATGCCCG | 3[140] | 3[162] |
| GCGAAAGACGCAAAGGCCACGGGAACTTAAATGCCCG | 2[97] | 3[97] |
| TGATTATCAGATATACGTGGCACTTAAATGCCCG | 3[602] | 3[624] |
| CTATTAGTCTTTCGCCGCTACAGTTAAATGCCCG | 3[644] | 3[666] |
| AAGCACAGAGCCTAATTATTGTTAGCGATTAAGACTCCTTTTAAATGCCCG | 7[464] | 8[448] |
| TGAACAGCTTGATACCGATAGTTTTAAATGCCCG | 8[363] | 8[341] |
| AAATCAGCCAGTAATAACACTTTTTGAAGCCTTAAATCTTAAATGCCCG | 7[506] | 8[490] |
| GGTGCCGTCGAGAGGGGTGATATTTAAATGCCCG | 8[405] | 8[383] |
| AACGAACCTCCCGACTTGCGGGATTAAATGCCCG | 8[531] | 8[509] |
| CCGAACGGTGTACAGAGGCGTTAAATGCCCG | 8[321] | 8[299] |
| GGAGCAGCCACCACCCGCGCATAACGACAATGACAACAATTAAATGCCCG | 7[338] | 8[322] |
| TTTCACGAGAATGACCATTTTCATTTGGTCAATAACCTGTTTAAATGCCCG | 7[212] | 8[196] |
| TCGGTCATACCGGGGGGTTCTGCTTAAATGCCCG | 8[69] | 8[47] |
| TTACCAATAGGCTTGCAGTGCGGAAGTTTAGACTGGATATTAAATGCCCG | 7[254] | 8[238] |
| AAAACGGAATACCCAAAAGAACTTTAAATGCCCG | 8[489] | 8[467] |
| AGACGTCGTCACCCTCAGCTTGACGCTGGCTGACCTTCTTAAATGCCCG | 7[296] | 8[280] |
| AAATGCGTTATACAAATTCTTACTTAAATGCCCG | 8[573] | 8[551] |
| TAAGATCTGTAAATCGTTGTTAATTGTAAAGCCAACGCTCTTAAATGCCCG | 7[548] | 8[532] |
| ATGAATCCCAGTCACGATCGAACGTGCCGGCCAGAGCACATTAAATGCCCG | 7[86] | 8[70] |
| ATTCTTTTCATAATCAAAATCACTTAAATGCCCG | 8[447] | 8[425] |
| AATCGTTGAGTAACATTGGAATTACCTAATTACATTTAACTTAAATGCCCG | 7[590] | 8[574] |
| ATTTTGCCAGAGGGTAATAGTTTAAATGCCCG | 8[279] | 8[257] |
| AGCGCCACCACGGAATACGCCTCAGACCAGAGCCACCACCTTAAATGCCCG | 7[422] | 8[406] |
| TCACACAGAACCACCACTACTCAGGTAGCCCGGAATAGGTTAAATGCCCG | 7[380] | 8[364] |
| CTTTTTTTGTCTCTTCGCTGGCTTAAATGCCCG | 8[111] | 8[89] |
| GTTGAAACAAACATCAAAAAACTTAAATGCCCG | 8[615] | 8[593] |
| GCTTGACCATTAGATACATTTCGTTAAATGCCCG | 8[237] | 8[215] |
| TTAACAAGAGAATCGATGAACGGTTAAATGCCCG | 8[195] | 8[173] |
| GTATTAGAGCCGTCAATAGATAATTAAATGCCCG | 8[657] | 8[635] |
| TCAGGCTGCGCAACTGTTGGGAATTAAATGCCCG | 8[153] | 8[131] |
| TTTTTATCCAATAAATCTCTACCCCGGTAAAATAGCATGTTAAATGCCCG | 7[170] | 8[154] |
| GGAATAACAGAGATAGACATACAAACTTGAGGATTTAGAATTAAATGCCCG | 7[632] | 8[616] |
| TTCCGAATTGTAAAAGTGTCGCCAGCGCGGGCGGGCCTTTAAATGCCCG | 7[128] | 8[112] |
| GCCTTACGCTGCGCGTAAAATTATTTTTTGACGCTCAATCTTAAATGCCCG | 7[674] | 8[658] |

| Biotin modifizierte Staple-Stränge | 5'-Ende | 3'-Ende |
|---|---|---|
| AACGCCAAAAGGCGGATGGCTTA | 4[251] | 4[229] |
| AAGAAACAATGACCGGAAACGTC | 4[461] | 4[439] |
| GTACATCGACATCGTTAACGGCA | 4[83] | 4[61] |
| ATACCACCATCAGTGAGGCCAAACCGTTGTAGCAA | 5[665] | 4[672] |

**Tabelle S9** Ausgelassene Staple-Stränge, um einen strukturellen Schaden in einem 12HB-Nanolineal nachzuahmen. Für Inkorporationsstudien wurden drei Staple-Stränge mit Cy5 am 5'-Ende markiert (rot markiert). Die Zahlen für das 5'- Ende 3'-Ende der Staple-Stränge stellen die Helixnummer in der entsprechenden caDNAno-Datei dar. Die Zahl in Klammern stellt die Start- und Endposition des Staple-Strangs in der entsprechenden Helix dar.

| Fehlende Staple-Stränge | 5'-Ende | 3'-Ende |
|---|---|---|
| (Cy5)- GGTGCCGTCGAGAGGTGAT | 8[405] | 8[383] |
| TACCTGGTTTGCCCCAGCA | 10[373] | 11[386] |
| AAGTAAGAGCCGCCAGTACCAGGCGG | 8[382] | 9[398] |
| (Cy5)- ATTAAAATAAGTGCGACGATTGGCCTTG | 2[391] | 3[391] |
| ATCAGAGCCTATACGGGGTCTTAATGCCCCCTGC | 5[371] | 4[378] |
| GGCGACACACCCTCTCAGGTTACTGTACGTTCCAGTAAA | 11[387] | 0[371] |
| TCACACAGAACCACCACTACTCAGGTAGCCCGGAATAGG | 7[380] | 8[364] |
| AACAGAGTGTGTGGGTGTGTGCTCACAGAAGGATTAGGAT | 4[396] | 7[398] |
| (Cy5)- GAATTGTAGCCAGAATGGATCAGAGCAAATCCT | 0[389] | 11[398] |

### 1.12. Staple-Strang-Austausch vom Doppel-Punkt zum Triple-Punkt im NRO-Nanolineal

Um den möglichen Austausch von Staple-Strängen innerhalb eines DNA-Origamis mit Staple-Strängen aus einer Lösung zu untersuchen, haben wir ein NRO DNA PAINT Nanolineal mit zunächst zwei DNA PAINT-Markierungen in einem Abstand von 40 nm entworfen, die jeweils aus 3 Anbindestellen bestehen. Hierzu haben wir die 6 unmodifizierten Staple-Stränge mit den DNA PAINT Staple-Strängen in Tabelle S10 ausgetauscht. Nach dem Falten und Aufreinigen konnte das Doppelpunkt-NRO mit den DNA PAINT-Imager-Strängen aus Tabelle S5 aufgenommen werden.
Für die Untersuchung des Austauschs von Staple-Strängen innerhalb des DNA-Origami mit Staple-Stränge aus einer Lösung haben wir das Doppelpunkt-NRO mit einer Mischung aus drei DNA PAINT-Staple-Strängen inkubiert, um einen dritten Markierungspunkt für DNA PAINT in 40 und 70 nm Abstand zu den ersten beiden Markierungen zu bilden, was zu einem Triple-Punkt führt. Um die Inkoporation zu beschleunigen, haben wir einen 300-fachen Überschuss der invasiven DNA PAINT Staple-Stränge in Bezug auf die Doppelpunkt-NROs im entsprechenden Faltungspuffer (Tabelle S1) verwendet und die Probenlösung ab T= 50°C dem NRO-Faltungsprogramm (Tabelle S4) unterzogen, um gleichzeitig eine Denaturierung des bereits gefalteten Doppelpunkt-NRO zu verhindern. Um auch teilweise beschädigte Staple-Stränge nachzuahmen, haben wir das anfängliche Doppelpunkt-NRO mit kürzeren unmodifizierten Staple-Strängen gefaltet, die durch die invasiven DNA PAINT Staple-Stränge verdrängt werden sollen. Wir haben 3 anfängliche Staple-Stränge, die in einem Fall 4 nt zu kurz sind, in einem zweiten Fall 8 nt zu kurz, verwendet, was zu einem 4 nt bzw. 8 nt Toehold auf dem Scaffold-Strang führt. Für alle drei Fälle von anfänglichen Staple-Strängen (0, 4, 8 nt Scaffold-Toeholds) wurden die anfänglichen Doppelpunkt-NROs mit den oben erwähnten invasiven DNA PAINT-Staple-Strängen inkubiert.

Um die Inkorporation der DNA PAINT Staple-Stränge zu untersuchen, wurden DNA PAINT Experimente mit einer 5 nM Lösung der 8 nt ATTO655 Imager Stränge durchgeführt.

**Tabelle S10** DNA PAINT Staple-Stränge für ein Doppelpunkt NRO Nanolineal (2x3 Docking-Sites) und invasive Staple-Stränge zur Bildung eines dritten Markierungspunkts. Die drei anfänglichen Staple-Stränge, die durch invasive Staple-Stränge verdrängt werden sollen, weisen 0 bis 8 nt verkürzte Sequenzen auf, um Toeholds im Scaffold-Strang zu erzeugen (ausgelassene Sequenzen, die in grün und blau hervorgehoben sind). Alle DNA PAINT Staple-Stränge weisen an ihren 3'-Enden die 8 nt Andock-Sequenz für die 8 nt-Imager-Stränge in Tabelle S5 auf (rot hervorgehoben). Zur Immobilisierung sind Staple-Stränge mit Biotin am 3'-Ende modifiziert. Die Zahlen für das 5'- Ende 3'-Ende der Staple-Stränge stellen die Helixnummer in der entsprechenden caDNAno-Datei dar. Die Zahl in Klammern stellt die Start- und Endposition des Staple-Strangs in der entsprechenden Helix dar.

| Doppelpunkt-DNA-PAINT-Staple-Stränge | 5'-Ende | 3'-Ende |
|---|---|---|
| TCAATATCGAACCTCAAATCAATTCCGAAATTAAATGCCCG | 21[160] | 22[144] |
| TCGGCAAATCCTGTTGATGGTGGACCCTCAATTAAATGCCCG | 22[143] | 21[159] |
| AACGTGGCGAGAAAGAGGGAAACCAGTAATTAAATGCCCG | 23[128] | 23[159] |
| GTTTATTTTGTCACAATCTTACCGAAGCCCTATAATCATTAAATGCCCG | 7[248] | 9[255] |
| GAGAGATAGAGCGTCTTTCCAGAGGTTTTGAATTAAATGCCCG | 9[256] | 11[255] |
| GCCTTAAACCAATCAATAATCGGCACGCGCCTTTAAATGCCCG | 11[256] | 13[255] |
| **Triple-Punkt-Stapel-Stränge** | **5'-Ende** | **3'-Ende** |

| | | |
|---|---|---|
| ACAAACGGAAAAACCCCAAAAAAACACTGGAGCA-TTAAACCCG | 16[47] | 14[48] |
| AACAAGAGGGATAAAAATTTTTAGCATAAAGC-TTAAACCCG | 14[47] | 12[48] |
| TAAATCGGGATTCCCAATTCTGCGATATAATG-TTAAAATGCCCG | 12[47] | 10[48] |
| **Biotin modifizierte Staple-Stränge** | **5'-Ende** | **3'-Ende** |
| CGGATTCTGACGACAGTATCGGCCAAGGCGATTAAGTT | 16[63] | 18[56] |
| ATAAGGGAACCGGATATTCATTACGTCAGGACGTTGGGAA | 4[63] | 6[56] |
| GAAACGATAGAAGGCTTATCTGGTCTCATCGAGAACAAGC | 10[191] | 12[184] |
| TAGAGAGTTATTTTCATTTGGATAGTAGTAGCATTA | 10[127] | 12[120] |
| AGCCACCACTGTAGCGCGTTTTCAAGGGGAGAAGAAAA | 4[255] | 6[248] |
| GAGAAGAGATACTTGCTGTGGGGAGAAACAATAA | 16[255] | 18[248] |

### 1.13. Selbstheilung von 12HB Nanolinealen unter degradierenden Bedingungen

Für die Untersuchung von Selbstheilungsprozessen eines DNA-Origami-Nanolineals unter degradierenden Bedingungen haben wir ein 12HB Drei-Punkt-Nanolineal entworfen, welches für DNA-PAINT-Experimente mit den 6 oder 8 nt Imager-Strängen, die in Tabelle S5 gegeben sind, geeignet ist. Wir haben drei Markierungspunkte auf dem 12HB Origami mit 107 und 70 nm Abständen entworfen, indem wir 3x10 Staple-Stränge durch die entsprechenden DNA PAINT Staple-Stränge in Tabelle S11 ausgetauscht haben. Die DNA PAINT Staple-Stränge weisen eine 10 nt lange Andock-Sequenz für DNA PAINT-Experimente auf, die komplementär zu den verwendeten Imager-Strängen ist.

Selbstheilungsstudien unter degradierenden Bedingungen wurden durch Inkubation der immobilisierten DNA PAINT Nanolineale im Faltungspuffer des 12HB (Tabelle S1) mit zusätzlich 0,2% (vol) oder 10% (vol) fetalem Rinderserum (FBS) von ThermoFisher Scientific durchgeführt. Um die stabilisierende Wirkung zusätzlicher zufälliger Oligonukleotide zu testen, haben wir eine Mischung der unmodifizierten Staple-Strängen des 6HB Origami (Tabelle S17) hinzugefügt, welche keine signifikante Überlappung zum Scaffold-Strang des 12HB Origami aufweisen. Hierzu haben wir die Mischung der 146 verschiedenen Oligonukleotiden in den Inkubationspuffer in einer Gesamtkonzentration von 5 µM, 500 nM oder 50 nM (d.h. 34,2 nM, 3,2 nM oder 0,3 nM pro Strang) hinzugefügt. Um die selbstheilende Wirkung durch Staple-Stränge zu testen, haben wir in einem weiteren Inkubationsexperiment eine Mischung der 222 unmodifizierten 12HB Staple-Stränge zur FBS-Lösung hinzugefügt. Die unmodifizierten 12HB Staple-Stränge (Tabelle S15) wurden in einer Gesamtkonzentration von 5 µm, 500 nM oder 50 nM (d.h. 22,5 nM, 2,2 nM oder 0,2 nM pro Strang) zugegeben. Während der Inkubation mit dem jeweiligen FBS-Puffer wurden die Proben bei Raumtemperatur gelagert. Vor jedem DNA PAINT-Experiment wurde die Inkubationslösung von der Probe genommen und die Oberfläche dreimal mit dem Imaging-Puffer (1x PBS, 12,5 mM $MgCl_2$) gewaschen. Nach dem DNA-PAINT_Experiment wurde der Imaging-Puffer dreimal weggewaschent und eine neue Inkubationslösung aufgebracht. Für Experimente mit 0,2% FBS wurde in DNA-PAINT-Experimenten eine 2 nM-Lösung des 8 nt ATTO655 Imager-Strangs verwendet. Für Studien mit höheren Schadensraten in 10% FBS wurde eine 10 nM-Lösung des 6 nt ATTO655 Bildstrangs in DNA-PAINT-Experimenten verwendet.

**Tabelle S11** Sequenzen der Staple-Stränge, die zur Erzeugung einer Dreipunkt-Markierung auf dem 12HB Nanolineal verwendet wurden. Alle DNA PAINT Staple-Stränge weisen an ihren 3'-Enden die 8 nt Andock-Sequenz für die 8 nt-Imager-Stränge in Tabelle S5 (rot hervorgehoben). Zur Immobilisierung sind Staple-Stränge mit Biotin am 3'-Ende modifiziert. Die Zahlen für das 5'- Ende 3'-Ende der Staple-Stränge stellen die Helixnummer in der entsprechenden caDNAno-Datei dar. Die Zahl in Klammern stellt die Start- und Endposition des Staple-Strangs in der entsprechenden Helix dar.

| Dreipunk-DNAPAINT-Staple-Stränge | 5'-Ende | 3'-Ende |
|---|---|---|
| GTATGTGAAATTGTTATCCTTAAATGCCCG | 10[79] | 11[92] |
| CTTTTTTTGTCTCTTCGCTGGCTTAAATGCCCG | 8[111] | 8[89] |
| CCAGCCAACTTCTGATTGCCGTTTTGGGTAAAGTTAAACTTAAATGCCCG | 4[102] | 7[104] |
| GCCCGCACAGGCGGCCTTTAGTGTTAAATGCCCG | 7[63] | 7[85] |
| CCGGAAGACGTACAGCGCCGCGATTACAATTCCTTAAATGCCCG | 0[95] | 11[104] |
| TAAAGGATTGTAAGCGCACAAACGACATTAAATGTGAGTTAAATGCCCG | 11[135] | 0[119] |
| GGATAACCTCACAATTTTTTATTAAATGCCCG | 3[98] | 3[120] |
| GCGAAAGACGCAAAGGCCACGGGAACTTAAATGCCCG | 2[97] | 3[97] |
| TTCCGAATTGTAAAAGTGTCGCCAGCGCGGGCGGGCCTTTAAATGCCCG | 7[128] | 8[112] |
| CGAGTAACAACCTTTTACCAGTCTTAAATGCCCG | 0[118] | 0[96] |
| GATGTTTTTTTTTCACCATTAAATGCCCG | 10[289] | 11[302] |
| CCGAACGGTGTACAGAGGCGTTAAATGCCCG | 8[321] | 8[299] |
| GGAGCAGCCACCACCCGCGCATAACGACAATGACAACAATTAAATGCCCG | 7[338] | 8[322] |

| | | |
|---|---|---|
| TTCCATTGACCCAAAGAGGCTTTGAGGATTAAATGCCCG | 2[307] | 3[307] |
| CTAAAGACTTTTAGGAACCCATGTTAAATGCCCG | 3[308] | 3[330] |
| ACTACCTTTAAACGGGTAACAGGGAGACGGGCATTAAATGCCCG | 0[305] | 11[314] |
| GAGAGCCTGAGACGCATTTTCTGTAACGATCTAAAGTTTTAAATGCCCG | 11[345] | 0[329] |
| ATTTGCCAAGCGGAACTGACCAACGAGTCAATCATAAGGGTTAAATGCCCG | 4[312] | 7[314] |
| TTGTCGTCTCTTACTACAATGCCTTAAATGCCCG | 0[328] | 0[306] |
| ACTACTTAGCCGGAACGAGGCGCTTAAATGCCCG | 7[273] | 7[295] |
| ACAGTTTTTAGATTTCAATTACCGTCGCAGAGGCGAATTTTAAATGCCCG | 4[606] | 7[608] |
| ATTTGGCAAATCAACAGTTGAAATTAAATGCCCG | 7[609] | 7[631] |
| TTCTGGAATAATCCTGATTTTGCCCGGCCGTAATTAAATGCCCG | 0[599] | 11[608] |
| GTATTAGAGCCGTCAATAGATAATTAAATGCCCG | 8[657] | 8[635] |
| CCGATAATAAAAGGGACTTAACACGCGAACCAGCAGTTAAATGCCCG | 11[639] | 0[623] |
| GGAATAACAGAGATAGACATACAAACTTGAGGATTTAGAATTAAATGCCCG | 7[632] | 8[616] |
| GATAGTGCAACATGATATTTGAATGGTTAAATGCCCG | 2[643] | 3[643] |
| AACACCCTAAGGGAGCCCTTAAATGCCCG | 10[625] | 11[638] |
| GATTTTAGACAGGCATTAAAAATATTAAATGCCCG | 0[664] | 0[642] |
| TGATTATCAGATATACGTGGCACTTAAATGCCCG | 3[602] | 3[624] |
| **Biotin modifizierte Staple-Stränge** | **5'-Ende** | **3'-Ende** |
| AACGCCAAAAGGCGGATGGCTTA | 4[251] | 4[229] |
| AAGAAACAATGACCGGAAACGTC | 4[461] | 4[439] |
| GTACATCGACATCGTTAACGGCA | 4[83] | 4[61] |
| ATACCACCATCAGTGAGGCCAAACCGTTGTAGCAA | 5[665] | 4[672] |

### 1.14. Selbstregeneration und Selbstheilung einer enzymatisch spaltbaren Helligkeitsmarkierung

Für die Etablierung einer selbstregenerierenden Helligkeitsmarkierung auf einem 6HB-Helligkeitslineal durch enzymatische Spaltung haben wir 2x10 Anbindestellen für die externe Markierung der 6HB-Struktur entworfen. Die beiden Markierungspunkte wurden entlang der 400 nm Achse des 6HB in einem Abstand von 290 nm angeordnet. Hierzu haben wir die 2x10 unmodifizierten Staple-Stränge des 6HB in caDNAno mit den in Tabelle S12 angegebenen Anbinde-Staple-Strängen ausgetauscht. Die 20 nt ATTO655 Imager-Strang-Sequenz (Tabelle S14) wurde so entworfen, dass sie die spezifische *Nb.BtsI*-Bindungssequenz *CACTGC* aufwies, so dass das Enzym an einen extern gebundenen Imager-Strang binden und es in zwei 10 nt Fragmente zerschneiden konnte.

6HB Helligkeitstandards wurden immobilisiert und extern in 1x PBS mit 12,5 mM $MgCl_2$ und 5 nM ATTO655 Imager Strang (Tabelle S14) über 1 Stunde markiert. Überschüssige Imager-Stränge wurden weggespült. Die enzymatische Spaltung der an die Anbindestellen des 6HB gebundenen Imager-Stränge wurde im 1x CutSmart® Puffer mit 12,5 mM $MgCl_2$ und 100 Einheiten/ml *Nb.BtsI* realisiert. Zur Überprüfung der Aktivität des Restriktionsenzyms wurden intern markierte 6HB-Helligkeitsstandards immobilisiert, extern markiert und aufgenommen. Dann wurde das Enzym hinzugefügt und die Probe wurde nach einer Nacht der Inkubation aufgenommen. Nach dem Abwaschen des Enzyms wurden die immobilisierten Helligkeitsstandards wieder extern markiert und aufgenommen. Helligkeitswerte wurden für einzelne, ausgewählte DNA-Origami extrahiert, gemittelt und auf die Anfangshelligkeit normiert.
Für eine selbstheilende Markierung durch enzymatische Spaltung wurden immobilisierte 6HB Helligkeitsstandards mit 1x CutSmart® Puffer mit 12,5 mM $MgCl_2$,100 Einheiten/ml *Nb.BtsI* und 5 nM Imager-Strang gleichzeitig inkubiert. Nachdem wir 30 Minuten auf Einstellung stationärer Bedingungen gewartet haben, konnte die selbstheilende Markierung aufgenommen werden.

**Tabelle S12** Die Sequenzen der Staple-Stränge, die für den 6HB Helligkeitsstandard verwendet wurden. Anbindestellen-Staple-Stränge weisen eine 20 nt-Anbinde-Sequenz mit Bindungsstelle für *Nb.BtsI* auf. ). Zur Immobilisierung sind Staple-Stränge mit Biotin am 3'-Ende modifiziert. Die Zahlen für das 5'- Ende 3'-Ende der Staple-Stränge stellen die Helixnummer in der entsprechenden caDNAno-Datei dar. Die Zahl in Klammern stellt die Start- und Endposition des Staple-Strangs in der entsprechenden Helix dar.

| Anbindestellen-Staple-Stränge | 5'-Ende | 3'-Ende |
|---|---|---|
| CAGATTCAGTACCGCATTCCAAGAACGGTTGTAGAAACCAAATTTTTTACTAGCAGTGATCTTAGCAT | 5[1134] | 2[1134] |
| CAATAATCGGCTAGAATATCCCATCCTAGTCCTGAACATTGGTTTTTACTAGCAGTGATCTTAGCAT | 2[1133] | 5[1133] |
| ATAGCAAGCAAAATGAATCATTACCGCGATTTTATTTTCATCTTTTTACTAGCAGTGATCTTAGCAT | 3[1106] | 1[1119] |
| ACGTAATTTAGGCAAAAGTACCGAGACAAAAAAGGGTAAAGCCTTTTTACTAGCAGTGATCTTAGCAT | 1[1162] | 4[1162] |
| AACGCGCCTTTAACACGACCTAATAGGTAAAGTAATATTTTTTACTAGCAGTGATCTTAGCAT | 4[1161] | 0[1148] |

| | | |
|---|---|---|
| GCTAATGCAGAACGCAATAAACAACAACATGGTTCTGTCCAGACGTTTTTACTAGCAGTGATCTTAGCAT | 3[1148] | 1[1161] |
| ATGTATCATATGCGGAACCCTTCTGACCATAAGGCGTTAAAGTTTTTACTAGCAGTGATCTTAGCAT | 4[1203] | 0[1190] |
| GTGTCTCTCTCTCTCACTCATCTCGAGAACATTATTTACAAGAATTTTTACTAGCAGTGATCTTAGCAT | 1[1120] | 4[1120] |
| GGCCAACAGAGAATGAGGCATTTTCGAGAGCGCCATATTTAATTTTTACTAGCAGTGATCTTAGCAT | 5[1176] | 2[1176] |
| CAACGCCAACATGACTCAACTAGGGGGCACCAGTAACATTCTTTTTTACTAGCAGTGATCTTAGCAT | 2[1175] | 5[1175] |
| ATCTCTGACCTCCTAGTCGAAACCTGGCACGAATATAGTTTTTTTACTAGCAGTGATCTTAGCAT | 4[195] | 0[182] |
| GGTTAAGTTGGGTAAACGACGGCGCGGGGGCGGTTATCGGTGTTTTTACTAGCAGTGATCTTAGCAT | 1[238] | 4[238] |
| ACTGCCCGAAATTGTCATGGTCATAGCTAAACGGAGGATCCCTTTTTACTAGCAGTGATCTTAGCAT | 5[168] | 2[168] |
| CGCGCACGACTTAAAAACGCGGGGAGACCAAGCTTTCTCCCTTTTTACTAGCAGTGATCTTAGCAT | 4[237] | 0[224] |
| CATTTCTCCGAAGAGACGCATTTCACATGTGGGCCTTGAATCTTTTTACTAGCAGTGATCTTAGCAT | 3[182] | 1[195] |
| TTAATGACTGTAAGGATACCGACAGTGCTGTCTAATCTATTTTTTTTACTAGCAGTGATCTTAGCAT | 5[210] | 2[210] |
| GGGAGTGACTCTATCAACTCGTGTGGTCGTGCCCAACCTTTTTTTACTAGCAGTGATCTTAGCAT | 1[196] | 4[196] |
| ACGCTCGCCCCTCTCACTCTCCCGCCAAGAATTGTAGCTGCATTTTTACTAGCAGTGATCTTAGCAT | 2[209] | 5[209] |
| GCTGCAAGGCGATGCCTCGCTATTAAAGGGCGTGCGTATTTTTTACTAGCAGTGATCTTAGCAT | 2[251] | 5[251] |
| TGTGAATTCATGGGGATGTTCTTCTAAGGGAGGAGAAGCCAGTTTTTACTAGCAGTGATCTTAGCAT | 3[224] | 1[237] |

| Biotin modifizierte Staple-Stränge | 5'-Ende | 3'-Ende |
|---|---|---|
| AGATTTAAGTCCACTTAATTGCTGAATAAACTAAAGTACGGG | 0[475] | 3[475] |
| ATTTTTTCAGGGCGTCTCAGACGTTAACAACTTTCAATA | 0[769] | 3[769] |
| TCAGAGGCATTGCAAACGATTTTTTTGAGAGAATAACATA | 0[1063] | 3[1063] |
| CCTGTGTGCTTTCCGGTTGGTGTAATGAACCTCGATAAAGAT | 0[181] | 3[181] |
| ATCGCGCACCAAATTGCGTAGATTTAGTACCTCTTACAT | 0[1315] | 3[1315] |

**Tabelle S13** Intern markierte Staple-Stränge für Kolokalisierungsexperimente, die die enzymatische Aktivität des *Nb.BtsI* prüfen. Die Zahlen für das 5'- Ende 3'-Ende der Staple-Stränge stellen die Helixnummer in der entsprechenden caDNAno-Datei dar. Die Zahl in Klammern stellt die Start- und Endposition des Staple-Strangs in der entsprechenden Helix dar.

| Intern beschriftete Staple-Stränge (ATTO532) | 5'-Ende | 3'-Ende |
|---|---|---|
| ATTO532-GGAATTATCATCACTTATTTGTGCGGTTAAAGAGGCGGT | 2[1343] | 5[1343] |
| ATTO532-ACGCTCGCCCTGCTCAATGTCCCGCCAAGAATTGTAGCTGCA | 2[209] | 5[209] |

**Tabelle S14** Imager-Strang für den 6HB Helligkeitsstandard und mit Bindestelle für *Nb.BtsI*. Andock-Sequenz ist rot, die *Nb.BtsI*-Bindungssequenz blau, die Position der Spaltung durch *Nb.BtsI* wird durch einen Schrägstrich angezeigt.

| Sequenz (5' bis 3') | Fluorophor-Markierung auf 3' | Andocklänge (nt) |
|---|---|---|
| ATGCTAAGAT/CACTGCTAGTTT | ATTO655 | 20 |

## 2. Ergebnisse und Diskussion

### 2.1. Selbstregenerierende Helligkeitsmarkierung auf 12HB Helligkeitsstandard

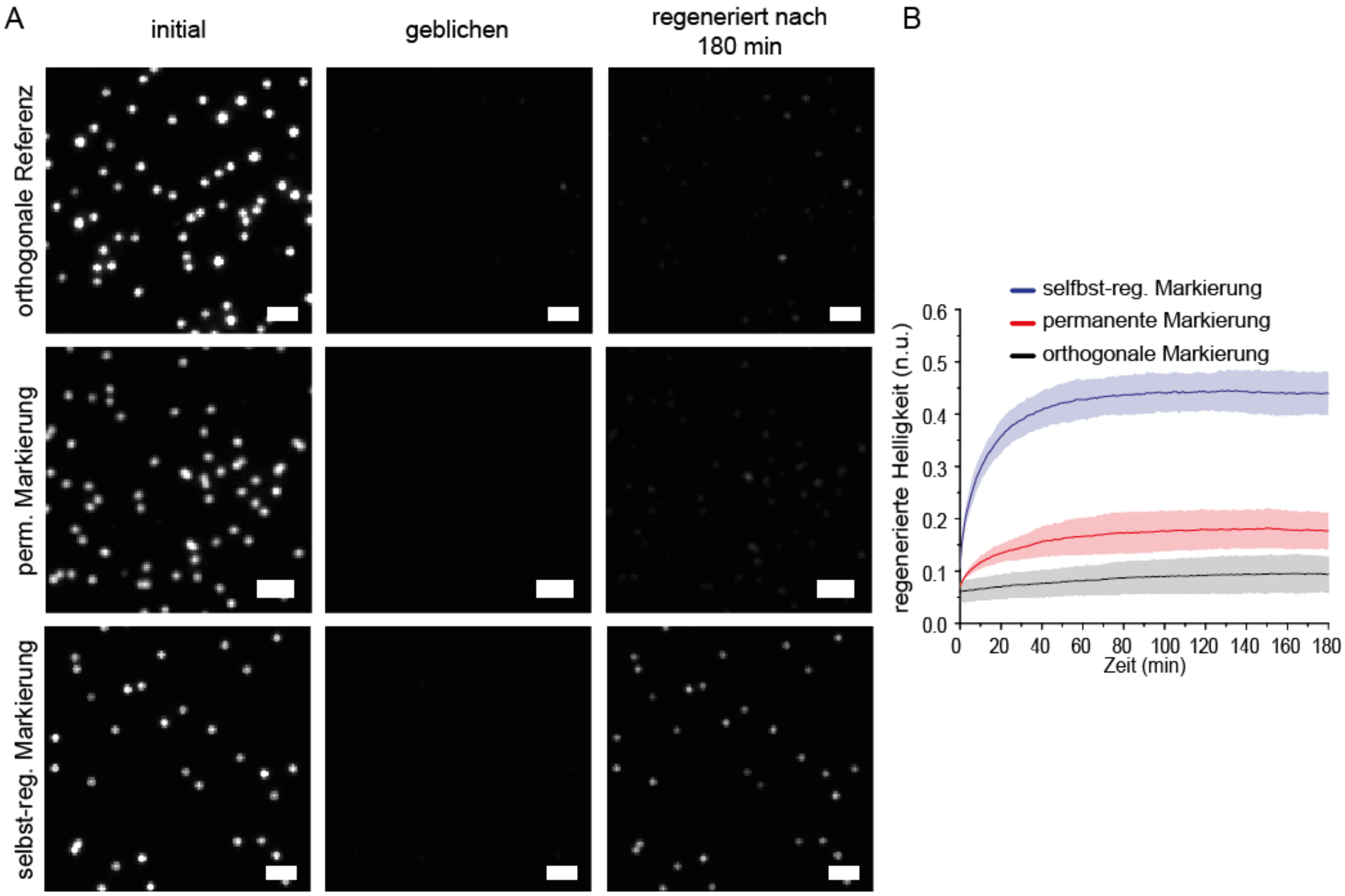


**Abbildung S2.** (A) Beispielhafte TIRF-Bilder von graduellem Bleichen und Erholung (initial, geblichen, und wiederhergestellt nach 180 min) einer orthogonalen permanenten Markierung, einer permanenten Markierung und einer selbstregenerierenden Markierung mit ATTO655 (ohne Photostabilisierung). Die Proben wurden über 3 min mit 75 W/cm2 Anregung bei 640 nm geblichen. Maßstabsbalken repräsentieren 2 µm. (B) Extrahierte gemittelte und normalisierte Einzel-DNA-Origami-Intensitätsspuren nach Photobleichen. Die selbstregenerierende Markierung (blau) zeigt eine Erholung von rund 40%. Die permanente Markierung weist eine kleine Erholung aufgrund nachträglicher Markierung (15%) auf, während eine orthogonale Referenz keine signifikante Erholung der Helligkeit zeigt. Die Daten stellen den Durchschnitt von drei unabhängigen Experimenten dar, hervorgehobene Bereiche stellen die Standardabweichung dar.

Exemplarische TIRF-Bilder mit anfänglicher, geblichener und wiederhergestellter Helligkeit nach 180 min für permanente und dynamische Helligkeitsmarkierungen (ATTO655) sind in Abbildung S2A gegeben. Um zu untersuchen, ob eine mögliche Wiederherstellung auf eine unspezifische Bindung der Imager-Stränge an die immobilisierte DNA-Origami zurückzuführen ist, haben wir auch die Wiederherstellung eines orthogonalen Imager-Strangs gemessen, d.h. eines Oligonukleotids, das mit ATTO655 markiert ist, aber mit einer 20 nt-Sequenz, die nicht komplementär zu den verwendeten Anbindestellen ist. Entsprechende extrahierte und gemittelte Intensitätsspuren nach Photobleichen pro einzelnem Helligkeitsstandard sind in Abbildung S2B dargestellt. Während der orthogonale Imager-Strang nur eine sehr langsame, unbedeutende Erholung aufgrund der unspezifischen Bindung von Imager-Strängen an die Nanostrukturen aufwies, ergab die permanente Markierung eine etwas höhere Erholung von etwa 15%. Diese geringe Wiederherstellung erklärt sich durch die nachträgliche Markierung von zunächst unzugänglichen Anbindestellen. Studien über die Zugänglichkeit von extern markierten DNA-Origami zeigen in der Regel Zugänglichkeiten der Anbindestellen im Bereich von 60 bis 90%.[10] Nach dem Bleichen der permanenten Markierungen und nachfolgenden ROS-induzierten Schäden an den Anbindestellen könnten zunächst unzugängliche Anbindestellen für intakte permanente Imager-Stränge in der Lösung zugänglicher werden. Die selbstregenerierende Markierung zeigte jedoch eine deutlich verbesserte Erholung von rund 40% unter identischen Bedingungen.

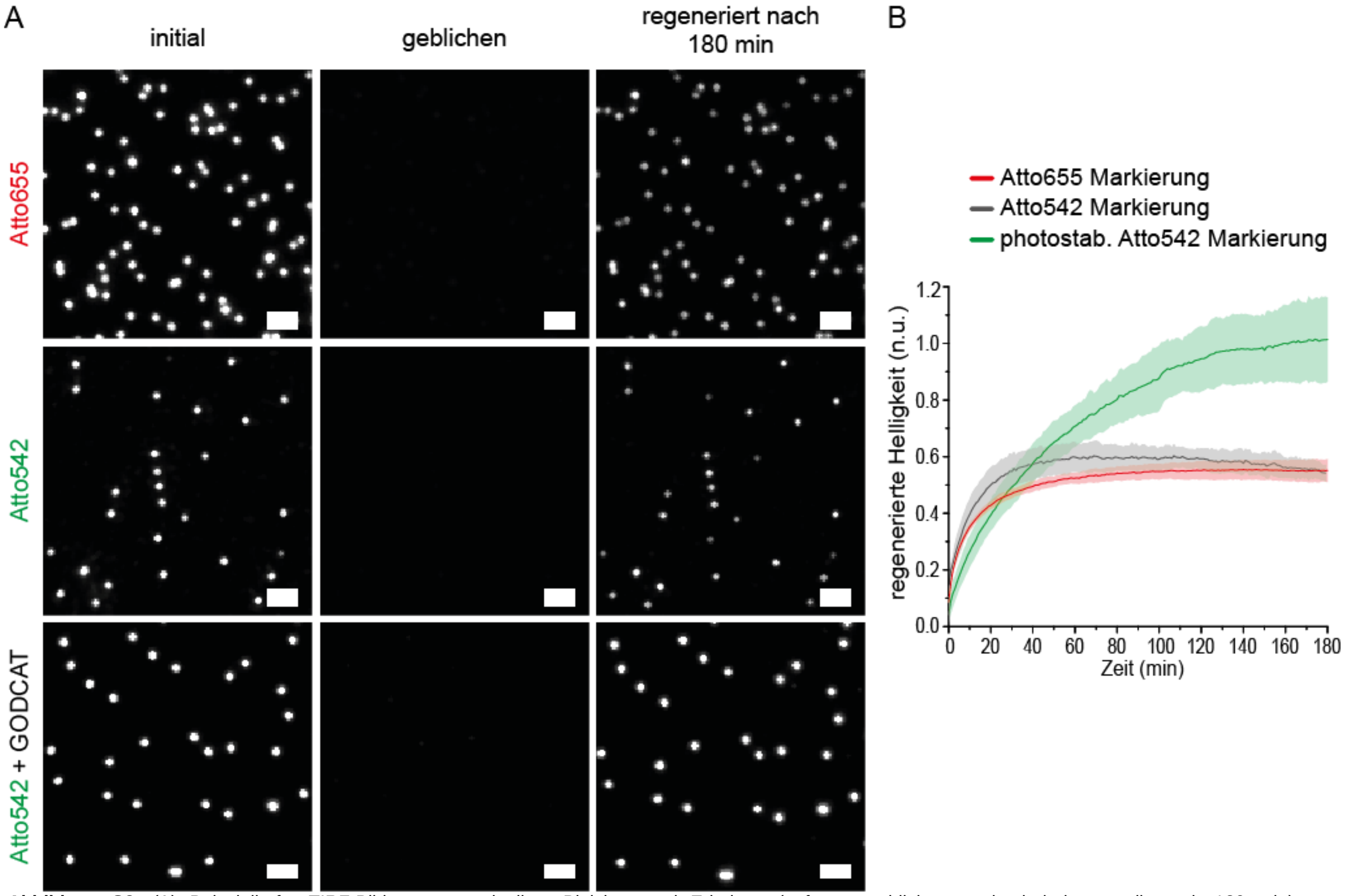


**Abbildung S3**. (A) Beispielhafte TIRF-Bilder von graduellem Bleichen und Erholung (anfangs, geblichen, und wiederhergestellt nach 180 min) von selbstregenerierenden Markierungen ohne Photostabilisierung (ATTO655 und ATTO542) und einer selbstregenerierenden Markierung mit Photostabilisierung (ATTO542). Die Proben wurden über 3 min mit 0,5 kW/cm$^2$ Anregungsleistung geblichen. Maßstabsbalken repräsentieren 2 µm. (B) Entsprechende extrahierte, gemittelte und normierte Einzel-DNA-Origami-Intensitätsspuren nach Photobleichen. Selbstregenerierende Markierungen ohne Photostabilisierung (rot und grau) zeigen eine begrenzte Erholung von etwa 50 bis 60%. Die photostabilisierte (GODCAT, Trolox) ATTO542 Markierung wies eine vollständige Wiederherstellung von bis zu 100% seiner ursprünglichen Helligkeit auf. Die Daten stellen den Durchschnitt von drei unabhängigen Experimenten dar, hervorgehobene Bereiche stellen die Standardabweichung dar.

Um Einschränkungen durch photoinduzierte Schäden zu überwinden, haben wir einen Imager-Strang verwendet, der mit dem Rhodamine-Farbstoff ATTO542 modifiziert wurde und durch ein enzymatisches Sauerstoff-Abfangsystem und ROXS[5, 11] photostabilisiert werden kann. Zur Sauerstoffentfernung wurde ein 2,5-facher TAE-Puffer mit Glukose, Glucose-Oxidase und Katalase verwendet. Um Triplet-Zustände der ATTO542 Farbstoffe zu quenchen, wurde eine Trolox/Trolox Quinon-Mischung als Reduktions- und Oxidationssystem verwendet (ROXS). Graduelles Bleichen und Erholung der Helligkeit selbstregenerierender Markierungen mit und ohne Photostabilisierung sind in Abbildung S3 gegeben. Um die photostabilisierten Markierungen vollständig zu bleichen, wurden höhere Laserleistungen von 0,5 kW/cm$^2$ über 3 min aufgewendet. Während die selbstregenerierenden ATTO655- und ATTO542-Markierungen ohne Photostabilisierung erneut eine begrenzte Erholung von nur bis zu 60 % zeigten, ergab die photostabilisierte ATTO542-Markierung eine vollständige Erholung von bis zu 100% der urspränlgichen Helligkeit. Unter den verwendeten Zeitraffer-Aufnahmebedingungen und der angewandten Photostabilisierung konnte eine vollständige Reparatur der photoinduzierten Schäden an der Helligkeitsfunktion durch selbstregenerierende Markierungen realisiert werden.

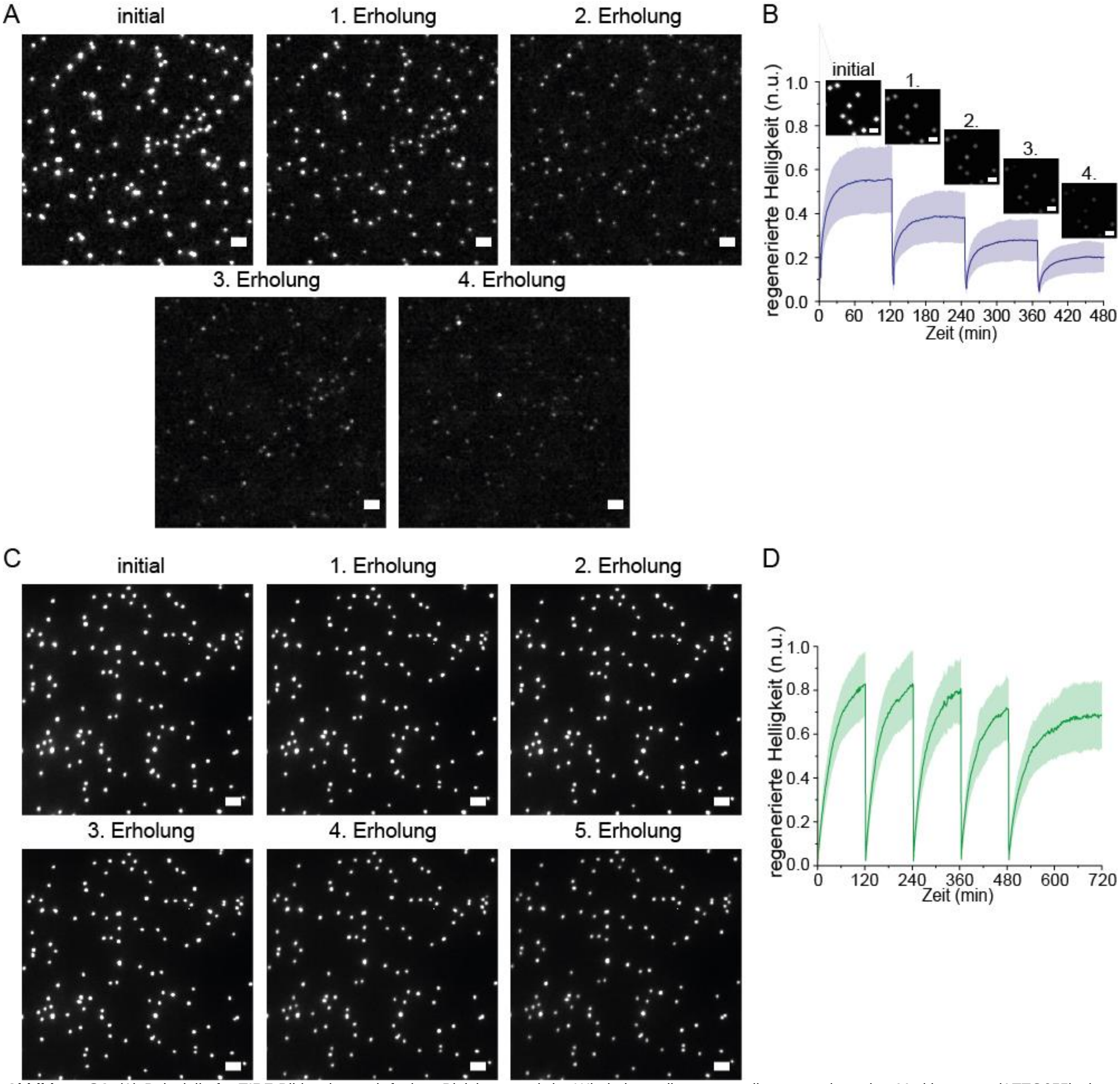


**Abbildung S4.** (A) Beispielhafte TIRF-Bilder des mehrfachen Bleichens und der Wiederherstellung von selbstregenerierenden Markierungen (ATTO655) ohne Photostabilisierung über 4 Bleichereignisse (3 min Bleichen mit 75 W/cm$^2$ bei 640 nm). Maßstabsbalken repräsentieren 1 µm. (B) Entsprechende extrahierte, gemittelte und normierte Einzel-DNA-Origami-Intensitätsspuren über 4 Bleich- und Erholungszyklen. (C) Beispielhafte TIRF-Bilder des mehrfachen allmählichen Bleichens und der Wiederherstellung der selbstregenerierenden Markierung (ATTO542) mit Photostabilisierung über 5 Bleichereignisse (3 min Bleichen mit 0,5 kW/cm$^2$ bei 532 nm). Maßstabsbalken repräsentieren 1 µm. (D Entsprechende extrahierte, gemittelte und normierte Einzel-DNA-Origami-Intensitätsspuren über 5 Bleich- und Erholungszyklen. Die Daten stellen den Durchschnitt eines Experiments dar, hervorgehobene Bereiche stellen die Standardabweichung dar.

Um die Selbstregeneration der Markierungen über mehrfache Schadensereignisse zu untersuchen, haben wir das gleiche Sichtfeld mehrmals gebichen und die Wiederherstellung der Helligkeit nach jedem Bleichereignis in Zeitraffer gemessen. Beispielhafte TIRF-Bilder nach jedem Bleich- und Erholungszyklus der selbstregenerierenden Markierung ohne Photostabilisierung (Abbildung S4A) und entsprechende extrahierte durchschnittliche Intensitätsspuren der einzelnen DNA Origami in Abbildung S4B zeigten, dass sich die selbstregenerierenden Markierungen auch nach 4 Bleichereignissen wieder auf über 20% der anfänglichen Helligkeit erholen konnten. Die photoinduzierten Schäden an den Anbindestellen durch ROS sind dabei deutlich sichtbar, da die Wiederherstellung über jedes Bleichereignis von anfänglichen ca. 60% auf ca. 20% abnimmt. Analoges mehrfaches Bleichen und Wiederherstellen der selbstregenerierenden ATTO542 Helligkeitsmarkierung mit Photostabilisierung (Abbildung S4C und D) ergab eine stark erhöhte Erholung über mehrere Bleichereignissen. Unter den verwendeten Aufnahmebedingungen (120 min für die Wiederherstellung) erholte sich das photostabilisierte, selbstregenerierende Markierung in den ersten drei Erholungszyklen auf über 80 %. Erst nach dem vierten Bleichereignis war eine verminderte Erholung von rund 70% und nach dem fünften Bleichen von 60% und damit ein zunehmender Schaden an den Anbindestellen sichtbar. Die Ergebnisse aus Abbildung S3 deuten auf eine vollständige Wiederherstellung der photostabilisierten selbstregenerierenden Markierung nach 180 min hin. Um die Wahrscheinlichkeit einer Defokussierung oder zu starkem Probendrift während der Datenerfassung zu minimieren, wurde die Wiederherstellung für mehrere Bleichereignisse nur über

120 min (d. h. vor dem vollständigen Austausch aller defekter Imager-Stränge) untersucht, bis der nächste Bleichzyklus initialisiert wurde.

## 2.2. Reparatur der geknickten 12HB Nanolineale

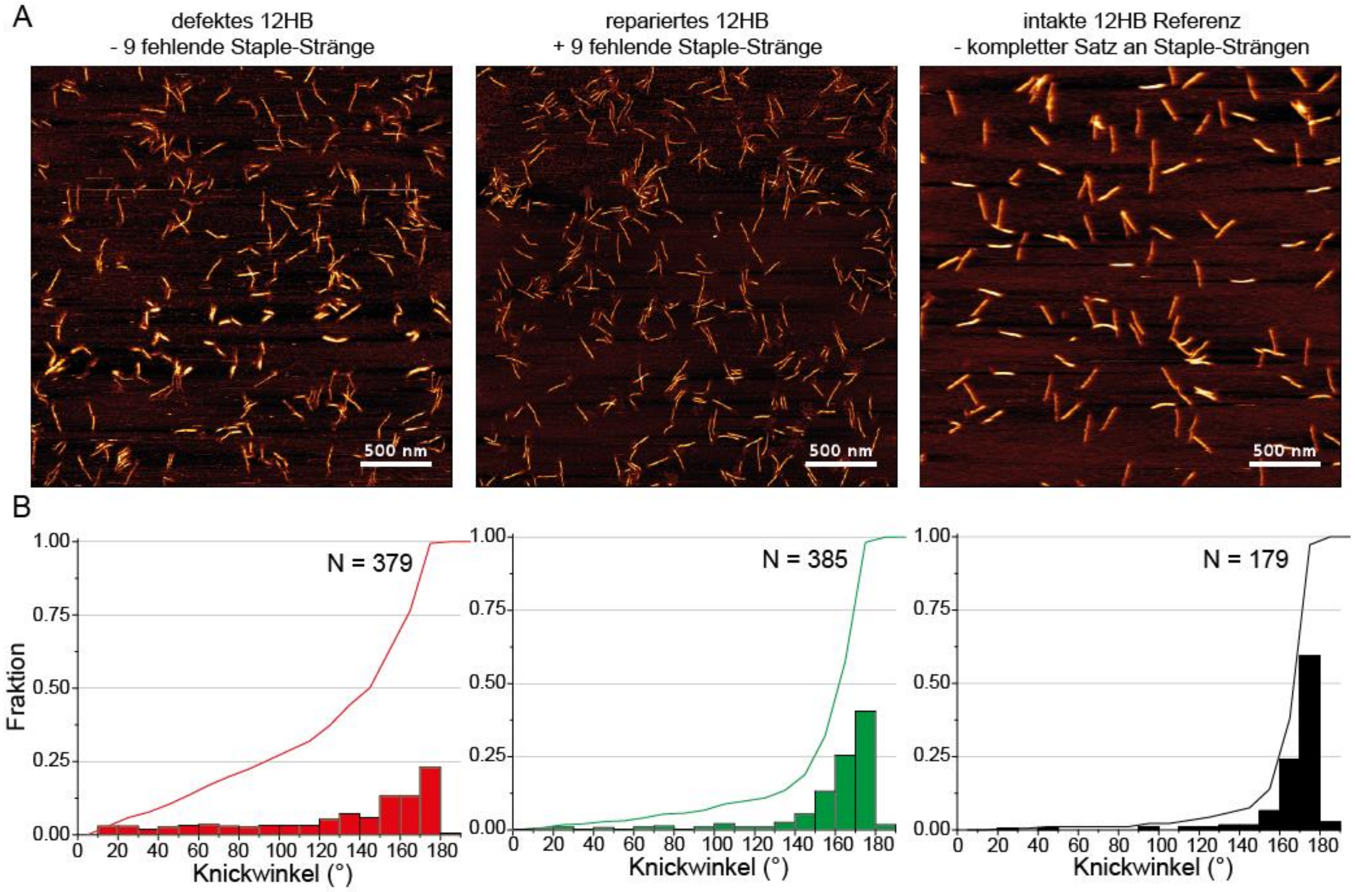


**Abbildung S5.** A) Beispielhafte AFM-Bilder der 12HB DNA-Origami gefaltet mit 9 ausgelassenen Staple-Strängen in der zentralen Region und mit Faltungsprogramm 1, der reparierten 12HB DNA Origami mit eingebauten fehlenden Staple-Strängen nach einem zweiten Faltvorgang mit Faltungsprogramm 1 für T≤50°C und eine intakte 12HB Referenz gefaltet mit dem kompletten Satz an Staple-Strängen. Maßstabsbalken repräsentieren 500 nm. B) Entsprechende Winkelverteilungshistogramme, die durch manuelle Winkelmessung über N asugewählte Molekülen aus den AFM-Bildern erhalten wurden. Linien stellen kumulative Verteilungen dar.

Beispielhafte AFM-Bilder in Abbildung S5A zeigten eine große Population defekter 12HB-DNA-Origami, wenn bei der ersten Faltung der DNA-Origami 9 Stapel-Stränge im zentralen Bereich des Nanolineals weggelassen wurden. Die defekten Strukturen waren meist geknickt und zeigten eine verminderte Höhe im Bereich des einsträngigen Scaffold-Strangs. Um die Inkorporation der 9 fehlenden Staple-Stränge in die bereits gefalteten defekten 12HB-Nanolineale zu beschleunigen, haben wir eine Temperaturrampe nach Faltungsprogramm 1 in Tabelle S3, jedoch mit T ab 50°C, verwendet und einen 300-fachen Überschuss der 9 zugesetzten Staple-Stränge in Bezug auf die gereinigten 12HB eingesetzt. Die niedrige Starttemperatur wurde gewählt, um Degradierung der bereits gefalteten DNA-Origami zu verhindern. In einem ähnlichen Ansatz wurde ein Scaffold-Strang mit einer geringen Anzahl von Staple-Strängen in einem ersten Faltungsschritt mit hoher Starttemperatur gefaltet. In einer zweiten Faltung mit niedrigeren Starttemperaturen konnte der Satz fehlender Staple-Stränge den vorgefalteten Scaffold-Strang erfolgreich in die gewünschte Form falten.[12] Nach Zugabe der fehlenden Staple-Stränge wurde die Population der defekten 12HB signifikant verringert. Quantitative Analyse wurde durch manuelle Winkelmessungen zwischen den beiden Hälften ausgewählter Nanolineale mit ImageJ durchgeführt. Um eine Untersuchung nur jener Nanolineale, deren Strukturen durch das unvollständige Hybridisieren des Scaffold-Strangs beeinflusst wurden, sicherzustellen, wurden nur jene analysiert, die als isolierte Monomere immobilisiert vorlagen,während Aggregate verworfen wurden. Die erhaltenen Winkelverteilungen in Abbildung S5B zeigen eine breite Verteilung von 0 bis 180° für die defekten Strukturen. Die 12HB-Probe nach der Reparatur mit den 9 fehlenden Staple-Stränge zeigte eine verbesserte und engere Winkelverteilung, die hin zu der Winkelverteilung einer intakten 12HB Referenz-Probe verschoben wurde, die mit einem kompletten Satz von Staple-Strängen gefaltet wurde. Die Klassifizierung aller Nanolineale mit einem Winkel unter 160° als defekt führte zu einer defekten Population von 63% nach dem ersten Falten unter Auslaßen der 9 Staple-Stränge und von nur 32% nach zweitem Falten unter Zugabe der fehlenden Staple-Stränge. Die Winkelverteilungen deuten darauf hin, dass die 9 fehlenden Staple-Stränge erfolgreich in das defekte 12HB-Origami inkorporiert wurden und die emulierten Strukturschäden teilweise repariert wurden, was zu einer verbesserten strukturellen Integrität der Nanolineale führte.

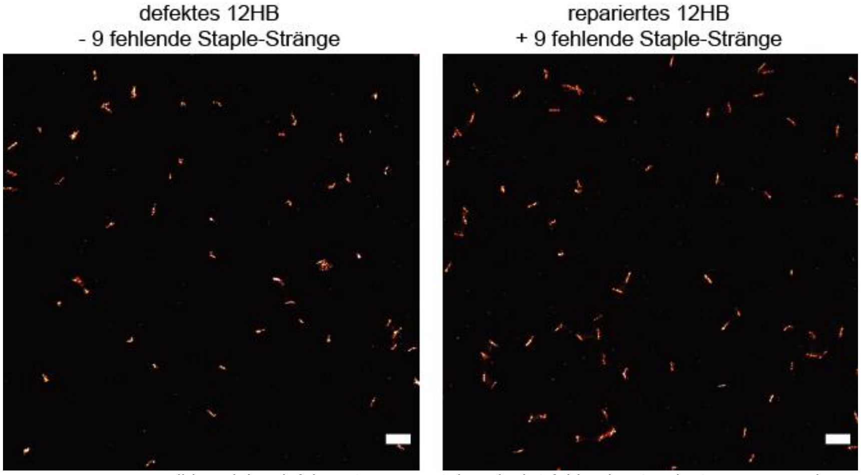


**Abbildung S6.** Beispielhafte Kontur DNA PAINT Bilder. Links: defekte 12HB DNA Origami mit 9 fehlenden Staple-Strängen. Rechts: reparierte 12HB DNA Origami. Maßstabsbalken repräsentieren 500 nm.

Zusätzlich haben wir die defekten und reparierten 12HB-Nanolineale mittels DNA PAINT und einer dichten Anbindestellenverteilung über die gesamte Länge des 12HB untersucht. Mit dieser Markierungsstrategie konnten wir die Kontur der Nanolineale mit Superauflösung ähnlich der AFM-Bildgebung abbilden und visualisieren (Abbildung S6). DNA PAINT Bilder der defekten 12HB Nanolineale zeigten eine große Population (72%) von zusammengebrochenen oder abgeknickten 12HB und eine kleine Population linearer Nanolineale. Die reparierten 12HB-Nanolineale zeigten eine signifikant erhöhte Population linearer, intakter Nanolineale, während die Population der sichtbar defekten Nanolineale auf 38% zurückging.
Es fällt auf, dass die sichtbar defekten 12HB-Nanolineale in den DNA-PAINT-Bildern mehr zusammengebrochene Strukturen zeigten als in entsprechenden AFM-Bildern. Wir schreiben diesen Unterschied den verschiedenen Immobilisierungsstrategien zu (Poly-L-Ornithin auf Glimmer in AFM, Biotin-NeutrAvidin Immobilisierung auf BSA passivierter Glasoberfläche in DNA PAINT). Für die AFM-Aufnahmen wurden die Nanolineale über ionische Wechselwirkungen mit der positiv geladenen Oberfläche und damit über die gesamte Länge des 12HB immobilisiert. In DNA PAINT wurden die 12HB über nur 4 Biotin modifizierte Staple-Stränge, zwei auf jeder Stelle des emulierten Schadens, immobilisiert und sollten daher eine höhere Flexibilität aufweisen. Die zusammengebrochenen Nanolineale könnten defekte, flexible Strukturen darstellen, die nur über eine Seite des emulierten Schadens immobilisiert wurden.

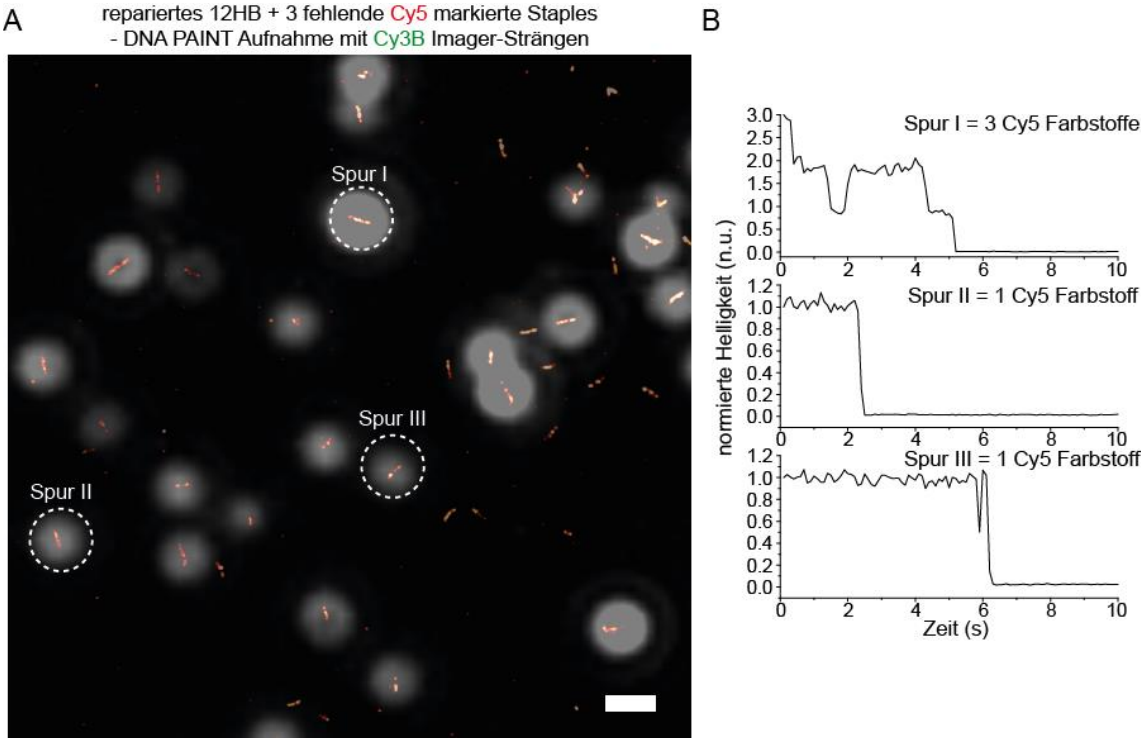


**Abbildung S7.** (A) Kolokalisiertes Weitfeld-DNA-PAINT-Bild von reparierten 12HB, die mit sechs unmodifizierten fehlenden Staple-Strängen und drei Cy5 modifizierten fehlenden Staple-Strängen in der zweiten Faltung repariert wurden. Kontur DNA PAINT Bilder wurden mit einem 8 nt Cy3B Imager in grün aufgenommen. Weitfeld-Signale der Cy5-markierten Staple-Stränge (graue PSF) zeigen eine erfolgreiche Inkorporation für einen hohen Anteil der 12HB DNA-Origami. (B) Beispielhaft extrahierte Intensitätsspuren einzelner Nanolineale zeigen Bleichschritte für 1 bis 3 integrierte Cy5-Farbstoffe pro repariertem DNA-Nanolineal. Maßstabsbalken repräsentieren 500 nm.

Neben der durchgeführten AFM- und DNA PAINT-Charakterisierung der defekten und reparierten 12HB-Nanolineale haben wir ein kolokalisiertes WEitfeld-DNA-PAINT-Experiment durchgeführt, um die Inkorporation der 9 fehlenden Staple-Stränge zweifelsfrei zu klären (Abbildung S7). Hierzu tauschten wir 3 der 9 fehlenden Staple-Stränge gegen Cy5-markierte Staple-Stränge aus. Nach der

Reparatur mit dem teilweise Cy5-markierten Satz an Staple-Strängen, erhielten wir beugungsbegrenzte Cy5-Signale in Rot (640 nm) und entsprechende DNA PAINT-Bilder derselben Regionen mit einem Cy3B-markierten 8 nt DNA PAINT-Imager-Strang. Das kolokalisierte Bild der beugungsbegrenzten Cy5-Signalen und DNA-PAINT-Informationen ergab eine erfolgreiche Inkorporation der Cy5-markierten Staple-Stränge in die Mehrzahl der 12HB-Nanolineale, die mit DNA PAINT vermessen wurden, da nur ein Drittel der neun fehlenden Staple-Stränge mit Cy5 gekennzeichnet waren. Während die DNA-PAINT-Bilder die Struktur der Nanolineale zeigten, konnten die beugungsbegrenzten Einzelpunkte weiter untersucht werden, indem die entsprechenden Intensitätsspuren bis zum Bleichen extrahiert wurden. Während die meisten Transienten die Inkorporation einer Cy5-Markierung aufwiesen, zeigte eine Minderheit auch zwei oder sogar drei inkorporierte Cy5-markierte Staple-Stränge (siehe beispielhaft Intensitätsspuren in Abbildung S7 B). Die kolokalisierten Bilder deuten auf eine signifikante Inkoporation mindestens eines Teils der neun fehlenden Staple-Stränge hin, so dass die oben gezeigten Änderungen in AFM- und DNA-PAINT-Experimenten während der Reparatur tatsächlich auf die Inkorporation fehlender Staple-Stränge zurückgeführt werden kann.

Die Ergebnisse der AFM-Aufnahmen und der DNA PAINT-Experimente zeigten konsistente Ergebnisse. Der unvollständige Satz von Staple-Strängen in der ersten Faltung führte zu einer defekten DNA-Origami-Population, die teilweise repariert und in ihrer strukturellen Integrität verbessert werden konnte. Der Vergleich mit einer intakten Referenzstruktur zeigte, dass die Reparatur die emulierten Schäden nicht in der gesamten Nanolinealpopulation beseitigen konnte, aber zu einer signifikanten Verbesserung der strukturellen Verteilung führte.

### 2.3. Staple-Strang-Austausch vom Doppel-Punkt zum Triple-Punkt im NRO-Nanolineal

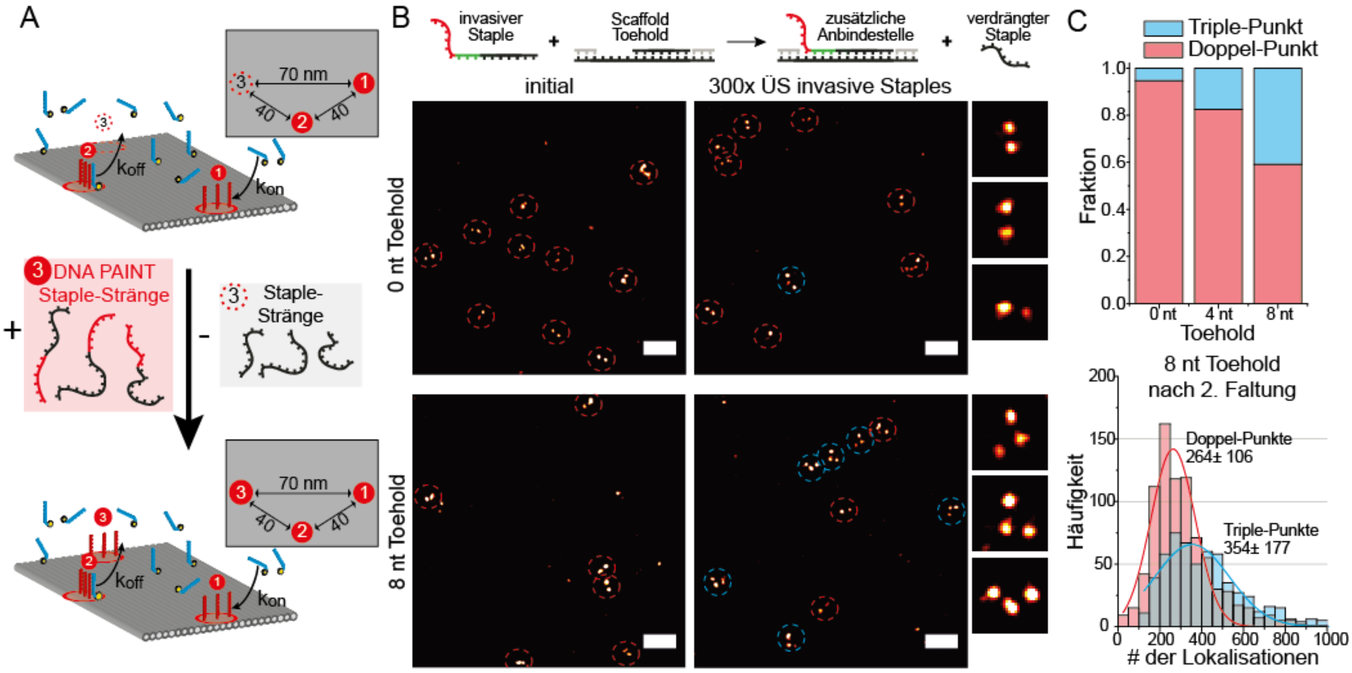


**Abbildung S8.** (A) Schema des rekonfigurierbaren NRO-Nanolineals. Durch Zugabe von DNA PAINT Staple-Strängen in einer zweiten Faltung kann ein Doppelpunkt-Nanolineal (40 nm) in ein Triplepunkt-Nanolineal umgewandelt werden. (B) Schema des beschleunigten Austauschs der Staple-Strängen durch Toeholds auf dem Scaffold-Strang. Exemplarische DNA PAINT-Bilder von 0 nt und 8 nt Toehold Nanolinealen vor und nach Zugabe der Triplepunkt-Staple-Stränge in einer zweiten Faltung zeigen doppelte Punkte (rote Kreise) vor und teilweise dreifache Punkte (blaue Kreise) nach der Inkubation. Beispielhafte Vergrößerungen zeigen Doppelpunkt-Nanolineale (0 nt Toehold) und Triplepunkt-Nanolineale (8 nt Toehold) nach dem zweiten Falten. Maßstabsbalken stellen 200 nm dar. (C) Relative Fraktionen der Doppel- und Tiplepunkt-Nanolineale für 0,4 und 8 nt Toehold Proben nach der zweiten Faltung, die eine zunehmende Triplepunkt-Population mit zunehmender Toeholdlänge offenbaren. Beispielhaft extrahierte Lokalisationen aus DNA-PAINT-Experimenten für die 8 nt Toehold-Probe nach der zweiten Faltung deuten auf eine 34%ige Zunahme der Anbindestellen durch Inkorporation der invasiven Staple-Stränge hin.

Das vorherige Experiment mit den abgeknickten 12HB-Nanolinealen zeigt, dass DNA-Stränge in bestehende DNA-Origami-Nanostrukturen integriert werden können, aber es handelt sich dabei um keinen Selbstheilungsmechanismus, da es denkbar ist, dass Staple-Stränge auch in intakten DNA-Origami-Strukturen austauschen werden. Zu diesem Zweck haben wir ein rechteckiges DNA-Origami mit zwei Markierungspunkten (40 nm Abstand) entworfen, welche aus Anbindestellen für DNA-PAINT-Messungen bestehen (Abbildung S8). Wir fügten anschließend Staple-Stränge mit DNA PAINT Anbindestellen hinzu, die bei Inkorporation einen dritten Punkt auf dem DNA-Origami bilden würden. Um die Austauschkinetik zu beschleunigen, haben wir die Doppelpunkt-NROs mit einem 300-fachen Überschuss der invasiven Staple-Stränge in Lösung inkubiert unter Verwendung der Temperaturrampe des NRO-Faltungsprogramms, aber beginnend bei T=50°C, d.h. unterhalb der Denaturierungstemperatur (Tabelle S4). Interessanterweise wurde nur eine verschwindend kleine Anzahl von Dreifach-Punkt-DNA-Origamis beobachtet, was darauf hindeutet, dass der Stapelaustausch kinetisch blockiert war. Wenn jedoch das DNA-Origami zuvor mit kürzeren Staple-Strängen im Bereich der dritten Markierung synthetisiert wurde, so dass ein Toehold von 4 oder 8 Nukleotiden im Scaffold gebildet wurde, konnten die verlängerten Staple-Stränge die vorhandenen Staple-Stränge effizienter ersetzen (siehe Schema und Bilder in Abbildung S8). Nach der Inkubation mit den verlängerten Staple-Strängen zeigten zwischen 20 und 40% der DNA-Origamis das dreifache Markierungsmuster, wie in Abbildung S8 B und C dargestellt, was die Vorstellung bestätigt, dass ein Toehold für effiziente Strangverdrängungsreaktionen auch innerhalb eines intakten DNA-Origami erforderlich ist.[12-14] Die erfolgreiche Inkorporation der Staple-Stränge, die den dritten Markierungspunkt bilden, konnte weiterhin untersucht werden, indem die Anzahl der Lokalisationen pro DNA-Origami-Nanostruktur betrachtet wurde. Die Anzahl der Anbindestellen wird während der Inkoporation des dritten Markierungspunkts erhöht, was auch zu einer Zunahme der Lokalisationen pro DNA-PAINT-Experiment führen sollte. Für die 8 nt Toehold-Probe ergaben die ausgewählten Triplepunkt-Nanolineale eine durchschnittliche Anzahl von Lokalisationen von etwa 354, verglichen mit nur 264 Lokalisationen für die ausgewählten Doppelpunkt-Nanolineale innerhalb derselben Probe. Für einen quantitativen Austausch und Inkorporation werden 150% der Lokalisationen der Doppelpunkt-Nanolineale für die Triplepunkt-Population erwartet (da 6 Anbindestellen theoretisch auf 9 Anbindestellen erhöht werden). Der beobachtete Anstieg der Lokalisationen auf 134% zeigt, dass unter den verwendeten Bedingungen durchschnittlich etwa zwei der drei Anbindestellen-Staple-Stränge inkorporiert wurden.

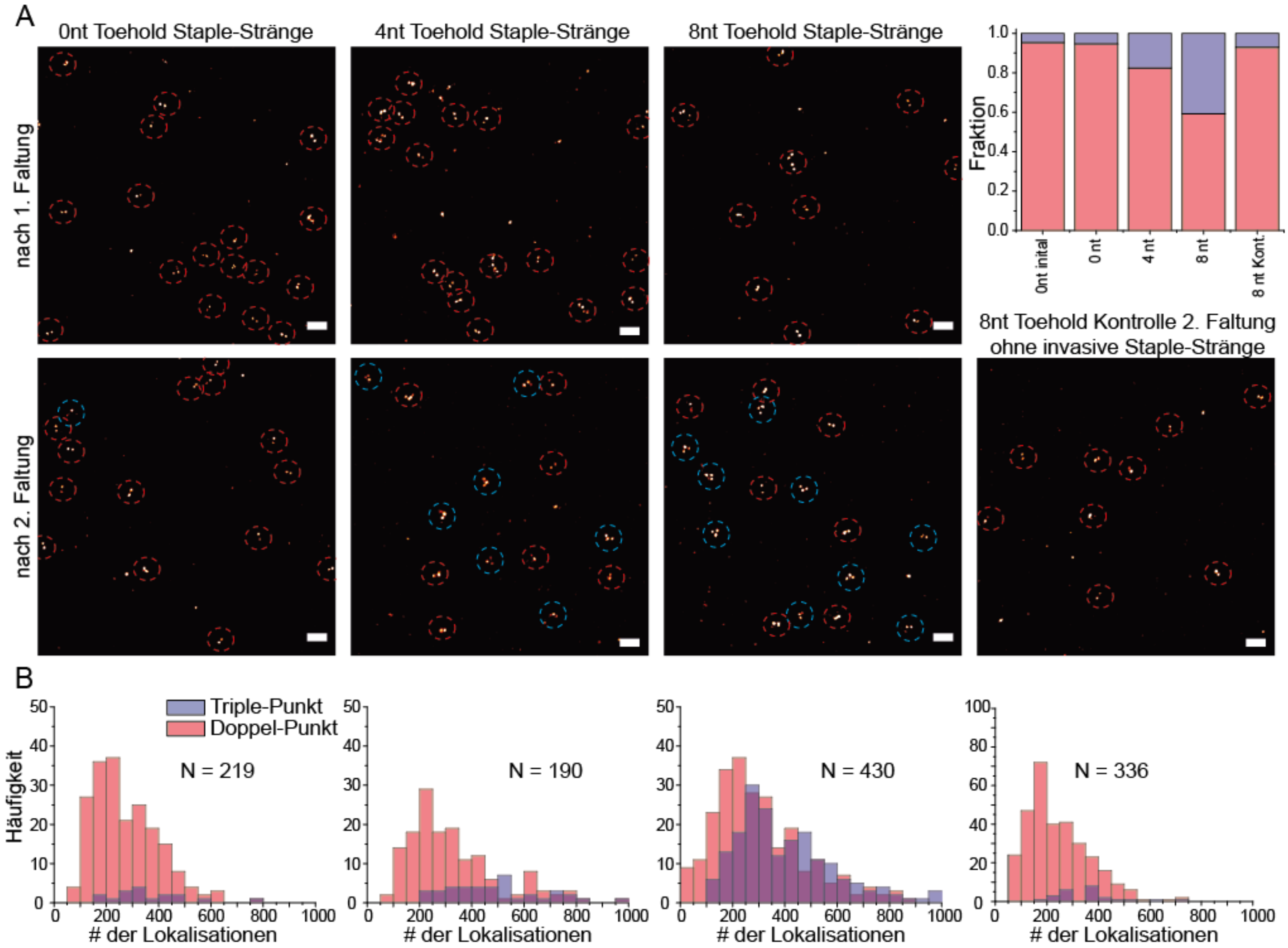


**Abbildung S9**. (A) Beispielhafte DNA PAINT-Bilder der 0 nt, 4 nt und 8 nt Toehold Nanolineale zeigen Doppelpunkte (rote Kreise) vor und teilweise Triplepunkte nach (blaue Kreise) der Zugabe der Triplepunkt-Staple-Stränge in einer zweiten Faltung. Exemplarisches DNA PAINT-Bild einer 8 nt Toehold Probe nach zweiter Faltung ohne Zugabe der Triplepunkt-Staple-Stränge zeigen nur Doppelpunkt-Nanolineale. Maßstabsbalken stellen 200 nm dar. Relative Fraktionen der Doppel- und Triplepunkt-Nanolineale für 0 bis 8 nt Toeholds und für eine Referenz nach der zweiten Faltung zeigen steigende Triplepunkt-Populationen mit zunehmender Toehold-Länge, aber keine signifikante Triplepunkt-Population ohne Zugabe der invasiven Staple-Stränge. (B) Extrahierte Lokalisationen aus DNA-PAINT-Experimenten für die 0 bis 8 nt Toeholdproben und der 8 nt Referenzprobe nach der zweiten Faltung zeigen eine Erhöhung der Anzahl der Anbindestellen durch Inkorporation der invasiven Staple-Stränge.

Ein weiterer Vergleich exemplarischer DNA-PAINT-Bilder der 0 bis 8 nt Toehold-Proben und den entsprechenden extrahierten Anzahlen der Lokalisationen sind in Abbildung S9 gegeben. Bei der manuellen Selektion der Doppel- und Triplepunkt -Nanolineale in den erhaltenen DNA-PAINT-Bildern beobachteten wir einen kleinen Bruchteil von Pseudo-Triplepunkten in den Proben, noch bevor wir die Triplepunkt-Staple-Stränge hinzugefügt hatten. Untersuchung der 0 nt Toehold-Probe vor Zugabe der invasiven Staple-Stränge ergab z.B. einen kleinen Bruchteil von etwa 5% solcher Pseudo-Triplepunkte. Wir schreiben diese Population NRO-Dimeren zu, die zufällig dreifache Punkte bilden, indem sich zwei einzelne Doppelpunkt-Nanolineale überlagern. Während die Zugabe der invasiven Staple-Stränge zu keiner signifikanten Erhöhung der Triplepunk-Population für die 0 nt-Toeholdprobe führte, konnte ein signifikant höherer Anteil von etwa 20 % für die 4 nt Toehold-Probe und etwa 40% für die 8 nt Toehold-Probe gefunden werden. Nach der Toehold vermittelten Strangverschiebungskinetik wird der Austausch durch die Erhöhung der Toeholdlängen beschleunigt. Ob die zunehmende Zahl der Triplepunkt-NROs in erster Linie auf die Bildung von zusätzlichen Pseudo-Triplepunkten durch unspezifische Dimerisierung des DNA-Origami während der zweiten Faltung zurückzuführen ist, haben wir eine Referenzprobe für die zweite Faltung vorbereitet. Eine 8 nt Toehold Doppelpunkt-NRO-Probe wurde mit dem gleichen Puffer und der Temperaturrampe der NRO-Faltung (ab T= 50°C) behandelt, jedoch ohne Zugabe der invasiven Staple-Stränge, die den dritten Markierungspunkt bilden. DNA-PAINT-Bilder zeigten keinen signifikanten Anstieg der Pseudo-Triplepunkt-Population (Abbildung S9), was darauf hindeutet, dass die erhaltenen Triplepunkt-Populationen nach Zugabe der invasiven Staple-Stränge auf eine erfolgreiche Inkoporation in die vorhandenen DNA-Origami-Nanostrukturen zurückgeführt werden können.

### 2.4. Selbstheilung von 12HB Nanolinealen unter degradierenden Bedingungen

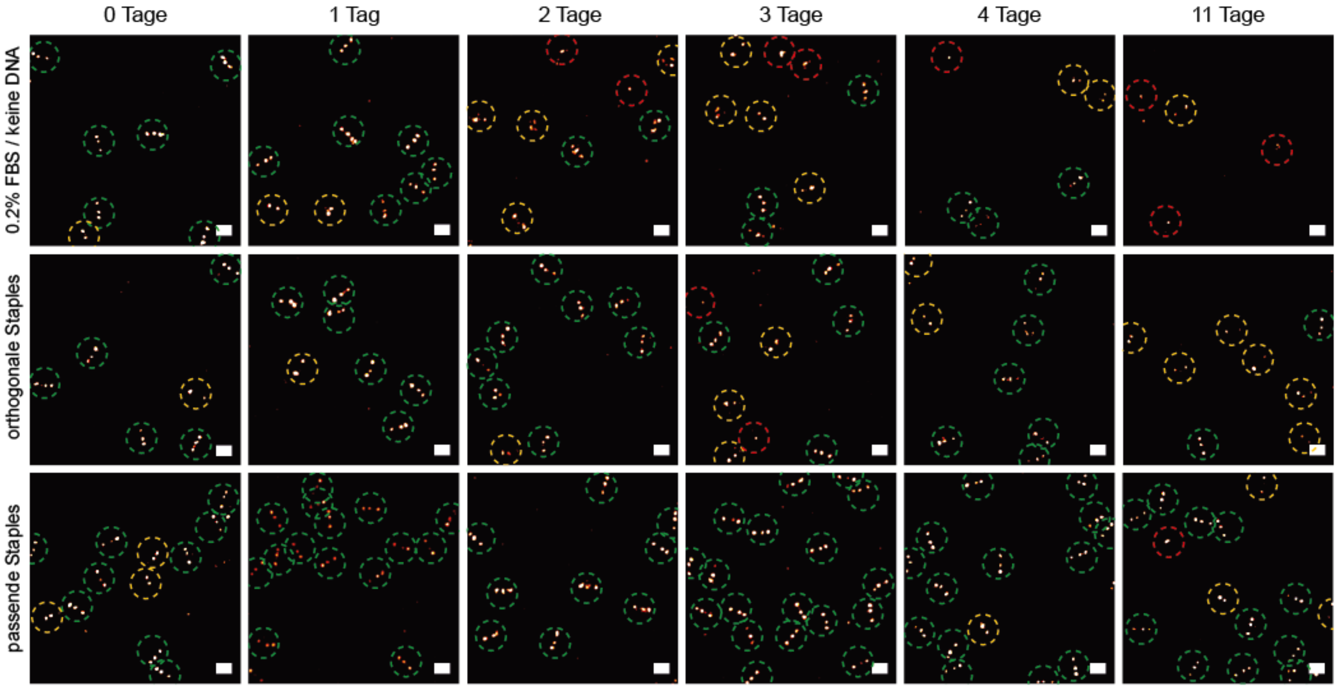


**Abbildung S10**. Beispielhafte DNA PAINT-Bilder von 12HB Triple-Spot-Nanolinealen in 0,2% FBS-Lösung (oben), mit hinzugefügten orthogonalen DNA-Strängen (Mitte) und mit hinzugefügten passenden Staple-Strängen des Nanolineals (unten) über 11 Tage Inkubation. Drei-Punkt-Nanolineale werden durch grüne, Zwei-Punkt- Nanolineale durch gelbe und Einzel-Punkt-Nanolineale durch rote Kreise hervorgehoben. Maßstabsbalken stellen 200 nm dar.

Beispielhafte DNA-PAINT-Bilder von immobilisierten Drei-Punkt-Nanolinealen (107 und 70 Markierungsabstände) in den drei verschiedenen Inkubationsbedingungen sind in Abbildung S10 gegeben. Während die in 0,2% FBS-Lösung inkubierte Probe eine schnelle Degradation zeigte, d.h. den Verlust von Markierungspunkten und eine abnehmende Oberflächendichte, führte die Zugabe von orthogonalen Oligonukleotiden zu einer sichtbaren Stabilisierung über die untersuchte Zeit von 11 Tagen. Die Zugabe eines Satzes der passenden unmodifizierten Staple-Strängen stabilisierte die Nanolineale in der degradierenden Umgebung erheblich, so dass auch nach 11 Tagen die Mehrheit der Nanolineale noch einen doppelten oder dreifachen Punkt aufwies. Für eine quantitativere Analyse der induzierten strukturbedingten Schäden haben wir die Anzahl der Lokalisationen und Auszeiten pro ausgewähltem Nanolineal extrahiert. Für eine Degradierung der DNA PAINT Nanolineale wird mit einer Abnahme der Anbindestellen im Laufe der Zeit gerechnet. Eine verringerte Anzahl von Anbindestellen führt zu einer geringeren Anzahl von Bindungsereignissen und damit auch zu Lokalisierungsereignissen gebundener Imager-Farbstoffe innerhalb einer vorgegebenen Zeit. Gleichzeitig erhöht eine abnehmende Anzahl von Anbindestellen die Zeit zwischen den Bindungsereignisen, d.h. der Auszeit. Um den Einfluss systematischer Schwankungen des verwendeten Weitfeldmikroskops auf die quantitative Analyse der auftretenden Schäden der DNA PAINT-Nanolineale zu verringern, haben wir die Degradationsstudie für jede Inkubationsbedingung dreimal durchgeführt. Die Mittelung über alle drei Reihen an Experimenten führte zu den Kurven in Abbildung 3F-G im Haupttext. Die extrahierten Lokalisationen und Auszeiten stimmen mit den den qualitativen Ergebnissen der DNA PAINT-Bildern überein.

Die Kombination der Ergebnisse der vorherigen NRO-Experimente, dass nur beschädigte Staple-Stränge mit unvollständiger Hybridisierung des Scaffold-Strangs effektiv ausgetauscht werden, mit der gezeigten Stabilisierung von 12HB-Nanolinealen durch intakter Staple-Stränge in Lösuzng, kommen wir zu dem Schluss, dass das gegebene Beispiel unsere Definition von Selbst-Heilung erfüllt. Das angewandte Selbstreparatursystem verbesserte die strukturelle Integrität der Nanolineale unter Verschleiß erheblich und könnte zur Realisierung langlebiger Superauflösungs-Nanolineale eingesetzt werden.

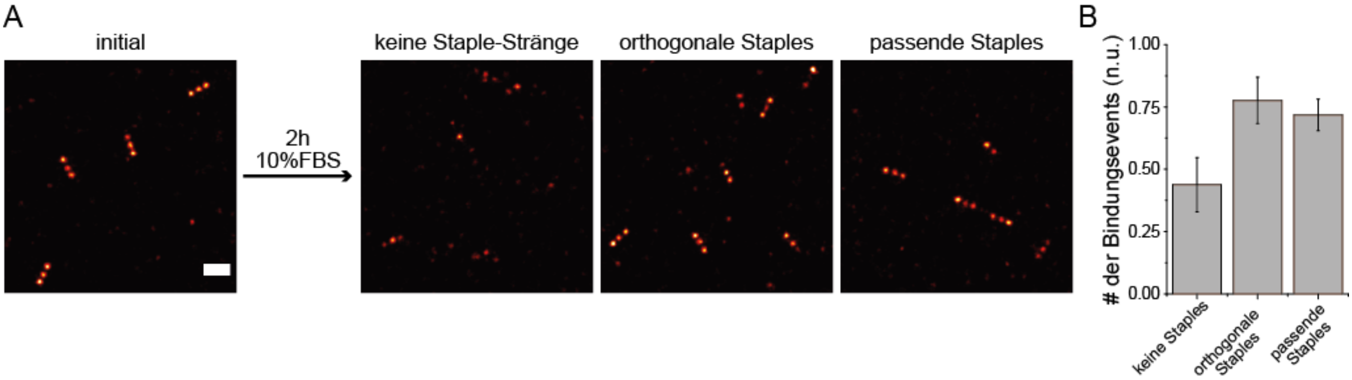


**Abbildung S11.** (A) Exemplarische DNA-PAINT-Bilder von 12HB-Drei-Punkt-Nanolinealen nach 2 h Inkubation in 10% FBS-Lösung ohne zusätzliche DNA-Stränge (links), mit hinzugefügten nicht passenden DNA-Strängen (Mitte) und mit hinzugefügten passenden DNA-Staple-Strängen (rechts). Maßstabsbalken stellen 200 nm dar. (B) Entsprechende extrahierte, gemittelte und normierte Anzahl von DNA PAINT-Bindungsereignissen pro Nanolineal nach 2h Inkubation in 10% FBS-Lösung mit und ohne hinzugefügten DNA-Strängen. Die Anzahl der Bindungsereignisse wurde bezüglich einer intakten Referenz normiert. Jede Säule stellt den Durchschnitt von drei unabhängigen Stichproben dar, Fehlerbalken stellen die Standardabweichung dar.

Um eine mögliche Selbstheilung auch bei höheren Schadensraten zu untersuchen, haben wir DNA PAINT-Studien mit DNA PAINT-Nanolinealen durchgeführt, die 2 Stunden lang in 10% FBS-Lösung inkubiert wurden. Um DNA PAINT-Bilder schneller aufzunehmen, haben wir eine 10 nM-Lösung des 6 nt ATTO655 Imager-Strangs in Tabelle S5 in einem 1x PBS Puffer mit 12,5 mM $MgCl_2$ verwendet. Mit diesen Parametern und einer Belichtungszeit von 25 ms pro Bild konnten wir innerhalb von 10 Minuten hochauflösende Bilder der immobilisierten 12HB-Nanolineale erzielen. Exemplarische DNA-PAINT-Bilder einer intakten Referenz und nach 2 h Inkubation in 10% FBS-Lösung sind in Abbildung S11A gegeben. Inkubation mit 10% FBS führte zu einer schnellen Degradation der immobilisierten 12HB-Nanolineale, während die Zugabe intakter nicht komplementärer oder komplementärer DNA-Staple-Stränge zu einer signifikanten Stabilisierung führte, so dass die Mehrheit der Nanolineale drei Punkte aufwies, die für eine intakte Struktur charakteristisch sind. Um einen quantitativeren Vergleich zu erhalten, haben wir die Anzahl der Bindungsereignisse pro DNA-Origami-Nanolineal nach der Inkubation extrahiert, gemittelt und normiert auf die Anzahl der Bindungsereignisse einer intakten Referenzstruktur (Abbildung S11B). Das kommerzielle Setup, das für diese Experimente verwendet wurde (ONI nanoimager S), ist ein geschlossenes System. Bei Messungen mit kontinuierlicher Anregung wie DNA PAINT führt der Wärmeeintrag durch die Laserbelichtung zu einer unkontrollierten Erwärmung des gesamten Mikroskopkörpers. Andererseits ist die Anzahl der Lokalisationen während eines DNA-PAINT-Experiments das Produkt von Bindungszeiten (in Einheiten einzelner Bildbelichtungszeiten) und Bindungsereignissen. Da die Bindungszeiten stark von der Temperatur abhängen, sind somit auch die Anzahl der Lokalisationen temperaturabhängig. Die Bindungsereignisse hingegen sind bei kleinen Temperaturschwankungen stabiler, da sie vor allem von der Konzentration des Bildstrangs in Lösung abhängen. Um einen temperaturunabhängigen Vergleich zu ermöglichen, haben wir daher die Anzahl der Bindungsereignisse pro ausgewähltem Nanolineal extrahiert und die verschiedenen Inkubationsbedingungen verglichen (Abbildung S11B). Zwei Stunden Inkubation mit 10% FBS führten zu einer Abnahme der Bindungsereignisse pro Nanolineal auf unter 50%. Die Zugabe eines Satzes nicht übereinstimmender Staple-Stränge und der Satz passender Staple-Stränge bei einer Gesamtkonzentration von 5 µM führten zu einer signifikanten Stabilisierung, was in einer finalen Anzahl der Bindungsereignisse von rund 75 % resultierte. Die vergleichbaren Ergebnisse für nicht übereinstimmende und übereinstimmende DNA-Staple-Stränge zeigten, dass die sakrifizielle Degradierung der hinzugefügten DNA der effektive Stabilisierungsmechanismus in 10% FBS ist. Ein selbstheilender Effekt konnte hingegen nicht beobachtet werden. Die hohe Konzentration an Nukleasen in der 10%igen FBS-Lösung induziert eine schnelle Degradierung der DNA-Origami-Nanostrukturen, die durch Selbstheilung nicht kompensiert werden kann wie in einer 0,2%-FBS-Inkubation über Tage gezeigt. Die Selbstheilung von DNA-Origami beschränkt sich somit auf niedrige Schadensraten, während die sakrifizielle Degradierung der zugesetzten DNA die Nanolineale auch bei schnellen Schadensraten effektiv stabilisieren kann.

Um die Stabilisierung des DNA-Origami-Nanolineals in 10% FBS durch sakrifizielle Degradierung von zugesetzten DNA-Staple-Strängen weiter zu untersuchen, haben wir der 10%igen FBS-Inkubationslösung verschiedene Konzentrationen von DNA-Staple-Strängen (Gesamtkonzentration von 5 µM, 500 nM und 50 nM) hinzugefügt. Beispielhafte Verteilungen der Anzahl der Bindungsereignisse, die aus ausgewählten DNA PAINT-Nanolinealen nach zwei Stunden Inkubation extrahiert wurden, sind in Abbildung S12 als Boxplots dargestellt. Für jede Konzentration an hinzugefügten DNA-Staple-Strängen führten die nicht komplementären und die passenden Staple-Stränge zu vergleichbaren Bindungsereignissen, was zeigt, dass keine Selbstheilung die Nanolineale stabilisiert hat. Die Zugabe von DNA-Staple-Strängen mit einer Gesamtkonzentration von 50 nM führte zu vergleichbaren Schäden wie keine Zugabe von DNA, während die Zugabe von Staple-Stränge mit 500 nM zu einer signifikanten Stabilisierung, aber zu verringerten Bindungsereignissen pro Nanolineal führte als die Zugabe von 5 µM DNA-Staple-Stränge. Die Ergebnisse deuten darauf hin, dass selbst bei nur 500 nM Lösung der hinzugefügten DNA Stränge DNA-Origami-Strukturen unter stark degradierenden Bedingungen wie in 10% FBS via sakrfizieller Degradierung signifikant stabilisiert werden könnten.

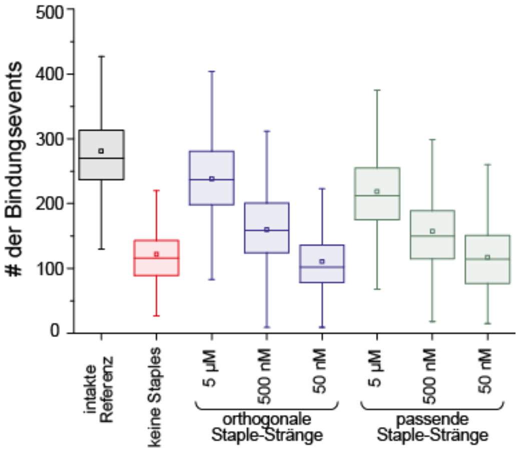


**Abbildung S12.** Exemplarische Box-Plots der Anzahl der Bindungsereignisse pro einzelnem Nanolineal. Intakte Referenzprobe in schwarz hervorgehoben, Probe in 10% FBS in rot, Proben mit 10% FBS und nicht passenden DNA-Staple-Strängen in blau und Proben inkubiert mit 10% FBS und passenden Staple-Strängen in grün. Quadrate geben den Durchschnitt, zentralen Linien den Median an. Die Boxlinien repräsentieren die 25% und 75% Quantile und die Balken den 1,5-fachen Interquartilbereich.

### 2.5. Selbstregeneration und Selbstheilung einer enzymatisch spaltbaren Helligkeitsmarkierung

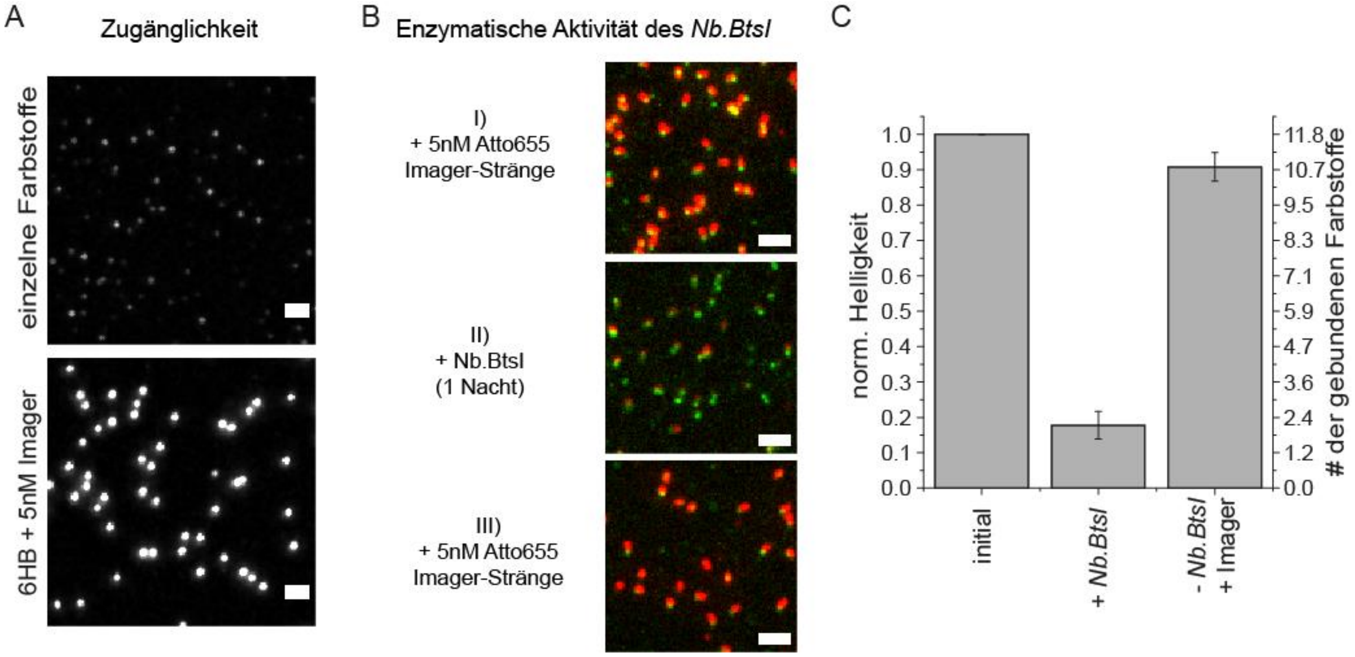


**Abbildung S13**. (A) Exemplarische TIRF-Bilder von immobilisierten einzelnen ATTO655-markeirten Oligonukleotiden (oben) und 6HB-Helligkeitsstandards mit 20 nt ATTO655 Imager-Strängen (unten) für Zugänglichkeitsstudien. Maßstabsbalken stellen 2 µm dar. (B) Zwei-Farben kolokalisierte TIRF-Bilder von intern markierten (ATTO532) 6HB Helligkeitsstandards extern markiert mit ATTO655-Markierungen, nach Inkubation mit *Nb.BtsI* und nach erneuter Markierung mit intakten ATTO655 Imager-Strängen. Maßstabsbalken stellen 2 µm da. (C) Extrahierte,gemittelte Helligkeitswerte initial, nach Zugabe von *Nb.BtsI* und nach erneuter Markierung mit intakten Imager-Strängend. Die Anzahl der gebundenen Imager-Stränge konnte anhand des Intensitätsvergleichs einzelner Farbstoffe und der 6HB-Helligkeitsstandards in (A) abgeschätzt werden. Die Daten stellen gemittelte normalierte Helligkeitswerte von drei unabhängigen Messungen dar, Fehler stellen die Standardabweichung dar.

Um die externe Markierungseffizienz des 6HB Helligkeitsstandards abzuschätzen, haben wir die Intensität einzelner immobilisierter ATTO655-markierter Oligonukleotide und des 6HB-Helligkeitsstandards verglichen (Abbildung S13A). Der entworfene Helligkeitsstandard wies eine Markierungsanzahl von 11,8 Farbstoffmolekülen (59%) auf. Als nächstes untersuchten wir die enzymatische Aktivität des verwendeten *Nb.BtsI,* indem wir die Helligkeitswerte vor und nach der Inkubation mit dem Restriktionsenzym verglichen. Daher haben wir die 6HB Helligkeitsstandards intern mit ATTO532 markiert (modifizierte Staple-Stränge in Tabelle S13), um Helligkeitsstandards mit vollständiger Markierungsabpaltung lokalisieren zu können. Spaltung der Markierungen durch *Nb.BtsI* über Nacht führte zu einem durchschnittlichen Helligkeitsverlust auf unter 20%. Dreifaches Waschen und Hinzufügen von Imager-Strängen stellte die Helligkeit zu über 90% wieder her, was darauf hindeutet, dass die Mehrheit der Anbindestellen nicht durch das Enzym beeinflusst wurden (Abbildung S13C).

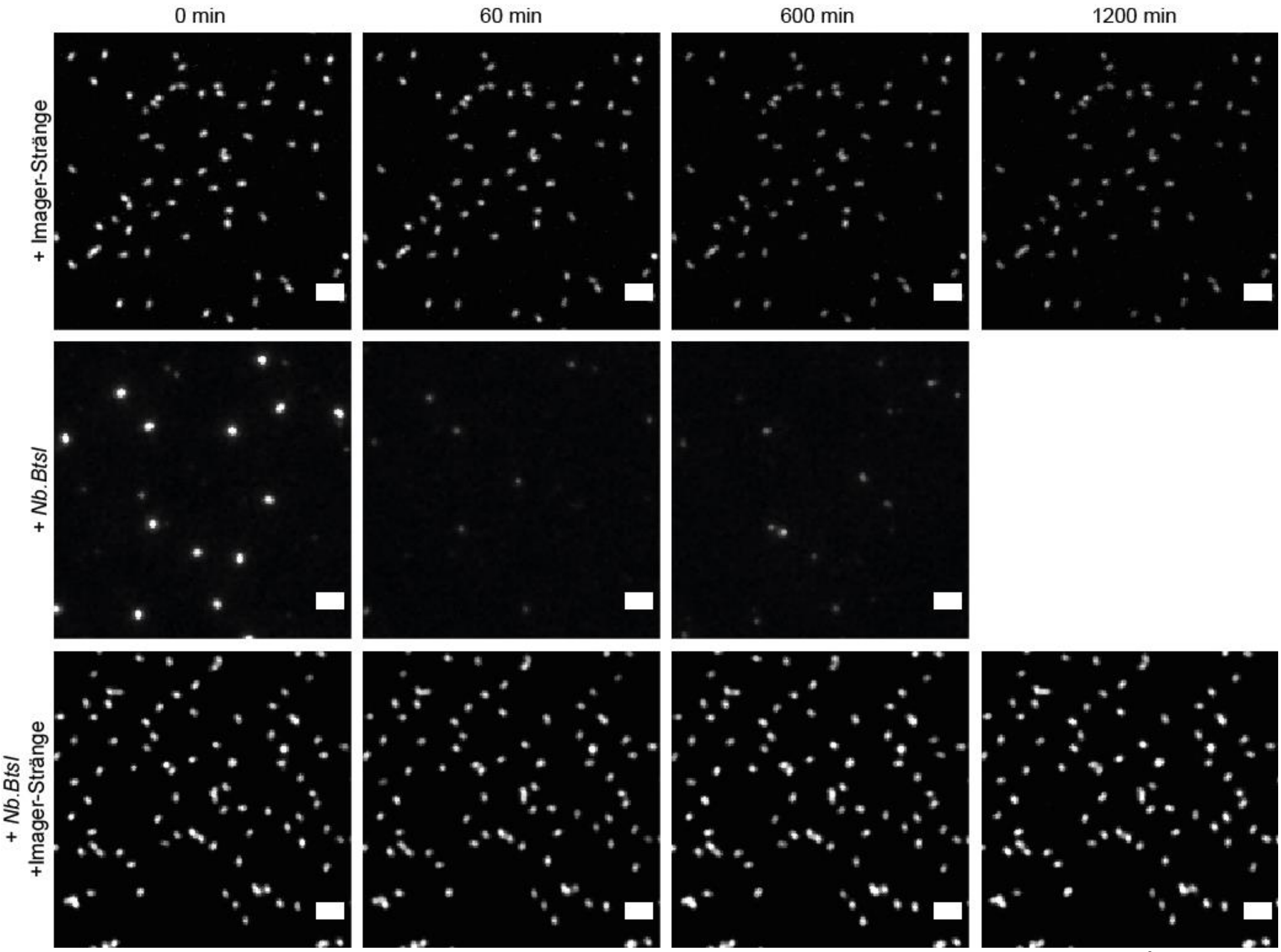


**Abbildung S14.** Exemplarische TIRF-Bilder über die Zeit für drei verschiedene Markierungsbedingungen unter Zeitraffer-Bildaufnahme (75 W/cm$^2$ für 100 ms alle 10 min). Oben: Photobleichen der permanenten Markierung in 5nM Imager-Strang-Lösung über die Zeit. Mitte: Enzymatischer Schaden, der durch *Nb.BtsI* induziert wird, führt zu einem schnellen Verlust des Helligkeitssignals. Unten: Die gleichzeitige Zugabe von *Nb.BtsI* und 5 nM Imager Strang Lösung führt zu einem stabilen Helligkeitssignal über die Zeit. Die transiente Bindung wird durch einen stationären Zustand zwischen enzymatischer Spaltung, schneller Dissoziation und Assoziation erreicht. Maßstabsbalken repräsentieren 2 µm.

Um die Konzepte der Selbstregeneration und Selbstheilung zu untersuchen und zu verdeutlichen, haben wir drei verschiedene Inkubationsbedingungen mittels Zeitraffer TIRF-Aufnahmen (3 mW bei 640 nm alle 10 min) verglichen. Abbildung S14 zeigt exemplarische TIRF-Bilder von immobilisierten 6HB Helligkeitsstandards über die Zeit inkubiert mit einer 5 nM Lösung von Imager-Strängen (oben), mit einer Lösung von *Nb.BtsI* (Mitte) und einer Lösung mit 5 nM Imager-Strängen und *Nb.BtsI* (unten). Während die alleinige Zugabe des Restriktionsenzyms die Markierungen spaltete und zu einem schnellen Verlust des Helligkeitssignals nach 1 Stunde führte, konnte die Inkubation in einer 5 nM Imager-Strang-Lösung das langsame Photobleichen über die Zeit nicht kompensieren. Die gleichzeitige Zugabe des Restriktionsenzyms *Nb.BtsI* und der Imager-stränge führte zu einer dynamischen stabilen Helligkeitsmarkierung, welche auch nach 20 h keine Photobleicheffekte unter den verwendeten Zeitrafferbedingungen zeigte.

Zusammenfassend lässt sich sagen, dass das angewandte System die Bausteine der Helligkeitsfunktion, d.h. die Imager-Stränge, unspezifisch in Bezug auf das Photobleichen wiederherstellt, da geblichene und photoaktive Farbstoffe gleichermaßen ausgetauscht werden. In Bezug auf die enzymatische Schädigung durch das Restriktionsenzym dissoziieren nur beschädigte Einheiten schnell genug als zwei 10-nt-Fragmente, welche durch einen intakten Imager-Strang in der Lösung ersetzt werden können. Die Selbstreparatur in diesem Beispiel zeigt, dass Selbstregeneration und Selbstheilung gleichzeitig innerhalb eines Systems auftreten können, wenn verschiedene Schadensquellen vorliegen.

## 3. Anhang

**Tabelle S15**. Unmodifizierte Staple-Stränge des 12HB DNA Origami. Sequenzen sind vom 5'- zum 3'-Ende angegeben. Die Zahlen für das 5'- Ende 3'-Ende der Staple-Stränge stellen die Helixnummer in der entsprechenden caDNAno-Datei dar. Die Zahl in Klammern stellt die Start- und Endposition des Staple-Strangs in der entsprechenden Helix dar.

| Unveränderte Staple-Stränge | 5'-Ende | 3'-Ende |
|---|---|---|
| AAAGGGCGCTGGCAAGTATTGGC | 11[681] | 10[668] |
| GCGCCTGAATGCCAACGGCCCAGCCTCCCGCGTGCCTGTTCTTCTTTTT | 7[42] | 8[25] |
| TTGACGGGGAAAGCTTCACCAGAATGGCATCACT | 11[651] | 6[658] |
| CATTCAACCCAAAAATGTAGAACCCTCATGAATTAGTACAACC | 9[147] | 5[160] |
| TCAGAGGTGTGTCGGCGAGAATGAGTGCACTCTGTGGT | 4[60] | 7[62] |
| GGCATAAGCGTCTTCGAGAAACGCA | 8[466] | 9[482] |
| TACATAAATCTGGGCACTACAACT | 8[634] | 9[650] |
| CAATCCAAAATACTGAACAGTAG | 3[457] | 10[458] |
| CATAGTTAATTTGTAAATGTCGC | 3[541] | 10[542] |
| GAACAAGAGTCCACCAATTTTTTTTTTTCGTAGG | 11[483] | 6[490] |
| TTGAAGCCCTTTTTAAGAAAAAGT | 7[441] | 7[463] |
| AAGCACAGAGCCTAATTATTGTTAGCGATTAAGACTCCTT | 7[464] | 8[448] |
| GATGTTTTTTTTTCACCA | 10[289] | 11[302] |
| GGTCACGCCAGAGGAGKTTAG | 3[373] | 10[374] |
| TGAACAGCTTGATACCGATAGTT | 8[363] | 8[341] |
| AAAATTCCATTCAGGCTTGTGCAAAA | 8[256] | 9[272] |
| TCCCATCCTAATGAGAATAACAT | 0[496] | 0[474] |
| ATCAGCGGGGGGCTTTCAGAG | 3[56] | 3[78] |
| TTCGCTATTCGCACACAACAAAGTTAATTTCATCTTC | 5[539] | 4[546] |
| TTGAGAATATCTCTCTCTCTCACTCATCGAGAACA | 5[497] | 4[504] |
| GGGCGTGAAATTAGCGCCATTCGC | 8[130] | 9[146] |
| GGCGCCCCGCCGAATCCTGAGAAGTGAGGCCGATTAAAGG | 3[667] | 0[665] |
| TTTTTTTTTTAATAAAGTAATTC | 3[476] | 3[498] |
| AAATCAGCCAGTAATAACACTATTTTTGAAGCCTTAATC | 7[506] | 8[490] |
| AGCACTAAATCGGATCGTATTTAGACTTATATCTG | 11[609] | 6[616] |
| GGTGCCGTCGAGAGGTGAT | 8[405] | 8[383] |
| GTCAGAATCAGGCAGGATTCGCG | 3[205] | 10[206] |
| TTTTTTATAACGTGCTTTCCTCTTTATAACAGTACTAT | 2[698] | 3[678] |
| AGACGGGAGAATTGACGGAAATT | 0[454] | 0[432] |
| TAAGCCAGAGAGCGAGAGAAACTCGATAGCCGAACAAAA | 4[480] | 7[482] |
| CGCCTGACGGTAGAAAGATTCTAATGCAGATACAT | 5[245] | 4[252] |
| CAGTCTTGATTTTAAGAACTCAACGTTGCGTAT | 0[263] | 11[272] |
| CATAGAATTTGCGGTTTGAAAGAGGA | 8[298] | 9[314] |
| GCGCAGCGACCAGCGATTATATCATCGCCTGAT | 5[287] | 4[294] |
| TTTTTAAAACGCTCATGGAAATA | 8[698] | 8[679] |
| AATCAGTTAAAACGGGAGAAA | 3[121] | 10[122] |
| AGACAACCTGAACAGTATTCGAC | 3[625] | 10[626] |
| TTTGCAACCCTCTCTGGCGGTGGTGAGGTTTCAGTTGATCCTTTTT | 3[25] | 10[29] |
| TGCAACACTATCATAACCCTCGT | 7[231] | 7[253] |
| AACGAACCTCCCGACTTGCGGGA | 8[531] | 8[509] |
| CCGAACGGTGTACAGAGGCG | 8[321] | 8[299] |
| ATTCAAGGGGAAGGTAAATGTGGCAAATAAATC | 0[431] | 11[440] |
| AGBACCTACAAGGTTGAGGCA | 3[350] | 3[372] |

| | | |
|---|---|---|
| TAAATCGGTTGGTGCACATCAAAAAAAAAAA | 6[153] | 2[140] |
| AGACGGCGAACGGCGAG | 10[667] | 11[680] |
| CCCTTCATATAAAAAACGTAGAGCCTTAAAGGTGAATTA | 11[429] | 0[413] |
| AACTTTAATCATGGGTAGCAACG | 3[266] | 3[288] |
| ACCATCACCCAAAAAACAGTTCATTTGATTCGCC | 11[567] | 6[574] |
| TGCCTAATGAGTGAGAAAAGCTCATATGTAGCTGA | 11[147] | 6[154] |
| TTTTTTGGTAATGGGTAACCATCCCACTTTTT | 1[21] | 2[25] |
| GGAGCAGCCACCACCCTTCGCATAACGACAATGACAACAA | 7[338] | 8[322] |
| AAAAGTGTCAGCAACAATTGCAGGCGCT | 6[69] | 2[56] |
| GGTTTGCGCATTTTAACGCGAGGCGT | 8[508] | 9[524] |
| AAAAGAATAGCCCGATACATACGCAGTAGCTATC | 11[441] | 6[448] |
| TTTCACGAGAATGACCATTTTCATTTGGTCAATAACCTGT | 7[212] | 8[196] |
| TCGGTCATACCGGGGGGTTCTGC | 8[69] | 8[47] |
| CCTCCGAAATCGGCAAAAT | 10[415] | 11[428] |
| TTCCATTGACCCAAAGAGGCTTTGAGGA | 2[307] | 3[307] |
| ACGCGTGGCTCTAAGACGACGACAATA | 2[517] | 3[517] |
| GTCCGTCCTGCAAGATCCGGATTCTCGCGCATTGGACGA | 9[105] | 5[118] |
| GTCAGTCGTTTAACGAGATGGCAATTCA | 6[615] | 2[602] |
| GAGCTTAAGAGGTCCCAATTCTGCAATTCCATATAACAGT | 4[228] | 7[230] |
| GCAGCACTTTGCTCTGAGCCGGGTCACTGTTGCCCTGCGGCTTTTT | 10[48] | 0[21] |
| TACCTGGTTTGCCCCAGCA | 10[373] | 11[386] |
| AATGCTGTAGCTGAGAAAGGCCG | 4[209] | 4[187] |
| CTATTAAAGAACGTGGA | 10[499] | 11[512] |
| CGGTAGTACTCAATCCGCTGCTGGTCATGGTC | 0[53] | 11[62] |
| CTTGAAAACACCCTAACGGCATA | 3[247] | 10[248] |
| AAGTAAGAGCCGCCAGTACCAGGCGG | 8[382] | 9[398] |
| AAAAGATAGGGTT | 10[457] | 11[470] |
| TTCGCCATAAACTCTGGAGGTGTCCAGC | 2[55] | 3[55] |
| AGGGCGAAAAACCGATTTAACGTAGGGCAAATACC | 11[525] | 6[532] |
| CCCACATGTGAGTGAATAACTGATGCTTTTAACCTCCGGC | 11[555] | 0[539] |
| TTTTTAGGAGCGGGCGCTAGGAGGAAGAAAAGCGAATTTTT | 10[702] | 11[702] |
| TGCCATACATAAAGATTAACTGAACACCAACAGCCGGAATAG | 9[441] | 5[454] |
| TTTTTCCGGTGCAGCACCGATCCCTTACACTTGCC | 5[29] | 4[52] |
| ACAGCTGATTGCCCGTCGCTGCGCCCACACGTTGA | 11[315] | 6[322] |
| ATTAAAATAAGTGCGACGATTGGCCTTG | 2[391] | 3[391] |
| AAAACGAAAGAGGCTCATTATAC | 0[286] | 0[264] |
| TGTCCAAGTACCAGAAAACCCCAG | 3[499] | 10[500] |
| TTACCAATAGGCTTGCAGTGCGGAAGTTTAGACTGGATA | 7[254] | 8[238] |
| TTAGTGTGAATCCCTCTAATAAAACGAAAGAACGATGAATTA | 9[231] | 5[244] |
| ATCAGAGCCTATACGGGGTCTTAATGCCCCCTGC | 5[371] | 4[378] |
| TTACCTCTTAGCAAATTTCAACCGATTG | 6[447] | 2[434] |
| AAAACGGAATACCCAAAAGAACT | 8[489] | 8[467] |
| GTCCACGCGCCACCTCCGTGAAACA | 11[364] | 6[364] |
| TTTTTATCCAGCGCAGTGTCACTGC | 7[21] | 7[41] |
| GATGAATAAATCCTGTAGGTGAGGCGGTAGCGTAAGTCCTCA | 9[609] | 5[622] |
| GCTAAATCGGTTTGACTATTATA | 3[182] | 3[204] |
| CAGCTTTGAATACCAAGTTACAA | 7[567] | 7[589] |
| GGTTGCTTTGACGAGCACTTTT | 3[679] | 3[698] |
| CATGCCAGTGAGCGCTAATATCCAATAATAAAGAGC | 5[455] | 4[462] |

| | | |
|---|---|---|
| TATGCATTACAGAGGATGGTTTAATTTC | 2[265] | 3[265] |
| ACTGCCCGCTTTCCTGAAAAGCTATTATAAATA | 11[189] | 6[196] |
| TGATTTAGAAAACTCAAGAGTCAATAGT | 6[573] | 2[560] |
| TGGGCGCGCGGGTGATTCATTAGAGTAACCTGCTC | 11[273] | 6[280] |
| TGCAACTCAAAGGCCGTACCAAAAACACA | 6[195] | 2[182] |
| AAATAGGTAATTTACAAATAAGAAACGA | 2[475] | 3[475] |
| TGTTCCAACGCTAACGAACAAGTCAGCAGGGAAGCGCATT | 11[471] | 0[455] |
| GTGCCTGCTATAACAGGGAGAGAGTTTCAAAGCGAACCA | 11[219] | 0[203] |
| GTTTGATGGTGGTTCAGAACCCCGCCTCACAGAAT | 11[399] | 6[406] |
| TCACCGTCACCGGCGCACTCT | 0[412] | 0[390] |
| AGACGTCGTCACCCTCAGATCTTGACGCTGGCTGACCTTC | 7[296] | 8[280] |
| TTTAGCAAACGCCACAATAACTATTCCCTTATAAATGG | 9[525] | 5[538] |
| AGCGTATCACACACCACAGACCCGCAGTTGCAGCAAGCG | 0[347] | 11[363] |
| GTATGTGAAATTGTTATCC | 10[79] | 11[92] |
| CCGAACTTTAATAAAAGCAAAGCGGATT | 2[223] | 3[223] |
| GTGAGTTAAGGCCGCTGACACTCATGAAGGCACCACAACCT | 11[303] | 0[287] |
| GCGCCCGCACCCTCGAGGTGAATT | 8[340] | 9[356] |
| ACAGTTTTTAGATTTCAATTACCGTCGCAGAGGCGAATT | 4[606] | 7[608] |
| TTTAGAACGCGAATTACTAGAAAACTATAAACACGGAAT | 4[564] | 7[566] |
| TGACCTAAATTTTTAAAAAAGT | 4[545] | 4[523] |
| TAAAGAGGCAAAATTTTATAA | 3[163] | 10[164] |
| GTTTACCGCGCCCAATAGCAAGC | 7[483] | 7[505] |
| TACCGGGATAGCAATGAATATAT | 3[331] | 10[332] |
| AAATTGTGTCGAGAATACCACAT | 4[293] | 4[271] |
| AAATGCGTTATACAAATTCTTAC | 8[573] | 8[551] |
| CAGATATAGGCTTGAACACGTTAGTAAAGCCCAAAAATTT | 9[315] | 5[328] |
| TAAGATCTGTAAATCGTTGTTAATTGTAAAGCCAACGCTC | 7[548] | 8[532] |
| CATTCTATCAGGGCGATGG | 10[541] | 11[554] |
| CTCCAATTTAGGCAGAGACAATCACACAAAAATAATAATA | 11[513] | 0[497] |
| GAGACAAAGATTATCAGGTCATTGACGAGAGATCTACAAA | 4[186] | 7[188] |
| AGGGACAAAATCTTCGCGCCAAAGAC | 2[433] | 3[433] |
| AAAATTTTTTAAAGAGCAAAAAAAA | 8[592] | 9[608] |
| CATCGGGAGAAATTCAAATAT | 4[587] | 4[565] |
| ATCATTTACATAAAAGTATCAAATTATAAGAAACTTCAATA | 9[567] | 5[580] |
| GCTACGACAGCAACTAAAAAACCG | 3[289] | 10[290] |
| TTAGGTTGGGTTATAGATAAGTC | 0[538] | 0[516] |
| TATTGCCTTTAGCGTCAGACTGT | 7[399] | 7[421] |
| TTTTTCCGGGTACCCTCTCGAATTCGTAATCTGGTCA | 11[29] | 10[49] |
| CTAAAGACTTTTAGGAACCCATG | 3[308] | 3[330] |
| GTGGAACGACGGGCTCTCAACTT | 3[79] | 10[80] |
| TCAGGTGAAATTTCTACGGAAACAATCG | 6[111] | 2[98] |
| AAGACGCTGAGACCAGAAGGAGC | 3[560] | 3[582] |
| AGCAGTCGGGAAACCTGTC | 10[205] | 11[218] |
| AACAACATGTTCATCCGAGAAAA | 3[518] | 3[540] |
| ATAATGAATCCTGAGATTACCATGTGACAAAAACTTATT | 9[483] | 5[496] |
| GAGGTAACGTTATTATATAAACACAAATAATGGAAGGGT | 11[597] | 0[581] |
| ACCGCATTCCAACGGTATTCTAAGCGAGATATAGAAGGCT | 4[522] | 7[524] |
| CAGCATCAACCGCACGGCGGGCCGTT | 8[46] | 9[62] |
| GCTCAAGTTGGGTAACGGGGAAAAATTTGTGAGAGATA | 11[93] | 0[77] |

| | | |
|---|---|---|
| GGAATCGGAACATTGCACGTTAA | 3[583] | 10[584] |
| ATAAGAAGCCCACAAACTTGAGCCATTATCAATACATCAGT | 9[399] | 5[412] |
| GGCGACACACCCTCTCAGGTTACTGTACGTTCCAGTAAA | 11[387] | 0[371] |
| CATGTCAGAGATTTGATGTGAATTACCT | 6[279] | 2[266] |
| AATAGCTGTCACACGCAACGGTACGCGCGCTTAATGTAGTA | 9[651] | 5[664] |
| GCAGCACCGTAAGTGCCCGTATA | 4[419] | 4[397] |
| ATGAATCCCAGTCACGATCGAACGTGCCGGCCAGAGCACA | 7[86] | 8[70] |
| TATGTGATAAATAAGGCGTTAAA | 7[525] | 7[547] |
| TTAATGAATCGGCCATTCACACAATACGCATAGT | 11[231] | 6[238] |
| ATTCTTTTCATAATCAAAATCAC | 8[447] | 8[425] |
| AATCGTTGAGTAACATTGGAATTACCTAATTACATTTAAC | 7[590] | 8[574] |
| ATTTTGCCAGAGGGTATATAGT | 8[279] | 8[257] |
| AGCGCCACCACGGAATACGCCTCAGACCAGAGCCACCACC | 7[422] | 8[406] |
| AAAAAAGGCAGCCTTTACAATCTTACCAGTTTG | 0[473] | 11[482] |
| TAATCGTAGCATTACCTGAGAGTCTG | 8[172] | 9[188] |
| CAAGTGCTGAGTAAGAAAATAAATCCTC | 6[405] | 2[392] |
| GGCTAAAGTACGGTCTGGAAG | 7[189] | 7[211] |
| CCTACATACGTAGCGGCCAGCCATTGCAACAGGTTTTT | 8[678] | 9[698] |
| CTATTTCGGAACGAGTGAGAATA | 4[377] | 4[355] |
| TCAACATCAGTTAAATAGCGAGAGTGAGACGACGATAAAA | 4[270] | 7[272] |
| AATAACGCGCGGGGAGAGG | 10[247] | 11[260] |
| AAGAGATTCATTTTTTAAGAGGAAGC | 6[237] | 2[224] |
| CAAATGGTTCAGAAGAACTAGTAGAT | 8[214] | 9[230] |
| AAAAGGGCGACAATTATTTATCC | 3[434] | 3[456] |
| ATAGCTGTCTCTGGAACGTCCATAACGCCGTAAAA | 11[63] | 6[70] |
| TGTAGGGGATTTAGTAACACTTTTC | 2[349] | 3[349] |
| AAAAATCTACGTGCGTTATATT | 0[244] | 0[222] |
| AGAGTTTATACCAGTAGCACCTGAAACCATCGATA | 5[413] | 4[420] |
| GTGTATTAAGAGGCTGAGACTCC | 7[357] | 7[379] |
| GAAGTCACACACAAATGGCAAAGAATACTCGGAAGAGAATCC | 9[273] | 5[286] |
| CGGTTAACAAAGCTGCTGTACAACAACAAGGACGTTGGGAAG | 11[261] | 0[245] |
| ACTACCTTTAAACGGGTACAGGGAGACGGGCA | 0[305] | 11[314] |
| AATCCAAAAAAAGGCTCCAAAA | 7[315] | 7[337] |
| GAGAGCCTGAGACGCATTTTCTGTAACGATCTAAAGTT | 11[345] | 0[329] |
| AAATCCCCGAAACAATTCATGAGGAAGT | 6[321] | 2[308] |
| TACCTAATCAAAATCATTCAATATTACGTGA | 0[557] | 11[566] |
| GTATACAGGTAATGTGTAGGTAGTCAAATCACCAT | 5[161] | 4[168] |
| AACGTTGTAGAAACAGCGGATAGTTGGGCGGTTGT | 5[77] | 4[84] |
| GTTTATGTCACATGGGAATCCAC | 3[415] | 10[416] |
| ATATTCACAAACAAATTCATATG | 3[392] | 3[414] |
| GACCGGAAGCAATTGCGGGAGAA | 0[202] | 0[180] |
| TCACACAGAACCACCACTACTCAGGTAGCCCGGAATAGG | 7[380] | 8[364] |
| AGCCTCCCCAGGGTCCGGCAAACGCG | 8[88] | 9[104] |
| TTCATTTTCTGCTAAACAACTGAACAACTAAAAAAGGA | 5[329] | 4[336] |
| TCGTTCACCGCCTGGCCCT | 10[331] | 11[344] |
| CGGAAGCACGCAAACTTATTAGCGTT | 8[424] | 9[440] |
| GAGCAAGGTGGCATTTACTCCAACAGGTTCTTTACGTCAACA | 9[189] | 5[202] |
| ATTGCGAATAATGTACAACGGAG | 4[335] | 4[313] |
| CTTTTTTTCGTCTCGTCGCTGGC | 8[111] | 8[89] |

| | | |
|---|---|---|
| GACCGTCGAACGGAAGCTAATGCAGA | 6[531] | 2[518] |
| GCGTCATACATGCCCTCATAGTT | 0[370] | 0[348] |
| GAAAGTTCAACAATCAGCTTGCTTAGCTTTAATTGTATCG | 4[354] | 7[356] |
| TGTAAATCATGCTCCTTTTGATAATTGCTGAAT | 5[203] | 4[210] |
| TTCACCTAGCGTGGCGGGTGAAGGGATACCAGTGCATAAAAAA | 9[63] | 5[76] |
| ATTTGCCAAGCGGAACTGACCAACGAGTCAATCATAAGGG | 4[312] | 7[314] |
| TAGAACCTACCAGTCTGAGAGAC | 0[580] | 0[558] |
| GGGTTACCTGCAGCCAGCGGTTTTT | 4[51] | 4[29] |
| GAATTATCCAATAACGATAGCTTAGATT | 2[559] | 3[559] |
| TTGTCGTCTCTTACTACAATGCC | 0[328] | 0[306] |
| ACTACTTAGCCGGAACGAGGCGC | 7[273] | 7[295] |
| TTTTTGTCCATCACGCAAATTCCGAGTAAAAGAGTCTTTTTT | 4[702] | 5[702] |
| TTTTTCGGGAGCTAAACAGGTTTTTAGAATCAGAGTTTTT | 0[694] | 1[694] |
| AATCATAATAACCCGGCGTCAAAAAAGA | 6[489] | 2[476] |
| AGCAAGCCGTTTAAGAATTGAGT | 4[503] | 4[481] |
| AACAGAGTGTGTGGGTGTGTGCTCACAGAAGGATTAGGAT | 4[396] | 7[398] |
| CCAGCCAACTTCTGATTGCCGTTTTGGGTAAAGTTAAAC | 4[102] | 7[104] |
| TGAAATTGTTTCAGGGAACTACAACGCC | 6[363] | 2[350] |
| GCCCGCACAGGCGGCCTTTAGTG | 7[63] | 7[85] |
| CAGTAAGAACCTTGAGCCTGTTTAGT | 8[550] | 9[566] |
| ACCAAATTACCAGGTCATAGCCCCGAGTTTTCATCGGCAT | 4[438] | 7[440] |
| TCTTATACTCAGAAAGGCTTGATGATATTGACACGCTATT | 9[357] | 5[370] |
| GCCTTATACCCTGTAATACCAATTCTTGCGCTC | 0[179] | 11[188] |
| TTTTTGCGTCCGTGCCTGCATCAGACGTTTTT | 9[25] | 6[21] |
| TTATGGCCTGAGCTCTCAGAGCATAAAA | 2[181] | 3[181] |
| CGAGCACAGACTTCAAATACCTCAAAAGCTGCA | 0[221] | 11[230] |
| GCATCAAAAAGAAGTAAATTGGG | 3[224] | 3[246] |
| TAAGTAGAAGAACTCAAACTATCG | 7[651] | 7[673] |
| ATTTGGCAAATCAACAGTTGAAA | 7[609] | 7[631] |
| GTTGAAACAAACATCAAGAAAAC | 8[615] | 8[593] |
| GAATTGTAGCCAGAATGGATCAGAGCAAATCCT | 0[389] | 11[398] |
| GCTTGACCATTAGATACATTTCG | 8[237] | 8[215] |
| CTGAAAACCTGTTTATCAAACATGTAACGTCAA | 0[515] | 11[524] |
| GACTTTCTCCGTGGCGCGGTTG | 0[76] | 0[54] |
| ACACAACATACGGATGGCTATTAATCGGCC | 11[105] | 6[112] |
| TTTTTAACAATATTACCCTGGTAATCCAGTTTTT | 6[694] | 7[694] |
| TGCCTGAACGACAAATGAATGCGCGAACT | 6[657] | 2[644] |
| CAAATATCAAACCAGATGAAT | 4[629] | 4[607] |
| CAATATGATATTGATGGGCGCAT | 4[167] | 4[145] |
| TTCTGGAATAATCCTGATTTTGCCCGGCCGTAA | 0[599] | 11[608] |
| TTAACAAGAGAATCGATGAACGG | 8[195] | 8[173] |
| GGGCCGGAAGCATAAAGTG | 10[121] | 11[134] |
| GTTTGAGGGGACCTCATTTGCCG | 4[125] | 4[103] |
| GTATTAGAGCCGTCAATAGATAA | 8[657] | 8[635] |
| GCTAATGCCGGAGAGGGTAGCTA | 7[147] | 7[169] |
| TACTTCTTTGATAAAAATCTAAAA | 4[671] | 4[649] |
| GAAAGATCGCACTCCAGCCAGCT | 7[105] | 7[127] |
| TCAGGCTGCGCAACTGTTGGGAA | 8[153] | 8[131] |
| ATACCCTTCGTGCCACGCTGAACCTTGCTGAACCT | 5[623] | 4[630] |

| | | |
|---|---|---|
| CATAATATTCCGTAATGGGATCCGTGCATCTGCCA | 5[119] | 4[126] |
| TTTTTATCCAATAAATCTCTACCCCGGTAAAACTAGCATG | 7[170] | 8[154] |
| CCGATAATAAAAGGGACTTAACACGCGAACCAGCAG | 11[639] | 0[623] |
| CATCAGCGTCTGGCCTTCKAVAGGAACCTGGGG | 0[137] | 11[146] |
| GGAATAACAGAGATAGACATACAAACTTGAGGATTTAGAA | 7[632] | 8[616] |
| CCGGAAGACGTACAGCGCCGCGATTACAATTCC | 0[95] | 11[104] |
| TTCGCGGATTGATTGCTCATTTTTTAAC | 2[139] | 3[139] |
| TAAAGGATTGTATAAGCGCACAAACGACATTAAATGTGAG | 11[135] | 0[119] |
| GATAAAAATTTTTAGCCAGCTTT | 0[160] | 0[138] |
| GATAGTGCAACATGATATTTGAATGG | 2[643] | 3[643] |
| GGATAACCTCACAATTTTTGTTA | 3[98] | 3[120] |
| TCAATAATAAAGTGTATCATCATATTCC | 2[601] | 3[601] |
| CAATAGGAACGCAAATTAAGCACAA | 3[140] | 3[162] |
| GCGAAAGACGCAAAGCCGCGGGAAC | 2[97] | 3[97] |
| TTCCGAATTGTAAAAGTGTCGCCAGCTGGCGGGCCT | 7[128] | 8[112] |
| ACATCATTTAATTGCGTAGAAACAGTACCTTTTA | 5[581] | 4[588] |
| AAGATAAAAACAGTTGGATTATAC | 0[622] | 0[600] |
| AACACCCTAAAGGGAGCCC | 10[625] | 11[638] |
| GCATCGAGCCAGATATCTTTAGGACCTGAGGAAGGTTATC | 4[648] | 7[650] |
| CGTAAAGGTCACGAAACCAGGCAATAGCCCGCGCTGGT | 4[144] | 7[146] |
| CGAGTAACAACCTTTTACCAGTC | 0[118] | 0[96] |
| GCCTTACGCTGCGCGTAAAATTATTTTTTGACGCTCAATC | 7[674] | 8[658] |
| CCGAACCCCCTAAAACATCGACCTTTAGAGC | 0[641] | 11[650] |
| TGCGTACTAATAGTAGTTGAAATGCATATTTCAACGCAAG | 11[177] | 0[161] |
| GATTTTAGACAGGCATTAAAAATA | 0[664] | 0[642] |
| TGATTATCAGATATACGTGGCAC | 3[602] | 3[624] |
| TGGCAAGTTTTTTGGGGTC | 10[583] | 11[596] |
| TCAGCTAACTCACATTAAT | 10[163] | 11[176] |
| CTATTAGTCTTTCGCCGCTACAG | 3[644] | 3[666] |
| AACGCCAAAAGGCGGATGGCTTA | 4[251] | 4[229] |
| AAGAAACAATGACCGGAAACGTC | 4[461] | 4[439] |
| GTACATCGACATCGTTAACGGCA | 4[83] | 4[61] |
| ATACCACCATCAGTGAGGCCAAACCGTTGTAGCAA | 5[665] | 4[672] |

**Tabelle S16**. Unmodifizierte Staple-Stränge des NRO DNA Origami. Sequenzen sind vom 5'- zum 3'-Ende angegeben. Die Zahlen für das 5'- Ende 3'-Ende der Staple-Stränge stellen die Helixnummer in der entsprechenden caDNAno-Datei dar. Die Zahl in Klammern stellt die Start- und Endposition des Staple-Strangs in der entsprechenden Helix dar.

| Unveränderte Staple-Stränge | 5'-Ende | 3'-Ende |
|---|---|---|
| CATAAATCTTTGAATACCAAGTGTTAGAAC | 17[224] | 19[223] |
| AAGCCTGGTACGAGCCGGAAGCATAGATGATG | 20[143] | 19[159] |
| TCATTCAGATGCGATTTTAAGAACAGGCATAG | 5[96] | 7[95] |
| GCCATCAAGCTCTTTTTTAACCACAAATCCA | 16[143] | 15[159] |
| TATAACTAACAAAGAACGCGAGAACGCCAA | 16[175] | 14[176] |
| TTGCTCCTCTAAATATCGCGTTTGAGGGGGT | 10[111] | 8[112] |
| GTATAGCAAACAGTTAATGCCCAATCCTCA | 1[224] | 3[223] |
| AAAGTCACAAAAAACACAGCCAGCGTTTTA | 9[224] | 11[223] |
| GGCCTTGAAGAGCCACCCTGAGAAACCAT | 3[192] | 5[191] |
| TTAACGTCTAACATAAAACACAGGTAACGGA | 10[175] | 8[176] |
| AGTATAAAGAGAGCTAATGCAGATGTCTTTC | 14[239] | 12[240] |

| | | |
|---|---|---|
| GATGTGCGCAGGAAGATCGCACAATGTGA | 18[79] | 16[80] |
| TCAAATATAACCTCCGGCTTAGGTAACAATTT | 15[192] | 17[191] |
| TTTCGGAAGTGCCGTCGAGAGGGGTTTCG | 2[207] | 0[208] |
| GAGGGTAGGATTCAAAAGGGTGAGACATCCAA | 14[111] | 12[112] |
| TATTAAGAAGCGGGGTTTTGCTCGTAGCAT | 2[175] | 0[176] |
| GCCCTTCAGAGTCCACTATTAAAGGGTGCCGT | 21[64] | 23[63] |
| ATGCAGATACATAACGGGAATCGTCATAAAAAGCAAAG | 7[56] | 9[63] |
| AGCCAGCAATTGAGGAAGGTTATCATTTT | 22[207] | 20[208] |
| TAAATGAATTTTCTGTATGGGATTAATTTCTT | 0[111] | 1[95] |
| AAACAGCTTTGCGGGATCGTCAACACAAAA | 1[96] | 3[95] |
| CGGATTCTGACGACAGTATCGGCCAAGGCGATTAAGTT | 16[63] | 18[56] |
| GCAATTCACATATTCCTGATTATCAAAGTGTA | 19[160] | 20[144] |
| GCGCAGACAAGAGGCAAAAGAATCCCTCAG | 4[79] | 2[80] |
| AGAGAGAAAAAAAGAAAATAGCACACACAAACT | 9[160] | 10[144] |
| GACAAAAGGTAAATAATCGCCATATTTAACAAAACTTTT | 13[184] | 15[191] |
| ACACTCATCCATGTTACTTAGCCGAAAGCTGC | 3[96] | 5[95] |
| CTACCATAGTTTGAGTACATTTAAAATAT | 19[224] | 21[223] |
| TATATTTTGTCATTGCCTGAGAGTGGAAGATTGTATAAGC | 13[64] | 15[71] |
| CGGATTGCAGAGCTTAATTGCTGAAACGAGTA | 9[64] | 11[63] |
| TAAATCATATAACCTGTTTAGCTAACCTTTAA | 12[111] | 10[112] |
| GTACCGCAATTCTAAGAACGCGAGTATTATTT | 12[207] | 10[208] |
| TCTTCGCTGCCGCGCTGGTGCGGCCTTCC | 18[111] | 16[112] |
| GATTTAGTCAATAAAGCCTCAGAGAACCCTCA | 11[64] | 13[63] |
| GCAAGGCCTCACCAGTAGCACCATGGGCTTGA | 5[160] | 6[144] |
| ATTACCTTTGAATAAGGCTTGCCCAAATCCGC | 6[111] | 4[112] |
| CTTATCATTCCCGACTTGCGGGAGCCTATT | 12[239] | 10[240] |
| TTATACCACCAAATCAACGTAACGAACGAG | 6[79] | 4[80] |
| GTAATAAGTTAGGCAGAGGCATTTATGATATT | 13[160] | 14[144] |
| CAACCGTTTCAAATCACCATCAATTCGAGCCA | 14[143] | 13[159] |
| GATGGTTTGAACTAGTAAATTTACCATTA | 6[143] | 5[159] |
| GCACAGACAATTTTTGAATGGTCAGTA | 23[224] | 22[240] |
| AGCAAGCGTAGGGTTTTTTGTAGGGAGCC | 21[96] | 23[95] |
| TCCACAGACAGCCCTCATAGTTAGCGTAACGA | 0[175] | 0[144] |
| TCACCAGTACAAACTACAACGCCTAGTACCAG | 0[207] | 1[191] |
| ATTATACTAAGAAACCACCAGAAGTCAACAAGGT | 19[192] | 21[191] |
| TAAGAGCAAATGTTTAGACTGGATAGGAAGCC | 7[96] | 9[95] |
| ATACATACCGAGGAAACGCAATAAGAAGCGCATTAGACGG | 7[192] | 9[199] |
| CAACTGTTGCGCGCGCGCCATTCAAAACATCA | 18[143] | 17[159] |
| GATGGCTTATCAAAAGATTAAGAGCGTCC | 10[79] | 8[80] |
| TAGGTAAACTATTTTTGAGAGATCAAACGTTA | 13[96] | 15[95] |
| AGGCAAAGGGAAGCGATCGGCAATTCCA | 17[128] | 19[127] |
| ATTATCATTCAATAATCCTGACAATTAC | 20[175] | 18[176] |
| GAAATTATTGCCTTTAGCGTCAGACCGGAACC | 6[239] | 4[240] |
| AATGGTCAACAGGCAAGGCAAAGAGTAATGTG | 11[96] | 13[95] |
| CCAATAGCTCTCGTAGGAATCATGGCATCAA | 11[160] | 12[144] |
| ATACCCAACAGTATTAGCAAATTAGAGC | 8[175] | 6[176] |
| ATAAGGGAACCGGATATTCATTACGTCAGGACGTTGGGAA | 4[63] | 6[56] |
| CACCAGAAAGGTTGAGGCAGGTCATGAAAG | 4[175] | 2[176] |
| ATCCCAATGAGAATTAACTGAACAGTTACCAG | 10[207] | 8[208] |

| | | |
|---|---|---|
| CATGTAATAGAATAAAGTACCAAGCCGT | 14[175] | 12[176] |
| CCAACAGGAGCGAACCAGGGAGCCTTTAC | 10[143] | 9[159] |
| GCTATCAGAAATGCAATGCCTGAATTAGCA | 14[79] | 12[80] |
| GACCTGCTCTTTGACCCCCAGCGAGGGAGTTA | 4[111] | 2[112] |
| AGGAACCCATGTACCGTAACACTTGATAA | 0[239] | 1[223] |
| CAGCGAAACTTGCTCTGTGTTGCTAA | 2[79] | 0[80] |
| GCTTTCCGATTACGCGCGCGGCTCTTTC | 17[96] | 19[95] |
| ACAACTTTCAACAGTTTCAGCGGATGTATCGG | 0[79] | 1[63] |
| CAGCAAAAGGAAACCACCAATGAGCCGC | 6[175] | 4[176] |
| ACCTTTTTTTTTTTTTTTAATTTCATAGGGCTT | 16[207] | 14[208] |
| CGATAGCATTGAGCCATTTGGGAACGTAGAAA | 5[192] | 7[191] |
| GCCCGAGAGTCCACGCTGGTTTGCAGCTAACT | 22[111] | 20[112] |
| ATTTTAAAATCAAAATTATTTGCACGGATTCG | 20[239] | 18[240] |
| ACCTTGCTTGGTCAGTTGGCAAAGAGCGGA | 22[175] | 20[176] |
| CTGAGCAAAAATTAATTACATTTTGGGTTA | 18[175] | 16[176] |
| CCTGATTGCAATATATGTTGTGATCAATAGT | 18[239] | 16[240] |
| TCAATATCGAACCTCAAATATCAATTCCGAAA | 21[160] | 22[144] |
| AAAGGCCGGAGACAGCTAGCTGATAAATTAATTTTTGT | 13[120] | 15[127] |
| CTTTAGGGCCTGCAACAGTGCCAATACGTG | 21[224] | 23[223] |
| AATAGTAAACACTATCATAACCCTCATTGTGA | 8[111] | 6[112] |
| TCACCGACGCACCGTAATCAGTAGCAGAACCG | 6[207] | 4[208] |
| GCCCGTATCCGGAATAGGTATCAGCCCAAT | 2[239] | 0[240] |
| TGTAGCCATTAAAATTCGCATTAAATGCCGGA | 16[111] | 14[112] |
| TCGGCAAATCCTGTTGATGGTGGACCCTCAA | 22[143] | 21[159] |
| TGACAACTCGCTCTGCTTGCATTATACCA | 1[128] | 3[127] |
| CCACCCTCTATTCACAAACAAATACCTGCCTA | 4[207] | 2[208] |
| CCCGATTTAGAGCTTGACGGAAAAAAGAATA | 23[96] | 22[112] |
| AAGTAAGCAGACACCACGGAATAATATTGACG | 8[239] | 6[240] |
| AAATTAAGTTGACCATTAGATACTTTTGCG | 12[79] | 10[80] |
| CACATTAAAATTGTTATCCGCTCATGCGGGCC | 20[111] | 18[112] |
| TTAAAGCCAGAGCCGCCACCCTCGAGAGAGAA | 3[224] | 5[223] |
| ATATTCGGAACCATCGCCCACGCAGAGAAGGA | 2[143] | 1[159] |
| TTCTACTACGCGAGCTGAAAAGGTTACCGCGC | 12[143] | 11[159] |
| AACGTGGCGAGAAAGAGGGAAAACCAGTAA | 23[128] | 23[159] |
| GAATTTATTTAATGGTTTGAAATATTCTTACC | 16[239] | 14[240] |
| AGCGCGATGATAAATTGTCGTGACGAGA | 3[128] | 5[127] |
| AACGCAAAGATAGCCGAACAAACCCTGAAC | 7[224] | 9[223] |
| AGAAAACAAAGAAGATGATGAAACAGGCTGCG | 17[160] | 18[144] |
| CGCGCAGATTACCTTTTTTAATGGGAGAGACT | 18[207] | 16[208] |
| CACAACAGGTGCCTAATGAGTGCCCAGCAG | 19[128] | 21[127] |
| GCGGAACATCTGAATAATGGAAGGTACAAAAT | 20[207] | 18[208] |
| TAAAAGGGACATTCTGGCCAACAAAGCATC | 23[160] | 22[176] |
| AATTGAGAATTCTGTCCAGACGACTAAAACCAA | 14[207] | 12[208] |
| GCGAAAAATCCCTTATAAATCAAGCCGGCG | 21[128] | 23[127] |
| AACACCAAATTTCAACTTTAATCGTTTACC | 5[128] | 7[127] |
| TAAATCAAAAAGCGCGTCTCGGAAACC | 15[128] | 17[127] |
| GAAACGATAGAAGGCTTATCTGGTCTCATCGAGAACAAGC | 10[191] | 12[184] |
| GCCTCCCTCAGAATGGAAAGCGCAGTAACAGT | 4[239] | 2[240] |
| GCGAACCTCCAAGAACGGGTATGACAATAA | 11[224] | 13[223] |

| | | |
|---|---|---|
| TTAGGATTGGCTCTACTCCTCAATAACCGAT | 1[160] | 2[144] |
| ATCGCAAGTATGTAAATGCTGATGATAGGAAC | 15[160] | 16[144] |
| GCGGATAACCTATTATTCTGAAACACACGATT | 1[192] | 3[191] |
| AAGGAACATAAAGGGCAACATCIACG | 8[207] | 6[208] |
| ACCCTTCTGACCTGAAAGCGTAAGACGCTGAG | 23[192] | 22[208] |
| ATATTTTGGCTTTCATCAACATTATCCCCA | 15[96] | 17[95] |
| TCAAGTTTCATTAAAGGTGAATAAAAGA | 5[224] | 7[223] |
| TCTAAAGTTTTGTCGTCTTTCCAGCCGACAA | 0[143] | 1[127] |
| TTCCAGTCGTAATCATGGTCATAAAAGGGG | 20[79] | 18[80] |
| AAAGCACTAAATCGGAACCCTAATCCAGTT | 23[64] | 22[80] |
| AATACTGCCCAAAAGGAATTACGGCTCA | 8[79] | 6[80] |
| TTTATCAGGACAGCATCGGAGAGACACCAACCTAAAACGA | 1[64] | 3[71] |
| TTGACAGGCCACCACCAGAGCCGCGATTTGTA | 3[160] | 4[144] |
| CTGTGTGATTGCGTTGCGCTCACTAGAGTTGC | 19[96] | 21[95] |
| GCGAGTAAAAATTTAAATGTTACAAAG | 16[79] | 14[80] |
| TAGAGAGTTATTTTCATTTGGATAGTAGTAGCATTA | 10[127] | 12[120] |
| CGAAAGACTTTGATAAGAGGTCATATTTCGCA | 9[96] | 11[95] |
| TCATCGCCAACAAAGTACAACGGACGCCAGCA | 4[143] | 3[159] |
| TTAACACCAGCACTAACAACTAATCGTTATTA | 22[239] | 20[240] |
| TTATTACGAAGAACTGGCATGATTGCGAGAGG | 7[160] | 8[144] |
| GCCAGTTAGAGGGTAATTGAGCGCTTTAAGAAA | 10[239] | 8[240] |
| ACAACATGCCAACGCTCAACAGTCTTCTGA | 13[224] | 15[223] |
| CATTTGAAGGCGAATTATTCATTTTTTTTTTT | 17[192] | 19[191] |
| TGAAAGGAGCAAATGAAAAATCTAGAGATAGA | 21[192] | 23[191] |
| TGGAACACCGCCTGGCCCTGAGGCCCGCT | 22[79] | 20[80] |
| TACCGAGCTCTAAATTCGAAACCTGTCGCAGCTGATT | 19[56] | 21[63] |
| GTTTATTTTGTCACAATCTTACCGAAGCCCTATAATCA | 7[248] | 9[255] |
| ACAAACGGAAAACCCCAAAAACACTGGAGCA | 16[47] | 14[48] |
| GTTTATCAATATGCGTTATACAAACCGACCGTGTGATAAA | 13[256] | 15[263] |
| ACGGCTACAAAAGGAGCCTATAATGTGAGAAT | 2[47] | 0[48] |
| GACCAACTAATGCTACTACGAAGGGGGTAGCA | 4[47] | 2[48] |
| AAGGCCGCTGATACCGATAGTTGCGACGTTAG | 2[111] | 0[112] |
| CTCCAACGCAGTGAGACGGGCACCCCCCTGCA | 22[47] | 20[48] |
| ACCGATTGTCGGCATTTTCGGTCATAATCA | 6[271] | 4[272] |
| CAGAAGATTAGATAATACATTTGTCGACAA | 22[271] | 20[272] |
| TGCATCTCTCTCTAGTCACGACGGCGCAG | 17[32] | 19[31] |
| TTAGTATCACAATAGATAAGTCCACGAGCA | 14[271] | 12[272] |
| GTTTTAACTTAGTACCGCCCCCCCCCCA | 2[271] | 0[272] |
| TTAATGAACTAGAGGATCCCGGGGGGGAACG | 20[47] | 18[48] |
| CTTTTACAAAATCGTCGCTATTAGCGATAG | 18[271] | 16[272] |
| ATCCCCCTATACCACATTCAACTAGAAAAATC | 8[47] | 6[48] |
| AGAAAGGAACAACTAAAGGAATTCAAAAAAAA | 0[47] | 1[31] |
| TTTTATTTAAGCAAATCAGATATTTTTTGT | 12[175] | 10[176] |
| AGCCACCACTGTAGCGCGTTTTCAAGGGGAGAAGAAAA | 4[255] | 6[248] |
| AACAAGAGGGATAAAAATTTTTAGCATAAAGC | 14[47] | 12[48] |
| GCCGTCAAAAAAGAGGTGAGGCCTATTAGT | 21[256] | 23[255] |
| TGTAGAAATCAAGATTAGTTGCTCTTACCA | 12[271] | 10[272] |
| GAGAGATAGAGCGTCTTTCCAGAGGTTTTGAA | 9[256] | 11[255] |
| CCACCCTCTTTTCAGGGATAGCAACCGTACT | 0[271] | 1[255] |

| | | |
|---|---|---|
| CTTTAATGCGCGAACTGATAGCCCCACCAG | 23[256] | 22[272] |
| CCAGGGTTGCCAGTTTGAGGGGACCCGTGGGA | 18[47] | 16[48] |
| CAAATCAAGTTTTGGGGGAAACGTGGA | 23[32] | 22[48] |
| ACGCTAACACCACAAGAATTGAAAATAGC | 10[271] | 8[272] |
| CTTGTGCAGATAAAAAAAAATAAAGACTCC | 8[143] | 7[159] |
| TACGTTAAAGTACTTGAGAAGAACCGAACT | 6[47] | 4[48] |
| TAATCAGCGGATTGACCGTAATCGTAACCG | 15[32] | 17[31] |
| TTTTCACTCAAAGGGCGAAAAACCATCACC | 21[32] | 23[31] |
| GCCTTAAACCAATCAATAATCGGCACGCGCCT | 11[256] | 13[255] |
| AATAGCTATCAATAGAAATTCAACATTCA | 8[271] | 6[272] |
| CATCAAGTAAAACGAACTAACGAGTTGAGA | 5[32] | 7[31] |
| CAGGAGGTGGGGGGAGTGCCTTGAGTCTCTGAATTTACCG | 1[256] | 3[263] |
| AAATCACCTTCCAGTAAGCGTCAGTAATAAA | 4[271] | 2[272] |
| CTCGTATTAGAAATTGCGTAGATACTACTAC | 20[271] | 18[272] |
| TTTACCCCAACATGTTTTAAATTTCCATAT | 9[32] | 11[31] |
| CCTAAATCAAAATCATAGGTCTAAACAGTA | 15[224] | 17[223] |
| GTCGACTTCGGCCAACGCGGGGTTTTTC | 19[32] | 21[31] |
| CGTAAAACAGAAATAAAATCCTGTGCCCGAAAGATTAGA | 19[248] | 21[255] |
| AGGCTCGAGGCTTTGAGAGGGGAAA | 1[32] | 3[31] |
| GAGAAGAGATACTTGCTGTGGGGAGAAACAATAA | 16[255] | 18[248] |
| TTTAGGACAAATGCTTTAACAATCAGGTC | 7[32] | 9[31] |
| AATACGTTTGAAAGAGGACAGACTGACCTT | 3[32] | 5[31] |
| CTTAGATTTAAGGCGTTAAATAAGCCTGT | 16[271] | 14[272] |
| TAAATCGGGATTCCCAATTCTGCGATATAATG | 12[47] | 10[48] |
| AACAGTTTTGTACCAAAACATTTTATTTC | 11[32] | 13[31] |
| CTGTAGCTTGACTATTATAGTCTTCATTGA | 10[47] | 8[48] |
| AGACGACAAAGAAGTTTTGCCATAATTCGAGCTTCAA | 7[128] | 9[135] |
| AACGCAAAATCGATGAACGGTACCGGTTGA | 13[32] | 15[31] |

**Tabelle S17**. Unmodifizierte Staple-Stränge des 6HB DNA Origami. Sequenzen sind vom 5'- zum 3'-Ende angegeben. Die Zahlen für das 5'- Ende 3'-Ende der Staple-Stränge stellen die Helixnummer in der entsprechenden caDNAno-Datei dar. Die Zahl in Klammern stellt die Start- und Endposition des Staple-Strangs in der entsprechenden Helix dar.

| Unveränderte Staple-Stränge | 5'-Ende | 3'-Ende |
|---|---|---|
| TACAACGAAAGGAGGTAAAATACGTAATAGGCAAAAGAATTT | 0[685] | 3[685] |
| ATCTCTGACCTCCTAGTCGAAACCTGGCACGAATATAGTT | 4[195] | 0[182] |
| ACCTTTTTAACCCACATAGCTCTTAGTAGTGAATTTATC | 3[1232] | 1[1245] |
| ACCATCAGCGTCCAAATAGTAAAATGTTTAAGAGGCTTTTGC | 5[546] | 2[546] |
| ATCAATATCTGGTCAGTTGGCAAATC | 3[1372] | 3[1397] |
| GTGTCTCTCTCTCTCACTCATCGAGAACATTTACAAGAAA | 1[1120] | 4[1120] |
| TCAGAGGCATTGCAAACGATTTTTTTGAGAGAATAACATA | 0[1063] | 3[1063] |
| AACAGTTGAAAGGAATTGAGGAGAG | 2[1397] | 2[1372] |
| CTTTTCTTAAACAGTAACCACACCAAAGGCTCCAAACA | 4[741] | 0[728] |
| AACCGTTCTAGCAAAGGCCGGAGACAGTGATTCAAAAATCCT | 2[377] | 5[377] |
| TATCCTGACGCTCACTCTTATCTGGTATCGACTTGCGGGACA | 0[1105] | 3[1105] |
| GCTGCAAGGCGATGCCTCGCGCTATTAAGCGCGTGCGTAT | 2[251] | 5[251] |
| AGATTTAAGTCCACTTAATTGCTGAATAAACTAAAGTACGGG | 0[475] | 3[475] |
| GGCCAACAGAATATGAGGCATTTTCGAGAGCGCCATATTTAA | 5[1176] | 2[1176] |
| GCGCGTTTTCATCGAGCGAGAGAATCAAAGCAGCACCGAGTA | 2[923] | 5[923] |
| ATAACGTACACTGAGCCCAATAGGAACCATCCTCAGAGCCAC | 5[798] | 2[798] |

| | | |
|---|---|---|
| GGAGGCCAGGATTATAAGAGGCTGAGACTGTATTTCGGAACC | 5[840] | 2[840] |
| TGCCCAGCGATTATGTATATCATCCTGAAGAAAGGCGAAGGC | 1[658] | 4[658] |
| ATGCGCGATAGCCTCTTAGAATCCTTGACCTTGCTTCTGTA | 5[1260] | 2[1260] |
| TACGAGCCGGAAGCTAAATTCGTAAA | 1[142] | 1[167] |
| AAAGAGTCATCTTTTCATAGCCCCCTTAACCGTCAGACTGTA | 5[924] | 2[924] |
| AATAATTAATTTTCAGATTAAGACGCGCGCTATTAGAAAACAG | 1[1246] | 4[1246] |
| TATCCGGCTTAGGTAATTTTTGAATGAGAAGAGTCAATTA | 4[1245] | 0[1232] |
| CAATAATCGGCTAGAATATCCCATCCTAGTCCTGAACATTGG | 2[1133] | 5[1133] |
| CAACTCGTATTACAACTTTACAAACAATATGATTTAGAAGTA | 3[1358] | 1[1371] |
| AGAATAAGTTTATAACATCACTTGCCGTAGAAAAAAAAAA | 4[993] | 0[980] |
| TAATAAGAGAGATATTATACAATTCTTTTAATTGAGAATAAA | 0[1189] | 3[1189] |
| ACGCTGAGAGCCAGCAGCAAATGAAAAATCTAA | 5[1372] | 5[1404] |
| CAACGCCAACATGACTCAACTAGGGCACCAGTAACATTCT | 2[1175] | 5[1175] |
| AAAGCGCCATTCCGTGGCCGGAAACCCCTTTCCGGCACCG | 3[266] | 1[279] |
| TATTATTCTGAACGCGTATAACAGTTAGCCTTGACTAAAACA | 2[839] | 5[839] |
| TAAACCAACCAGTCTCAACAATAGATAAATTTACK | 0[1147] | 3[1147] |
| ATCGCGCACCAAATTGCGTAGATTTAGTACCTCTTACAT | 0[1315] | 3[1315] |
| TACCGTAGCTCTCCCGTACTCAGGAGGCAGACGCCACAA | 0[811] | 3[811] |
| TTGTTATCTAAAATCTTTAGGAGCACTA | 1[1372] | 1[1401] |
| ATTATGATAGCCCGTACTAATAGTAGTACAAAGAATTAGCGC | 0[433] | 3[433] |
| ACCGGCATTTTCGGTCATAATCAAAATCGAGGCCACCGTAAT | 1[910] | 4[910] |
| AATGGCATCAATTCAGATAGGGTTGAGTCGCAAATGGTCAAC | 4[447] | 0[434] |
| GTAAACAGGGCTTAAGCTAAA | 4[167] | 4[145] |
| GTAAATTGGGCTGAGAAACACAGAACGGAAGGCTTG | 3[602] | 1[615] |
| TAAAGCCTCAGAAATCATACAGGCAAGCATTAAATAAATC | 2[419] | 5[419] |
| AAAGCCTGTTTAGCGAATCATAATTACTCCATAAGAATAAAC | 3[1190] | 1[1203] |
| ATGGGCGCATCGTCGCGGATTGACCGTCTCCGTACGGGCA | 2[293] | 5[293] |
| GAGGCTTGCAGGTTCAACCATCGCCCACTTGCGGTGTAGC | 2[713] | 5[713] |
| AATCAGATATAGAATCGTCTGAAATGGAAGCACCGTTTT | 4[1119] | 0[1106] |
| ATCAATAACACGGAACGTTATTATAACAAAGAAACCGA | 0[1357] | 3[1357] |
| AAACATTAAATCGGAATTTCAACTTTAATTGGCTCATTATATA | 0[601] | 3[601] |
| GCGAACCAAAACCGAAATCAGGTCTTTATGCATCAAAAAA | 0[517] | 3[517] |
| TTGACGGATCAACGCAAGAACCGGATATCGCATAGGCTGGCT | 5[630] | 2[630] |
| CACCACCCTCAGAGTTTTTAATCAGTACCGGAACCAGACT | 4[909] | 0[896] |
| ACAGGAGTGTACCCTACATGGCTTGTGATCGTTCCAGTAAGC | 3[854] | 1[867] |
| CCTGTGAGCGAGTATTCGCCTGGCCCGCCATCAAAACG | 4[321] | 0[308] |
| CACCCTCATTTTACAGAACCGCCACCCTTTTATADCACGT | 2[797] | 5[797] |
| CCCTCCTGCGCGCTTGATACCGATAGGCATAACCGATAGC | 0[727] | 3[727] |
| AATAACGGATTCACAAATATACAGTAACTCAGGTTAAAAATAC | 2[1301] | 5[1301] |
| TAGCATAAAGCTAAATACTTTTGCGGGAATCCCTTCATCCAAA | 1[406] | 4[406] |
| TCGCCAGAGGGTATACTGCGGAATCGGGCCCACCCTCGTT | 1[532] | 4[532] |
| GAGCTGAAAGGCATATTTTCATTTGGGAAATAACCTGTTTA | 3[434] | 1[447] |
| GAAAGCGGAGATTTACTACAGCGCGAAACTTACAAAACAC | 5[672] | 2[672] |
| GGTCACGCAGCGAAGCTTGTGGGATCTTTATTCTCGCGCT | 5[714] | 2[714] |
| GGTGCCTAATGAGTCTACGGTGCTTGTT | 3[138] | 3[167] |
| TTTCGAGGTGAAGTATCGGTTTATCAGCGTAGGAGCCTTTAA | 3[728] | 1[741] |
| CTTAACCGTGCATCCTCGAAGATCGCCAGTGAGGGGAACA | 1[280] | 4[280] |
| TCAAACTATTACGCAGAACTGGCATGATCAAAACCGAGAAA | 5[1008] | 2[1008] |
| CCCGAAAGACTTTTGAGACAAAGCGGATCCCTGACACGTCAA | 2[503] | 5[503] |

| | | |
|---|---|---|
| ATGTAAATTGACTCACCGACTTGAGCCGTTGTATCATATG | 1[952] | 4[952] |
| TCACGACCAGGGGGGCGCAACTGTGGGGCCCCCTGGCGGC | 0[265] | 3[265] |
| ATAGCACACAAAATGAATCATTACCGCGATTTTATTTTCATC | 3[1106] | 1[1119] |
| GCTAATGCAGAACGCAATAAACAACATGGTTCTGTCCAGACG | 3[1148] | 1[1161] |
| CATGAGGAAGTTAAGAGGACTAAAGACTAACGGCTACAGAGG | 3[686] | 1[699] |
| ACTCCTTATCGGCCCTTGAAGCCCTGTTACCAGAAG | 0[1021] | 3[1021] |
| ATCAACATTAAACCCTCTGTAGCCAGCGATAATTCGCGTCT | 3[308] | 1[321] |
| CCAAAGTTTTGTCGCGTACTATGGTTGCACAAAACTACAACTA | 4[783] | 0[770] |
| TTGGGAAGAAAAAGCGATTTTAAGAACCATTGTGGCCGTAA | 2[587] | 5[587] |
| AGTGAGATGGTTTAACCCTAAAGGGAGCATTCAGTGAATAAC | 4[615] | 0[602] |
| GCACATGAAAGTATGGATTAGCGGGGTTGCGGGAGGTAACAG | 1[826] | 4[826] |
| AAAGCAATAGCTCTTTGCTGGTAATATCAGAGATAACCCAAG | 4[1035] | 0[1022] |
| TTGCACGTAAAACACATCAAAACAATGGAAGGGTTA | 3[1316] | 1[1329] |
| TTCTTTGATCACCGGGAAATTATTCATTCGATTCAACCGATT | 5[966] | 2[966] |
| GTATGGCTTAGAGCTATTAAAGAACGTGAGGTCAGGATTAGT | 4[489] | 0[476] |
| AGCATCACCTCTCTGAACCTCAAATATCAAACC | 4[1404] | 4[1372] |
| CAGATTCAGTACCGCATTCCAAGAACGGTTGTAGAAACCAAT | 5[1134] | 2[1134] |
| AGGCATGTCAATCACTGGTGCCCCAGTCATTGCCTGAGTA | 4[363] | 0[350] |
| TCAAATATATTTCGAAATCCAATCGCAAATGTAGTAAGAAA | 2[1217] | 5[1217] |
| TGGGCGCGTTGTAAACGCCAGGGTTTTCAGAAAGGGGGATGT | 5[252] | 2[252] |
| ACCGGTCAATACACGGCGAACGTGGCGTAAATTGTGTCGAT | 4[657] | 0[644] |
| GCATCCAATAAGACAGGAAAAAACGCTCGTCTTTCCAGAGAAG | 4[1077] | 0[1064] |
| CTCACATTAATTGCGTGCGCTC | 5[145] | 5[167] |
| ACGCTCGCCCCTCTCACTCCCGCCAAGAATTGTAGCTGCA | 2[209] | 5[209] |
| CGGGTACCGAGCATAAAGTGTAAAGCCTGG | 2[167] | 2[138] |
| GGGAGTGACTCTCTCAACTCGTGTGGTCGTGCCCAACCTT | 1[196] | 4[196] |
| TGTGAATTCATGGGGATGTTCTTCTAAGGGAGGAGAAGCCAG | 3[224] | 1[237] |
| TAGGGAGAATTAACAGCGCTAATATCAGCAGAACAAAAAAAATG | 1[1036] | 4[1036] |
| GCAACAGTTGATTCCATTAGATACATTTTGTTCGTAGCTC | 1[448] | 4[448] |
| ATCGTAAAACTAAAGAGAATCGATGACGTAGTCTGGAGCAA | 3[350] | 1[363] |
| CATTAAAAGAAGTGCCACCCTCAGACAGCATTGACAGCA | 0[895] | 3[895] |
| GTGAAACGTCACCACCACGCAAATTAACCATTTGGGAATTAG | 4[951] | 0[938] |
| GTTTGATAGATCTAGCCGGAGAGGGTAGTCAATATGATATTC | 5[378] | 2[378] |
| AGTGAGAATAGAGTATGGGATTTTGCTAAGTAATTTAATGC | 2[755] | 5[755] |
| CTGAGTTAAGGCCAGAGCATCGGAACTGGCAAGACAATG | 1[700] | 4[700] |
| AAGACACCACGGCAACATATAAAAGAAAAAATACATAAA | 3[980] | 1[993] |
| ACGACAGATTGCCCACAACCCGTCGGATGGGATAGCTC | 0[307] | 3[307] |
| GCCAAAAGGAATATCTAATGCAGATACATAGGAATACCACAT | 3[560] | 1[573] |
| CGTTTGCCTGTCCAATGAAACCATCGATGTTTGTTTAGCA | 0[937] | 3[937] |
| TTCATTCCATATTATGTTTTAAATGCTAATGCTCAGTTG | 2[461] | 5[461] |
| AATCCTGGCCAGAACACAAACACAAAAAGCGAGGTTGAGGCA | 5[882] | 2[882] |
| GAGGGAGGGAAGCACACCGCCAAGAAGAAAAATGCAATAC | 2[965] | 5[965] |
| GCCGCTACACGTTGAGAGAATTGCGAATGTCAGTTTCAGCGG | 5[756] | 2[756] |
| TAACCATAAATCAATCTATCAGGGCGATTCATAAATATCAA | 4[531] | 0[518] |
| CCCTCAGAACCGGCTCTCCCTCAGAGCCTCGCCACCACCGGA | 3[896] | 1[909] |
| CTGCCATATCGGCCGTGTCCTTAGTGCTAATAACCCCGCTGT | 0[223] | 3[223] |
| AATTAATTACATCAAACATCAAGAAAAAAAGAAGATGAT | 3[1274] | 1[1287] |
| TACGTGGGAAATACTCTGTGTAAAGAGAGAAAACTTTT | 5[1218] | 2[1218] |
| CATTTTGAATCTTATGCCCAGCTACACCGGTTGAAGCC | 5[1092] | 2[1092] |

| | | |
|---|---|---|
| AACAAATATCGCGTAAGCAAACTCCAACGACTATATTATA | 1[490] | 4[490] |
| TGATAGGTGTATCATCGTTAGAATCAGATTGCTCAGTACCTG | 4[825] | 0[812] |
| GAATATTCCTGATTGATTGTTTGGATTAGAGGTTTGAGT | 1[1330] | 4[1330] |
| ACGAGTAATCTTGATAACAAAGCTGCTCCCCCGATTTGAAAG | 1[616] | 4[616] |
| CGCAATAATAACCAGATAGCCGAACAAATTTTAAGGAAGAAC | 2[1007] | 5[1007] |
| AACAGAAATAAAGAGCAGAAGATAAAACTACTTCTGAATAAA | 4[1329] | 0[1316] |
| ACAGCTGTATCGGCTGCCAGTTTGGGGTTACGTTGGTGTAG | 5[294] | 2[294] |
| CGCGCACGACTTAAAAACGCGGGGAGACCAAGCTTTCTCCC | 4[237] | 0[224] |
| AGTAGTTGCTATTTCCAACGCTAACGAGCATGGAAACGCGAG | 1[1078] | 4[1078] |
| TTAAGGAACAACTAAAAATCTCCAAAAAGCCGCGCGAATTTT | 1[742] | 4[742] |
| GCAAGCGTCATTTTCGCATTAAATTTTTTTTTTTAAAATTGT | 5[336] | 2[336] |
| AAACACAATGAAATAACCCAATAAGAGTACAAGAATTGAGT | 3[1022] | 1[1035] |
| TACAATGCCTGAGTCCGAAATCGGCAAAGAAGTTTATTAT | 4[405] | 0[392] |
| ATGGTTTCACAGACTGGGTTATATAACTGACAAAGAACGCCT | 0[1231] | 3[1231] |
| ATTTTTTCAGGGCGTCTCAGACGTTAACAACTTTCAATA | 0[769] | 3[769] |
| AGTTAACAATTTCAAGCCCTAAAACATCATTACCTCACAAA | 4[1287] | 0[1274] |
| TTAATGACTGTAAGGATACCGACAGTGCTGTCTCTAATCTATTT | 5[210] | 2[210] |
| AAAAGAACCCTGTAATCGGTGTACCAAGCAAAATTAAGCAA | 5[420] | 2[420] |
| ACTCCATTAAACGGCGGGCTAGGGCGCGGTAGCAAAG | 4[699] | 0[686] |
| CATTTCTCCGAAGAGACGCATTTCACATGTGGGCCTTGAATC | 3[182] | 1[195] |
| TTTACGAGGCATAGCAAGTTTTTTGGGTTGAGATTTAG | 4[573] | 0[560] |
| TTTTGAGGGTGGTTAATGTGTAGGTAAACAAATCACCATCAT | 0[391] | 3[391] |
| ACTGATAAATTAATCAAAGTATCAGGCAGGCGAAAGGGTG | 1[364] | 4[364] |
| ACGTAATTTAGGCAAAAGTACCGACAAAAAAGGGTAAAGCC | 1[1162] | 4[1162] |
| TACCCAAGGAAAGCAGGGAACCGAACTGAGAGACACCAGGCGGA | 0[643] | 3[643] |
| CATGGTAATAAGTTGGGATTTTAGACAGTCTGAATTTACCCT | 4[867] | 0[854] |
| AGCACTATTACGAGAAAACGAACTAACGAGCCAGTCAGGACG | 5[588] | 2[588] |
| GTATAGCCCGGCTCGAGAGGTGTGATCAAGGCGGATAAGT | 3[812] | 1[825] |
| TCATCTACGTTAATTAGAAAGATTCTCGAGGTAATTACC | 1[574] | 4[574] |
| AATCAGCGTCCACGTATGTACCCCGGTGTGTATAAGCAAATA | 0[349] | 3[349] |
| GGAATTATCATCACTTATTTTGTGCGGTTAAAGAGGCGGT | 2[1343] | 5[1343] |
| AACGCGCCTTTAACACGACCTAATAGGTAAAAAAAAA | 4[1161] | 0[1148] |
| AAGCCATTCAGGCTTTTTTTTTCACACCCAGCCAGCAG | 4[279] | 0[266] |
| TTAGCAAGGCCGTTCCAGTAGCACCABCABTTTAGAGCCAGCAAA | 3[938] | 1[951] |
| ACGAGGCGCAGACACCATTACTTAGCTCTCAAATCCGCGACC | 3[644] | 1[657] |
| CGCCAGCGTAATTGTGAACACCCTGAACCATAAAAAACACAGGGA | 5[1050] | 2[1050] |
| TCATTTTTGCGCCAGCTCCTTTTGATAAGAGAGAGTACCTTT | 3[476] | 1[489] |
| ACTGCCCGAAATTGTCATGGTCATAGCTAAACGGAGGATCCC | 5[168] | 2[168] |
| TCATCTTTGACCCTACCTAAAACGAAGGCCACTAAAGGGAA | 2[671] | 5[671] |
| GAACAAGGTTTGACCCAATTCTGCGAACGAGTGTCTGGAAGT | 5[462] | 2[462] |
| CCTGTGTGCTTTCCGGTTGGTGTAATGAACCTCGATAAAGAT | 0[181] | 3[181] |
| CTGGATACCCAAATTAGAGCAACACTAACCAAAATAGCGAC | 0[559] | 3[559] |
| AAACGTTAATATCCAAAACACAGGAAGATGATAATCAGTTGCA | 2[335] | 5[335] |
| AAAAGAAGTTTTAAAGACGACGATAAAATCATAACTACGTGA | 2[545] | 5[545] |
| ATGTATCATATGCGGAACCCTGTGACCATAAGGCGTTAAAG | 4[1203] | 0[1190] |
| GGTTAAGTTGGGTAAACGACGGCGCGGGCGGTTATCGGTG | 1[238] | 4[238] |
| CGAACGAAGAGGCGTGAATACCAAGTTATTATCGGGAGAAAC | 5[1302] | 2[1302] |
| GGTTTGTTAAAATTAACCAATAGGAACTGAGAGAGAAAAG | 1[322] | 4[322] |
| AATCGTCGCTATAATAATCAATATATGTTTAATGGTCTTTA | 2[1259] | 5[1259] |

| | | |
|---|---|---|
| GTGAATTATTAGTGTGTCACAATCAAAAGGGCGACCA | 0[979] | 3[979] |
| CATAGCGAACTGATTTTGAATTACCTTTTTTTGAATAACAA | 0[1273] | 3[1273] |
| GAGCCTGATTGCTTAATTATTCATTTCAGCCATTATAACGTC | 1[1288] | 4[1288] |
| AAAACGAGAATGCCATGCTATAACACAGTCAATGAATCCCCC | 3[518] | 1[531] |
| GGGGAATACCCAAAAGTATTAGCAAACTGAGTAAAAAAA | 1[994] | 4[994] |
| ACTAGTTAATTTCACGACCGTGTGATAATGAAAGCAATGCTG | 1[1204] | 4[1204] |
| CTAATCCTTTGCCCCCTGCAACAGTGCCAATACATTTGAGTC | 4[1371] | 0[1358] |
| CAGTATTTAATCCTATCAGATGATGGCATCACCAGAGAGAGC | 5[1344] | 2[1344] |
| GGTCACACCACACAGAGCCGCCGCCGCCAACGCCAG | 2[881] | 5[881] |
| ACAACTAATAGATTAGAGCCGTCAATAGAT | 0[1401] | 0[1372] |
| CCCAGGGATAGCAAGTGTCACCAGTTTTGACGCCGCCAC | 1[784] | 4[784] |
| GTGGCCTTGATTTGGAAAGCGCAGTCGAACGGTCCAGAAC | 1[868] | 4[868] |
| TTATTTATCCCAGTACAAAATAAACAGCACACCTAATTTGCC | 3[1064] | 1[1077] |
| TTATCCGCTCACAATTCCACACAACACACA | 0[167] | 0[142] |
| GCGTAACGATCTTCAGACCCCTCATAAAGCCTGTAGCATT | 3[770] | 1[783] |
| GACCTTCATCAAGACAGATGAACGGTGTACAACTTTAGAGC | 2[629] | 5[629] |
| CAAGAGAGATTAAATAACGGTCAGTATGCCCCCTGCCAT | 0[853] | 3[853] |
| ATATTTTAAATGAAAATTTTTAGAACCCCTTCAACGCAGAGA | 3[392] | 1[405] |
| TTAAATCAAGATTTTTTAGCGAACCTCCTCTAAGAATACCTA | 2[1091] | 5[1091] |
| AGGGCGAAGACCGGTTTAATTCCTTTCATTAAGAGGAAG | 5[504] | 2[504] |
| AGCGCATTAGACAGTAGCAGCCTTTACATAACGTCATATTAC | 2[1049] | 5[1049] |

## Verweise


[1] N. D. Derr, B. S. Goodman, R. Jungmann, A. E. Leschziner, W.M. Shih, S. L. Reck-Peterson, *Science* **2012**, *338*,662-665.
[2] S. Woo, P. W. Rothemund, *Nat Chem* **2011**, *3*,620-627.
[3] R. Schreiber, S. Kempter, S. Holler, V. Schüller, D. Schiffels, S. S. S. Simmel, P.C. Nickels, T. Liedl, *Small* **2011**, *7*,1795-1799.
[4] P.C. Nickels, B. Wünsch, P. Holzmeister, W. Bae, L.M. Kneer, D. Grohmann, P. Tinnefeld, T. Liedl, *Science* **2016**, *354*,305-307.
[5] T. Cordes, J. Vogelsang, P. Tinnefeld, *J Am Chem Soc* **2009**, *131*,5018-5019.
[6] R. Roy, S. Hohng, T. Ha, *Nat Methods* **2008**, *5*,507-516.
[7] A. D. Edelstein, M. A. Tsuchida, N. Amodaj, H. Pinkard, R. D. Vale, N. Stuurman, *2014* **2014**.
[8] A. Edelstein, N. Amodaj, K. Hoover, R. Vale, N. Stuurman, *Current Protocols in Molecular Biology* **2010**, *92*, 14.20.11-14.20.17.
[9] J. Schnitzbauer, M. T. Strauss, T. Schlichthaerle, F. Schueder, R. Jungmann, *Nature Protocols* **2017**, *12*, 1198-1228.
[10] M. T. Strauss, F. Schueder, D. Haas, P.C. Nickels, R. Jungmann, *Nature Communications* **2018**, *9*, 1600.
[11] J. Vogelsang, R. Kasper, C. Steinhauer, B. Person, M. Heilemann, M. Sauer, P. Tinnefeld, *Angewandte Chemie International Edition* **2008**, *47*,5465-5469.
[12] Y. Zhang, Q. Li, X. Liu, C. Fan, H. Liu, L. Wang, *Small* **2020**, *16*,2000793-2000793.
[13] F.C. Simmel, B. Yurke, H. R. Singh, *Chemical Reviews* **2019**, *119*, 6326-6369.
[14] D. Y. Zhang, E. Winfree, *Journal of the American Chemical Society* **2009**, *131*,17303-17314.


## Autorenbeiträge

C. Forthmann, V. Glembockyte, M. Scheckenbach und P. Tinnefeld haben die Experimente entworfen. M. Scheckenbach hat Experimente und Datenanalysen durchgeführt. M. Scheckenbach und T. Schubert haben DNA-PAINT-Experimente und Analysen von immobilisierten Nanolinealen unter degradierenden Bedingungen durch FBS-Lösung durchgeführt. C. Forthmann, V. Glembockyte und P. Tinnefeld haben die Studie betreut. V. Glembockyte, M. Scheckenbach und P. Tinnefeld haben das Manuskript geschrieben. Alle Autoren haben das Manuskript geprüft und genehmigt.
